# Diretriz sobre Ecocardiografia de Estresse – 2026

**DOI:** 10.36660/abc.20260223

**Published:** 2026-05-04

**Authors:** Ana Cristina Camarozano, Salvador Vicente Spina, José Roberto Matos Souza, Marcelo Dantas Tavares de Melo, Daniela do Carmo Rassi Frota, Silvio Henrique Barberato, Fabio Villaça Guimarães, Harry Acquatella, Vera Márcia Lopes Gimenes, Jorge Alfredo Lowenstein, Ricardo H Pignatelli, José Maria Del Castillo, Joselina Luiza Menezes Oliveira, José Sebastião de Abreu, José Luis de Castro e Silva Pretto, Issam Shehadeh, Alex dos Santos Felix, Mercedes Maldonado Andrade, Antônio Carlos Sobral Sousa, Maria Estefânia Bosco Otto, Andrea de Andrade Vilela, Pedro Gutierrez Fajardo, Liz Andréa Villela Baroncini, Cristiano Vieira Machado, Fábio Cañellas Moreira, José María Hernández Hernández, Marco Stephan Lofrano Alves, Samira Saady Morhy, Márcio Silva Miguel Lima, Miguel Osman Dias Aguiar, Wilson Mathias, Letícia dos Santos de Oliveira Rocha, João César Nunes Sbano, Barbara Athayde Linhares Martins Vrandecic, Fabio Roston, Arnaldo Rabischoffsky, Juliana Paixão Etto Rolim, Raphael Aparecido Barreto da Silva, Guilherme de Rossi, Kamila Fernanda Staszko, Jolie Britt, Marcelo Luiz Campos Vieira

**Affiliations:** 1 Universidade Federal do Paraná Curitiba PR Brasil Universidade Federal do Paraná, Curitiba, PR – Brasil; 2 Hospital Aeronáutico Central Buenos Aires BA Argentina Hospital Aeronáutico Central, Buenos Aires, BA – Argentina; 3 Centro Médico Don Bosco Ramos Mejía Pcia BA Argentina Centro Médico Don Bosco, Ramos Mejía Pcia, BA – Argentina; 4 Universidade de Campinas Campinas SP Brasil Universidade de Campinas, Campinas, SP – Brasil; 5 Universidade Federal da Paraíba João Pessoa PB Brasil Universidade Federal da Paraíba, João Pessoa, PB – Brasil; 6 Hospital das Clinicas da Universidade Federal de Goiás Goiânia GO Brasil Hospital das Clinicas da Universidade Federal de Goiás, Goiânia, GO – Brasil; 7 Pontifícia Universidade Católica do Paraná Curitiba PR Brasil Pontifícia Universidade Católica do Paraná, Curitiba, PR – Brasil; 8 Instituto do Coração de Marília Marilia SP Brasil Instituto do Coração de Marília, Marilia, SP – Brasil; 9 Centro Medico de Caracas Caracas CCS Venezuela Centro Medico de Caracas, Caracas, CCS – Venezuela; 10 Hospital do Coração São Paulo SP Brasil Hospital do Coração, São Paulo, SP – Brasil; 11 Investigaciones Medicas Buenos Aires Buenos Aires BA Argentina Cardiodiagnóstico, Investigaciones Medicas Buenos Aires, Buenos Aires, BA – Argentina; 12 Texas Children's Hospital- Baylor College of Medicine Houston TX Estados Unidos da América Texas Children's Hospital- Baylor College of Medicine, Houston, TX – Estados Unidos da América; 13 Escola de Ecografia de Pernambuco Recife PE Brasil Escola de Ecografia de Pernambuco, Recife, PE – Brasil; 14 Universidade Católica de Pernambuco Recife PE Brasil Universidade Católica de Pernambuco, Recife, PE – Brasil; 15 Universidade Federal de Sergipe Aracaju SE Brasil Universidade Federal de Sergipe, Aracaju, SE – Brasil; 16 Clinicardio de Fortaleza e Cardioexata Fortaleza CE Brasil Clinicardio de Fortaleza e Cardioexata, Fortaleza, CE – Brasil; 17 Hospital São Vicente de Paulo Passo Fundo RS Brasil Hospital São Vicente de Paulo, Passo Fundo, RS – Brasil; 18 Clínica Cardiovision Esteio RS Brasil Clínica Cardiovision, Esteio, RS – Brasil; 19 Instituto Nacional de Cardiologia Rio de Janeiro RJ Brasil Instituto Nacional de Cardiologia, Rio de Janeiro, RJ – Brasil; 20 Diagnósticos da América S.A. (DASA-RJ) Rio de Janeiro RJ Brasil Diagnósticos da América S.A. (DASA-RJ), Rio de Janeiro, RJ – Brasil; 21 Complexo Hospitalar Americas Rio de Janeiro RJ Brasil Complexo Hospitalar Americas, Rio de Janeiro, RJ – Brasil; 22 Instituto Dante Pazzanese de Cardiologia São Paulo SP Brasil Instituto Dante Pazzanese de Cardiologia, São Paulo, SP – Brasil; 23 Hospital São Lucas Rede D'Or de Aracaju Aracaju SE Brasil Hospital São Lucas, Rede D'Or de Aracaju, Aracaju, SE – Brasil; 24 Universidade de Brasília Brasília DF Brasil Universidade de Brasília, Brasília, DF – Brasil; 25 Hospital Israelita Albert Einstein São Paulo SP Brasil Hospital Israelita Albert Einstein, São Paulo, SP – Brasil; 26 Hospital Lefort Morumbi São Paulo SP Brasil Hospital Lefort Morumbi, São Paulo, SP – Brasil; 27 Hospital de Especialidades San Francisco de Asis Guadalajara JAL México Hospital de Especialidades San Francisco de Asis, Guadalajara, JAL – México; 28 Cardiotest Guadalajara JAL México Cardiotest, Guadalajara, JAL – México; 29 Laboratorio de Ecocaerdiografia Guadalajara JAL México Laboratorio de Ecocaerdiografia, Guadalajara, JAL – México; 30 Hospital Nossa Senhora Das Gracas Curitiba PR Brasil Hospital Nossa Senhora Das Gracas, Curitiba, PR – Brasil; 31 Fleury Medicina e Saúde São Paulo SP Brasil Fleury Medicina e Saúde, Grupo Fleury, São Paulo, SP – Brasil; 32 Santa Casa De Misericórdia De Porto Alegre Porto Alegre RS Brasil Santa Casa De Misericórdia De Porto Alegre, Porto Alegre, RS – Brasil; 33 Imaging Cardiac Department. Doctors Hospital Auna Monterrey NL México. Imaging Cardiac Department. Doctors Hospital Auna, Monterrey, NL – México.; 34 Instituto Do Coração do Hospital das Clínicas da Faculdade de Medicina da Universidade de São Paulo São Paulo SP Brasil Instituto Do Coração do Hospital das Clínicas da Faculdade de Medicina da Universidade de São Paulo, São Paulo, SP – Brasil; 35 Hospital Beneficência Portuguesa de São Paulo São Paulo SP Brasil Hospital Beneficência Portuguesa de São Paulo, São Paulo, SP – Brasil; 36 Hospital Pequeno Príncipe Curitiba PR Brasil Hospital Pequeno Príncipe, Curitiba, PR – Brasil; 37 Grupo Fleury Medicina e Saúde São Paulo SP Brasil Grupo Fleury Medicina e Saúde, São Paulo, SP – Brasil; 38 Hospital Biocor/Rede D'or Belo Horizonte MG Brasil Hospital Biocor/Rede D'or, Belo Horizonte, MG – Brasil; 39 Clínica Clinicardio Londrina PR Brasil Clínica Clinicardio, Londrina, PR – Brasil; 40 Hospital Norte Paranaense HONPAR Arapongas PR Brasil Hospital Norte Paranaense HONPAR, Arapongas, PR – Brasil; 41 Grupo Cura (Labimagem e Ultramed) Londrina PR Brasil Grupo Cura (Labimagem e Ultramed), Londrina, PR – Brasil; 42 Hospital Pró-Cardíaco Rio de Janeiro RJ Brasil Hospital Pró-Cardíaco, Rio de Janeiro, RJ – Brasil; 43 Hospital Sírio Libanê São Paulo SP Brasil Hospital Sírio Libanê, São Paulo, SP – Brasil; 44 Alta (Rede Dasa) São Paulo SP Brasil Alta (Rede Dasa), São Paulo, SP – Brasil; 45 Centro Especializado em Cardiologia LTDA Campinas SP Brasil Centro Especializado em Cardiologia LTDA, Campinas, SP – Brasil; 46 Complexo do Hospital de Clínicas da Universidade Federal do Paraná (CHC-UFPR) Curitiba PR Brasil Complexo do Hospital de Clínicas da Universidade Federal do Paraná (CHC-UFPR), Curitiba, PR – Brasil; 47 Baylor College of Medicine Houston TX Estados Unidos da América Baylor College of Medicine, Houston, TX – Estados Unidos da América; 48 Texas Children's Hospital Houston TX Estados Unidos da América Texas Children's Hospital, Houston, TX – Estados Unidos da América; 49 Faculdade de Medicina da Universidade de São Paulo São Paulo SP Brasil Faculdade de Medicina da Universidade de São Paulo, São Paulo, SP – Brasil

**Table:** 

Diretriz sobre Ecocardiografia de Estresse – 2026	
O relatório abaixo lista as declarações de interesse conforme relatadas à SBC pelos especialistas durante o período de desenvolvimento deste posicionamento, 2025/2026.	
Especialista	Tipo de relacionamento com a indústria
Alex dos Santos Felix	Nada a ser declarado
Ana Cristina Camarozano	Nada a ser declarado
Andrea de Andrade Vilela	Nada a ser declarado
Antônio Carlos Sobral Sousa	Nada a ser declarado
Arnaldo Rabischoffsky	Nada a ser declarado
Barbara Athayde Linhares Martins Vrandecic	Nada a ser declarado
Cristiano Vieira Machado	Nada a ser declarado
Daniela do Carmo Rassi Frota	Declaração financeira A - Pagamento de qualquer espécie e desde que economicamente apreciáveis, feitos a (i) você, (ii) ao seu cônjuge/ companheiro ou a qualquer outro membro que resida com você, (iii) a qualquer pessoa jurídica em que qualquer destes seja controlador, sócio, acionista ou participante, de forma direta ou indireta, recebimento por palestras, aulas, atuação como proctor de treinamentos, remunerações, honorários pagos por participações em conselhos consultivos, de investigadores, ou outros comitês, etc. Provenientes da indústria farmacêutica, de órteses, próteses, equipamentos e implantes, brasileiras ou estrangeiras: - AstraZeneca do Brasil Ltda: Forxiga.
Fábio Cañellas Moreira	Nada a ser declarado
Fabio Roston	Declaração financeira A - Pagamento de qualquer espécie e desde que economicamente apreciáveis, feitos a (i) você, (ii) ao seu cônjuge/ companheiro ou a qualquer outro membro que resida com você, (iii) a qualquer pessoa jurídica em que qualquer destes seja controlador, sócio, acionista ou participante, de forma direta ou indireta, recebimento por palestras, aulas, atuação como proctor de treinamentos, remunerações, honorários pagos por participações em conselhos consultivos, de investigadores, ou outros comitês, etc. Provenientes da indústria farmacêutica, de órteses, próteses, equipamentos e implantes, brasileiras ou estrangeiras: - Boston scientific: Proctoria de oclusão de apêndice atrial esquerdo.
Fabio Villaca Guimarães filho	Nada a ser declarado
Guilherme De Rossi	Outros relacionamentos Participação societária de qualquer natureza e qualquer valor economicamente apreciável de empresas na área de saúde, de ensino ou em empresas concorrentes ou fornecedoras da SBC: - Área da Saúde
Harry Acquatella	Nada a ser declarado
Issam Shehadeh	Declaração financeira A - Pagamento de qualquer espécie e desde que economicamente apreciáveis, feitos a (i) você, (ii) ao seu cônjuge/ companheiro ou a qualquer outro membro que resida com você, (iii) a qualquer pessoa jurídica em que qualquer destes seja controlador, sócio, acionista ou participante, de forma direta ou indireta, recebimento por palestras, aulas, atuação como proctor de treinamentos, remunerações, honorários pagos por participações em conselhos consultivos, de investigadores, ou outros comitês, etc. Provenientes da indústria farmacêutica, de órteses, próteses, equipamentos e implantes, brasileiras ou estrangeiras: - GE Healthcare: Handson Ecocardiografia de Estresse. Outros relacionamentos Financiamento de atividades de educação médica continuada, incluindo viagens, hospedagens e inscrições para congressos e cursos, provenientes da indústria farmacêutica, de órteses, próteses, equipamentos e implantes, brasileiras ou estrangeiras: - Torrent: Cardiologia; Medley Sanofi : Zinpass Eze.
João Cesar Nunes Sbano	Nada a ser declarado
Jolie Britt	Nada a ser declarado
Jorge Alfredo Lowenstein	Nada a ser declarado
José Luis de Castro e Silva Pretto	Nada a ser declarado
José Maria Del Castillo	Nada a ser declarado
José María Hernández Hernández	Outros relacionamentos Financiamento de atividades de educação médica continuada, incluindo viagens, hospedagens e inscrições para congressos e cursos, provenientes da indústria farmacêutica, de órteses, próteses, equipamentos e implantes, brasileiras ou estrangeiras: - Vitalmex: LVAD – Vitacor.. Vínculo empregatício com a indústria farmacêutica, de órteses, próteses, equipamentos e implantes, brasileiras ou estrangeiras, assim como se tem relação vínculo empregatício com operadoras de planos de saúde ou em auditorias médicas (incluindo meio período) durante o ano para o qual você está declarando: - Vitalmex: LVAD – Vitacor.
José Roberto Matos Souza	Nada a ser declarado
José Sebastião de Abreu	Nada a ser declarado
Joselina Luzia Menezes Oliveira	Nada a ser declarado
Juliana Paixão Etto Rolim	Nada a ser declarado
Kamila Fernanda Staszko	Nada a ser declarado
Letícia dos Santos de Oliveira Rocha	Nada a ser declarado
Liz Andréa Villela Baroncini	Nada a ser declarado
Marcelo dantas tavares de melo	Nada a ser declarado
Marcelo Luiz Campos Vieira	Nada a ser declarado
Márcio Silva Miguel Lima	Nada a ser declarado
Marco Stephan Lofrano Alves	Nada a ser declarado
Maria Estefânia Bosco Otto	Nada a ser declarado
Mercedes Maldonado Andrade	Nada a ser declarado
Miguel Osman Dias Aguiar	Outros relacionamentos Participação societária de qualquer natureza e qualquer valor economicamente apreciável de empresas na área de saúde, de ensino ou em empresas concorrentes ou fornecedoras da SBC: - Área da Saúde
Pedro Gutierrez-Fajardo	Nada a ser declarado
Raphael Aparecido Barreto da Silva	Declaração financeira B - Financiamento de pesquisas sob sua responsabilidade direta/pessoal (direcionado ao departamento ou instituição) provenientes da indústria farmacêutica, de órteses, próteses, equipamentos e implantes, brasileiras ou estrangeiras: - Lilly: obesidade, lipoproteína(a); Amgen: insuficiência cardíaca, lipoproteína(a), doença arterial coronariana; Sanofi: fibrilação atrial; Vertex: insuficiência renal; Boehringer: insuficiência cardíaca; AstraZeneca: dislipidemia.
Ricardo H Pignatelli	Nada a ser declarado
Salvador Vicente Spina	Nada a ser declarado
Samira Saady Morhy	Nada a ser declarado
Silvio Henrique Barberato	Declaração financeira A - Pagamento de qualquer espécie e desde que economicamente apreciáveis, feitos a (i) você, (ii) ao seu cônjuge/ companheiro ou a qualquer outro membro que resida com você, (iii) a qualquer pessoa jurídica em que qualquer destes seja controlador, sócio, acionista ou participante, de forma direta ou indireta, recebimento por palestras, aulas, atuação como proctor de treinamentos, remunerações, honorários pagos por participações em conselhos consultivos, de investigadores, ou outros comitês, etc. Provenientes da indústria farmacêutica, de órteses, próteses, equipamentos e implantes, brasileiras ou estrangeiras: - Pfizer: Amiloidose; AstraZeneca: ICFEP. Outros relacionamentos Financiamento de atividades de educação médica continuada, incluindo viagens, hospedagens e inscrições para congressos e cursos, provenientes da indústria farmacêutica, de órteses, próteses, equipamentos e implantes, brasileiras ou estrangeiras: - Pfizer: Amiloidose.
Vera Marcia Lopes Gimenes	Nada a ser declarado
Wilson Mathias Junior	Nada a ser declarado

## Sumário

**1. O Papel da Ecocardiografia de Repouso Realizada no Momento da Ecocardiografia de Estresse Físico ou Farmacológico** 9
**2. A Evolução da Ecocardiografia de Estresse: do Princípio aos Dias de Hoje** 9
**3. Métodos Provocativos de Isquemia** 9
  **3.1. Ecocardiografia de Estresse com Exercício (Bicicleta, Esteira Ergométrica ou Ciclomaca)** 10
  **3.2 Ecocardiografia de Estresse Farmacológico** 10
  **3.3. Ecocardiografia de Estresse no Espasmo Coronário** 10
  **3.4. Ecocardiografia de Estresse com Marca-Passo** 11
**4. Ecocardiografia de Estresse Físico (Esteira, Bicicleta e Ciclomaca)** 11
**5. Ecocardiografia de Estresse com Dobutamina na Avaliação de Isquemia Miocárdica** 13
  **5.1. Protocolo da Ecocardiografia de Estresse Farmacológico com Dobutamina** 13
  **5.2. Tipo de Resposta Miocárdica** 16
  **5.3. Avaliação Durante a Ecocardiografia de Estresse Farmacológico com Dobutamina e na Recuperação** 18
  **5.4. Dilatação Isquêmica Transitória do Ventrículo Esquerdo** 18
  **5.5. Comparação com Outras Modalidades de Imagem** 19
  **5.6. Valor da Ecocardiografia de Estresse Farmacológico com Dobutamina Negativa** 19
  **5.7. Complicações da Ecocardiografia de Estresse Farmacológico com Dobutamina** 19
**6. Ecocardiografia de Estresse com Dipiridamol** 20
  **6.1. Protocolos** 20
  **6.2. Efeitos Colaterais** 21
  **6.3. Contraindicações** 21
  **6.4. Reserva de Fluxo Coronariano** 21
  **6.5. Acurácia** 21
**7. Ecocardiografia de Estresse com Adenosina ou Regadenoson** 22
  **7.1. Uso Durante a Ecocardiografia de Estresse** 23
    **7.1.1. Indicações** 23
  **7.2. Protocolo para Ecocardiografia de Estresse com Adenosina** 24
  **7.3. Protocolo para Ecocardiografia de Estresse com Regadenoson** 24
  **7.4 Contraindicações** 24
  **7.5. Efeitos Colaterais** 24
  **7.6. Acurácia Diagnóstica** 24
**8. Outras Modalidades de Ecocardiografia de Estresse: Marca-Passo, Ergonovina, *Handgrip* e Hiperventilação** 24
  **8.1. Ecocardiografia de Estresse por Marca-Passo** 24
  **8.2. Associação do *Handgrip* à Ecocardiografia de Estresse** 25
  **8.3. Ecocardiografia de Estresse com Ergonovina e Hiperventilação** 25
**9. Ecocardiografia de Estresse Diastólico** 25
  **9.1. Resposta Normal e Anormal ao Exercício na Função Diastólica** 25
  **9.2. Indicações para Ecocardiografia de Estresse** 25
  **9.3. Protocolos Utilizados na Ecocardiografia de Estresse Diastólico** 26
  **9.4. Interpretação da Ecocardiografia de Estresse Diastólico** 26
**10. Ecocardiografia de Estresse no MINOCA (Ausência de Coronariopatia Obstrutiva) e na Doença Microvascular** 26
  **10.1. Fisiopatologia** 26
  **10.2. Principais Métodos Diagnósticos no MINOCA** 26
  **10.3. Ecocardiografia de Estresse no MINOCA** 28
**11. Ecocardiografia de Estresse na Doença Valvar Mitral** 29
  **11.1. Estenose Mitral** 30
  **11.2. Regurgitação Mitral** 30
  **11.3. Protocolo de Exame – Ecocardiografia de Esforço para Avaliação de Lesão Valvar Mitral** 31
**12. Ecocardiografia de Estresse na Doença Valvar Aórtica** 31
  **12.1. Estenose Aórtica** 31
  **12.2. Insuficiência Aórtica** 33
**13. Ecocardiograma de Estresse na Cardiomiopatia Dilatada** 33
**14. Ecocardiografia de Estresse na Cardiomiopatia Chagásica** 35
**15. Ecocardiografia de Estresse na Cardiomiopatia Hipertrófica** 36
**16. Ecocardiografia de Estresse na Avaliação da Função do Ventrículo Direito e na Hipertensão Pulmonar** 37
**17. Ecocardiografia de Estresse na Avaliação Pós-COVID-19** 37
**18. Ecocardiografia de Estresse e Reserva de Fluxo Coronariano nos Vasos Nativos** 38
**19. Ecocardiografia de Estresse e Reserva de Fluxo Coronariano em Enxertos** 39
  **19.1. Ecocardiografia de Estresse após Revascularização Miocárdica** 39
  **19.2. Reserva de Fluxo Coronariano após Revascularização Miocárdica** 39
**20. Ecocardiografia de Estresse na Análise de Viabilidade Miocárdica** 41
  **20.1. Protocolo de Estresse para Análise de Viabilidade Miocárdica** 41
  **20.2. Análise da Viabilidade Miocárdica pelo *Strain* Longitudinal** 42
**21. Ecocardiografia de Estresse com Agente de Realce Ultrassonográfico na Análise da Borda Endocárdica** 42
  **21.1. Agente de Realce Ultrassonográfico** 43
    **21.1.1. Forma de Administração** 43
**22. Ecocardiografia de Estresse com Agente de Realce Ultrassonográfico na Análise da Perfusão Miocárdica** 45
**23. Ecocardiografia de Estresse com Strain Miocárdico na Cardiopatia Isquêmica** 46
  **23.1. Strain derivado do speckle tracking versus *strain* baseado no Doppler tecidual durante ecocardiografia de estresse** 46
  **23.2. Ecocardiografia de estresse com *strain* 2D: Valor diagnóstico e prognóstico na doença arterial coronariana** 47
  **23.3. Qual *strain* pode ser útil na ecocardiografia de estresse: global ou regional, longitudinal ou circunferencial? E qual a importância do espessamento pós-sistólico?** 48
  **23.4. O *strain* tem memória isquêmica?** 49
  **23.5. O *strain* longitudinal global basal pode predizer doença arterial coronariana na ausência de anormalidade parietal visual sob estresse?** 51
  **23.6. Limitações** 51
**24. Ecocardiografia de Estresse com Strain Miocárdico para Além da Cardiopatia Isquêmica** 52
**25. Ecocardiografia de Estresse Tridimensional** 53
  **25.1. A Tecnologia da Ecocardiografia Tridimensional e sua Aplicação na Imagem do Ecocardiograma** 53
  **25.2. A Aplicação da Ecocardiografia Tridimensional Junto à Ecocardiografia de Estresse** 53
**26. A Ecocardiografia de Estresse na Sala de Emergência: Indicações e Aplicações** 55
  **26.1. Utilidade da Ecocardiografia de Estresse na Emergência** 55
**27. Ecocardiografia de Estresse no Pós-Transplante Cardíaco** 57
**28. Ecocardiografia de Estresse em Subgrupos Especiais (Bloqueio de Ramo Esquerdo, Bloqueio do Ramo Direito e Fibrilação Atrial)** 58
  **28.1. Bloqueio de Ramo Esquerdo** 58
  **28.2. Bloqueio do Ramo Direito** 58
  **28.3. Fibrilação Atrial** 58
**29. Ecocardiografia de Estresse no Pré-Operatório de Cirurgia Vascular, em Mulheres e Idosos** 59
  **29.1. Pré-Operatório de Cirurgia Vascular** 59
  **29.2. Mulheres** 59
  **29.3. Idosos** 60
**30. Ecocardiografia de Estresse na Pediatria: Indicações e Protocolos** 60
**31. Vantagens e Desvantagens da Ecocardiografia de Estresse em Pediatria** 62
**32. Prognóstico da Ecocardiografia de Estresse** 63
**33. Novas Aplicações da Ecocardiografia de Estresse no Pós-Radioterapia de Tórax e no Vasoespasmo** 64
**34. Ecocardiografia de Estresse com o Protocolo ABCDE** 64
  **34.1. Protocolo ABCDE** 65
**35. Recomendações PARA o Manejo da Ecocardiografia de Estresse** 67
**Referências** 68

**Figure:**
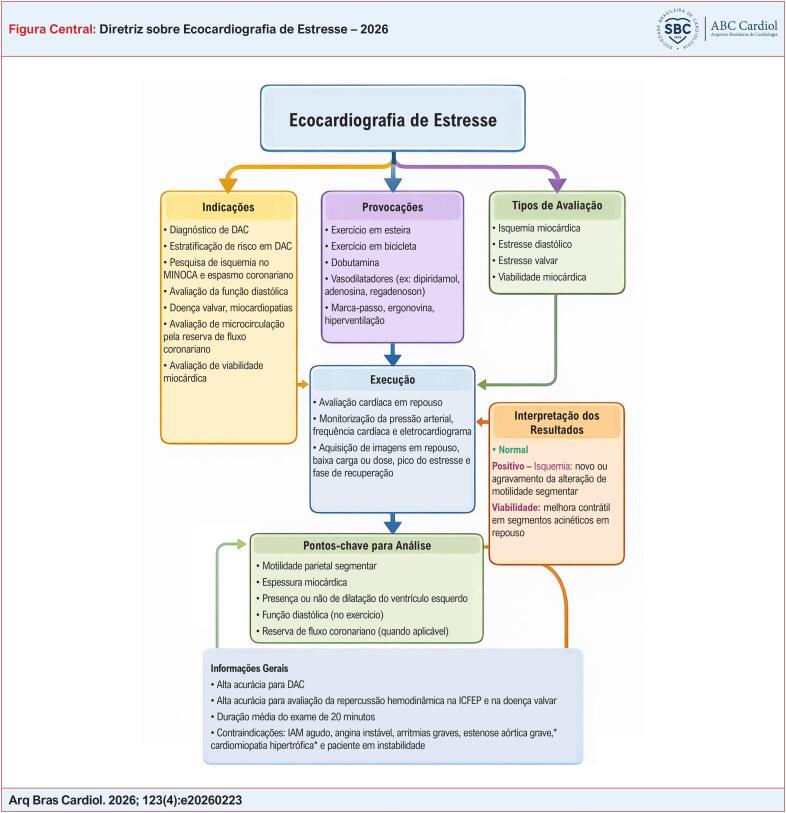
* Pode ser considerado em situações específicas.

## 1. O Papel da Ecocardiografia de Repouso Realizada no Momento da Ecocardiografia de Estresse Físico ou Farmacológico

Uma das primeiras descrições do uso da ecocardiografia de estresse de esforço (EEE), em 1979, comparou imagens de repouso com aquelas obtidas sob esforço em bicicleta.^1^ Quando analisamos as imagens do artigo, ficam claras as limitações da época, com a dificuldade de interpretar imagens em repouso e ao esforço devido à sua definição. Mesmo assim, alterações segmentares observadas em 10 pacientes foram encontradas naqueles com estenoses coronárias importantes. O método evoluiu desde então e o exame de repouso passou a contar com ferramentas diagnósticas mais apuradas, como Doppler em diversas modalidades, gravação digital de ciclos com alta taxa de quadros, medidas confiáveis de volumes cavitários, análise da deformação de ventrículos e átrios, estimativas das pressões cavitárias, análise de áreas valvares e vários marcadores de risco passíveis de análise ao repouso. Essa análise ampla e complexa em repouso, com avaliação morfológica e funcional completa, estabeleceu o exame de repouso como modalidade isolada.

Picano et al., em um consenso europeu recente,^2^ definiram a ecocardiografia de repouso, realizada junto ao eco estresse, com funções restritas, como análise da qualidade das imagens, mapeamento das janelas padrão, exclusão de outras razões óbvias para os sintomas (por exemplo, derrame pericárdico, estenose aórtica, cardiomiopatia hipertrófica) e sobretudo, eliminação de contraindicações ao exame com estresse, como isquemia aguda, dissecção da aorta e embolia pulmonar, entre outras. Como definida nesse consenso e em outras diretrizes,^3^ a ecocardiografia de repouso realizada imediatamente antes da ecocardiografia de estresse não é completa, pois tem a finalidade de observar os parâmetros que podem sofrer alterações dinâmicas. Portanto, um olhar direcionado à indicação clínica do método e às alterações em repouso que devem ser avaliadas sob estresse não pode ser utilizada ou requerida como avaliação morfológica e funcional completa de uma ecocardiografia padrão.

Assim, a ecocardiografia convencional de repouso é um exame diferenciado e deve ser solicitada, preferencialmente, em outro momento. Mais do que isso, trata-se do exame anterior e de primeira linha para afastar as situações que limitam, contraindicam ou impedem a realização da ecocardiografia de estresse, como os exemplos citados acima, e a própria imagem do paciente, que pode ser limitada ao ultrassom, comprometendo a avaliação da ecocardiografia de estresse.

## 2. A Evolução da Ecocardiografia de Estresse: do Princípio aos Dias de Hoje

A doença arterial coronariana (DAC) continua a ser uma causa importante de morbimortalidade e, portanto, recursos diagnósticos e terapêuticos capazes de reduzir esse panorama permanecem objetos de grande interesse na área médica, do ponto de vista individual e comunitário, com impacto socioeconômico relevante.

A existência de métodos diagnósticos acessíveis, de fácil execução, boa reprodutibilidade, baixo custo, preferencialmente não invasivos e com boa acurácia sempre interessaram às linhas de pesquisa na cardiologia, especialmente no que se refere à doença coronariana.

O uso de fármacos para ocasionar o desequilíbrio entre oferta e consumo de oxigênio inicialmente era associado à eletrocardiografia com o uso de dipiridamol, posteriormente substituído pela dobutamina. Porém, o uso desses fármacos associados à ecocardiografia se estabeleceu a partir dos estudos iniciais de Picano et al., que utilizavam dipiridamol, e de Berthe e Piérard em 1986, que utilizavam dobutamina.^5^ Na prática clínica diária, esses fármacos prevaleceram em termos de uso, com protocolos de dose bem definidos, critérios diagnósticos e níveis de segurança estabelecidos, muitas vezes associados ao uso da atropina, com a finalidade de maximizar os níveis de frequência cardíaca (FC) e, assim, otimizar os índices diagnósticos.^6-13^

A opção pelo esforço físico, seja ele realizado em esteiras ou bicicletas ergométricas ou em cicloergômetros, por ser um método mais fisiológico, continua a ser a modalidade de estresse de primeira escolha para pacientes com capacidade física e condições para a realização do esforço. Em pacientes com limitações ao exame sob esforço físico, como aqueles com sequelas ortopédicas ou neurológicas, pneumopatias, limitações visuais ou incoordenação para execução da atividade física, entre outras, a alternativa é o uso de protocolos farmacológicos.^7^

Tanto a modalidade de esforço físico quanto os testes farmacológicos se mostraram, dados os resultados obtidos nas últimas décadas, seguros para a avaliação de coronariopatas, idosos, mulheres, pacientes renais crônicos, valvopatas, transplantados e portadores de doenças vasculares periféricas. Ambos apresentam índices diagnósticos valiosos, semelhantes aos métodos da medicina nuclear, e com altos índices de concordância com métodos anatômicos estruturais, como a angiotomografia e a cineangiocoronariografia.^2,3,14,16^

Deve-se ressaltar que, além da finalidade de detecção de isquemia miocárdica, a ecocardiografia sob estresse é extremamente valiosa para a pesquisa de viabilidade miocárdica em pacientes com evento prévio, conceito fisiopatológico de importância diagnóstica, terapêutica e prognóstica para os portadores de coronariopatia.^10-12^

Por fim, observa-se que, além dos limites da doença coronariana, o método já é amplamente indicado na avaliação de alterações da função diastólica, especialmente entre pacientes com insuficiência cardíaca com fração de ejeção preservada (ICFEp), cardiomiopatias não isquêmicas (doenças de depósito e infiltrativas), avaliação de doenças valvares mitral e aórtica, estudo de cardiopatias congênitas, hipertensão pulmonar, avaliação do coração do atleta, avaliação de doença vascular do enxerto e no pós-transplante, entre outros.^2,3,14-20^

## 3. Métodos Provocativos de Isquemia

O objetivo principal da EEE na DAC é demonstrar o desenvolvimento da isquemia miocárdica, sua extensão e distribuição e, secundariamente, a estratificação de risco por meio de alterações hemodinâmicas (dilatação ventricular, queda da pressão arterial [PA], disfunção diastólica, alteração da reserva de fluxo coronariano [RFC] e/ou reserva miocárdica [RM], entre outras).^21^

**Figura 3.1:**
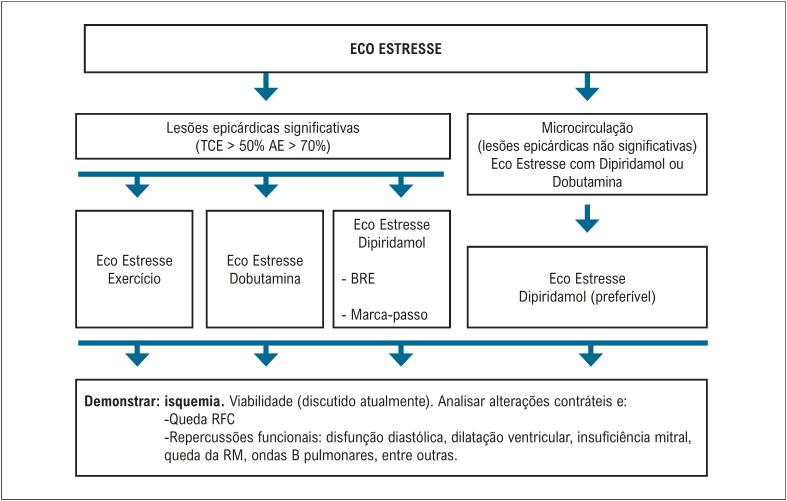
Objetivos anatômicos e funcionais da ecocardiografia de estresse. TCE: tronco da coronária esquerda; AE: artéria epicárdica; RFC: reserva de fluxo coronariano; RM: reserva miocárdica; BRE: bloqueio de ramo esquerdo.

A isquemia miocárdica pode ser evidente na presença de lesões epicárdicas obstrutivas importantes (artéria coronária principal esquerda (tronco da coronária esquerda) > 50% ou lesões epicárdicas > 70%) ou na presença de microcirculação comprometida sem lesões epicárdicas importantes. A utilidade de cada teste se baseia na detecção dessas anormalidades^22^ (Figura 3.1).

Também é interessante buscar a queda do fluxo em lesões intermediárias não importantes, sem isquemia em testes convencionais, por meio da medida da RFC.^23^

### 3.1. Ecocardiografia de Estresse com Exercício (Bicicleta, Esteira Ergométrica ou Ciclomaca)

É o mais fisiológico dos testes provocativos, baseado na demonstração de isquemia por meio do aumento do duplo produto (PA sistólica [PAS] máxima × FC máxima alcançada) e do consumo de oxigênio miocárdico, e considerado a primeira escolha (exceto em caso de contraindicações ou de indisponibilidade do centro). É mais útil em lesões epicárdicas importantes, acometendo principalmente a camada subendocárdica, avaliada pela motilidade parietal (espessamento), extensão e distribuição, com risco significativo quando o envolvimento é de três ou mais segmentos. Além disso, permite a observação simultânea das repercussões hemodinâmicas da isquemia: disfunção diastólica (fluxo transmitral, E/e’), dilatação ventricular, insuficiência mitral, queda da reserva miocárdica, arritmias e linhas B pulmonares, entre outras.

### 3.2 Ecocardiografia de Estresse Farmacológico

- Dobutamina: baseia-se na demonstração de isquemia em lesões epicárdicas importantes, por meio do aumento do duplo produto e do consumo de oxigênio miocárdico pela ação nos receptores adrenérgicos que aumenta o inotropismo e cronotropismo cardíacos. É utilizado em pacientes que não conseguem realizar exercício, quando a EEE não está disponível ou em pacientes com anormalidades de motilidade regional basal em busca de isquemia e viabilidade (este último ainda em discussão). Tem a mesma utilidade da EEE.
- Vasodilatadores (dipiridamol e adenosina): atuam na microcirculação, demonstrando isquemia por meio da vasodilatação direta das arteríolas saudáveis, em detrimento das arteríolas doentes, causando o "efeito de roubo de fluxo da artéria coronária com obstrução". Isso ocorre pelo acúmulo da adenosina no sistema vascular (de modo indireto com o dipiridamol e direto com adenosina exógena), cuja ação vasodilatadora é muito potente. Esses fármacos não aumentam significativamente o duplo produto, o que permite uma avaliação mais prática da RFC e do *strain* miocárdico. São úteis em pacientes com bloqueio completo do ramo esquerdo (BCRE), marca-passo permanente, contraindicação para exercício ou dobutamina, suspeita de doença microvascular, fibrilação atrial, hipertensão arterial e sintomáticos com lesões epicárdicas.

### 3.3. Ecocardiografia de Estresse no Espasmo Coronário

- Teste de ergonovina: é o padrão ouro devido à sua alta sensibilidade (93%) e especificidade (91%). Estimula os receptores α-adrenérgicos.^24-26^
- 5-hidroxitriptamina (serotonina): tem alto risco e não é indicada para uso rotineiro, de modo que não obteve reprodutibilidade clínica. As alterações contráteis que indicam isquemia são precoces, antes mesmo da dor ou de alterações eletrocardiográficas, e podem induzir a síndrome de Takotsubo.^27^
- Teste de hiperventilação: provoca espasmo coronário por meio da diminuição do íon H+ no plasma com aumento do pH (alcalose) e do aumento do cálcio intracelular, causando uma contração e vasoespasmos das células musculares lisas das artérias coronárias epicárdicas. Sua sensibilidade é alta (90%).^28,29^
- Teste pressórico ao frio (*cold pressor test*): na presença de disfunção endotelial e elevação de catecolaminas, ocorre vasoconstrição coronariana.^28,29^
- A combinação da ecocardiografia de estresse físico (EEF) ou dobutamina associado ao teste pressórico ao frio e/ou à hiperventilação pode aumentar a sensibilidade e o poder diagnóstico do vasoespasmo coronariano.

### 3.4. Ecocardiografia de Estresse com Marca-Passo

**Figura 3.2:**
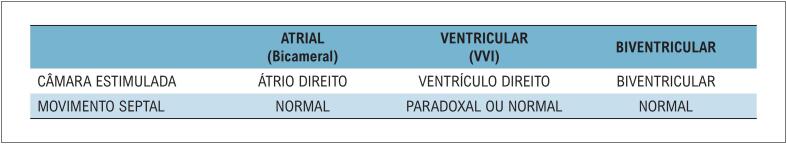
Ecocardiografia de estresse por marca-passo de acordo com a câmara estimulada e movimento septal.

Utilizado em pacientes portadores de marca-passo definitivo (MCPD), com estimulação de átrio direito e/ou ventrículo direito. O mecanismo é o aumento da FC, que aumenta o duplo produto e causa isquemia. A avaliação do espessamento regional leva em consideração os movimentos septais causados pelo local de implantação do cateter marca-passo (Figura 3.2).

É altamente sensível (98%) e seguro (96%), pois pode reduzir instantaneamente a FC.^30,31^

Trata-se de uma boa alternativa diagnóstica de isquemia em pacientes que não podem realizar exercício, têm contraindicação ao teste farmacológico ou portam marca-passo (Figura 3.2).

## 4. Ecocardiografia de Estresse Físico (Esteira, Bicicleta e Ciclomaca)

A EEF é um exame que investiga a funcionalidade do miocárdio de forma eficaz, fisiológica e segura, procurando reproduzir o dia a dia de indivíduos com capacidade física preservada. De maneira prática e não invasiva, permite obter informações valiosas sobre o diagnóstico de isquemia miocárdica, comportamento da PA sistêmica, etiologia de arritmias cardíacas e avaliação do comportamento dos gradientes de pressão da via de saída do ventrículo esquerdo (VSVE), da PAS pulmonar e de lesões valvares. É importante ressaltar que a análise desses parâmetros deve ser correlacionada com a possível ocorrência de sintomas antes, durante e após o esforço físico.^3^ Nas últimas duas décadas, foi demostrada a utilidade dessa metodologia para o manejo de portadores de diversas patologias, tais como, coronariopatias, cardiomiopatias, valvopatias, arritmias cardíacas, entre outras, sendo útil também na estratificação de risco no pré-operatório de procedimentos cirúrgicos não cardíacos.^3^

Frente às novas modalidades de imagens cardíacas, como angiotomografia das coronárias (ATC) e a ressonância magnética do coração (RNM-C), a EEF, surpreendentemente, evoluiu bastante. Embora a RNM-C tenha demonstrado a capacidade indiscutível de diagnosticar, de forma detalhada, diversas patologias cardíacas, a acurácia da RNM-C no diagnóstico de isquemia miocárdica não é superior à da EEF ou à da cintilografia do miocárdio. E por ser uma metodologia de fácil acesso, baixo custo e alta reprodutibilidade, a EEF é de grande utilidade para o acompanhamento dos portadores dessas cardiopatias.^33^ É importante que as novas gerações de ecocardiografistas também se interessem pelo método, tanto como exame útil na investigação clínica, como na pesquisa científica, buscando sempre se aprimorar na obtenção de imagens de qualidade satisfatória de modo a não comprometer a interpretação dos resultados.^7^

Inicialmente, o ecocardiografista deve avaliar as condições clínicas do paciente, bem como a sua preferência entre esteira, bicicleta ergométrica ou ciclomaca, caso o serviço disponha de mais de uma opção. Também é importante discutir as limitações das ferramentas estressoras, de forma que a escolha seja embasada na capacidade física do paciente, assim como nas condições locais de cada serviço. É importante, ainda, que o paciente seja informado que a EEF é composta de quatro componentes: eletrocardiografia, teste ergométrico, ecocardiografia transtorácica direcionada e ecocardiografia de estresse, considerando também a sintomatologia e capacidade física do paciente. Cabe ao ecocardiografista ficar atento à execução de todas as etapas do exame: o repouso, o pré-teste, a fase de esforço e, por fim, a fase de recuperação. A presença de paramédicos durante todo o exame também se faz necessária, para que não sejam comprometidas as gravações dos traçados eletrocardiográficos e as imagens ecocardiográficas.

Na maioria dos serviços, a esteira ergométrica constitui o carro-chefe, sobretudo por ser a metodologia de preferência da maioria dos pacientes. Com ela, o ecocardiografista experiente consegue, na maioria das vezes, que o paciente atinja a FC máxima preconizada (220 − idade), sobretudo se conscientizado dessa necessidade. Contudo, a imagem ao ecocardiograma muitas vezes fica prejudicada quando o esforço máximo é alcançado devido à presença do pulmão pela hiperventilação, então recomenda-se alcançar uma frequência intermediária entre a submáxima (85% da máxima) e a máxima para a boa interpretação das imagens, uma vez que são estas que definirão o teste e devem ser obtidas, idealmente, até 90 segundos após o esforço máximo (na esteira ergométrica). Essa conduta facilita a aquisição e a gravação das imagens ecocardiográficas, no pico do esforço físico, com o paciente já deitado na maca, em decúbito lateral esquerdo, o que deve ser obtido rapidamente após a aquisição da FC almejada, para não reduzir a sensibilidade do teste. Já quando a bicicleta ergométrica é utilizada, o momento da parada do exercício é quase sempre definido pelo paciente (desde que alcance a FC almejada para que o teste seja conclusivo), e a captação de imagem, realizada durante o esforço e na posição sentada, é mais laborioso para o ecocardiografista. Isso levou à invenção da ciclomaca, que facilita a captação de imagens, uma vez que o indivíduo se encontra em posição semissupina durante todo o exame.^32^ Nesse caso, a posição com maior elevação da ciclomaca permite um teste de melhor qualidade, dada maior propulsão das pernas que o paciente obtém.

**Figura 4.1:**
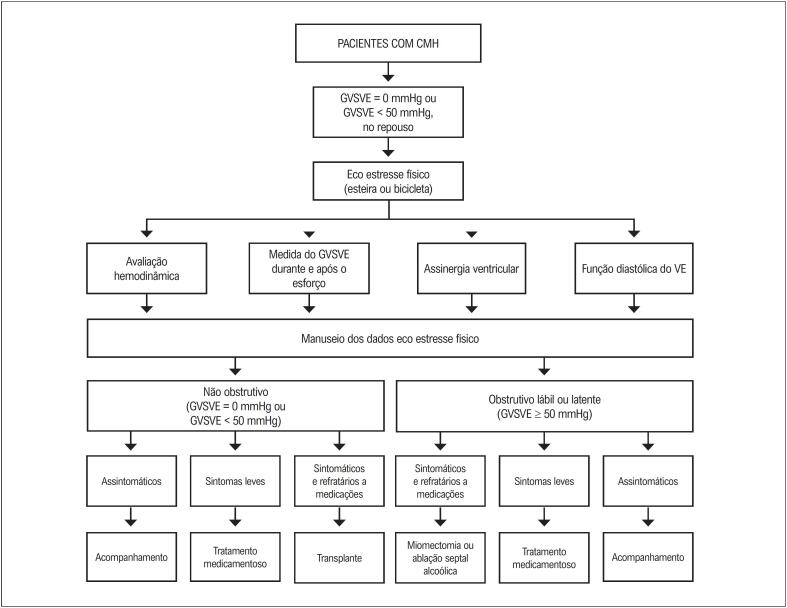
Fluxograma proposto para utilização de ecocardiografia de estresse físico em pacientes com CMH sem gradiente (GVSVE = 0 mmHg) ou com GVSVE (< 50 mmHg). CMH: cardiomiopatia hipertrófica; GVSVE: gradiente da via de saída do ventrículo esquerdo; VE: ventrículo esquerdo.

Atualmente, além das indicações clássicas da EEF que envolvem os pacientes que podem se exercitar e referem dor torácica, DAC suspeita ou conhecida, cansaço ou suspeita de ICFEp, as diretrizes da American Heart Association de cardiomiopatia hipertrófica (CMH) de 2021^33^ recomendam a avaliação inicial da medida do gradiente de VSVE de todos os pacientes com CMH em repouso, durante a manobra de Valsalva, nas posições semissupina, sentada ou ereta. Para pacientes assintomáticos e com gradiente de VSVE de repouso < 50 mmHg, indica-se a realização da EEF, que considera CMH obstrutiva quando o gradiente supera 50 mmHg. O gradiente de VSVE lábil ou obstrutivo é definido como aquele não observado durante a ecocardiografia em repouso, mas induzido pelo teste de esforço. Também é importante ressaltar que um gradiente de VSVE ≥ 50 mmHg em repouso contraindica a EEF. Além disso, é preciso avaliar a presença de movimento sistólico anterior da valva mitral, regurgitação mitral, assinergia ventricular e disfunção diastólica grau 3.^34^ (Figura 4.1).

**Figura 4.2:**
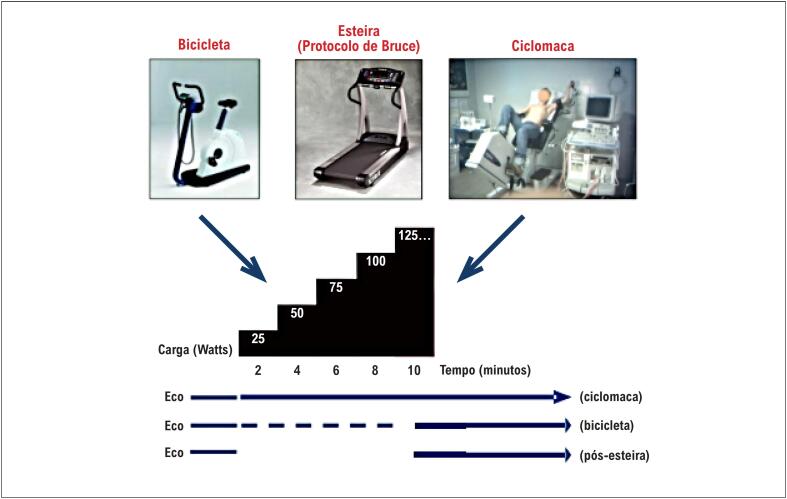
Protocolos da ecocardiografia de estresse físico nas modalidades de bicicleta ergométrica, esteira ergométrica e ciclomaca (supina). A captura da imagem na bicicleta e na ciclomaca é contínua, enquanto na esteira ergométrica ocorre somente antes e após o esforço imediato. O protocolo de Bruce ainda é muito utilizado na esteira ergométrica, enquanto na bicicleta ou ciclomaca utiliza-se preferencialmente o protocolo de Balke com incremento de carga a cada 2 minutos (incrementos de 25 W), conforme figura,^2^ podendo ir além de 125W.

Os protocolos da EEF nas modalidades de bicicleta ergométrica, esteira ergométrica e ciclomaca se encontram na Figura 4.2.

## 5. Ecocardiografia de Estresse com Dobutamina na Avaliação de Isquemia Miocárdica

Na isquemia miocárdica, a base da ecocardiografia de estresse farmacológico com dobutamina (EED) é a avaliação da resposta fisiológica do miocárdio a diferentes graus de obstrução da artéria coronária, especialmente na perfusão subendocárdica, enquanto a camada subepicárdica é afetada apenas em estágio posterior, se a isquemia persiste.^35,36^

A EED é uma modalidade alternativa para avaliação de isquemia miocárdica quando o paciente não consegue realizar exercício físico, inicialmente desenvolvida para avaliação de pacientes com DAC conhecida ou suspeita.^37^ A indicação mais frequente da EED é para avaliação da presença, localização ou extensão de isquemia miocárdica com especificidade de 88% que resulta em alto valor preditivo negativo para infarto do miocárdio e óbito cardíaco.^38^A Tabela 5.1 apresenta as indicações para EED nas diretrizes.^39^

**Tabela 5.1:** Indicações da ecocardiografia de estresse farmacológico com dobutamina na isquemia miocárdica^3,12,35-43^

1) Diagnóstico de DAC com probabilidade pré-teste intermediária.
2) Dor torácica aguda em pacientes de risco intermediário (sem anormalidades no eletrocardiograma e biomarcadores negativos).
3) Sintoma de angina em pacientes com stent ou revascularização prévios.
4) Obstrução coronária moderada à angiotomografia ou angiografia coronária: significado funcional.
5) Estratificação de risco pós-infarto do miocárdio.
6) Etiologia de dispneia.

A EED pode não detectar isquemia de pequenos vasos distais e pacientes com obesidade ou enfisema podem ter janelas acústicas ruins, resultando em imagens subótimas mesmo com o uso do agente de realce ultrassonográfico (ARUS) ou contraste para ultrassom.

A seleção do exame apropriado se baseia no quadro clínico do paciente, incluindo a natureza dos sintomas, o perfil de risco, os pontos fortes e as limitações da modalidade escolhida.^40^

Para os equipamentos e medicamentos necessários para a realização da EED, consulte a Tabela 5.2.^39^

**Tabela 5.2:** Equipamento e medicamentos necessários para a realização da ecocardiografia de estresse farmacológico com dobutamina^3,12,35-43^

1) Ecocardiógrafo digital com protocolo de estresse para avaliação simultânea de imagens no formato *quad screen.*
2) Aparelho para monitorização (preferencialmente contínua) de eletrocardiograma e de pressão arterial.
3) Equipamento e medicamentos para ressuscitação cardiopulmonar com desfibrilador/cardioversor.
4) Oxigênio para suprimento (se necessário).
5) Equipamento para intubação.
6) Bomba de infusão para dobutamina.
7) Drogas para reação alérgica, além de dobutamina, atropina e metoprolol (e de outros fármacos e seus antídotos, caso o protocolo farmacológico seja outro).

### 5.1. Protocolo da Ecocardiografia de Estresse Farmacológico com Dobutamina

O protocolo foi definido em 2007 pela American Society of Echocardiography em suas diretrizes.^12^

Preparo do paciente: Os pacientes devem estar em jejum por 4 a 6 horas antes do exame e podem continuar com seus medicamentos regulares. Porém, devem ser interrompidos pelo menos 24 horas antes do teste: amiodarona, bloqueadores dos canais de cálcio, nitratos, medicamentos que contêm cafeína e nicotina (particularmente para o estresse com dipiridamol) e, especialmente, betabloqueadores (para evitar resultado falso negativo).^37^ Idealmente, os últimos devem ser interrompidos 3 a 4 dias antes do exame caso o objetivo seja o diagnóstico de isquemia miocárdica.

Etapas do procedimento: 1) acesso endovenoso; 2) eletrodos em 12 derivações; 3) colocação do esfigmomanômetro; 4) ecocardiografia dirigida em repouso, para comparação das imagens sob estresse; 5) aquisição de imagens da ecocardiografia de repouso, dose baixa, intermediária (opcional), pico de dobutamina e fase de recuperação, com cineloops para fins de registro nos cortes principais: eixo longo, eixo curto do VE (nível dos músculos papilares), apical de quatro câmaras, apical de duas câmaras e opcional, o apical de três câmaras (valor adicional para avaliação do ápice do VE ou em substituição do paraesternal longitudinal). Alguns autores optam pela aquisição de todos os cortes pela janela apical (cortes apicais de quatro, três e duas câmaras). É importante contemplar todas as paredes do VE irrigadas pelos principais vasos coronarianos; 6) monitorização contínua do eletrocardiograma (ECG), da FC e da PA; 7) Se necessário, é realizado o esforço isométrico (*handgrip*) em associação; infusão de atropina para potencializar o exame; e infusão de metoprolol para antagonizar os efeitos da dobutamina (quando necessário); 8) relatório do exame.

Ação e farmacocinética da dobutamina: A dobutamina apresenta forte efeito agonista adrenérgico que atua nos receptores adrenérgicos B1 para aumentar a FC e a contratilidade e nos receptores B2 para provocar vasodilatação periférica. Sua ação tem início 1 a 2 minutos após a infusão e tem meia-vida de 2 minutos. A dobutamina é o agente mais utilizado devido à sua alta sensibilidade e efeitos colaterais controláveis.^37^ Tem metabolização hepática e nos tecidos periféricos, mas não há necessidade de redução da dose definida para pacientes com disfunção renal ou hepática. Em geral, é administrada em doses crescentes, começando com 5 mcg/kg/min e aumentando em intervalos de 3 minutos até 10, 20, 30 e 40 mcg/kg/min. A dobutamina pode ser iniciada na dose baixa de 2,5 mcg/kg/min; e passar pela dose de 7,5 mcg/kg/min para facilitar o reconhecimento da viabilidade miocárdica em segmentos anormais (acinéticos) no repouso.

A meta é alcançar a FC alvo (85% da FC máxima prevista para a idade), o que torna o teste conclusivo. Contudo, nova alteração da contratilidade ou uma piora das alterações anteriores leva à interrupção do exame por isquemia, mas arritmias importantes, hipertensão arterial acentuada e sintomas intoleráveis também podem levar a interrupção do teste. Quando não é possível alcançar a FC alvo apenas com dobutamina, o esforço isométrico máximo e/ou atropina podem ser adicionados para aumentar a sensibilidade do EED, particularmente nos pacientes em uso de betabloqueadores, bloqueador de canal de cálcio ou nitratos e naqueles com doença uniarterial.^3,41,42^ Deve-se ter cuidado ao classificar o exame como submáximo quando o paciente atinge 85% da frequência cardíaca máxima prevista para a idade. Essa frequência corresponde à FC alvo, sendo, portanto, suficiente para caracterizar um exame conclusivo, e não submáximo ou inconclusivo.

A atropina é administrada em doses de 0,25 a 0,50 mg em intervalos de 0,5 a 1 minuto, conforme necessário para atingir a FC alvo, até um total de 2,0 mg. Para evitar efeitos colaterais, a dose de 0,25 mg é sugerida para pacientes idosos, com pequena superfície corporal, neuropatas ou que estão próximos da FC alvo. A administração de atropina na dose mais precoce de dobutamina (20 ou 30 mcg/kg/min) facilita o alcance precoce com menos efeitos colaterais, menor dose acumulada de dobutamina e menor tempo. A suspensão dos betabloqueadores facilita o alcance da FC alvo, e a não suspensão adequada dos mesmos pode levar à episódios de hipertensão ou arritmia^3,41^ no pico de dobutamina devido ao desbloqueio súbito dos receptores beta-adrenérgicos.

Betabloqueadores podem ser administrados para reverter os efeitos da dobutamina (principal utilização), mas também para aumentar a sensibilidade do exame para a doença univascular quando aplicados no pico de estresse, na vigência da dobutamina e de modo rápido.^42^

Os pacientes podem desenvolver arritmias menores, ou mais significativas, como fibrilação atrial e/ou taquicardia ventricular não sustentada, que geralmente desaparecem após a interrupção da infusão de dobutamina. Nesses casos, devem ser administrados betabloqueadores. Em caso de taquicardia ventricular sustentada, a primeira consideração deve ser uma isquemia miocárdica.^3^

Os medicamentos usados na EED, assim como suas doses e efeitos colaterais, se encontram na Tabela 5.3.

**Tabela 5.3:** Drogas endovenosas usadas durante o teste de ecocardiografia de estresse, efeito colateral e contraindicações^3,12,35-43^

Dobutamina	Dose de 5 a 40 mcg/kg/min com aumento progressivo a cada 3 minutos. Efeitos colaterais: náusea, tremor, palpitação, cefaleia, hipotensão, hipertensão arterial, dor precordial, arritmia. As contraindicações se encontram na Tabela 5.4.
Atropina	Dose de até 2 mg iniciando na dose de 20 ou 30 mcg de dobutamina (protocolo precoce), caso não esteja indicada a interrupção do teste. Efeitos colaterais: taquicardia, palpitação, boca seca, midríase, náusea, agitação, retenção urinária, aumento da pressão intraocular. Contraindicação: glaucoma de ângulo fechado, hipertrofia de próstata com retenção urinária.
Metoprolol	Dose de 1 mg a 10 mg Efeitos colaterais: bradicardia, hipotensão arterial, tontura, cefaleia. Contraindicações: asma brônquica, doença pulmonar grave, síndrome bradi-taqui, bloqueio atrioventricular

As contraindicações absolutas e relativas para a dobutamina estão resumidas na Tabela 5.4.^3^

**Tabela 5.4:** Contraindicações da ecocardiografia de estresse farmacológico com dobutamina na isquemia miocárdica^38,39,43^

**Contraindicações absolutas**
1) Infarto agudo do miocárdio até 3-5 dias.
2) Angina instável.
3) Arritmia não controlada com sintomas e instabilidade.
4) Estenose valvar aórtica grave sintomática.
5) Miocardite, pericardite aguda
6) Hipertensão arterial grave (PA > 200/110 mmHg) em repouso.
7) Endocardite ativa.
8) Tromboembolismo pulmonar ou infarto pulmonar.
9) Cardiomiopatia hipertrófica obstrutiva.
10) Trombo ventricular recente ou móvel.
**Contraindicações relativas**
1) Hipertireoidismo, anemia importante, AVC recente.
2) Aneurisma intracraniano ou de aorta torácica (>40 mm).
3) Estenose valvar aórtica moderada.

AVC: acidente vascular cerebral; PA: pressão arterial.

Todos os pacientes devem assinar o termo de consentimento informado antes do exame.

Em relação à aquisição da imagem, é preciso tomar cuidado para que os cortes sejam feitos no mesmo plano e na mesma profundidade durante todo o exame para comparação mais confiável no formato *quad-screen*.^38^ Os ajustes devem ser realizados nas imagens basais de repouso e mantidos durante todo o exame.

### 5.2. Tipo de Resposta Miocárdica

**Figura 5.1:**
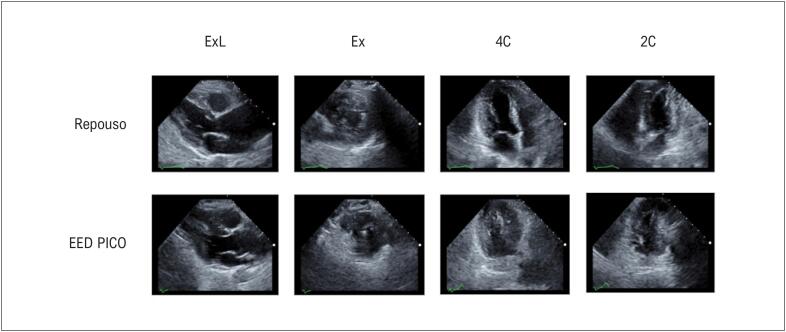
Ecocardiografia bidimensional – Ecocardiografia de estresse farmacológico com dobutamina (EED) – resposta normal do ventrículo esquerdo: redução da cavidade ventricular esquerda e hipercinesia de todas as paredes.

A EED normal é definida pela presença de movimento normal da parede regional e global em repouso, hipercinesia regional e global sob estresse e redução importante no tamanho da cavidade do VE devido à redução da pré e pós-carga (fechamento fisiológico da cavidade ventricular esquerda) (Figura 5.1).

**Figura 5.2:**
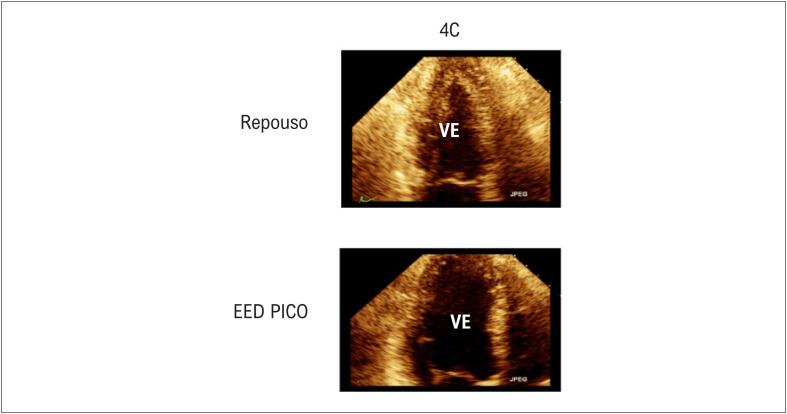
Ecocardiografia bidimensional – Ecocardiografia de estresse farmacológico com dobutamina (EED) – isquemia lateral e septo com dilatação do ventrículo esquerdo (VE).

A resposta isquêmica é definida pelo desenvolvimento de uma nova ou piora da anormalidade no movimento-espessamento da parede sob estresse em ao menos um segmento miocárdico (Figura 5.2).

A isquemia miocárdica pode ser causada por doença dos vasos epicárdicos ou por outro motivo, como doença microvascular, resposta hipertensiva ao estresse ou cardiomiopatia subjacente.

Uma anormalidade grave do movimento-espessamento da parede em repouso, como acinesia, sem alteração com o estresse é considerada uma resposta fixa e representa uma região infartada transmuralmente ou com uma borda de viabilidade epicárdica limitada. É preciso ressaltar que a acinesia de repouso que se transforma em discinesia ao estresse é considerada representativa de uma resposta mecânica de um segmento infartado ou reflexo do aumento da pressão intraventricular e não uma isquemia.

A presença de aneurisma deve ser registrada, dadas as implicações prognósticas e terapêuticas.^3^

As diferenças entre a resposta miocárdica segmentar e a global ao estresse na EED são apresentadas na Tabela 5.5.

**Tabela 5.5:** Tipo de resposta a ecocardiografia de estresse farmacológico com dobutamina em comparação com repouso e dose baixa

**Resposta normal segmentar:** hipercinesia e aumento da força de contração.
**Resposta isquêmica segmentar:** hipocinesia e diminuição da força de contração.
**Resposta normal global:** diminuição do volume diastólico e sistólico finais e aumento acentuado da fração de ejeção.
**Resposta isquêmica global:** diminuição da fração de ejeção e dilatação da cavidade ventricular.

A EED deve ser interrompida quando: (1) atingir a FC alvo; (2) houver detecção de alteração segmentar da contratilidade de pelo menos dois segmentos; (3) ocorrer sintoma limitante ou arritmia complexa; (4) houver hipotensão ou hipertensão arterial importante (PAS ≥ 220-240 mmHg e/ou PA diastólica [PAD] ≥ 120 mmHg); (5) o fim do protocolo for atingido. O uso de ARUS ou contraste para ultrassom deve ser considerado para melhorar a definição do endocárdio na ausência de boa visibilidade em repouso ao corte apical de dois ou mais segmentos contíguos.

Os efeitos colaterais mais observados na EED são sensação de calor, náusea, arrepio, urgência urinária, palpitações relacionadas à taquicardia e, raramente, leve cefaleia.^3^ A cefaleia mais acentuada na vigência de dobutamina deve levantar a suspeita de hipertensão arterial sistêmica.

A interpretação e a conclusão do teste na isquemia se encontram na Tabela 5.6.^3^

**Tabela 5.6:** Interpretação do espessamento segmentar no resultado da ecocardiografia de estresse farmacológico com dobutamina na pesquisa da isquemia miocárdica

1. Repouso = normal Estresse = hipercinesia Conclusão = normal
2. Repouso = normal ou hipocinesia Estresse = hipocinesia ou acinesia Conclusão = isquemia
3. Repouso = acinesia Estresse = melhora em baixa dose e piora em alta dose Conclusão = viabilidade e isquemia
4. Repouso = acinesia Estresse = acinesia ou discinesia Conclusão = cicatriz/fibrose

Os dados absolutos e relativos para a conclusão do teste se encontram na Tabela 5.7.

**Tabela 5.7:** Dados absolutos e relativos para conclusão da ecocardiografia de estresse

1- Atingir 85% da frequência cardíaca (FC) máxima prevista para a idade (220 – idade). 2- Dose máxima de drogas usadas (fim do protocolo) ou sintomas limitantes. 3- Sinais ecocardiográficos de isquemia miocárdica (independente de qualquer outro fator). 4- Taquicardia ventricular sustentada / arritmias complexas. 5- Queda sintomática da PA ou da FC (PAS > 20 mmHg).
Dados relativos para conclusão da ecocardiografia de estresse: 1- Piora da contratilidade em novos segmentos (um ou mais) ou segmentos já alterados em repouso. 2- Dilatação do ventrículo esquerdo. 3- Início de arritmia (fibrilação atrial, taquicardia supraventricular, arritmia ventricular complexa).

FC: frequência cardíaca; PA: pressão arterial; PAS: pressão arterial sistólica.

Diversos estudos demonstram a excelente acurácia da EED usando a arteriografia coronariana como padrão ouro para comparação. Para a detecção de doença arterial (DA), a EED tem sensibilidade semelhante à imagem tomográfica de perfusão nuclear, com maior especificidade. A EED tem maior sensibilidade para detecção de lesão na artéria descendente anterior e de tronco principal esquerdo ou multiarterial.^3^

Se não for detectada isquemia e o estresse for inadequado, seja por janela acústica limitada ou por não se atingir a frequência cardíaca alvo, o resultado do exame pode ser falso negativo.

Pacientes com resultados de ecocardiografia de estresse falso-positivos são semelhantes àqueles com resultados verdadeiro-positivos. Ambos devem receber manejo intensivo dos fatores de risco e acompanhamento cuidadoso.^3^

Deve constar do relatório como conclusão do exame:

Normal, isquemia, ausência de espessamento da parede (fibrose) ou combinação desses achados, desde que obtido o *endpoint* adequado. Caso nada tenha ocorrido ao final do protocolo e a frequência alvo não tenha sido atingida, o teste pode ser descrito como inconclusivo, ou mesmo como negativo até a FC atingida. Nesse último caso, porém, é importante ressaltar que a sensibilidade do mesmo se encontra reduzida para diagnóstico de isquemia miocárdica induzida por não ter atingido a FC alvo.

Para a interpretação das imagens, são comparados o tamanho, a forma e a função do VE em cada etapa nos quatro cortes simultâneos e em cada corte nas etapas simultâneas. Essas imagens devem ser obtidas na mesma posição em cada estágio. Na resposta normal sob estresse, o VE é menor do que em repouso, a forma é mantida e aumenta a excursão do endocárdio, com consequente aumento da espessura miocárdica.^43^

A dilatação e redução da fração de ejeção do VE (FEVE) durante a EED geralmente é indicativa de isquemia extensa.

Cada segmento é pontuado de acordo com os seguintes critérios:

1. Movimento normal da parede (espessamento miocárdico >50%);
2. Hipocinesia (espessamento miocárdico de 10 a 50%);
3. Acinesia ou hipocinesia grave (espessamento miocárdico <10%);
4. Discinesia (movimento sistólico paradoxal e movimento sistólico abaulado para fora).

O escore de motilidade parietal (WMSI, do inglês *wall motion score index*) é calculado pela divisão da soma de todos os escores segmentares pelo número total de segmentos miocárdicos (16 ou 17, com ápice do VE incluído). No miocárdio normal e não isquêmico, o índice é igual a 1.^39^ Os cortes ecocardiográficos que devem ser obtidos e a correspondente irrigação coronariana estão demonstrados na Tabela 5.8.

**Tabela 5.8:** Avaliação ecocardiográfica dos segmentos e irrigação coronária

CORTE	IRRIGAÇÃO CORONARIANA
Paraestenal longitudinal ou Apical 3C	ADA, ACD e ACX
Apical 4C	ADA, ACD e ACX
Apical 2C	ADA, ACD
Paraesternal transversal	ADA, ACD e ACX

ADA: artéria descendente anterior; ACD: artéria coronária direita; ACX: artéria circunflexa.

Uma dificuldade para interpretar o exame é que portadores de marca-passo podem ter resposta de frequência inadequada e também podem apresentar anormalidades regionais da parede devido ao efeito de estimulação. Por esse motivo, a dobutamina muitas vezes não consegue fornecer as respostas adequadas a esses pacientes.

Em relação à estratificação de risco e prognóstico, a EED geralmente é utilizada para o diagnóstico de DAC com isquemia, risco de eventos cardíacos e prognóstico. A estratificação de risco com a EED normal está associada a um prognóstico benigno e é semelhante ou superior a imagem de tomografia computadorizada por emissão de fóton único (SPECT, do inglês *single-photon emission computed tomography*) miocárdica normal (tálio-201, tecnécio-99 ou sestamibi).

Quando comparamos os pacientes submetidos a ecocardiografia de estresse de esforço, aqueles submetidos a EED geralmente são mais velhos, têm mais comorbilidades e neles o movimento da parede e a FE foram identificados na análise multivariada como os melhores preditores de eventos cardíacos.^3^

Os dados que devem constar do relatório da EED se encontram na Tabela 5.9.^38^ O segmento basal inferior e apical devem ser avaliados em múltiplas imagens para evitar um resultado falso positivo para isquemia.

**Tabela 5.9:** Recomendações para relato/laudo dos resultados da ecocardiografia de estresse farmacológico com dobutamina^38,39,43^

Informações clínicas e indicação do estudo.
**Repouso:** Avaliação regional do espessamento de parede.
Motilidade parietal: Número, localização e grau de alterações regionais (ou global).
2- Estimativa da fração de ejeção.
**Estresse:** Avaliação regional do espessamento da parede.
1- Motilidade parietal: Número, localização e grau de alterações regionais (ou global). 2- Estimativa da fração de ejeção.
3- A avaliação da contratilidade segmentar deve ser repetida quantas vezes forem necessárias.
4- Se houver uso de agente de realce ultrassonográfico (contraste), informar o agente e dose administrados.
Dados do protocolo utilizado:
1- Doses do(s) agente(s) para estresse farmacológico.
2- Se a FC alvo foi alcançada. FC e PA em cada estágio.
3- Achados clínicos e de ECG durante o estudo do repouso e do estresse.
4- Achados ecocardiográficos pós-teste.
5- Motivo do término do exame (conclusão do protocolo, isquemia, sintomas ou complicações).

FC: frequência cardíaca; ECG: eletrocardiograma; PA: pressão arterial.

### 5.3. Avaliação Durante a Ecocardiografia de Estresse Farmacológico com Dobutamina e na Recuperação

O desenvolvimento de anormalidades na motilidade parietal durante os estágios iniciais do estresse indica a presença de obstrução coronariana grave com pouca ou nenhuma reserva de perfusão miocárdica. Na dose baixa, na presença de obstrução coronariana moderada, com alguma preservação da reserva de perfusão, o aumento do fluxo sanguíneo acarreta aumento do espessamento e da motilidade parietal. Na dose alta, a combinação de taquicardia e obstrução coronariana resulta em declínio acentuado do fluxo subendocárdico, com diminuição correspondente na função regional.

A persistência de anormalidades na motilidade parietal no período de recuperação pode ser consequência do atordoamento e é um indicador de isquemia mais grave. Imagens de recuperação isquêmica tardia devem ser consideradas em pacientes com anormalidades graves e extensas do espessamento da parede induzidas por estresse. A maioria dos pacientes com resultados anormais na EED e submetidos à angiografia coronária apresenta DAC obstrutiva.^3^

A hipoperfusão induzida por estresse reflete a área do miocárdio em risco e a gravidade reflete a magnitude da anormalidade da motilidade parietal, e ambas estão exponencialmente correlacionadas com um aumento nos eventos adversos cardiovasculares.^3^

### 5.4. Dilatação Isquêmica Transitória do Ventrículo Esquerdo

Pacientes com ecocardiograma de estresse anormal e dilatação isquêmica transitória apresentam maior percentagem de doença multiarterial e taxa mais elevada de eventos adversos.

**Figura 5.3:**
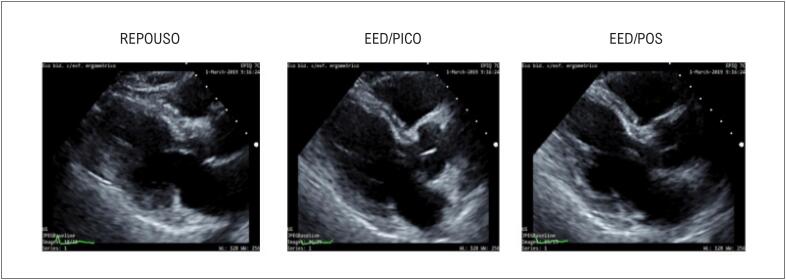
Ecocardiografia bidimensional – bloqueio de ramo esquerdo - eixo longitudinal: repouso – septo normal; EED/pico – septo pouco anômalo; ecocardiografia de estresse farmacológico com dobutamina (EED)/pós-imediato (POS) – septo muito anômalo.

A EED pode ser utilizada para detecção de isquemia e estratificação de risco em pacientes com bloqueio de ramo esquerdo (BRE)^3^ (Figura 5.3).

Em pacientes com BRE submetidos a EED e a coronariografia (Cine), Yanik et al.^44^ observaram boa sensibilidade, especificidade e acurácia da EED para identificação de isquemia no território da artéria descendente anterior (ADA) de 82%, 95% e 90%, nos territórios da artéria circunflexa e coronária direita de 88%, 96% e 93%. Em estudo semelhante com EED, tomografia de perfusão miocárdica e Cine, Mairesse et al.^45^ observaram que a EED teve bom desempenho na detecção de isquemia no território da ADA, com sensibilidade de 83%, especificidade de 92% e acurácia de 87%.

Os pacientes idosos têm a indicação de EED (estresse farmacológico em geral) como primeira opção, assim como pacientes com problemas ortopédicos ou neurológicos que não conseguem realizar o teste de esforço.^46^ Se a FC desejada não for alcançada, indica-se atropina e o esforço isométrico concomitantemente, com metade da força máxima por 3 a 4 minutos,^47^ mantendo a boca aberta e abdome relaxado para evitar a manobra vagal.

### 5.5. Comparação com Outras Modalidades de Imagem

Grandes estudos e meta-análises compararam o valor preditivo da EED e do SPECT em diferentes pacientes. O estudo PROMISE comparou imagens anatômicas usando angiotomografia com imagens funcionais em 10.003 pacientes estáveis com sintomas sugestivos de DAC. Contudo, realizou-se SPECT de estresse ou EED em apenas 1.083 pacientes. Nenhum desses estudos que compararam a EED com outras modalidades considerou o valor incremental dos achados auxiliares detectados por imagens ecocardiográficas realizadas no momento da ecocardiografia de estresse, incluindo detecção de causas não isquêmicas de sintomas cardíacos.^3^

A EED tem precisão diagnóstica e prognóstica semelhante à imagem de perfusão sob estresse com radionuclídeos ou ressonância magnética.

### 5.6. Valor da Ecocardiografia de Estresse Farmacológico com Dobutamina Negativa

Samiei et al.^48^ estudaram 705 pacientes sem história prévia de DAC e ecocardiografia de estresse de esforço ou EED negativas. Em seu estudo, os pacientes que tiveram eventos eram mais velhos, diabéticos ou hipertensos, e os resultados devem ser interpretados com mais cautela, dado o menor valor preditivo negativo da EED nesses pacientes. Além disso, a precisão diagnóstica pode ser significativamente afetada pelo uso de betabloqueadores, resultando em teste falso negativo.^43,49^

### 5.7. Complicações da Ecocardiografia de Estresse Farmacológico com Dobutamina

Complicações ocorrem em < 0,1% dos pacientes (meta-análise de 26 estudos). A mais comum é a arritmia na isquemia importante e na FE < 35%. Fibrilação atrial e arritmias ventriculares não sustentadas ocorrem em cerca de 3% dos pacientes. Contrações atriais ou ventriculares prematuras frequentes ocorrem em cerca de 10%. A taquicardia ventricular sustentada é rara.^43^ Pode ocorrer angina, hipotensão e obstrução da região médioventricular e da VSVE (esta pode levar a dor torácica e hipotensão arterial). Apesar da segurança, aproximadamente 50% dos pacientes relatam alguma reação: náusea, rubor, cefaleia, pescoço ou peito batendo, parestesia, urgência urinária, dispneia ou boca seca.^38^

**Figura 5.4:**
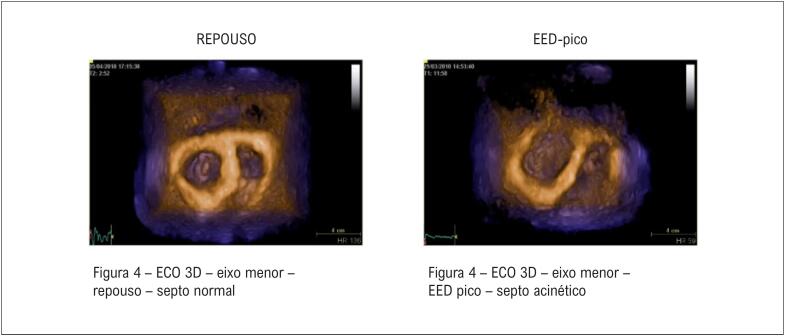
Ecocardiografia tridimensional (ECO 3D) – eixo menor – repouso – septo normal. ECO 3D – eixo menor – ecocardiografia de estresse farmacológico com dobutamina (EED) pico – septo acinético.

A Figura 5.4 mostra a um ecocardiograma com dobutamina, adicionando a técnica tridimensional, positivo para isquemia miocárdica induzida em região septal.

## 6. Ecocardiografia de Estresse com Dipiridamol

O dipiridamol é um estressor miocárdico farmacológico que provoca acúmulo de adenosina endógena por meio do estímulo de seus receptores A2 nas arteríolas coronarianas.^50^ O fármaco determina o aumento do fluxo sanguíneo coronariano e do fluxo sanguíneo miocárdico em até 4 a 5 vezes em relação ao basal, sem aumentar significativamente a FC e o volume sistólico e causando queda da PAS.^3^ Assim, na presença de uma estenose epicárdica crítica ou disfunção microcirculatória, sua administração resulta em heterogeneidade do fluxo sanguíneo coronário entre segmentos comprometidos por estenoses e artérias coronárias normais.^3^ Ocorre uma incompatibilidade entre oferta e demanda, o que leva a uma diminuição do fluxo subendocárdico em áreas de estenose arterial por meio de fenômenos de roubo de fluxo.^51^

As indicações, tipos de equipamentos, preparo do paciente, etapas do procedimento, aquisição de imagens, consentimento informado e tipos de resposta miocárdica são similares àqueles descritos para a dobutamina.

### 6.1. Protocolos

**Figura 6.1:**
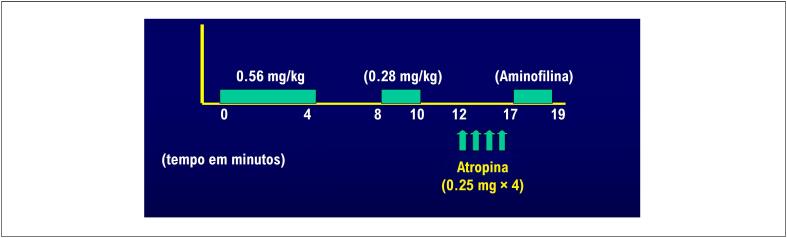
Protocolo dipiridamol-atropina alta dose escalonado.

**Figura 6.2:**
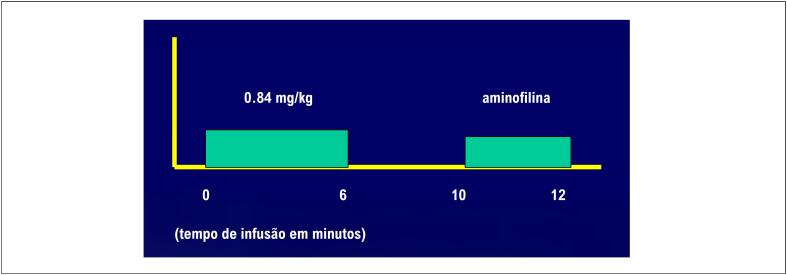
Protocolo dipiridamol alta dose acelerado.

Há dois protocolos padrão para a administração de dipiridamol:

1. **Protocolo escalonado:**
  Infusão intravenosa de 0,84 mg/kg durante 10 min, em duas infusões separadas, sendo 0,56 mg/kg durante 4 min, seguida de 4 min de observação sem medicação e, se ainda negativo o teste, 0,28 mg/kg adicionais em mais 2 minutos. Se nenhum *endpoint* (no caso, isquemia) for alcançado, adiciona-se 0,25 mg de atropina a cada minuto, totalizando o máximo de 1 mg neste tipo de teste (Figura 6.1).
2. **Protocolo acelerado:**
  Infusão intravenosa da dose global de 0,84 mg/kg em 6 minutos, seguida de um período de observação de 4 minutos sem medicação, não sendo necessário a administração de atropina ao final do teste (Figura 6.2).
  Em ambos os protocolos, deve-se disponibilizar aminofilina para uso imediato caso ocorra um evento adverso relacionado ao dipiridamol, que deve ser infundida rotineiramente ao final do teste, independentemente do resultado,^5,50^ devido à meia-vida mais longa do dipiridamol.
  O dipiridamol atua pela inibição da recaptação da adenosina endógena. A meia-vida do dipiridamol alfa (ou seja, o declínio inicial após o pico de concentração) é de aproximadamente 30 a 45 minutos. A meia-vida beta (o declínio terminal na concentração plasmática) é de aproximadamente 10 horas. O dipiridamol é metabolizado no fígado em conjugado de ácido glucurônico e excretado na bile.^52,53^ Seu efeito vasodilatador pode causar uma queda na PA e uma leve elevação na FC.

### 6.2. Efeitos Colaterais

Os principais efeitos colaterais do dipiridamol incluem a incidência de bloqueio atrioventricular (BAV), que pode chegar a BAV total, porém em menor grau que aquele observada com adenosina (2%); dor torácica em cerca de 20% dos pacientes, inespecífica e não necessariamente indicativa da presença de DAC; e efeitos colaterais menores, incluindo cefaleia, tontura, náuseas, hipotensão e rubor.^52^

### 6.3. Contraindicações

As principais contraindicações ao uso de dipiridamol (Tabela 6.1) incluem: pacientes com doença pulmonar broncoespástica com sibilância contínua ou história de doença reativa significativa das vias aéreas, hipertensão não controlada (PAS superior a 200 mmHg ou PAD superior a 110 mmHg), PAS inferior a 90 mmHg, hipersensibilidade conhecida ao dipiridamol, ingestão de cafeína nas últimas 24 horas e BAV de segundo ou terceiro grau sem marca-passo funcional.^52^ Entretanto, a presença de angina instável ou síndrome coronariana aguda complicada, arritmias cardíacas complexas, estenose valvar aórtica grave sintomática e CMH com obstrução da VSVE também impedem a realização de um teste estressor do miocárdio, assim como nos demais protocolos.^3^

**Tabela 6.1:** Contraindicações para a ecocardiografia de estresse com dipiridamol

Contraindicações para todos os protocolos	Contraindicações para ecocardiografia de estresse com dipiridamol
Angina pectoris instável	Doença pulmonar broncoespástica com sibilância contínua
Síndrome coronariana aguda ou complicada	História de doença reativa significativa das vias aéreas
Arritmia cardíaca complexa ICC descompensada	BAV de segundo ou terceiro grau sem marca-passo funcional
Estenose aórtica grave sintomática	Pressão arterial sistólica inferior a 90 mmHg
CMH e obstrução na via de saída do ventrículo esquerdo	Hipersensibilidade conhecida ao dipiridamol
	Ingestão de cafeína nas últimas 24 horas

BAV: bloqueio atrioventricular; ICC: insuficiência cardíaca congestiva.

### 6.4. Reserva de Fluxo Coronariano

O estresse farmacológico com dipiridamol é mais seguro, mais simples e o mais adequado para a avaliação combinada da RFC com movimento-espessamento parietal.^50^ Entretanto, as características individuais dos pacientes, o custo local do medicamento e as preferências do examinador devem ser levadas em consideração na escolha de um teste em detrimento de outro. Por exemplo, em pacientes com hipertensão arterial sistêmica grave e/ou história de arritmias atriais ou ventriculares significativas, o teste com dipiridamol pode ser mais adequado.

### 6.5. Acurácia

A acurácia do estresse farmacológico com dipiridamol (protocolo acelerado ou escalonado com atropina), quando comparado à dobutamina-atropina, tem alta precisão diagnóstica, semelhante à da ecocardiografia de estresse máximo em esteira pós-exercício, mas ao custo de uma sensibilidade menor, e especificidade maior. Além disso, o teste de ecocardiografia de estresse com dipiridamol-atropina pode superar os efeitos da terapia concomitante com betabloqueadores, atingindo níveis elevados e valor diagnóstico comparável.^54^ Contudo, o uso de betabloqueadores, por ter ação cardioprotetora, quando não suspenso antes do exame, pode induzir também aqui, a um resultado falso-negativo.

Diversas meta-análises compararam a precisão das imagens de perfusão com a ecocardiografia de estresse.^3^ Esses testes obtiveram sensibilidades semelhantes para a detecção de DAC, mas a ecocardiografia de estresse apresentou maior especificidade. Para detecção de lesão do tronco principal esquerdo ou DAC multiarterial, a ecocardiografia de estresse apresentou maior sensibilidade em comparação à imagem de perfusão miocárdica nuclear, que compara diferenças relativas na perfusão e pode não detectar a isquemia equilibrada ou global.^3,55-57^

## 7. Ecocardiografia de Estresse com Adenosina ou Regadenoson

A adenosina é uma purina presente em todos os tecidos corporais,^58,59^ originada dos nucleotídeos de adenina e da degradação de ATP. É metabolizada com a mesma facilidade com a qual é gerada, tornando seus níveis corporais muito variáveis.^58,60,61^ Sua ação é mediada por meio de quatro receptores de membrana, os receptores de adenosina A1, A2A, A2B e A3.^58,59,62^ Todos são acoplados à proteína G,^59^ sendo que A1 e A3 se ligam à proteína G inibitória e os receptores A2 se ligam à G estimulatória.^59-61^ Isso significa que recrutam diferentes vias mediante a ligação com a adenosina.^58-73^ Os receptores de adenosina podem então ser divididos em dois subtipos principais: receptores A1 e A3, que inibem a adenilato ciclase, e receptores A2 estimulados pela enzima adenilato ciclase. Os receptores A1 predominam no miocárdio, enquanto os receptores A2 são encontrados nas artérias coronárias (células endoteliais e musculares lisas). Portanto, a ativação dos receptores A2A dilata as artérias coronárias, causando hiperemia e aumento da carga sanguínea coronariana. A ativação dos receptores A2A na presença de estenose coronariana crítica desempenha um papel fundamental na vasodilatação inadequada e isquemia subendocárdica para fenômenos de roubo vertical e horizontal e anormalidade da motilidade segmentar regional. Essas ações também estão presentes no teste com dipiridamol.

A infusão intravenosa ou bolus intracoronário de adenosina induz fluxo máximo (hiperemia). Em condições fisiológicas, a artéria coronária epicárdica apresenta fluxo laminar e gradiente de pressão nulo ou baixo da parte proximal para a distal. A estenose coronariana induz aceleração do fluxo no local e pode diminuir a pressão distal às lesões em repouso, limitando a aceleração do fluxo da hiperemia e provocando uma diminuição adicional da pressão pós-estenótica durante o estresse.^22^

Em condições fisiológicas normais, a perfusão miocárdica é autorregulada por pré-arteríolas no epicárdio e arteríolas no miocárdio, de modo que o tônus microvascular contribui para a maior parte da resistência coronariana. Condições microvasculares patológicas, incluindo fibrose perivascular (hipertensão arterial,^64^ DAC,^65^ hipertrofia miocárdica, diabetes mellitus, transplante cardíaco, etc.), podem provocar angina microvascular, aumentando o tônus microvascular em repouso e/ou limitando a vasodilatação no estresse.

Portanto, o uso de vasodilatadores para induzir estresse parece justificado em testes funcionais não invasivos. Para otimizar o efeito isquêmico e avaliar alterações na contratilidade segmentar, é preciso usar doses maiores do que as necessárias para avaliação da perfusão e, por isso, os protocolos empregados no laboratório de ecocardiografia de estresse devem adotar doses maiores e mais rápidas de vasodilatador em relação à cintilografia de perfusão. O resultado é a elaboração de protocolos distintos.

Os efeitos cardiovasculares da adenosina exógena administrada por via intravenosa em seres humanos são inibição vagal e aumento da FC em doses baixas. Em doses altas, pode ocorrer inibição do nó sinusal e da condução atrioventricular, bradicardia, BAV, estimulação simpática, vasodilatação em todos os leitos arteriolares, exceto arteríolas renais pré-glomerulares, onde promove vasoconstrição e hiperventilação, que é explicada pela interação com quimiorreceptores carotídeos.

### 7.1. Uso Durante a Ecocardiografia de Estresse

**Figura 7.1:**
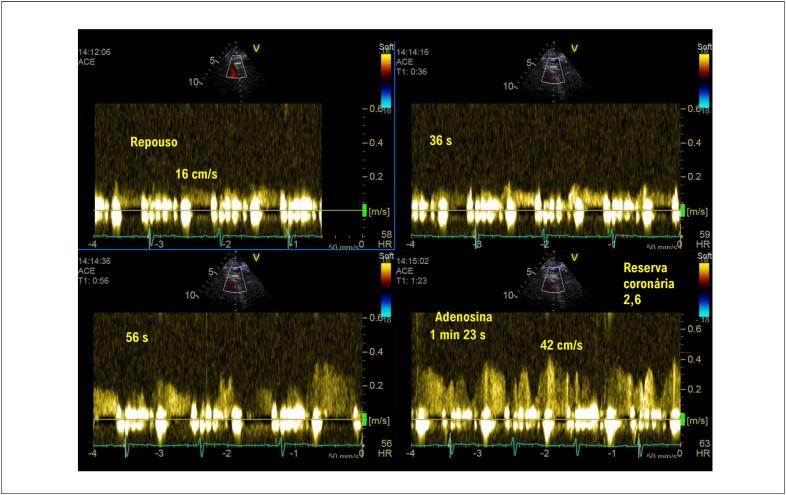
O uso de adenosina durante a ecocardiografia de estresse demonstra a contribuição importante do efeito vasodilatador no aumento da reserva de fluxo coronariano (RFC), com aumento progressivo e importante durante o exame, visto após 1,23 min de infusão da adenosina. A RFC passou de 16 cm/s no repouso para 42 cm/s.

A infusão intravenosa de adenosina promove o aumento discreto da FC e do débito cardíaco e a diminuição discreta da pressão sistêmica (similar ao dipiridamol). A taquicardia leve ocorre apesar dos efeitos cronotrópicos e dromotrópicos negativos diretos da adenosina devido à estimulação dos receptores miocárdicos A1, por estimulação direta dos quimiorreceptores arteriais simpáticos excitatórios,^67^ ou indiretamente, por vasodilatação sistêmica (Figura 7.1).

Em indivíduos normais, o fluxo sanguíneo coronariano aumenta de quatro a cinco vezes o fluxo basal após a infusão de adenosina, um aumento comparável àquele causado por altas doses de dipiridamol e substancialmente maior do que aquele induzido por exercício ou dobutamina, durante o qual o fluxo sanguíneo coronariano aumenta cerca de três vezes em relação ao valor basal.^67^ O efeito dilatador coronariano máximo é atingido até 2 minutos após a administração de adenosina e desaparece rapidamente até 2–5 minutos após a interrupção da infusão.^68^

Teoricamente, o regadenoson tem características atraentes, próximas às do agente "ideal", por seu rápido início de ação, duração adequada do efeito, que permite a aquisição da imagem, e menos efeitos colaterais. A razão é a estimulação seletiva dos receptores A2A especificamente responsáveis pela vasodilatação coronariana, evitando os efeitos indesejáveis da estimulação dos receptores A1, A2B e A3 (como dispneia, cefaleia e rubor). A incidência desses efeitos colaterais não é substancialmente reduzida em comparação com a adenosina, embora sua gravidade seja menor.^69^

#### 7.1.1. Indicações

A principal indicação do teste de estresse com adenosina ou regadenoson é a detecção de DAC epicárdica ou microvascular que cause isquemia miocárdica. Também pode ser utilizado para avaliação de viabilidade miocárdica, assim como o dipiridamol, porém com menor reprodutibilidade em comparação com a dobutamina.

O histórico de segurança e a meia-vida curta tornam a adenosina especialmente indicada em pacientes com estenose aórtica grave,^70,71^ no pós-operatório de crianças assintomáticas submetidas à operação de troca arterial para transposição das grandes artérias^72^ e pacientes idosos,^73^ que podem ser especialmente vulneráveis a complicações durante o estresse com dipiridamol ou dobutamina.

Pacientes com doença pulmonar obstrutiva crônica moderada ou grave que têm indicação para teste de estresse com imagem e querem evitar broncoconstrição e comprometimento respiratório induzido por adenosina podem participar de aplicações emergentes de regadenoson,^3,74,75^ embora nesses pacientes o uso de dobutamina possa ser mais razoável.

### 7.2. Protocolo para Ecocardiografia de Estresse com Adenosina

A dose de 140 μg/kg/min durante 6 minutos é recomendada rotineiramente para isquemia miocárdica. O aumento da dose até 160 e 210 μg/kg/min pode ser aconselhado em caso de teste negativo na etapa de 140 μg/kg/min e é necessário apenas em 16–18% dos casos.^68,76,77^ Quando os efeitos colaterais são intoleráveis, a titulação decrescente da dose também é possível. Pode ser potencializada com atropina ou *handgrip*. A rotina de todo exame sob estresse deve incluir a monitorização da eletrocardiografia, da PA e, nesse caso, da oximetria de pulso.

Aquisições das imagens padronizadas visando todos os territórios coronarianos (cortes apicais quatro câmaras, duas câmaras e três câmaras ou paraesternal longitudinal, transverso do VE, apical quatro e duas câmaras) devem ser realizadas em repouso, em estágio intermediário, com 2 minutos de infusão, antes do final da infusão e na fase de recuperação.

Para a avaliação do fluxo de reserva na coronária descendente anterior faz-se o registro da velocidade diastólica máxima do fluxo médio-distal em repouso e durante a hiperemia máxima, que normalmente acontece dentro de 90 a 120 segundos da infusão, e antes do final da infusão.

A dose de 100 μg/kg/min durante 2 minutos pode ser utilizada para avaliação de viabilidade miocárdica.

### 7.3. Protocolo para Ecocardiografia de Estresse com Regadenoson

Atualmente, o regadenoson é administrado em bolus de 0,4 mg em uma solução de 5 mL (sem ajuste de dose com base no peso) injetada ao longo de 10 segundos, seguida por um flush de soro fisiológico de 5 mL para garantir a administração adequada do medicamento. O tempo ideal para a aquisição de imagens de perfusão é de 2 a 10 minutos após a infusão do fármaco. O regadenoson permite a avaliação combinada de imagens de perfusão miocárdica e a análise da motilidade segmentar e é usado com mais frequência nos Estados Unidos e no Reino Unido. A adição de atropina pode ser utilizada para potencializar o estresse.^78^

### 7.4. Contraindicações

As contraindicações absolutas são hipotensão arterial e obstrução grave das vias aéreas, broncoespasmo ativo e BAV de segundo ou terceiro grau a não ser que protegidos por marca-passo. Deve-se evitar o uso desses agentes em pacientes com sinais ou sintomas de angina instável ou instabilidade cardiovascular.^75^

Drogas antianginosas e produtos que contêm xantinas diminuem a sensibilidade do teste de estresse com adenosina e, portanto, devem ser removidas 24 horas antes do estresse, incluindo alimentos como café, chás, chimarrão, chocolate, refrigerantes e até banana.

### 7.5. Efeitos Colaterais

Os efeitos colaterais são comuns e podem se tornar um fator limitante em número significativo de pacientes – até 20%.^79^ Os efeitos colaterais limitantes mais frequentes para os estressores vasodilatadores mediados por adenosina incluem falta de ar, rubor, cefaleia, hipotensão arterial, dor torácica intolerável (nem sempre relacionada à isquemia subjacente, possivelmente induzida pela estimulação direta dos receptores de adenosina A1 miocárdica) e BAV de alto grau. O BAV de grau I-II ocorre em 2% dos exames, a hipotensão arterial abaixo de 90 mmHg em 3% e o broncoespasmo grave em 0,1%.^80,81^ Efeitos colaterais menores, como sensação de calor, rubor, dor de cabeça, náuseas e outros sintomas aparecem em 80-84% dos casos.^81^ A redução hemodinamicamente significativa da PAS (> 20 mmHg) é mais provável em pacientes com estenose aórtica grave.^82^ A adenosina exógena tem efeito cronotrópico e dromotrópico negativo ainda mais pronunciado que a adenosina endógena,^83^ tornando a ocorrência de bloqueios atrioventriculares avançados mais frequente com a adenosina do que com o dipiridamol e o trifosfato de adenosina em doses equivalentes. A maioria das reações com adenosina se resolve em até 1 a 5 minutos. Em ocasiões muito raras, é necessária uma infusão de aminofilina.

### 7.6. Acurácia Diagnóstica

Com base em meta-análise publicada de 11 estudos, a ecocardiografia de estresse com adenosina, baseada em anormalidades da motilidade segmentar, apresentou a mesma sensibilidade (79%), especificidade (91,5%) e acurácia que a ecocardiografia de estresse físico, a ecocardiografia com dipiridamol e a ecocardiografia com dobutamina, com especificidade superior em comparação à imagem de estresse do SPECT.^84^

## 8. Outras Modalidades de Ecocardiografia de Estresse: Marca-Passo, Ergonovina, *Handgrip* e Hiperventilação

### 8.1. Ecocardiografia de estresse por Marca-Passo

Em pacientes com contraindicação para estresse físico ou farmacológico, o estresse por estimulação atrial transesofágica pode ser uma alternativa viável. A estimulação é realizada por um marca-passo externo conectado a um eletrodo esofágico bipolar e a um cateter de registro protegidos por bainha de 10F, introduzido por via nasal ou oral sob anestesia tópica. Utiliza-se a menor corrente capaz de fornecer captura estável, iniciando em 10 bpm acima da FC de repouso, com aumento de 20 bpm a cada 2 minutos, até 85% da FC máxima predita para a idade.^85^ Os critérios de interrupção são sintomas intoleráveis, arritmia significativa, ECG com infradesnível do ST ≥ 2 mm após 80 ms do ponto J, PAS > 220 mmHg, PAD > 110 mmHg e PAS < 90 mmHg. Eventos adversos graves são raros. Pode ocorrer BAV de segundo grau tipo Wenckebach. Em comparação com o estresse por dobutamina, apresenta menos hipotensão ou hipertensão arterial, arritmias ou sintomas limitantes, e tem tempo de duração e recuperação mais curtos e FC alvo atingida mais frequentemente. Há excelente concordância entre os dois métodos para a avaliação da motilidade segmentar parietal.^85^ Em pacientes portadores de MCPD, o estresse pode ser provocado por programação não invasiva.^86^ O alvo é 85% da FC máxima ou sinais de isquemia. Apresenta sensibilidade de 70%, especificidade de 90% e acurácia de 78% para o diagnóstico de DAC obstrutiva, sendo preditor independente de morte por qualquer causa.

### 8.2. Associação do *Handgrip* à Ecocardiografia de Estresse

O exercício isométrico por preensão das mãos (*handgrip*) produz aumento discreto da FC e do consumo de oxigênio em comparação com o exercício isotônico. Entretanto, produz aumento súbito e mais significativo da PA. Com o uso de um dinamômetro de mão, é possível estabelecer um protocolo submáximo (25-50% da preensão máxima) por 3-4 minutos.^87^ Possui baixa sensibilidade isoladamente para a pesquisa de DAC obstrutiva, mas é útil quando associado a outras formas de estresse farmacológico.^88^ Quando associado ao *strain*, apresenta sensibilidade de 80%, especificidade de 66% e AUC de 0,77 para diagnóstico de isquemia miocárdica.^89^ Também é utilizado como teste de estresse diastólico alternativo ao cicloergômetro para o diagnóstico de ICFEp. Possui vantagem em relação ao cicloergômetro quanto aos artefatos de movimento. O teste com 40% da contração máxima por 3-5 minutos produz consumo de O_2_ similar à baixa carga com cicloergômetro (20 W), com aumento comparável da relação E/e´.^90^

### 8.3. Ecocardiografia de Estresse com Ergonovina e Hiperventilação

A ergonovina é utilizada como teste de estresse por vasoconstrição. O protocolo inclui bolus endovenoso (0,025 a 0,05 mg) a cada 5 minutos até a ocorrência de sinais de isquemia ou dose total de 0,35 mg. Sua aplicação principal é o diagnóstico de angina vasoespástica, com sensibilidade de cerca de 91% e especificidade de cerca de 88%. Em um estudo com 3.094 pacientes, o estresse por ergonovina foi positivo em 8,6%. Não houve infarto do miocárdio ou morte documentada. Entretanto, 0,6% dos pacientes tiveram sintomas transitórios e um paciente apresentou morte súbita ressuscitada. O exame demonstrou valor prognóstico para eventos cardíacos adversos maiores.^91^ Outra forma de estresse utilizada para o diagnóstico de angina vasoespástica é a hiperventilação associada ou não ao estresse por imersão em água fria. Consiste em um período de hiperventilação com respiração profunda a 30 ipm por 6 minutos, seguido de imersão da mão em água gelada por 2 minutos. Utiliza-se monitorização com ECG e ecocardiografia durante o estresse e após 15 minutos do término. O critério de suspensão é a presença de dor torácica e elevação do ST > 1 mm em ao menos duas derivações. O surgimento de alteração segmentar nova durante o estresse é critério para diagnóstico de vasoespasmo. Em comparação com a injeção de acetilcolina intracoronária durante angiografia, o método apresenta sensibilidade de cerca de 91%, especificidade de 90% e acurácia de 91%.^92,93^

## 9. Ecocardiografia de Estresse Diastólico

A ICFEp é um importante problema de saúde pública em todo o mundo e se agrava à medida que a população envelhece e adquire mais comorbidades. A manifestação clínica mais comum é a dispneia ao esforço.^94,95^ O diagnóstico é complexo nos pacientes que apresentam sintomas apenas no exercício, pois os peptídeos natriuréticos, o débito cardíaco (DC) e a pressão de enchimento do VE (PEVE) podem estar normais ou levemente alterados em relação aos indivíduos com função diastólica (FD) normal. Nesses casos, o aumento da PEVE fica evidente apenas no exercício, ressaltando a importância de teste de esforço.^94,95^ A ecocardiografia de estresse diastólico (EEDiast) é uma opção capaz de fornecer informações nesses casos difíceis, utilizando parâmetros da avaliação da FD do VE em repouso e durante exercício.^96-98^

### 9.1. Resposta Normal e Anormal ao Exercício na Função Diastólica

A FD normal permite o enchimento adequado do VE em repouso e durante exercício, com aumento do DC, sem elevação da PEVE. Os parâmetros mais estudados são a relação da velocidade máxima do enchimento inicial mitral (E) pela velocidade diastólica precoce (e’) no Doppler tecidual do anel mitral em repouso e durante exercício. No relaxamento normal, durante o exercício, ocorre aumento proporcional dessas velocidades, mantendo a relação E/e’ inalterada.^96,97^ Nos pacientes com doença miocárdica e relaxamento anormal, não há o aumento de e’ equivalente ao de E, sendo esperado aumento da relação E/e’. Valores de E/e’ durante o exercício < 10 sugerem PEVE normal, enquanto um valor de > 14 (e’ média) ou >15 (usando somente a e’ septal) sugere aumento da PEVE. A estimativa da pressão sistólica da artéria pulmonar (PSAP) pela velocidade máxima do refluxo tricúspide (VMRT) em repouso e durante exercício também é útil, já que a PSAP se eleva com o aumento da PEVE.^94,97^

### 9.2. Indicações para Ecocardiografia de Estresse

**Figura 9.1:**
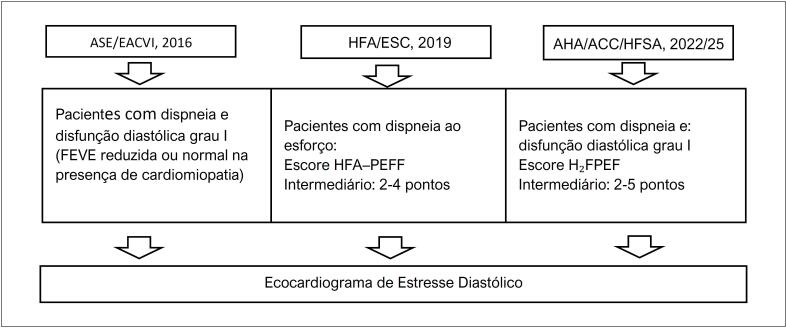
Indicações de ecocardiografia de estresse diastólico segundo as diretrizes.^94,95,98,100^

Para os pacientes com ICFEp com dispneia ao exercício, a avaliação em repouso pode ser insuficiente, justificando a avaliação com exercício para determinar aumento da PEVE.^94,97^ Diretrizes de diferentes sociedades sugerem a EEDiast na suspeita clínica de ICFEp.^3,94,98,99^ A Figura 9.1 exemplifica as recomendações.

O escore HFA-PEFF é o sugerido pela Sociedade Europeia para diagnóstico de ICFEP, enquanto o escore H_2_FPEF é o citado na diretriz americana para diagnóstico de ICFEp.

### 9.3. Protocolos Utilizados na Ecocardiografia de Estresse Diastólico

A EEDiast pode ser realizada com bicicleta ou esteira ergométrica ou na ciclomaca. A bicicleta na posição supina (ciclomaca) é a mais sugerida pelas avaliações da contratilidade segmentar e da FD durante os estágios e no pico.^3,94,99^ A esteira pode ser utilizada e a avaliação da contratilidade segmentar realizada após cessar o exercício (60-90 segundos após), sendo a FD avaliada a seguir. Em ambas as modalidades, os pacientes são estimulados a atingirem a FC máxima prevista para a idade ou o surgimento de sintomas limitantes. As Tabelas 9.1 e 9.2 mostram as vantagens e limitações dos protocolos e variáveis obtidas^3,98^ e os parâmetros adquiridos na ecocardiografia de estresse diastólico.

**Tabela 9.1:** Protocolos, vantagens e limitações

Modalidade	Protocolos	Vantagens	Limitações
Bicicleta	Padrão: carga inicial 25 W, 60 rpm, incrementos de 25 W cada 2 ou 3 minutos.	- Fisiológico. - Mais tempo para aquisição. - Mais estudos com validação.	- Pouca disponibilidade. - Dificuldade para obtenção das imagens. - Menor carga de trabalho alcançado em relação à esteira.
Esteira	Bruce: inclinação inicial de 10% e incrementos de 2% a cada estágio de 3 minutos. Bruce modificado: inclinação inicial de 0%, duração de 3 minutos a cada estágio e incrementos na inclinação.	- Fisiológico. - Disponibilidade. - Alcança altas cargas de trabalho. - Maior duplo produto à custa de FC mais elevada. - Mais específico para detectar isquemia do que a bicicleta.	- Dificuldade em adquirir imagens. - Possibilidade de normalização das alterações no pós-exercício. - Aguardar FC na recuperação < 100-110/min (evitar a fusão das ondas do Doppler). - Sem validação hemodinâmica.

W: Watts; rpm: rotações por minuto; FC: frequência cardíaca.

**Tabela 9.2:** Parâmetros adquiridos na ecocardiografia de estresse diastólico no repouso e durante (bicicleta) ou pós-exercício imediato (esteira)

Parâmetro	Aquisição	Vantagens	Limitações
E	- Apical 4 câmaras. - Medir no pico da velocidade inicial diastólica.	Factível e reprodutível.	- Fusão das velocidades E e A com FC elevada. - Diminui com a idade. - Adquirir quando a FC está entre 100 e 110 bpm.
e’ (lateral, medial)	- Apical 4 câmaras. - Doppler tecidual do anel mitral na região septal e lateral.	Factível e reprodutível.	- Fusão das velocidades e’ e a’ com FC elevada. - Diminui com a idade. - Acurácia limitada em pacientes com calcificação do anel mitral, anel protético, doença pericárdica.
Relação E/e’	- Razão entre as velocidades E, e’.	Validação hemodinâmica.	- Acurácia diminuída em indivíduos normais, calcificação do anel mitral, doença mitral e pericárdica significativas, alteração da contração segmentar.
VMRT	- Apical 4 câmaras ou paraesternal via de entrada do VD. - Doppler contínuo.	Adjunto indireto da estimativa da pressão de enchimento do VE.	- Pode acontecer aumento da velocidade por doença ou resposta normal ao aumento de fluxo no exercício. - Sinal completo não é obtido em 1/3 dos pacientes. - Menos confiável se houver regurgitação tricúspide acentuada.

VE: ventrículo esquerdo; VD: ventrículo direito; E: velocidade máxima do enchimento inicial mitral; A: velocidade tardia do enchimento do VE pela sístole atrial; e’: velocidade diastólica precoce no Doppler tecidual do anel mitral; FC: frequência cardíaca; bpm: batimentos por minuto; VMRT: velocidade máxima do refluxo tricúspide.

### 9.4. Interpretação da Ecocardiografia de Estresse Diastólico

A capacidade funcional deve ser comparada com o esperado para a idade e o sexo do paciente.^3,94,99^ A Tabela 9.3 mostra as respostas normal e alterada de pacientes submetidos a EEDiast.

**Tabela 9.3:** Achados ecocardiográficos possíveis encontrados na ecocardiografia de estresse diastólico

	Ecocardiografia de estresse diastólico com função diastólica normal		Ecocardiografia de estresse diastólico com disfunção diastólica	
	Pré-exercício	Durante ou imediatamente após exercício	Pré-exercício**	Durante ou imediatamente após exercício
E/e’ septal (média)	< 10	< 10	< 14	> 15 (>14)
VMRT* (m/s)	< 2,8	< 2,8	< 2,8	> 2,8 / >3,4

* Os valores de VMRT são diferentes de acordo com as diretrizes. Segundo a ASE/EACVI, é considerado alterado quando VMRT > 2,8 m/s (maior sensibilidade); de acordo com a HFA/ESC, é alterado quando VMRT > 3,4 m/s (maior especificidade).^94,99^

** Na EEDiast alterada, as velocidades de e’ septal em repouso devem ser < 7 cm/s, ou, se somente a e’ lateral for avaliada, devem ser < 10 cm/s.

A EEDiast é importante para detectar alterações que não se apresentam em repouso e que se apresentam apenas sob exercício físico, e as diretrizes atuais recomendam seu uso para identificar pacientes portadores de ICFEp.

## 10. Ecocardiografia de Estresse no MINOCA (Ausência de Coronariopatia Obstrutiva) e na Doença Microvascular

Define-se MINOCA (do inglês, *myocardial infarction with non-obstructive coronary arteries*) os casos de síndrome coronariana aguda (SCA) com aumento da troponina acima do 99º percentil e sintomas ou alterações eletrocardiográficas na ausência de doença coronária epicárdica com obstruções maiores que 50%.^101^ O MINOCA corresponde a aproximadamente 5-10% das SCAs.^101,102^ O prognóstico dos pacientes com MINOCA é variável e depende do mecanismo subjacente.^101^ Essa condição é mais prevalente em indivíduos mais jovens, particularmente mulheres, e os fatores de risco convencionais para doença coronariana, como dislipidemia, tabagismo e histórico familiar, têm uma prevalência reduzida.^103,104^ A identificação de MINOCA, excluindo os diagnósticos diferenciais possíveis, é de extrema importância para o manejo clínico apropriado e precoce.^104^

### 10.1. Fisiopatologia

Atualmente, o MINOCA pode ser classificado em 2 tipos:^103,104^

- Tipo I: Eventos relacionados a placas: rotura, erosão e dissecção da parede da coronária;
- Tipo II: Desequilíbrio entre oferta e consumo de O_2_, os quais ocorrerem nas seguintes situações: espasmo coronariano, embolia coronariana, disfunção endotelial, anemia, hipertensão, hipotensão, extremos de FC e hipoxemia.

É importante ressaltar que situações como a síndrome de Takotsubo não são consideradas MINOCA, pois não apresentam natureza isquêmica. No entanto, há relatos de doença coronariana associada em 10-29% dos pacientes com síndrome de Takotsubo.^104^ Outros diagnósticos diferenciais devem ser considerados e investigados extensivamente: miocardites, alterações relacionadas a sepse, tromboembolismo pulmonar e contusão cardíaca. Entre as causas isquêmicas, o estudo hemodinâmico invasivo para detectar a presença de obstrução coronariana significativa ou a oclusão de pequenos ramos secundários se faz necessário.^101-104^

### 10.2. Principais Métodos Diagnósticos no MINOCA

O diagnóstico inicia pelo quadro típico de dor precordial e aumento da troponina acima do 99º percentil, com comportamento de curva, associado ou não a alterações eletrocardiográficas.^104^ Diante do quadro de SCA, o paciente é submetido a angiocoronariografia, e a obstrução ≥ 50% é excluída pela revisão cuidadosa das imagens. O diagnóstico anatomofuncional pode ser complementado com o uso de ultrassom intracoronário e tomografia de coerência óptica, métodos que permitem analisar as características da placa e identificar a presença de rotura. A medida do fluxo fracionado de reserva miocárdica pode identificar placas que visualmente têm obstrução menor que 50%, mas com repercussão significativa.^103-105^

Atualmente, a RNM cardíaca desponta como o exame de escolha para avaliação de pacientes com suspeita de MINOCA. Esse método proporciona um diagnóstico diferencial preciso de situações clínicas que mimetizam MINOCA. Liang et al.^102^ reportam, em estudo retrospectivo com mais de 800 pacientes em que a RNM reclassifica os pacientes com MINOCA, que somente 27% dos casos considerados eram realmente MINOCA, enquanto 73% dos casos representavam outras patologias, como miocardites.

**Figura 10.1:**
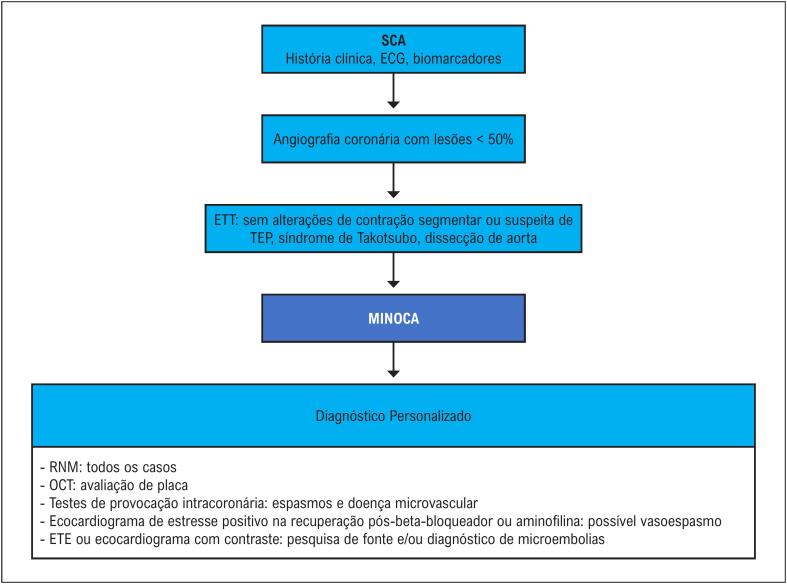
Algoritmo para o diagnóstico e/ou estratificação de MINOCA. SCA: Síndrome coronariana aguda; ETT: ecocardiografia transtorácica; TEP: tromboembolismo pulmonar; S.: Síndrome; RNM: ressonância magnética; OCT: Tomografia de coerência ótica; ETE: ecocardiografia transesofágica. Adaptado de Del Buono et al.^107^

A Figura 10.1 representa a sequência de exames que pode ser seguida na suspeita de MINOCA.

A ecocardiografia de estresse farmacológico tem seu papel no MINOCA ao avaliar ausência de alterações segmentares ou de forma mais assertiva nos casos de vasoespasmo, os quais podem ser diagnosticados na fase de recuperação dos testes farmacológicos com infusão mais rápida de betabloqueadores ou aminofilina.^2,3,106^

### 10.3. Ecocardiografia de Estresse no MINOCA

Na última Diretriz Brasileira de Angina e Infarto da Sociedade Brasileira de Cardiologia,^103^ observa-se indicação IIa para ecocardiografia de estresse no MINOCA de origem microvascular, com o objetivo de estratificação de risco, pois a presença de alterações de contração segmentar implica pior prognóstico.

**Figura 10.2:**
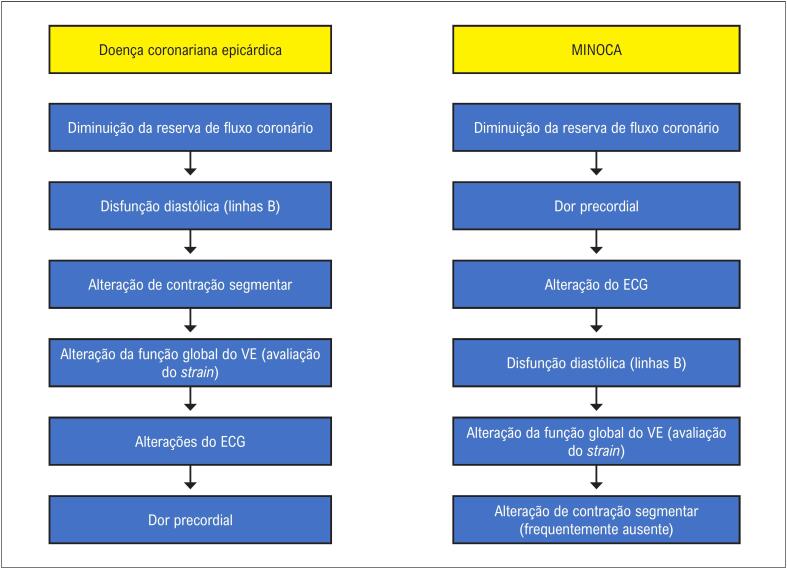
Comparação da cascata isquêmica na doença epicárdica (clássica) e no MINOCA (alternativa). Adaptado de Picano et al.^2^

A cascata isquêmica no MINOCA apresenta um comportamento diferente daquela observada na doença epicárdica, chamada de cascata isquêmica alternativa e clássica, respectivamente (Figura 10.2). Na cascata alternativa, a alteração primária ocorre na redução do fluxo de reserva coronariano. Dessa forma, o protocolo padrão de ecocardiografia de estresse somente com avaliação da contração segmentar tem baixa sensibilidade. Logo, o protocolo ABCDE^106^ é indicado para aumentar a acurácia do exame e estratificar o risco. Nesse protocolo, avalia-se os volumes ventriculares e contração segmentar (A), o ultrassom de pulmão para pesquisa de congestão nos quatro segmentos torácicos anteriores (B), a reserva contrátil do VE pelo cálculo da FE pelo método volumétrico (C), a RFC na ADA por Doppler (D) e o ECG (E) para análise da reserva cronotrópica.^2^ Sendo assim, recomendamos o uso do protocolo ABCDE, descrito em outra seção desta diretriz.^106^ Muitos pacientes referem dor torácica ao exame, a qual não ocorre ao final da cascata isquêmica clássica, mas sim no início.

A utilização de agentes de realce ultrassonográfico pode ser aplicada para avaliação da opacificação do VE e análise de bordos endocárdicos, como utilizado na doença coronariana epicárdica.^2,3^ Seu uso para a análise de reserva coronariana no protocolo ABCDE e pesquisa de perfusão miocárdica (*off label*), em centros com conhecimento especializado, pode ser útil.^2,3^

Vale ressaltar que o MINOCA é um diagnóstico de exclusão na SCA, no qual a ecocardiografia de estresse tem papel diagnóstico principalmente na doença microvascular e no espasmo coronariano, aliado ao protocolo ABCDE para aumentar a sensibilidade do exame.

## 11. Ecocardiografia de Estresse na Doença Valvar Mitral

A ecocardiografia de estresse tem papel importante em auxiliar no refinamento do diagnóstico de gravidade, na estratificação prognóstica e na definição terapêutica para pacientes com doença valvar mitral (DVM), sendo realizada preferencialmente por estresse físico.^108,109^ Condições fisiológicas e doenças associadas podem modificar a apresentação clínica e interferir na acurácia dos métodos diagnósticos convencionais na avaliação da gravidade das patologias mitrais, que sofre influência de condições hemodinâmicas, como pré-carga, pós-carga e FC. Em alguns casos podemos ter critérios de gravidade discordantes ao ecocardiograma, ou mesmo a dificuldade em avaliar os sintomas em determinados pacientes (aspectos socioculturais, alterações cognitivas, sedentários), podendo ocorrer a chamada "dissociação clínico-ecocardiográfica". É importante considerar a complementação com outros métodos de imagem e a avaliação funcional com a ecocardiografia de estresse para uma melhor definição terapêutica.

Há três indicações básicas de ecocardiografia de estresse para avaliação de doenças valvares mitrais nativas: 1) DVM grave assintomática, 2) DVM não grave sintomática, 3) DVM com baixo fluxo transvalvar. O uso do exercício físico é o mais indicado para o estudo da DVM, pois possibilita a avaliação de tolerância física e dos sintomas e a obtenção de dados hemodinâmicos de forma fisiológica (reprodutíveis com atividades da vida diária), embora a dobutamina possa também ser utilizada em casos de estenose mitral (EM) em pacientes com limitação ao exercício.^110^ Para a ecocardiografia de estresse, pode ser utilizado a esteira ergométrica, a bicicleta ergométrica ou ciclomaca; a última tem a vantagem de permitir a análise ecocardiográfica contínua, o que possibilita evidenciar alterações em baixa carga (antes do pico do esforço) e obter dados e imagens do pico do exercício, que podem ser subestimados com o uso de esteira, pois a captura é realizada após o pico de esforço.

### 11.1. Estenose Mitral

A EM reumática ainda é um importante problema de saúde pública no Brasil e em países em desenvolvimento, com alta incidência na África e algumas regiões da Ásia e América Latina,^111,112^ sendo a causa mais prevalente de EM no mundo, seguida pelas doenças degenerativas. A incidência de estenose degenerativa mitral relacionada a calcificação anular mitral aumenta com a idade e é a causa predominante nos países industrializados.^113^

A ecocardiografia de estresse tem indicação validada para a avaliação de gravidade da EM e sua repercussão hemodinâmica, especialmente em pacientes que apresentam discrepância entre o grau de estenose determinada pelos métodos de imagem e clínicos.^108,114,115^ Pacientes com EMs quantificadas como moderadas à ecocardiografia de repouso muitas vezes são bastante sintomáticos ao esforço, e a ecocardiografia de estresse pode, em alguns casos, demonstrar as alterações hemodinâmicas que justificam esses sintomas, com aumento significativo de gradientes transvalvares e PSAP (Tabela 11.1). São estabelecidos como critérios de mau prognóstico o aumento do gradiente médio em valor absoluto acima de 15 mmHg com o esforço^108,110^ ou o aumento da PSAP acima de 60 mmHg.^116^ Outras situações em que a ecocardiografia de estresse pode ser útil na EM incluem pacientes que serão submetidos a grandes cirurgias ou que pretendem engravidar, para predizer o comportamento da doença em situações hemodinâmicas adversas.^117^ Em casos de pacientes com limitação ao exercício, pode ser utilizada ecocardiografia de estresse farmacológico com dobutamina, que está validada para essa situação, e nesse caso o valor absoluto do gradiente médio que denota pior prognóstico é de 18mmHg.^118^

**Tabela 11.1:** Uso da ecocardiografia de estresse na avaliação das patologias valvares mitrais^116,108,110,117^

Patologia mitral	Tipos de estresse	Critérios estabelecidos em diretrizes	Critérios estabelecidos em estudos clínicos	Parâmetros que podem ser avaliados
Insuficiência mitral primária	Exercício físico	-	Aumento de PSAP induzida pelo exercício > 60 mmHg Reserva contrátil Aumento de EROA induzida pelo exercício > 10 mm	Sintomas Resposta pressórica PSAP induzida pelo exercício > 60 mmHg Novas alterações da contratilidade segmentar Aumento da FEVE induzida pelo esforço < ou > 5% Aumento do GLS < ou > 2% induzido pelo esforço
Estenose mitral	Exercício físico (preferível)	Aumento de gradiente médio > 15 mmHg no pico do esforço (ou > 18 mmHg com dobutamina)	Aumento de PSAP > 60 mmHg	Sintomas Resposta pressórica PSAP induzida pelo exercício > 60 mmHg Novas alterações da contratilidade segmentar Queda da FEVE induzida pelo esforço Aumento de gradiente médio > 15 mmHg no pico do esforço

Adaptado de Lancellotti et al.^116^

Deve ser observado o risco de edema agudo de pulmão e complicações hemodinâmicas quando esses pacientes são submetidos a ecocardiografia de estresse.^118^

### 11.2. Regurgitação Mitral

A principal causa de regurgitação mitral (RM) em países desenvolvidos é a doença degenerativa mitral,^119^ enquanto a febre reumática ainda é um fator etiológico importante e que impacta a morbidade e mortalidade nos países em desenvolvimento.^120^ Diretrizes recentes estabelecem critérios de gravidade para a RM,^133^ com um papel importante para a ecocardiografia na definição do momento ideal para intervenção, sobretudo em pacientes sintomáticos, ou em assintomáticos com remodelamento ventricular significativo, disfunção do VE (FEVE ≤ 60%), fibrilação atrial (FA) e/ou hipertensão pulmonar.^114,122^

A ecocardiografia de estresse tem papel fundamental na avaliação dos pacientes com RM, sobretudo em pacientes com discordância entre a clínica e os dados ecocardiográficos, como no paciente sintomático com RM não grave.^123^ O exame realizado sob esforço tem o benefício adicional de avaliar a capacidade funcional do paciente, esclarecendo a correlação entre sintomas e hemodinâmica assim como a estratificação de risco em pacientes que se referem assintomáticos com RM grave. Pacientes sintomáticos com RM aparentemente não grave à ecocardiografia basal podem ter mecanismos dinâmicos que se tornam mais claros durante a ecocardiografia de estresse^124^; por esse motivo, a ecocardiografia de estresse pode ser indicada em pacientes sem outra causa aparente de dispneia ao esforço portadores de doenças valvares mitrais, para excluir a presença de RM dinâmica.

A ecocardiografia de estresse fornece importantes dados objetivos e pode ser realizada em conjunto com o teste cardiopulmonar, adicionando outras variáveis de avaliação funcional, como o consumo de O_2_.

Em pacientes com RM primária, os principais dados que podem ser analisados durante a ecocardiografia de estresse são a piora da RM sob esforço (aumento do ERO > 10 mm), disfunção do VD e aumento da PSAP > 60 mmHg^125,126^ (Tabela 11.1), todos preditores de pior prognóstico. A presença de sintomas ou queda da PAS também são indicadores de pior prognóstico.

Em estudo de Kusunose et al., a presença de TAPSE < 1,9 cm ou PSAP > 57 mmHg sob esforço foram preditores de evolução para cirurgia valvar mitral em pacientes assintomáticos com RM grave.^127^ Além disso, a ausência de reserva miocárdica também foi demonstrada como fator de pior prognóstico em pacientes com RM submetidos a ecocardiografia de estresse, como o aumento de FE ≤5% ou aumento absoluto de valores de *strain* longitudinal global do VE ≤ 2%.^128,129^

A ecocardiografia de estresse pode ser utilizada para avaliação de pacientes com RM secundária, sobretudo quando suspeitamos de componente isquêmico como mecanismo funcional. Em pacientes com miocardiopatia dilatada não isquêmica, é comum a melhora do grau da RM resultante de mobilização de reserva contrátil, que é sinal de bom prognóstico, enquanto nos pacientes com cardiomiopatia isquêmica pode haver piora do grau de RM, o que indica um pior prognóstico.^130,131^ Essa piora também sinaliza a necessidade de abordagem da valva mitral durante a cirurgia de revascularização miocárdica.

O protocolo de exame sugerido para a ecocardiografia de esforço na DVM é apresentado a seguir.

### 11.3. Protocolo de Exame – Ecocardiografia de Esforço para Avaliação de Lesão Valvar Mitral

A ecocardiografia de estresse com exercício físico pode ser realizada em cicloergômetro semissupino (ciclomaca), bicicleta ou esteira ergométrica. O exercício é interrompido quando surgem sintomas limitantes, quando a FC alvo prevista é ultrapassada ou na presença de cansaço ou complicações (arritmias, dor precordial típica, tontura, queda pressórica). A monitorização eletrocardiográfica e de PA não invasiva durante o procedimento é obrigatória.

No exame com cicloergômetro, é possível começar com carga de 25 W, com incremento de 25 W a cada 2-3 minutos. Essa técnica permite acompanhar a ecocardiografia de forma contínua, avaliando a função de VD, os parâmetros de avaliação da lesão valvar (gradientes médios, grau da regurgitação valvar mitral), a função do VE e a PSAP. A carga de exercício máxima obtida é menor em relação àquela obtida em esteira ergométrica, mas suficiente para a avaliação das doenças valvares em esforço.

No caso da esteira ergométrica, comumente são utilizados os protocolos de Bruce ou Bruce modificado, ou protocolo de rampa, sob supervisão de profissional habilitado em teste de exercício, interrompido por cansaço ou possíveis complicações, com aquisição de imagens e dados ecocardiográficos em repouso e após o esforço imediato. É preciso tomar cuidado para não prolongar em demasiado o exame pós-esforço, que idealmente deve ser realizado em até 90 segundos para a captura das imagens.

## 12. Ecocardiografia de Estresse na Doença Valvar Aórtica

### 12.1. Estenose Aórtica

Estenose valvar aórtica é uma patologia crescente em nosso meio, especialmente devido à degeneração valvar senil, devido ao envelhecimento populacional.

A estenose aórtica (EAO) importante é definida quando a área valvar (AVA) é ≤ 1,0 cm^2^, e normalmente o gradiente de pressão através da valva está correlacionado com o fluxo transvalvar e o orifício valvar.^132^

Para obter uma avaliação valvar completa, é necessário avaliar o gradiente e o fluxo transvalvar aórtico, além da AVA em repouso e sob estresse, especialmente quando o fluxo transvalvar está reduzido, uma vez que se a AVA estiver menor que 1 cm^2^ com um gradiente transvalvar elevado em repouso (gradiente médio > 40 mmHg) e V_máx_ VE/AO > 4,0 m/s), o diagnóstico de EAO importante já está estabelecido.^114^

Entre as quatro categorias da estenose aórtica (alto gradiente; *low-flow, low-gradient* clássica [LFLG]; *low-flow, low-gradient* paradoxal e *normal-flow, low gradient*), a que mais carece da ecocardiografia de estresse é a LFLG clássica, ou seja, com FE reduzida. Um baixo gradiente em repouso na vigência de AVA reduzida não exclui EAO grave, que pode ocorrer pela FE estar reduzida, mas outra possibilidade é a EAO de baixo fluxo com FE preservada (paradoxal).

**Figura 12.1:**
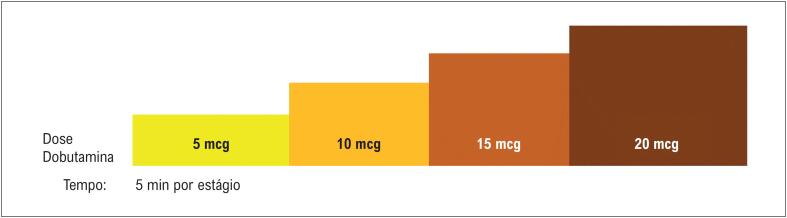
Protocolo de dobutamina em baixa dose utilizado na estenose aórtica low flow-low gradient clássica.

A ecocardiografia de estresse é indicada de fato nesses casos quando a FE está reduzida, ou seja, AVA ≤ 1,0 cm^2^, FE < 50% e gradiente médio transvalvar aórtico < 40 mmHg. Nesses casos, torna-se importante diferenciar a estenose valvar aórtica fixa verdadeiramente grave da pseudoestenose aórtica, na qual o problema principal é a cardiomiopatia superestimando a lesão valvar aórtica devido à abertura inadequada dessa valva, secundária ao comprometimento miocárdico.^133-135^ O protocolo de dobutamina utilizado se encontra na Figura 12.1.

**Figura 12.2:**
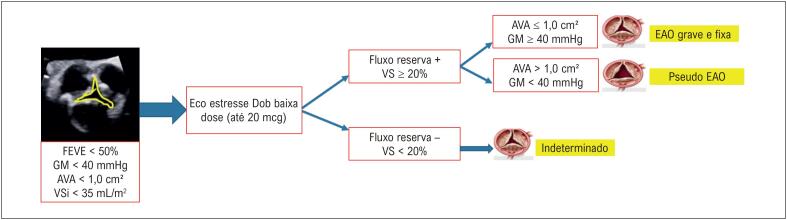
Indicação da ecocardiografia de estresse com dobutamina na estenose aórtica low-flow, low-gradient clássica e as possíveis respostas do teste de acordo com a presença ou ausência de reserva de fluxo. EAO: estenose aórtica; Dob: dobutamina; FEVE: fração de ejeção do ventrículo esquerdo; AVA: área valvar aórtica; VSi: volume sistólico indexado; GM: gradiente médio.

Na EAO LFLG clássica, a EED em baixa dose é indicada para avaliar a presença de reserva contrátil/reserva de fluxo. Quando presente, ela permite avaliar a gravidade da EAO, na qual três padrões de resposta são possíveis^133^ (Figura 12.2):

1. Aumento da reserva de fluxo ≥ 20% com aumento evidente da AVA para > 1,0 cm^2^ e pequeno aumento no gradiente transvalvar, indicando pseudoestenose aórtica, onde o tratamento clínico deve ser recomendado.
2. Aumento da reserva de fluxo ≥ 20% sem modificação na AVA, mantendo-se ≤ 1,0 cm^2^, e aumento do gradiente transvalvar aórtico (geralmente para gradiente médio acima de 40 mmHg), indicando estenose aórtica grave e fixa, na qual a intervenção valvar é recomendada.
3. Quando não há aumento adequado da reserva de fluxo (o volume sistólico aumenta menos de 20%) e, portanto, os demais parâmetros praticamente não mudam e a análise fica comprometida, o exame é indeterminado. Nesse caso, a tomografia com escore de cálcio ajuda a estimar a gravidade da EAO. Esse cenário ocorre em um terço dos pacientes e implica pior prognóstico. Contudo, esse padrão não deve contraindicar a intervenção valvar, uma vez que muitos pacientes melhoram o desempenho cardíaco após a troca valvar.

Uma sugestão de Clavel et al.^136^ para volume sistólico < 20% durante a EED é calcular a área valvar projetada considerando um fluxo normal estimado. Em outras palavras, o fluxo é obtido pelo volume sistólico/FE e a AVA projetada a uma taxa de fluxo normal que é padronizada como 250 mL/s e pode ser calculada pela seguinte fórmula: AVA projetada = AVA repouso + (alteração AVA/Alteração de fluxo) × (250 – taxa de fluxo), onde AVA projetada ≤ 1,0 cm^2^ indica EAO grave.^136-138^ O aumento > 20 bpm à EED, ou a presença de arritmias ou hipotensão arterial deve sinalizar a interrupção do teste.

Para os pacientes com EAO LFLG paradoxal, com FE ≥ 50%, AVA ≤ 1,0 cm^2^, gradiente médio < 40 mmHg e volume sistólico indexado ≤ 35 mL/m^2^, a EED não é oficialmente recomendada, dada a limitação nas evidências científicas e a possibilidade de hipotensão arterial importante por risco de gradiente dinâmico na VSVE. Tomografia com escore de cálcio demonstrando valores ≥ 2000 unidades de Agatston em homens e ≥ 1200 em mulheres sugere a possibilidade de EAO significativa. Além disso, valores ≥ 3000 em homens e ≥ 1600 em mulheres confirmam EAO significativa.^137^

**Figura 12.3:**
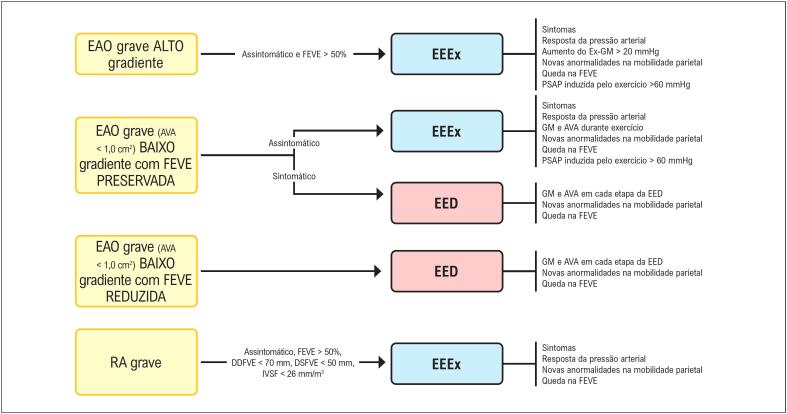
Diferentes modalidades de ecocardiografia de estresse e parâmetros a serem analisados de acordo com o tipo de lesão valvar aórtica. EEEx: ecocardiografia de estresse com exercício; EED: ecocardiografia de estresse com dobutamina; RA: regurgitação aórtica; EAO: estenose aórtica; AVA: área valvar aórtica; Ex: exercício; Ex-GM: gradiente médio durante o exercício; DDFVE: diâmetro diastólico final do ventrículo esquerdo; FEVE: fração de ejeção do ventrículo esquerdo; DSFVE: diâmetro sistólico do ventrículo esquerdo; IVSF: índice de volume sistólico final do ventrículo esquerdo; PSAP: pressão sistólica da artéria pulmonar.

Para pacientes assintomáticos com EAO grave de alto gradiente, a ecocardiografia de exercício preferencialmente em ciclomaca tem sido considerada capaz de adicionar valor prognóstico além do teste ergométrico isoladamente.^99^ A presença de um "salto" no gradiente, ou seja, elevação no gradiente transvalvar médio > 20 mmHg sob exercício, reflete menor complacência valvar e maior gravidade da lesão.^99,139^ Esses pacientes apresentam maior risco de eventos cardiovasculares. Outro achado que é revelado pela ecocardiografia de estresse é o aumento da pressão pulmonar sistólica > 60 mmHg. Contudo, ainda não há dados robustos para a indicação de rotina da ecocardiografia de estresse com exercício nesses pacientes^110^ (Figura 12.3).

### 12.2. Insuficiência Aórtica

Na insuficiência aórtica (IAO) grave e crônica, o paciente pode ainda estar assintomático e evoluir com disfunção ventricular latente ou explícita, que pode ser irreversível.

Nesses casos, a ecocardiografia de estresse com exercício ou dobutamina consegue identificar a presença ou ausência de reserva contrátil (aumento da FE em pelo menos 5% ou do *strain* longitudinal em valores absolutos ≥ 2%) ou reserva de fluxo (aumento no volume sistólico ≥ 20%) antes da intervenção. Esse dado é preditor de recuperação funcional após troca valvar aórtica.^110,140^

Principalmente devido ao pequeno número de estudos disponíveis, A validação da ecocardiografia de estresse para prever resultados nesse subgrupo de pacientes assintomáticos com IAO grave é limitada.^114,141^

## 13. Ecocardiograma de Estresse na Cardiomiopatia Dilatada

A cardiomiopatia dilatada (CMD) é uma doença cardíaca primária caracterizada por dilatação miocárdica na ausência de outras etiologias previamente conhecidas e disfunção sistólica. Dado o número crescente de casos diagnosticado, a avaliação do estado atual e o prognóstico do paciente se faz cada vez mais importante, pois a evolução no tratamento possibilita o aumento da sobrevida.

A ecocardiografia de estresse tem se mostrado de grande importância nesse grupo para avaliação etiológica, diagnóstico e estratificação prognóstica, por meio da análise da reserva contrátil; do estado funcional dos pacientes em condições de estresse induzido, daqueles que se beneficiarão do tratamento com betabloqueadores e da identificação de pacientes com pior prognóstico em termos de morte cardíaca e necessidade de transplante.

Apesar de não haver um consenso estabelecido sobre o protocolo, observa-se o uso frequente de dobutamina em altas doses (até 40 mcg/kg/min) sem o acréscimo de atropina, principalmente por evocar uma resposta contrátil mais completa e possibilitar a avaliação da reserva inotrópica nesses pacientes. Muitas vezes, a terapia inclui o uso de betabloqueadores, o que diminui a probabilidade de resultados falso-negativos, sem agregar maiores complicações.^99^ Sugere-se que o aumento da contratilidade, expresso pela variação do WMSI no pico da dose de dobutamina e os valores basais, e o aumento ≥ 5% da FEVE durante a infusão de dobutamina estejam relacionados a uma melhor taxa de sobrevida, baixa taxa de hospitalização por insuficiência cardíaca e aumento da FEVE durante o seguimento. Pacientes com reserva contrátil respondem melhor à terapia com betabloqueador, têm menor necessidade de transplante e apresentam relação inversa à extensão de fibrose intersticial.^99,142-146^ Entretanto, a ausência de reserva contrátil em pacientes com FEVE preservada ou reduzida com frequência está associada a RFC limitado, sendo portanto, um marcador de disfunção sistólica latente do VE e cardiomiopatia subclínica.^99^

O evento adverso associado com mais frequência a altas doses do medicamento é a arritmia ventricular complexa (extrassístole ventricular multifocal em 6,4% e taquicardia ventricular não sustentada em 1,6%), geralmente associados a pacientes com hipocalemia.^142,143^

O protocolo com vasodilatador (dipiridamol 0,84 mg por 10 min), com acréscimo do *handgrip* no pico de sua infusão (aumentando a sensibilidade do teste), raramente é utilizado para avaliar reserva contrátil, mas tem sido proposto como alternativa à dobutamina por ser menos arritmogênico, melhor tolerado e apresentar informações prognósticas semelhantes aos pacientes com doença isquêmica. Além de não ter a influência do uso de betabloquedor^147^ para a análise específica de reserva contrátil, contudo, não ganhou grande repercussão na prática clínica para essa indicação em comparação com a dobutamina.^49,148-150^

As Tabelas 13.1, 13.2, 13.3, 13.4 e 13.5 apresentam as recomendações a serem realizadas junto à ecocardiografia de estresse na CMD.

**Tabela 13.1:** Parâmetros a avaliar na ecocardiografia de estresse na cardiomiopatia dilatada

**1. O que avaliar na ecocardiografia de estresse na cardiomiopatia dilatada?**	
- Reserva contrátil	- Reserva diastólica
- Isquemia estresse induzida	- Regurgitação mitral dinâmica
- Variação na PSAP	- Congestão pulmonar (linha B)

**Tabela 13.2:** Parâmetros a medir na ecocardiografia de estresse na cardiomiopatia dilatada

**2. O que medir?**
- Ondas E e A
- e´(Doppler PW)
- Regurgitação mitral e tricúspide (Doppler CW)

**Tabela 13.3:** Momentos do teste para aferições na cardiomiopatia dilatada

**3. Quando medir (protocolo dobutamina)?**
- Basal - Baixa dose - Pico da infusão da medicação

**Tabela 13.4:** Respostas esperadas na ecocardiografia de estresse na cardiomiopatia dilatada

**4. Respostas esperadas:**
- Aumento ou não da contratilidade do VE
- Redução ou aumento da regurgitação mitral
- Variações da PSAP e relação E/e’
- Presença ou ausência de linhas B nos campos pulmonares

**Tabela 13.5:** Protocolos utilizados (A-dobutamina e B-dipiridamol) na ecocardiografia de estresse na cardiomiopatia dilatada

**Figura 12.4:**
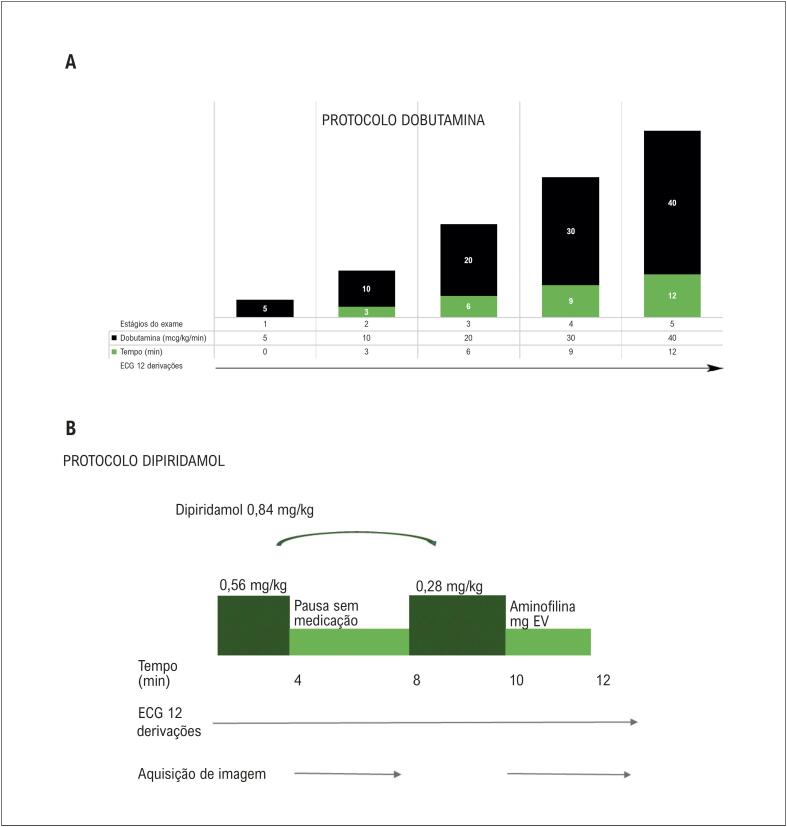
Protocolos utilizados (A: dobutamina e B: dipiridamol) na ecocardiografia de estresse na cardiomiopatia dilatada.

**5. Protocolos utilizados** (Figura 12.4).

## 14. Ecocardiografia de Estresse na Cardiomiopatia Chagásica

Pacientes com cardiopatia crônica da doença de Chagas (CCDC) frequentemente manifestam dor torácica atípica e apresentam anormalidades eletrocardiográficas, tais como alterações no segmento ST e ondas Q patológicas, além de distúrbios regionais de contratilidade e de perfusão miocárdica que simulam a apresentação da doença coronária aterosclerótica.^151^

Até poucas décadas atrás, os pacientes com doença de Chagas eram predominantemente trabalhadores rurais, com baixo perfil de risco para doença obstrutiva coronária. Com a urbanização crescente dessa população, a partir principalmente de 1980, eles passaram a ser expostos aos mesmos fatores de risco para doença aterosclerótica que os indivíduos não infectados pelo *Trypanosoma cruzi*.^152^ Entretanto, na maioria dos casos, a avaliação das coronárias epicárdicas revela a ausência de obstrução aterosclerótica subepicárdica significativa, com tais modificações atribuídas à disfunção microvascular coronariana.^153^

Alguns estudos avaliaram a aplicabilidade da ecocardiografia de estresse na doença de Chagas. A isquemia microvascular é considerada um distúrbio precoce na evolução da doença, precedendo a disfunção ventricular regional e possivelmente relacionada à indução de hibernação ou atordoamento miocárdico. Resultados obtidos por estudo de ecocardiografia de estresse utilizando vasodilatador (dipiridamol) demonstram diminuição da reserva vasodilatadora coronária, um índice de disfunção microvascular, em pacientes com a forma indeterminada da doença em comparação com controles normais.^154^

Quanto à resposta cronotrópica, a ecocardiografia com dobutamina demonstra que pacientes com doença de Chagas apresentam uma menor resposta inotrópica e cronotrópica quando comparados a um grupo controle. Além disso, a ecocardiografia de estresse farmacológico pode demonstrar a presença de reserva contrátil bifásica nesses pacientes, apesar da ausência de doença coronária epicárdica.^155^

Em relação ao valor preditor para a evolução da cardiopatia, a presença de arritmias ventriculares complexas na EEF foi um preditor independente de evolução da doença em um grupo de pacientes na fase inicial da cardiopatia.^156^

Por fim, no que se refere ao emprego da dobutamina em uma cardiopatia arritmogênica, a ecocardiografia de estresse farmacológico evidenciou segurança na cardiopatia chagásica em uma coorte de pacientes em investigação para doença coronária. A presença do índice de contração segmentar alterado em repouso foi um preditor independente para o surgimento de arritmias complexas durante o exame.^157^

## 15. Ecocardiografia de Estresse na Cardiomiopatia Hipertrófica

A CMH é uma doença cardiovascular genética comum com padrão autossômico dominante e distribuição igual por sexo. A prevalência varia com a idade, estado subclínico ou clínico e diferenças raciais.^158^ A suspeita clínica pode surgir pela manifestação de sintomas, detecção de sopro cardíaco, padrão eletrocardiográfico anormal ou um evento cardíaco. Além disso, pode ser um achado incidental durante a realização de ecocardiografia por outras indicações.^159^

A ecocardiografia desempenha um papel muito importante na confirmação do diagnóstico, determinação do padrão de hipertrofia, avaliação da obstrução da VSVE, movimentação anterior sistólico (MAS) da valva mitral e regurgitação mitral (RM), bem como na avaliação da função sistólica e diastólica do VE, do volume atrial esquerdo e da pressão pulmonar.^160^

A EEF é amplamente utilizada para medir a obstrução da VSVE dinâmica e indutível em pacientes sintomáticos com gradiente de pico da VSVE em repouso < 50 mmHg, para detectar RM e para monitorar a resposta ao tratamento. A importância dos gradientes > 50 mmHg, seja em repouso ou pós-esforço, reside no fato de haver um limite convencional para intervenção invasiva quando os sintomas não estão sob controle com farmacoterapia.^158^ O estresse sob exercício é o mais recomendado nesses casos.

A EEF pode ser realizada em ciclomaca semissupina, em bicicleta ou esteira. Sugere-se que a esteira provoca maior gradiente de VSVE. A escolha do protocolo deve considerar o estado geral do paciente, a experiência do médico e os recursos da instituição.

Antes do teste, é importante estar ciente das condições do paciente (classe funcional, estado de hidratação, horário de alimentação, bebidas alcoólicas, etc.) e dos fatores farmacológicos (vasodilatadores, diuréticos), pois podem influenciar o andamento do exame. Ao contrário de outras modalidades de estresse, os betabloqueadores não devem ser retirados antes do exercício para esse tipo de avaliação.

Os ARUS são úteis na confirmação de hipertrofia septal e apical, na detecção de trombos apicais e na exclusão de aneurismas apicais.^161^

É fundamental orientar o paciente sobre o objetivo do estudo e possíveis eventos. Como um dos objetivos da ecocardiografia de estresse é quantificar o gradiente da VSVE em pacientes sintomáticos sem gradiente ou com gradiente baixo em repouso, recomenda-se medir o gradiente com a maior frequência possível durante o exame. Além disso, é preciso avaliar SAM, RM, anormalidades de movimento da parede do VE e disfunção diastólica.

As alterações eletrocardiográficas devem ser monitoradas. Os parâmetros clínicos e ecocardiográficos são avaliados durante o exercício e na fase de recuperação precoce e tardia, enquanto a pré-carga diminui gradativamente. Se a EEF não provocar um aumento significativo no gradiente da VSVE, a avaliação do gradiente no ortostatismo pós-exercício deve ser considerada. Resposta hipertensiva, redução da PA superior a 10 mmHg em comparação com a PA de repouso antes do exame, dispneia, desconforto torácico e taquiarritmia sustentada são indicações para interrupção do exame.^162^ A obstrução da VSVE com baixa carga de exercício está correlacionada com uma baixa capacidade funcional. A obstrução da VSVE é um fator de risco para morte súbita e eventos cardiovasculares graves.^163^

Como a ecocardiografia de estresse é uma técnica que depende da experiência do operador, uma curva de aprendizado supervisionada é imprescindível para desenvolver experiência na avaliação de CMH, a fim de obter resultados confiáveis e reprodutíveis que permitam a tomada de decisões sobre o manejo e seguimento dos pacientes. Sabe-se que o curso clínico da CMH é heterogêneo e imprevisível. A ecocardiografia de estresse tem o potencial de diferenciar a CMH não obstrutiva das formas com gradiente dinâmico e, aliada à capacidade de exercício, à PA e à resposta da FC, prever o desenvolvimento futuro de insuficiência cardíaca de modo a direcionar as atuais estratégias de manejo disponíveis de acordo com cada paciente no contexto dessa doença genética complexa. Procedimentos como miectomia septal cirúrgica e ablação septal percutânea com álcool são considerados indicações Classe I para pacientes sintomáticos com gradiente em VSVE em repouso e aqueles sem resposta aceitável à terapia farmacológica.^164^

## 16. Ecocardiografia de Estresse na Avaliação da Função do Ventrículo Direito e na Hipertensão Pulmonar

A hipertensão pulmonar (HP) é um distúrbio fisiopatológico que pode envolver diversas condições clínicas e estar associada a inúmeras doenças respiratórias e/ou cardiovasculares. Independentemente do processo fisiopatológico subjacente e do local das alterações funcionais, a HP é definida como uma PA pulmonar média de repouso (PAPm) de > 20 mmHg medida por cateterismo cardíaco direito (CCD).^165^

Recentemente, tem crescido o interesse pela sua aplicação na detecção e avaliação da gravidade da HP e disfunção cardíaca direita.^166,167^

O CCD é o padrão-ouro para avaliar a hemodinâmica pulmonar e possibilita diagnosticar e classificar a HP. De acordo com o consenso atual, o CCD de exercício é o método recomendado para avaliar a hemodinâmica cardiopulmonar. No entanto, ele acarreta alguns desafios técnicos, exigindo profissionais experientes, equipamentos especiais e tempo adicional de procedimento.^165^

A ecocardiografia de estresse da circulação pulmonar e do ventrículo direito (VD) revela diferenças relevantes do ponto de vista prognóstico entre indivíduos saudáveis, atletas, residentes de altitude e pacientes com diversas condições cardiorrespiratórias, podendo avaliar a circulação pulmonar e o comportamento do ventrículo direito nesses indivíduos, além de auxiliar no diagnóstico de HP latente ou evidente, com ou sem alteração da função do VD, adaptando-se ao aumento da carga.^168^

Semelhante ao conceito de CCD de exercício, a complementação com um teste de estresse à ecocardiografia Doppler padrão pode melhorar a acurácia diagnóstica para detectar HP pós-capilar. Além disso, a ecocardiografia de estresse físico agrega valor no manejo de pacientes com HP, pois também nos permite avaliar a mecânica complexa do VD (por exemplo, reserva contrátil) e a capacidade da vasculatura pulmonar de acomodar o fluxo aumentado.^168^

As medidas ecocardiográficas da função do VD que são preditores independentes de mortalidade na HP incluem a excursão sistólica no plano anular tricúspide (TAPSE < 18 mm),^169-173^ a mudança de área fracionada do VD (FAC < 35%, a velocidade anular lateral do pico sistólico tricúspide (S’ < 9,7 cm/s^174^) e o índice de Tei (> 0,40 pelo Doppler pulsátil ou > 0,55 pelo Doppler tecidual^175^).

Todas as métricas descritas acima podem ser avaliadas em repouso e após o pico de estresse (exercício ou com ecocardiografia com dobutamina). A maioria dos dados até o momento foi derivada de voluntários saudáveis, mas vários estudos de pequeno e médio porte avaliam o papel prognóstico da reserva contrátil do VD em pacientes com HP, doença valvar e insuficiência cardíaca.^176^

Não se sabe se a avaliação ecocardiográfica não invasiva é igualmente confiável e geralmente viável para definir condições de doença com ou em risco de HP. Limitações semelhantes se aplicam à avaliação ecocardiográfica da função do VD durante o exercício.^167,176^

Uma medida possível e não invasiva da reserva contrátil do VD por meio da ecocardiografia de estresse físico foi proposta por Grünig et al., que demonstraram que um aumento da PA pulmonar (PAP) induzido pelo exercício foi uma medida da reserva contrátil do VD e fator prognóstico independente em pacientes com HP pré-capilar.^177^

## 17. Ecocardiografia de Estresse na Avaliação Pós-COVID-19

A maioria das publicações a respeito das manifestações cardiovasculares da COVID-19 avalia a fase aguda da infecção, onde se demonstrou que a presença de disfunção cardíaca detectada à ecocardiografia de repouso estava associada a pior prognóstico em pacientes com COVID-19 grave.^178^ Pelo alto risco da contaminação cruzada por gotículas, o uso da EEF foi adiado ou cancelado durante a pandemia.^179,180^ Em pacientes de baixo risco para COVID-19 nos quais a indicação era apropriada e o adiamento não era possível, deu-se preferência à ecocardiografia de estresse farmacológico.^179,180^

Após o fim do estado de emergência da pandemia, diversos estudos tentaram descrever as consequências da COVID-19 para o sistema cardiovascular no longo prazo, mas a maioria ainda empregou apenas a ecocardiografia de repouso. Posteriormente, algumas poucas publicações exploraram o uso da ecocardiografia de estresse como ferramenta para a avaliação de pacientes após a recuperação da infecção por SARS-CoV-2. Um estudo prospectivo em 174 atletas jovens com função ventricular normal ao repouso após COVID-19 visou identificar envolvimento miocárdico por meio de um amplo protocolo funcional que incluía EEF.^181^ Sinais de envolvimento miocárdico foram encontrados em cinco atletas (2,9%), três dos quais mostraram alterações clinicamente significativas ao esforço, incluindo arritmia ventricular em dois e alteração da contratilidade segmentar em um indivíduo.^181^ Embora raro, o comprometimento miocárdico só foi detectado durante o esforço, sugerindo que a triagem com EEF pode ser útil em casos selecionados para a orientação ao retorno da atividade atlética competitiva após infecção por SARS-CoV-2. Outro estudo avaliou 71 pacientes recuperando-se da COVID-19, a maioria sintomáticos, por meio de um protocolo que combinava EEF e teste cardiopulmonar.^182^ O consumo de oxigênio (VO_2_) no pico foi menor em pacientes pós COVID-19 em comparação com o grupo controle devido a uma combinação de incompetência cronotrópica e reserva atenuada do volume sistólico do VE (aumento insuficiente do *stroke volume* durante o esforço).^182^ Esses estudos ressaltam a importância de avaliar a função cardíaca em repouso e também em situações de estresse, como o exercício, para compreender completamente o impacto da infecção por SARS-CoV-2 no coração. Investigações em andamento poderão, por meio da utilização da ecocardiografia de estresse, nos ajudar a entender melhor a relação entre a história de infecção por SARS-CoV-2, suas implicações cardiovasculares e a atividade física.^183^

Menção especial deve ser feita ao uso de ARUS, ferramentas indispensáveis para a prática moderna da ecocardiografia de estresse. Após dezembro de 2020, coincidente com o início da vacinação contra COVID-19 na população americana, observou-se um aumento das reações adversas graves aos ARUS contendo polietilenoglicol (PEG) em sua composição, como Sonovue ou Lumason (Bracco Diagnostics) e Definity (Lantheus Medical Imaging). Cogitou-se como substrato a possibilidade de reatividade cruzada entre esses ARUS e as vacinas de COVID-19 baseadas em RNA mensageiro (Pfizer/BioNTec e Moderna), uma vez que ambos os grupos de fármacos contêm PEG.^184^ Um estudo retrospectivo caso-controle apontou que indivíduos com reações adversas graves ao Lumason ocorridas desde março de 2021 nos EUA tinham sido vacinados para COVID-19 mais frequentemente do que aqueles sem reações adversas.^185^

## 18. Ecocardiografia de Estresse e Reserva de Fluxo Coronariano nos Vasos Nativos

A RFC pode ser avaliada por meio da análise direta do fluxo da artéria durante a ecocardiografia de estresse. *Presets* específicos permitem visualizar, sem uso de microbolhas, a ADA durante a ecocardiografia de estresse com adenosina, dipiridamol ou mesmo com dobutamina, em pelo menos 90% dos casos, com menor taxa de sucesso na descendente posterior (DP) e circunflexa (CX), particularmente na EEF.^23,186-189^

**Figura 18.1:**
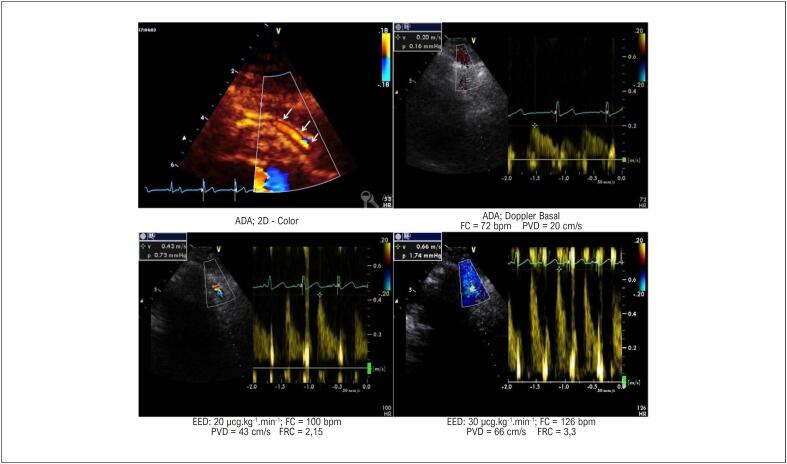
Obtenção da RFC basal normal na artéria descendente anterior e que aumenta significativamente (RFC = 3,3) com 30 mcg/kg/min de dobutamina. ADA: artéria coronária descendente anterior; EED: ecocardiografia de estresse com dobutamina; FC: frequência cardíaca; PVD: pico de velocidade diastólica; RFC (reserva de fluxo coronariano) = PVD no estresse **/** PVD basal. RFC normal (≥ 2) e precoce obtido antes de alcançar a FC alvo do exame, registrado em estágios intermediários do EED.

O cálculo da RFC consiste na divisão da velocidade diastólica obtida na hiperemia pela velocidade diastólica registrada em repouso (Figura 18.1). Na correlação entre a RFC obtida na ecocardiografia de estresse e o percentual de estenose coronariana, RFC ≥ 2 indica normalidade, compatível com bom prognóstico. A RFC pode estar reduzida (< 2) por estenose coronariana e/ou microcirculação comprometida, sendo preditor independente de desfecho adverso. Valores mais baixos da RFC são mais indicativos de estenose coronariana importante (Tabela 18.1).^187,190-194^

**Tabela 18.1:** Correlação entre reserva de fluxo coronariano e percentual de estenose coronariana

Autores	Nº casos	RFC (cut off)	Estressor	% Estenose	Sensibilidade	Especificidade	Acurácia
Nohtomi et al.^187^	110 ADA	< 2	Dipiridamol	> 50%	94%	65%	81%
Voci et al.^190^	44 (ADA+DP)	< 2	Adenosina	≥ 70%	89%	96%	93%
	18 DP	1,4 ± 0,54		≥ 70%	89%	91%	93%
	19 ADA	1,12 ± 0,49		≥ 70%	89%	100%	95%
Lowenstein et al.^191^	132 ADA	< 2	Dipiridamol	> 70%	87%	73%	80%
Matsumara et al.^192^	138 ADA	-	Adenosina				
	30 ADA	< 2		≥ 70%	90%	93%	
Soylu et al.^193^	44 ADA	< 1,6	Dipiridamol	≥ 55%	100%	94%	
Hyodo et al.^194^	175 artérias	-	Adenosina	> 50%			
	166 ADA	< 2		> 50%	86%	91%	
	149 DP	< 2		> 50%	92%	92%	
	142 CX	< 2		> 50%	91%	92%	

RFC: Reserva de fluxo coronariano; ADA: Artéria coronária descendente anterior; DP: Descendente posterior; CX: Circunflexa.

Na estenose intermediária (50% - 70%) da ADA, pode-se optar pela intervenção hemodinâmica quando a RFC é reduzida, ou pelo tratamento clínico se a RFC for normal, visto que no estudo de D’Andrea et al. não houve diferença na ocorrência de óbito, infarto, angina instável ou revascularização miocárdica nos dois grupos.^195^

A RFC anormal evidencia sensibilidade e especificidade elevadas para diagnóstico de restenose nas três principais coronárias (Tabela 18.1). Um estudo mostrou exequibilidade de registro elevada na ADA (95%), DP (85%) e CX (81%) e utilizou ARUS ou contraste de microbolhas em 22% dos casos.^194^

A dobutamina é o estressor mais utilizado para a ecocardiografia de estresse farmacológico e apresenta também ação vasodilatadora.^196^ O primeiro estudo a avaliar a RFC na ADA com dobutamina mostrou exequibilidade de 90%. Os casos com isquemia pelo critério de anormalidade contrátil apresentaram RFC = 1,51 ± 0,51, enquanto os sem isquemia tiveram RFC = 2,76 ± 0,65.^186^

Durante a ecocardiografia de estresse com dobutamina, uma RFC normal e precoce pode ser obtida antes de atingir a FC alvo do exame.^197^ Os casos com RFC normal e precoce apresentaram melhor desfecho combinado do que as RFCs normais registradas após a FC submáxima e as RFC anormais. Após obter uma RFC precoce e normal, o registro desta pode ser interrompido e apenas a ecocardiografia de estresse continuada.^179^

Em pacientes diabéticos submetidos a ecocardiografia de estresse com dipiridamol ou dobutamina, verificou-se, nos casos sem isquemia, que a RFC anormal na ADA foi preditor independente de mortalidade e similar por ambas as técnicas.^199^ A observação foi corroborada em um estudo com dipiridamol que avaliou a RFC em ADA e DP, constatando que a RFC anormal é preditor de pior prognóstico (óbito e infarto do miocárdio) em pacientes sem isquemia.^189^

## 19. Ecocardiografia de Estresse e Reserva de Fluxo Coronariano em Enxertos

A análise anatômica da lesão de vasos epicárdicos por coronariografia pode não ser suficiente para avaliar a repercussão da lesão coronariana, principalmente em obstruções intermediárias, de 50 a 70%, e em pacientes multiarteriais,^200^ em parte devido às modificações que a estenose provoca na regulação da circulação. Uma estenose > 50% da luz aumenta a resistência coronária e diminui a pressão após a obstrução, com diminuição da RFC.^201^ Por esse motivo, a avaliação fisiológica da circulação coronariana, como determinante da revascularização miocárdica, tem melhorado os resultados dos procedimentos, tanto cirúrgicos como percutâneos, assim como o prognóstico pós procedimento. Nesse sentido, entre outros métodos diagnósticos, além da coronariografia, destacam-se a ecocardiografia de estresse e a análise da RFC com Doppler transtorácico.

### 19.1. Ecocardiografia de Estresse após Revascularização Miocárdica

Assim como o WMSI > 1,7 na ecocardiografia de estresse realizada antes da revascularização é preditor de aumento das complicações pós-operatórias,^202^ um estudo anormal após a revascularização aumenta mais de duas vezes o risco de eventos cardiovasculares adversos,^203^ mesmo em pacientes assintomáticos. Apesar da ecocardiografia de estresse com vasodilatador ser a melhor opção para esses casos, o estresse com dobutamina em baixa dose, embora influenciado por diversos fatores, tem demonstrado boa sensibilidade (86,4%) para detectar a recuperação da reserva contrátil após a revascularização, principalmente em segmentos previamente hipocinéticos.^204^

### 19.2. Reserva de Fluxo Coronariano após Revascularização Miocárdica

**Figura 19.1:**
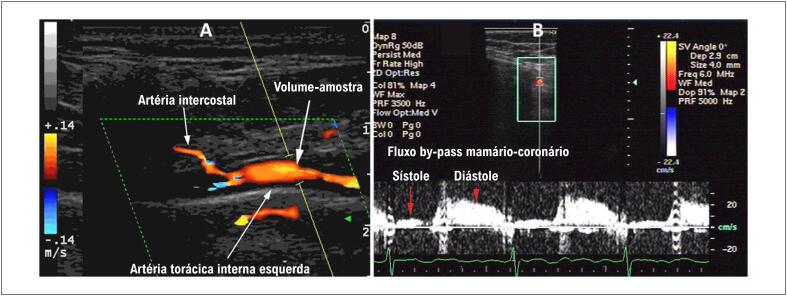
A: detecção do fluxo da artéria torácica interna com posicionamento do volume-amostra do Doppler pulsátil. B: registro do fluxo do enxerto mamário-coronário observando-se fluxo bifásico com predomínio diastólico, indicando que o enxerto se encontra pérvio.

**Figura 19.2:**
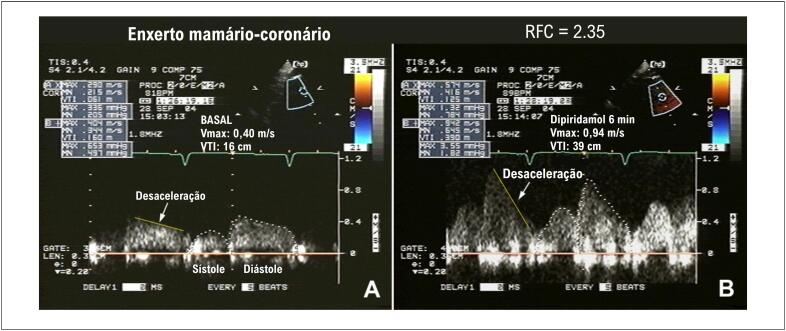
Fluxo coronariano em enxerto com artéria torácica interna. A: fluxo coronariano basal obtido no ramo descendente anterior após a anastomose. B: fluxo obtido 6 minutos após a infusão de dipiridamol mostrando uma reserva de fluxo coronariano (RFC) de 2,35. A rampa de desaceleração do traçado diastólico correlaciona-se com a resistência oferecida pelo leito vascular à passagem do fluxo durante a diástole. A vasodilatação diminui a resistência vascular com desaceleração mais rápida do fluxo.

A RFC fracionada realizada durante o estudo hemodinâmico, seja pelo gradiente pressórico ou pela velocidade do fluxo através da obstrução, é uma ferramenta importante para a análise funcional da doença coronariana obstrutiva, sendo recomendado como Classe IA.^205^ Em pacientes revascularizados, como substituto dos métodos invasivos, é possível usar a medição do fluxo coronariano com Doppler transtorácico e a RFC com vasodilatadores.^206^ Em enxertos realizados com artéria torácica interna (mamária interna), a ocorrência de fluxo bifásico com predomínio diastólico na artéria mamária indica patência do enxerto (Figura 19.1). O aumento da velocidade do fluxo coronariano distal ao local do enxerto após a infusão de vasodilatadores (usa-se dipiridamol 0,84 mg/kg em 6 minutos, embora a obtenção da RFC já seja considerada satisfatória em 4 minutos; ou adenosina 140 mcg/kg/min durante 2 minutos) permite determinar a RFC, sendo considerada boa resposta uma relação entre o fluxo basal e com hiperemia > 2^207^ (Figura 19.2).

**Figura 19.3:**
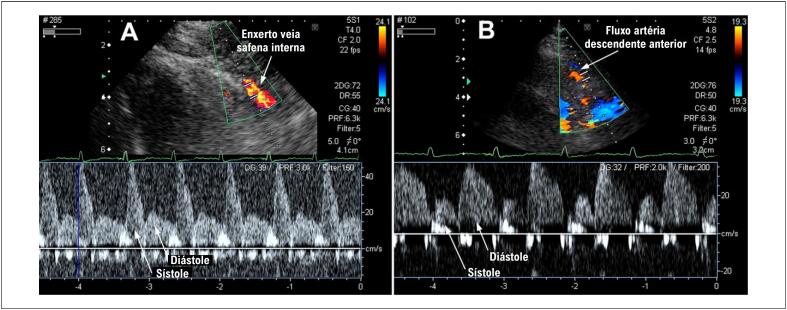
Fluxo coronariano em enxerto venoso para o ramo descendente anterior. A: fluxo no enxerto venoso, com predomínio sistólico. B: fluxo na artéria distal ao enxerto com fluxo bifásico com predomínio diastólico.

Em enxertos venosos, o fluxo no enxerto é predominantemente sistólico, sofrendo esvaziamento para a artéria coronária durante a diástole (Figura 19.3). A análise e interpretação da RFC é semelhante à do enxerto arterial.

Algumas condições podem diminuir a RFC nos enxertos arteriais sem necessariamente haver estenose, como: ateromatose da artéria subclávia esquerda ou do enxerto arterial que pode diminuir o fluxo para a artéria enxertada; ateromatose difusa do leito distal da artéria irrigada; infarto prévio com aumento da resistência microvascular; alterações da microcirculação coronariana; e momento da realização do exame, devido ao remodelamento gradativo dos enxertos arteriais, com aumento progressivo da RFC. Para os enxertos venosos, temos o espasmo do enxerto, condição rara, porém, grave; aterosclerose do enxerto; e hipoplasia fibro-intimal com fibrose.

## 20. Ecocardiografia de Estresse na Análise de Viabilidade Miocárdica

A abordagem clínica de áreas miocárdicas acinéticas com viabilidade por estudos de imagem (miocárdio hibernante) é controversa, principalmente após dois grandes estudos, o STICH (*Surgical Treatment for Ischaemic Heart Failure*) e o HEART (*Heart Failure Revascularization*) que, apesar de mostrarem melhora da FEVE, não impactaram positivamente no prognóstico desses pacientes no longo prazo.^208,209^ O miocárdio hibernante se refere a áreas disfuncionantes por hipoperfusão crônica, por doença coronariana obstrutiva, com possível reversibilidade após revascularização.^210^ Isso intriga a cardiologia e ainda é um tema controverso. Dentre os métodos de imagem, a tomografia por emissão de pósitrons (PET – *positron emission tomography*) tem a maior sensibilidade e valor preditivo negativo, enquanto a ecocardiografia de estresse farmacológico com dobutamina é a mais específica e com maior valor preditivo positivo por segmento analisado.^211^ Atualmente, há uma revisão conceitual em relação ao modo e possíveis benefícios da revascularização guiada pela análise prévia da viabilidade miocárdica. A concepção anterior valorizava a presença de viabilidade, independente da anatomia coronariana para revascularização. Essa estratégia demonstrava uma melhora da FE, da qualidade de vida, dos sintomas e da insuficiência cardíaca isquêmica, contudo, a FE em si não influenciava necessariamente a melhora da sobrevida. A abordagem atual visa uma avaliação mais funcional e específica ao miocárdio, melhorando o planejamento terapêutico com uma revascularização mais racional e fisiológica. Com isso, há uma preocupação maior em preservar os segmentos com isquemia e viabilidade que tenham uma anatomia favorável para revascularização cirúrgica, reduzindo, dessa forma, a taxa de infartos e arritmias fatais.^210^ Curiosamente, a revascularização percutânea guiada pela pesquisa de viabilidade não demonstrou benefícios.^212,213^

### 20.1. Protocolo de Estresse para Análise de Viabilidade Miocárdica

Antes do estudo de viabilidade miocárdica, a ecocardiografia transtorácica dirigida é realizada de forma habitual. Essa fase informa o examinador sobre os parâmetros basais a serem analisados sob condições dinâmicas, assim como sobre a qualidade da janela ecocardiográfica, antecipando a necessidade de utilização de agentes de realce ultrassonográfico. A avaliação de viabilidade é realizada pelo protocolo de ecocardiografia de estresse farmacológico com dobutamina em baixa dose (iniciando com 2,5 ou 5 mcg/kg/min com incrementos para 7,5 e 10 mcg/kg/min, a cada 3 a 5 minutos, chegando até 20 mcg/kg/min). O protocolo também pode ser realizado em pacientes em uso de betabloqueador. A despeito da avaliação de viabilidade com baixas doses de dobutamina, é interessante progredir com doses maiores (até 40 mcg/kg/min com ou sem atropina) para avaliar, além da viabilidade, a presença de isquemia miocárdica induzida por estresse que muito contribui para a mudança de conduta. Isso vale naqueles pacientes que permitem a evolução do protocolo, preferencialmente nos que não apresentam FE muito reduzida por hipocinesia acentuada de vários segmentos, além de áreas acinéticas. Nesses casos, o risco do protocolo em alta dose aumenta e a sensibilidade do método é reduzida para diagnóstico de isquemia pela alteração acentuada e "balanceada" de todas as paredes do VE.

**Figura 20.1:**
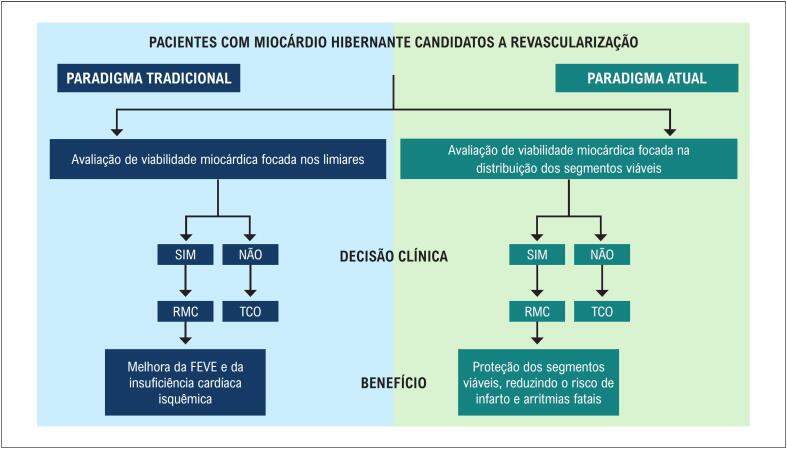
Algoritmo sintético de conduta na abordagem do miocárdio hibernante. FEVE: fração de ejeção do ventrículo esquerdo; RMC: revascularização miocárdica; TCO: terapia clínica otimizada.

A presença de viabilidade é reconhecida pela ecocardiografia transtorácica com dobutamina quando os segmentos acinéticos em repouso apresentam aumento da espessura (≥0,5 cm) e da contratilidade (reserva contrátil) ao estresse. Em pacientes que apresentam resposta bifásica com o avanço do protocolo (melhora em baixa dose e piora em dose mais elevada de dobutamina, 20 mcg/kg/min ou mais), o fenômeno sugere maior número de segmentos viáveis e com isquemia, chamado também de isquêmico-viável, e é a melhor resposta que denota recuperação funcional pós-revascularização. Isso vale especialmente quando essa viabilidade, chamada resposta bifásica, ocorre em quatro ou mais segmentos acinéticos em repouso, em paciente com disfunção sistólica do VE. Geralmente, esse grupo de pacientes apresenta melhores respostas à revascularização miocárdica, inclusive com melhora da FE, ao contrário daqueles que só apresentam melhora da contratilidade com baixas e altas doses de dobutamina (melhora sustentada); assim como aqueles que apresentam piora da contratilidade nas regiões hipocinéticas em baixas doses de estresse farmacológico^214,215^ (Figura 20.1).

### 20.2. Análise da Viabilidade Miocárdica pelo *Strain* Longitudinal

**Figura 20.2:**
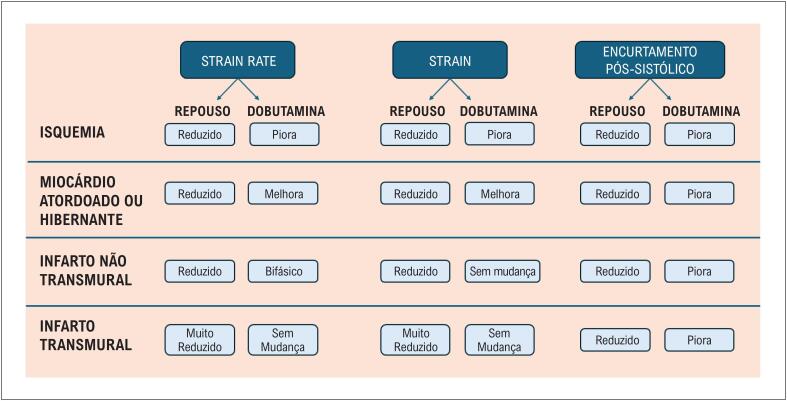
Principais alterações da análise de deformação miocárdica pelo strain longitudinal global na viabilidade miocárdica.

A avaliação da viabilidade miocárdica por meio da análise da deformação (*strain*) global longitudinal do VE por ecocardiografia bidimensional é uma área de pesquisa ativa e relevante. A medição do *strain* e do *strain rate* (SR) por ecocardiografia é uma técnica desafiadora, mas promissora para avaliar a função ventricular em repouso, viabilidade miocárdica e isquemia durante o teste de estresse. Em baixas doses de dobutamina, ocorre aumento do *strain* e do SR nos segmentos viáveis e redução do encurtamento pós-sistólico. Por outro lado, nos segmentos com acometimento transmural do infarto, observa-se apenas um aumento discreto e transitório do SR, sem aumento do *strain* global longitudinal e aumento do encurtamento pós-sistólico. As principais alterações da análise de deformação estão resumidas de forma sintética na Figura 20.2.^215-217^ Apesar dos resultados interessantes em agregar informações ao método, ainda faltam estudos que comprovem o seu benefício clínico, guiando a terapêutica a partir de dados incrementais da deformação miocárdica. Por isso, o *strain* global longitudinal não é atualmente uma técnica substitutiva.

Independentemente da decisão clínica de revascularização cirúrgica ou tratamento clínico, a presença e a extensão de segmentos viáveis demonstram um substrato para resposta terapêutica, como terapia de ressincronização cardíaca e terapia farmacológica, e não apenas a revascularização miocárdica.^218^

Assim, os pacientes que apresentam segmentos viáveis têm melhor resposta terapêutica e melhor prognóstico quando submetidos a intervenções clínicas ou cirúrgicas.

## 21. Ecocardiografia de Estresse com Agente de Realce Ultrassonográfico na Análise da Borda Endocárdica

Em todas as modalidades de estresse, o ARUS, nomenclatura mais recente para o contraste de microbolhas ou de ultrassom, pode e deve ser utilizado.

No caso da esteira, as imagens iniciais são obtidas em repouso e as imagens no pós-esforço imediato idealmente até 90 segundos após o término do exercício físico, quando o paciente deita e as imagens são adquiridas. No caso da ciclomaca ou bicicleta ergométrica, assim como no estresse farmacológico, as imagens são obtidas continuamente durante os diversos estágios do esforço. Com isso, a administração de ARUS difere de acordo com a modalidade de estresse adotada.

Sabemos que alguns pacientes não possuem janela ecocardiográfica satisfatória, o que pode comprometer a qualidade e interpretação do exame. Nesses casos, o uso desses agentes, que permitem a visualização perfeita dos bordos das paredes do coração, se tornam imprescindíveis. A visualização segmentar detalhada é fundamental para a detecção de DAC. A visualização de todos os segmentos em repouso e durante o estresse é importante para determinar a extensão da isquemia induzida ou a presença de infarto prévio.^219,220^

Atualmente, a tendência é não chamar esses agentes de "contraste", para que não sejam associados àqueles usados em radiologia ou na ressonância magnética, com potencial tóxico e alérgico infinitamente maiores do que o "contraste" para ultrassom.^221^ Por isso, esta diretriz adota a sigla "ARUS".

A ecocardiografia de estresse com ARUS apresenta maior sensibilidade, especificidade e acurácia diagnóstica.^222,223^

Além disso, o exame com ARUS pode ter melhor relação custo-benefício quando utilizado com indicação, especialmente quando dois ou mais segmentos não são bem visualizados ao corte apical,^223^ e assim evitar exames mais onerosos e com potencial de toxicidade renal ou uso de radiação.

### 21.1. Agente de Realce Ultrassonográfico

Atualmente, o único ARUS registrado no Brasil é o Sonovue (Bracco), cuja composição é de microbolhas a partir de gás de enxofre. Na sua grande maioria, essas bolhas são menores do que 5 mícrons ou micrômetros e atravessam os capilares pulmonares, chegando rapidamente (cerca de 2 segundos) ao lado esquerdo do coração e realçando os bordos das paredes do VE. Esses agentes melhoram a imagem e a confiança na interpretação do teste.^219-222^

A literatura que corrobora a segurança dos ARUS é enorme, e reações anafilactoides, apesar de muito raras, demandam a necessidade de ter medicações e materiais de ressuscitação cardiopulmonar prontamente acessíveis.^223^

#### 21.1.1. Forma de Administração

A maioria dos equipamentos modernos apresenta *presets* específicos para o uso dos ARUS na análise da borda endocárdica e opacificação da cavidade ventricular (e não para análise da perfusão). E embora o ARUS possa ser administrado na forma de bolus ou de infusão contínua, avalia-se que a forma em bolus é mais prática e de excelente resultado. A administração do ARUS deve ser o suficiente para opacificar o coração de forma homogênea, sem causar redemoinho no ápice ou atenuação na base do coração. Deve-se trabalhar com os protocolos de fábrica do aparelho que apresentam o IM baixo (< 0,3), com harmônica ou com sequência de pulso não linear.^224,225^ Em equipamentos mais modernos, um IM muito baixo (< 0,2) evita a destruição das microbolhas, realça o sinal das microbolhas e reduz os artefatos, permitindo uma excelente delimitação dos bordos endocárdicos.

Alguns serviços usam o ARUS em todos os exames de ecocardiografia de estresse como maneira de melhorar a acurácia do teste. Contudo, a relação custo-efetividade é questionável nesses casos, uma vez que a necessidade real de muitos centros parece girar em torno de 20% dos casos.

Durante a ecocardiografia de estresse farmacológico associada à ARUS, utilizam-se presets de estresse com contraste para o delineamento da borda endocárdica. Quando se deseja avaliar a perfusão (que depende de um software específico e mais sofisticado, que não é o mesmo para a análise da borda endocárdica), em vez do *preset* de análise das bordas endocárdicas, utiliza-se o *preset* adequado para perfusão miocárdica (com softwares do tipo *pulse inversion*, *power modulation* ou *cadence*). Vale considerar que a análise da perfusão miocárdica requer conhecimento especializado e uma curva de aprendizado maior que a análise da borda endocárdica.

Para aplicação do ARUS, utiliza-se um equipo com duas vias ou, preferivelmente, uma torneirinha no acesso periférico obtido com cateter tipo abocath de bom calibre, e deixa-se uma das vias em linha reta para a administração do ARUS. O ARUS é administrado antes do início do teste (imagens basais) e antes/durante cada etapa do protocolo de estresse para a obtenção de imagens comparáveis. Na primeira injeção, utiliza-se um *bolus* de aproximadamente 0,5 mL, e pode ou não ser administrado um *flush* de soro fisiológico (solução salina) lentamente após (a administração rápida do ARUS e/ou do soro ocasiona hipersinal com sombra nas regiões médio-distais do coração). Durante as etapas com infusão da dobutamina, não se utiliza mais o *flush* de solução salina após.

Durante a ecocardiografia de esforço em esteira, dá-se preferência à colocação de um acesso no membro superior esquerdo (preferência no antebraço) do paciente e a administração é realizada cerca de 15-20 segundos antes do pico de esforço e antes do paciente ser conduzido à maca de exame para aquisição das imagens ecocardiográficas no pós-esforço imediato. Também não há necessidade do *flush* após a administração do ARUS nesse caso. As imagens basais são obtidas assim que o paciente se deita na maca de exame.

**Figura 21.1:**
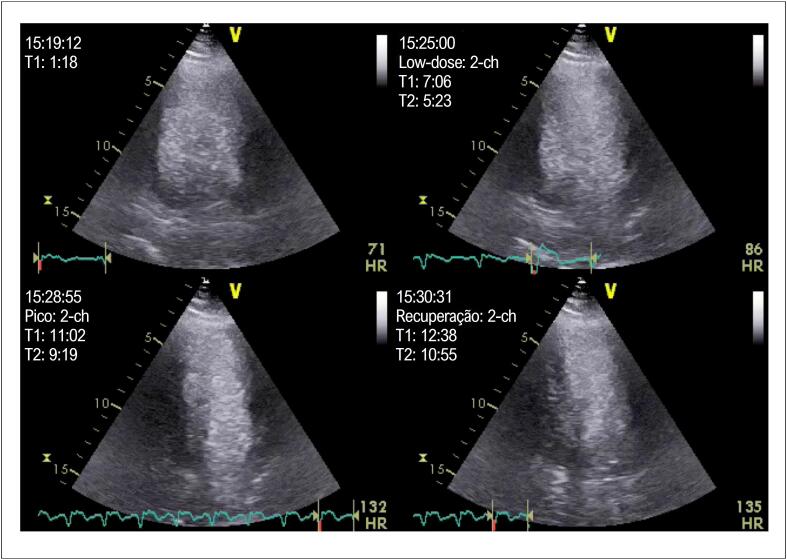
As imagens lado a lado em quad-screen facilitam a interpretação do teste nas diferentes fases do exame.

Por meio do software de estresse com *quad-screen*, o aparelho de ecocardiografia coloca as imagens cardíacas lado a lado, facilitando a interpretação do teste. Idealmente, o ARUS deve ser usado em todas as etapas do estresse (Figura 21.1).

Vale reforçar que quando o ARUS é utilizado junto à ecocardiografia de estresse, o ideal é iniciar a obtenção das imagens pela janela apical. Esta pode ser a única usada para a obtenção de todas as paredes do VE, pois o ápice do VE deve estar bem opacificado. Ele é o que mais sofre com a destruição das bolhas pela insonação e, por isso, deve ser avaliado primariamente.

## 22. Ecocardiografia de Estresse com Agente de Realce Ultrassonográfico na Análise da Perfusão Miocárdica

O volume de sangue na circulação coronariana se encontra majoritariamente (cerca de 90 %) no interior dos capilares. Da mesma forma que os ARUS, quando interagem com o ultrassom, opacificam a cavidade ventricular esquerda, as microbolhas dentro dos capilares conferem um aspecto cintilante intramiocárdico. Assim, a intensidade desse brilho está correlacionada com o volume de sangue dentro da microvascularização.

Após a administração do ARUS, a perfusão miocárdica (PM) é avaliada nas três incidências ecocardiográficas apicais do VE. Isso pode ser realizado tanto em infusão contínua do agente ou, mais rotineiramente, em bolus.

Em caso de análise concomitante de contração e perfusão miocárdica, utiliza-se a técnica de destruição/reperfusão (repreenchimento) das microbolhas, usando IM muito baixo, especialmente quando a análise é realizada com o software específico para análise da perfusão em tempo real (IM < 0,2), atualmente considerada ideal para essa análise (e não o software padrão para análise da borda endocárdica). Essa técnica é importante para definir a velocidade de repreenchimento da parede miocárdica e a presença ou não de isquemia.

**Figura 22.1:**
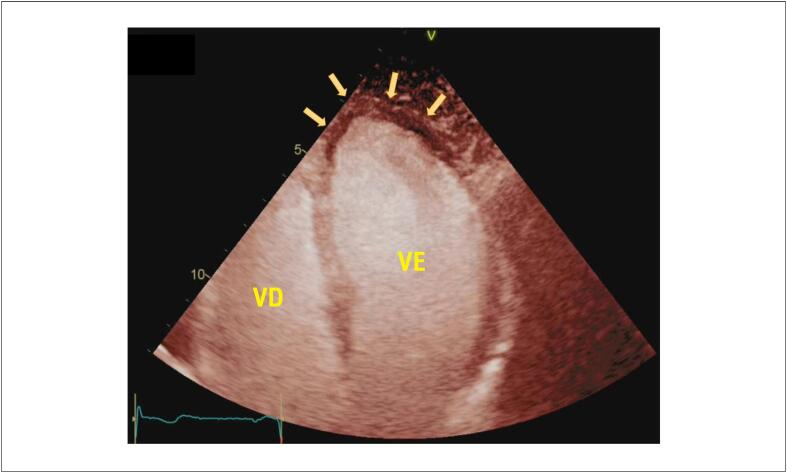
Mulher de 60 anos submetida a ecocardiografia com agente de realce ultrassonográfico sob estresse farmacológico pela dobutamina. Apresentou desconforto torácico e infradesnivelamento do segmento ST em derivações precordiais no traçado eletrocardiográfico. Observa-se área com déficit de perfusão miocárdica no ápice do ventrículo esquerdo associada a hipocinesia local (setas). Teste positivo para presença de isquemia miocárdica apical.

Após a administração do ARUS, assim que uma opacificação adequada do VE é atingida, é disparado um pulso de alta energia (flash; alto IM: 0,8 – 1,2)^226^ que destrói as microbolhas no volume sanguíneo capilar intramiocárdico, preservando relativamente as microbolhas intracavitárias. Havendo obstrução limitante do fluxo coronário nas artérias epicárdicas e/ou na microvascularização, o resultado final observado é um brilho miocárdico mais atenuado ou até ausente (zona enegrecida) do segmento afetado (Figura 22.1).

**Figura 22.2:**
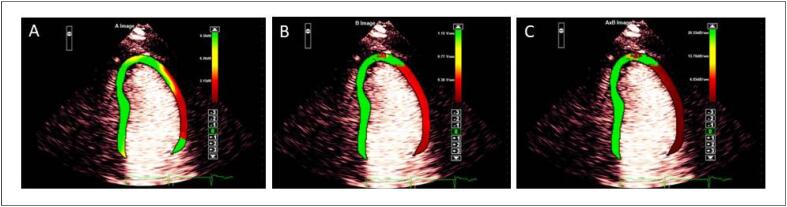
Exemplo de análise off-line da perfusão miocárdica. Janela apical de duas câmaras do VE. Presença de área extensa de déficit de perfusão miocárdica na parede anterior (em vermelho). A, análise do volume de sangue capilar (componente α). B, análise da velocidade sanguínea capilar (componente β). C, análise do fluxo de sangue da microvascularização (α × β).

Essa análise é realizada rotineiramente durante o exame. Contudo, a análise off-line se mostrou superior pelo fato de possibilitar uma análise objetiva dos dois componentes da PM: o volume de sangue capilar (componente α) e a velocidade sanguínea capilar (componente β). A PM seria o fluxo de sangue da microvascularização determinado pelo produto desses dois componentes (α × β), conforme demonstrado na Figura 22.2. A redução do componente α pode estar relacionada a áreas infartadas ou restrição significativa do fluxo microvascular, enquanto a diminuição do componente β está correlacionada com a redução do fluxo capilar associada a DAC obstrutiva ou MINOCA. No MINOCA, há um retardo com déficit da PM, mas com espessamento miocárdico preservado, dada a ausência de comprometimento subendocárdico.^227^ Esse diagnóstico é realizado pela análise da perfusão-contração, na qual se avalia em conjunto a PM e motilidade segmentar miocárdica, como também pela demonstração de uma reserva de fluxo da microvascularização < 2, preferencialmente realizada na ecocardiografia de estresse com vasodilatador.^228,229^ O componente β é superior ao α em termos de sensibilidade para detecção de DAC.^230^ Em geral, uma PM normal se baseia no repreenchimento miocárdico (reestabelecimento total do sinal medido em decibéis) em menos de 5 segundos no repouso (para FC de 60 bpm) ou até 2 segundos durante o estresse (para FC de 120 bpm). Essa análise deve ser realizada no final da sístole.^230^

A PM é um grande auxílio à avaliação convencional subjetiva da motilidade e espessamento segmentar miocárdico, base da avaliação da ecocardiografia sob estresse, aumentando a sensibilidade do método para detectar a DAC, conforme demonstrado por diversos estudos.^231-233^ O método também se mostrou superior em sensibilidade para detectar DAC obstrutiva em relação à cintilografia com SPECT.^234^ Um dos motivos é justamente o fato de a cintilografia detectar o volume sanguíneo capilar,^235^ enquanto a PM pelo ecocardiograma possibilita avaliar, também, a velocidade do fluxo sanguíneo capilar, que sabe-se ser um marcador mais sensível para a detecção de DAC.^230^

Estudos de acurácia para detecção de DAC demonstram que a ecocardiografia de estresse associada à análise de PM tem sensibilidade de 83% e especificidade 79% quando se utiliza um vasodilatador;^236,237^ quando se utiliza dobutamina ou estresse físico, tem sensibilidade e especificidade de 88% e 77%, respectivamente.^237^

Além de aumentar a detecção de DAC, a ecocardiografia com análise da PM é um bom indicador prognóstico. Um exame sob estresse adequado e negativo para a presença de isquemia miocárdica associado à análise de PM normal tem excelente evolução, com incidência muito baixa de eventos cardiovasculares graves e óbito no curto a médio prazo.^238^ Há também uma chance maior de remodelamento ventricular ao longo do tempo, sendo a PM uma variável superior a dados clínicos e FEVE para essa predição.^239^

A PM também pode auxiliar na avaliação da viabilidade miocárdica. Histologicamente, a base da viabilidade do tecido miocárdico, que mantém possibilidade de recuperação funcional, está na relação entre a densidade da microvascularização e a quantidade/extensão de colágeno (fibrose) tecidual. Sendo assim, áreas extensas com intenso déficit de PM denotam transmuralidade e menor viabilidade miocárdica, o que é comparável à análise da RMC com utilização de gadolínio.^240,241^

Recomendação:

- Nos serviços com equipe habilitada, a incorporação da ecocardiografia contrastada com análise de PM nos exames sob estresse agrega o poder de detecção do método para identificar e estratificar DAC obstrutiva;
- A PM pode auxiliar na análise da viabilidade miocárdica de segmentos que claramente não melhoram a contração com o teste com dobutamina.

## 23. Ecocardiografia de Estresse com *Strain* Miocárdico na Cardiopatia Isquêmica

A introdução da técnica de ecocardiografia com *speckle-tracking* (STE) permite a avaliação quantitativa da deformação ou tensão miocárdica durante a ecocardiografia de estresse. A imagem de tensão fornece uma nova abordagem para a avaliação da função miocárdica que é objetiva e quantitativa e tem diversas vantagens sobre a ecocardiografia tradicional. O objetivo desta diretriz é fornecer uma visão geral do estado atual do conhecimento sobre a utilidade do GLS ou SLG (*strain* longitudinal global) e do RLS (*strain* regional) na ecocardiografia de estresse, com foco especial na doença cardíaca isquêmica.

### 23.1. *Strain* Derivado do *Speckle Tracking* Versus *Strain* Baseado no Doppler Tecidual Durante Ecocardiografia de Estresse

Como mencionado, a ecocardiografia de estresse é uma ferramenta utilizada para avaliação da DAC, induzindo estresse fisiológico ao coração por meio de exercício ou estimulação farmacológica com dobutamina ou dipiridamol. A imagem abrangente, que avalia a deformação miocárdica, melhora a precisão da ecocardiografia de estresse no diagnóstico e prognóstico da DAC.

A imagem de Doppler tecidual e suas derivadas, como a deformação (*strain*) e taxa de deformação miocárdica (*strain rate*), fornecem abordagens alternativas para quantificar com mais precisão a contração regional em repouso ou durante o estresse.^49,242^ O Doppler tecidual é viável durante testes de estresse, mas requer uma alta taxa de quadros (*frame rate*), de pelo menos 140 quadros por segundo. No entanto, sua principal limitação é que as variáveis de pico de velocidade e deformação dependem do ângulo entre o feixe de ultrassom incidente e a parede miocárdica. Portanto, a região apical não pode ser avaliada^243^ pela análise Doppler.

A imagem de deformação ou *strain* 2D que não envolve Doppler é uma nova técnica ecocardiográfica usada para medir a deformação e a taxa de deformação. Ela traça pontos na imagem de ultrassom para realizar uma análise bidimensional do movimento.^244^

Os aplicativos de software disponíveis atualmente permitem o processamento espacial e temporal de imagens, bem como o reconhecimento e a seleção de tais pontos (as impressões digitais do coração) na imagem ecocardiográfica. O deslocamento de cada grupo de pontos (pixels) representa o movimento local do tecido. Ao rastrear essas manchas, é possível calcular a velocidade, a deformação e a taxa de deformação do tecido pelo ultrassom bidimensional (2D). A imagem de *strain* 2D sem Doppler é um procedimento simples, que requer apenas a aquisição de um ciclo cardíaco e pode ser facilmente processado e interpretado após a aquisição dos dados.^245^

A isquemia é definida como uma redução regional no espessamento miocárdico, que também pode resultar em atraso no início e no término da contração sistólica.

Observe que a isquemia não é um fenômeno apenas espacial, mas também temporal. Experimentalmente, com uma oclusão coronária de 40 segundos, o espessamento não apenas diminui, mas é retardado e continua no período pós-ejeção ou diastólico (tardocinese, contração pós-sistólica ou deformação pós-sistólica).

Infelizmente, o olho humano não possui resolução temporal suficiente para detectar alterações contráteis sutis em tempo real. Em compensação, a deformação é rastreada automaticamente durante o ciclo cardíaco, medindo a distância entre dois pixels de um segmento miocárdico. Numerosas regiões pequenas e agrupadas (kernels) são sondadas e o programa calcula a média dos seus movimentos antes de extrair curvas regionais e a média global. A qualidade do traçado pode ser verificada para cada segmento, com ajuste manual da região se necessário. Esse novo método permite a avaliação simultânea dos três componentes da deformação miocárdica: radial, longitudinal e circunferencial. Na prática, as incidências apicais são usadas para obter o SLG e a taxa de deformação, enquanto as incidências paraesternais de eixo curto são usadas para avaliar a deformação radial e circunferencial.^246^

Hanekom et al. realizaram um estudo envolvendo 150 pacientes para comparar o uso do *strain* 2D e do Doppler tecidual para detecção de isquemia durante ecocardiografia de estresse com dobutamina. O estudo descobriu que a medição do *strain* durante a ecocardiografia de estresse com dobutamina usando imagens de STE é viável e tem precisão semelhante à do Doppler tecidual na detecção de isquemia no território da ADA esquerda. No entanto, a deformação 2D não foi tão precisa quanto o Doppler tecidual na detecção de isquemia nos territórios da artéria circunflexa e artéria coronária direita.^247^

Na realidade, não há competição entre o *strain* baseado em Doppler e o *strain* 2D por STE. As vantagens e limitações de cada técnica se complementam. O *strain* Doppler deve ser usado quando as frequências cardíacas estão altas, com janelas de qualidade inferior, para avaliar a velocidade basal e medial, e o *strain* 2D quando priorizamos a precisão e análise apical do VE. Recomenda-se o uso do *strain* 2D com frequências cardíacas entre 40 e 100 bpm, com janelas de boa qualidade, para avaliação longitudinal e circunferencial do VE, além da rotação e torção, e priorizar a automaticidade e o menor tempo de estudo.

### 23.2. Ecocardiografia de Estresse com *Strain* 2D: Valor Diagnóstico e Prognóstico na Doença Arterial Coronariana

Reant et al. investigaram a aplicabilidade do strain 2D para detecção de isquemia durante ecocardiografia de estresse com dobutamina em suínos. Os principais resultados do estudo demonstraram que o *strain* 2D é tão confiável quanto a sonomicrometria para avaliação da função regional do VE. Os autores demonstraram que anormalidades longitudinais e circunferenciais precedem a diminuição da deformação radial na presença de isquemia. Essa observação pode ser explicada pelo fato de as fibras miocárdicas subendocárdicas terem orientação principalmente longitudinal, o que torna a função longitudinal mais sensível à isquemia e, portanto, alterada mais precocemente que a função radial.^248^

Elamragyy et al. realizaram um estudo prospectivo envolvendo 101 pacientes com probabilidade de risco intermediário de DAC. Os pacientes foram avaliados por EED e imagens de STE. Todos os pacientes foram submetidos a angiografia coronária dentro de 1 mês. A sensibilidade e a especificidade da EED para detecção de DAC foram de 79,6% e 92,3%, respectivamente. Ao combinar o valor de corte de SLG ≤ −20,5, a sensibilidade e a especificidade para o diagnóstico de DAC aumentaram para 95,9% e 84,6%, respectivamente.^249^

Outros autores também demonstraram que SLG durante a EED tem maior sensibilidade, mas menor especificidade em comparação com a análise da alteração da contratilidade parietal.

O estudo de Uusitalo et al. avaliou o *strain* e o índice pós-sistólico em 50 pacientes com risco intermediário para DAC após infusão de dobutamina durante a fase de recuperação inicial. Os resultados mostraram um aumento na precisão diagnóstica do teste. Em pacientes com DAC, observou-se que o *strain* foi de −16,4 ± 4,8, comparado com −19,3 ± 3,9 em indivíduos normais (p < 0,001) com aumento significativo no índice pós-sistólico.^250^

Os resultados do estudo de Uusitalo et al. sugerem que a incorporação de medições de tensão e índice pós-sistólico durante a recuperação precoce após a infusão de 20 mcg/kg/min de dobutamina pode melhorar a precisão diagnóstica do EED em pacientes com risco intermediário para DAC.

Embora os estudos de viabilidade sejam controversos, uma grande literatura sustenta que o comportamento do SLG e do SPI é capaz de reconhecer o miocárdio viável de acordo com a resposta a baixas doses de dobutamina.

Imagens de *strain* durante a EED são mais fáceis de obter em comparação com a ecocardiografia de exercício porque são menos afetadas pelo movimento respiratório. A EED também permite uma aquisição de imagem mais consistente, devido ao aumento estável da FC e da contratilidade, o que pode melhorar a precisão e a confiabilidade das medidas de deformação miocárdica.

Imagens de *strain* durante a EED têm sido estudadas extensivamente, mas faltam resultados sobre a viabilidade e a aplicabilidade da imagem durante a ecocardiografia com exercício. Isso pode se dever a dificuldades técnicas associadas à aquisição de imagens durante o exercício, bem como ao aumento do movimento do paciente e artefatos respiratórios que podem afetar a precisão das medidas de esforço.

Atualmente, existem poucos dados e apenas pequenas séries que comparam a aquisição de imagens convencionais com o uso de GLS e RLS durante ecocardiografia de estresse em esteira ou bicicleta supina.

A viabilidade da ecocardiografia de estresse com exercício foi avaliada em 67 pacientes adultos saudáveis, com resultados satisfatórios. O estudo analisou pacientes por faixa etária e sexo, com grupos divididos nas faixas etárias de 23 a 35, 35 a 55 e 55 a 80 anos. Os resultados mostraram que o SLG ou GLS aumentou significativamente durante o exercício em todas as faixas etárias, independentemente do sexo. O aumento absoluto médio na deformação longitudinal global foi de 5,3%.^251^ Isso sugere que a ecocardiografia de esforço pode ser uma ferramenta útil para avaliar a função cardíaca durante o teste de esforço.

Atualmente, é possível demonstrar, em pacientes selecionados, com boa janela, a alta viabilidade para análise do SLG em repouso (99%) e no pós-estresse (esforço) imediato, antes dos 30 segundos (97,5%) em bicicleta supina. A ausência de aumento ou diminuição da deformação longitudinal dos segmentos apicais foi concordante com a presença de isquemia detectada visualmente em 78,6% dos estudos, enquanto em 96,2% dos casos em que a isquemia foi negativa pelos critérios de anormalidade parietal subjetiva pela avaliação padrão, a deformação longitudinal apical aumentou.

No entanto, o exame somente era confiável para segmentos relacionados ao território da ADA. Assim, a análise do *strain* 2D foi viável no período pós-esforço imediato, mas as altas frequências cardíacas foram responsáveis pelos resultados inespecíficos nos segmentos medial e basal ínfero-póstero-lateral.^252^

As principais razões pelas quais apenas os segmentos apicais puderam ser analisados foram, possivelmente: 1) A resolução axial 2D é geralmente muito boa. 2) A resolução lateral diminui à medida que a profundidade de campo aumenta. Se a resolução lateral é baixa, a consequência é uma imagem "borrada", que não permite que os pixels sejam seguidos tão facilmente nos campos distantes. 3) Com os movimentos translacionais do coração durante o exercício, a dificuldade de rastreamento dos segmentos basal e medial aumenta. 4) Com a FC elevada, as mudanças de quadro a quadro são abruptas e a independência angular começa a ser perdida com uma densidade de linha (e de informação) menor, suficiente para rastreamento no campo próximo, mas não para o campo distante.^252^

Entre os exames disponíveis, a ecocardiografia de estresse com dipiridamol (EEDIP) é uma das técnicas recomendadas para o diagnóstico e prognóstico da DAC. A sua utilização em diferentes laboratórios não é generalizada por falta de conhecimento ou por razões pessoais, geográficas, econômicas ou de disponibilidade, e não por motivos científicos de fato. Sua utilização de acordo com o estado de arte da técnica, em altas doses e curto tempo de infusão com adição de atropina e/ou *handgrip,* permite um nível de acurácia diagnóstica semelhante à da EED, com taxa de complicações graves três vezes menor.^57,253^

No estudo retrospectivo de Lowenstein-Haber et al. envolvendo 150 pacientes encaminhados para EEDIP, foram avaliadas a reserva coronariana (RC) apical e da ADA e análise visual da motilidade parietal.^254^ *Strain* regional apical anormal durante ecocardiografia com dipiridamol, bem como baixa RC, foram os únicos preditores de pior resultado na progressão, independentemente da análise visual da anormalidade parietal.

Um relatório anterior do mesmo grupo também demonstrou que a análise de deformação 2D melhorou significativamente a sensibilidade da EEDIP para a detecção de DAC em relação à análise de contratilidade padrão (83,3% vs. 50%; p = 0,001), sem comprometer sua especificidade (100%).^255^

Os estudos de outros grupos que também comprovaram o valor aditivo da medição de deformação bidimensional à análise visual tradicional do escore parietal permitem conclusões idênticas.

### 23.3. Qual *Strain* Pode Ser Útil na Ecocardiografia de Estresse: Global ou Regional, Longitudinal ou Circunferencial? E Qual a Importância do Espessamento Pós-Sistólico?

A maioria dos estudos que avaliaram a viabilidade e a acurácia do SLG durante a ecocardiografia de exercício inclui pequenos grupos de indivíduos saudáveis.

Embora a análise RLS possa fornecer informações mais detalhadas sobre áreas específicas do coração, esta também pode ser tecnicamente mais difícil e complicada de realizar e interpretar. Por outro lado, o SLG facilita a avaliação mais completa da função miocárdica global e tem se demonstrado um preditor poderoso de resultados cardiovasculares adversos.

**Figura 23.1:**
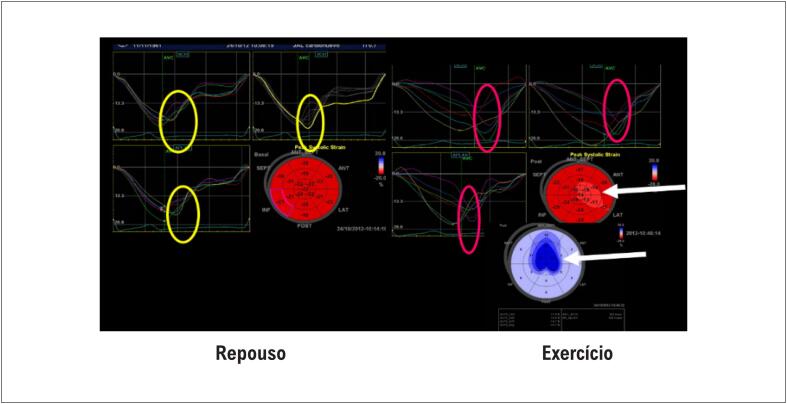
Homem de 50 anos com fatores de risco positivos e dor interescapular no início do exercício. Diminuição da tensão e da deformação pós-sistólica no pico do exercício (setas brancas). Angiografia coronária: duas obstruções significativas em conjunto na artéria descendente anterior.

No entanto, também é importante notar que a mera observação da amplitude da deformação pode não refletir toda a pesquisa. O momento do pico de deformação, tanto global quanto regionalmente (porcentagens de contração e deformação pós-sistólica e índices de dispersão mecânica), são fundamentais e podem fornecer informações sobre anormalidades na mecânica miocárdica, como visto no exemplo a seguir (Figuras 23.1 e 23.2).

**Figura 23.2:**
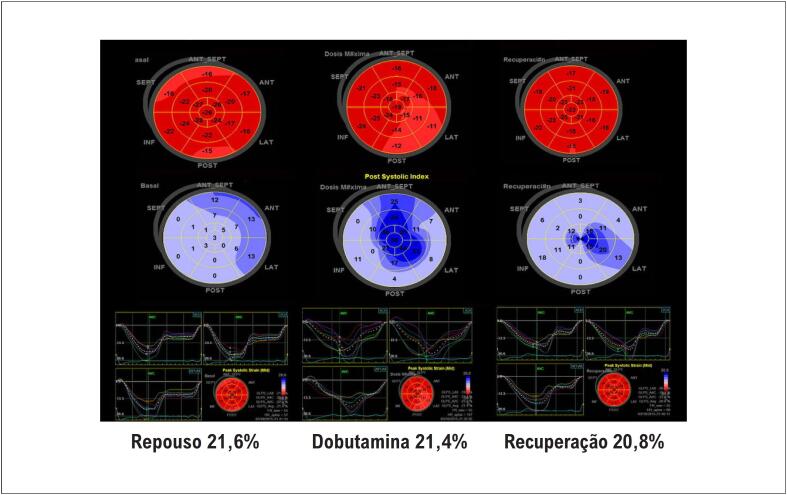
Strain global com pouco aumento com dobutamina, mas dispersão acentuada das curvas e contração pós-sistólica como sinal de isquemia. Canto superior esquerdo: mapa polar com strain basal de 21,6%. Médio: Strain à ecocardiografia de esforço com dobutamina 21,4%. Direita: Strain na recuperação 20,8%. Linha do meio: Em azul, a porcentagem de strain pós-sistólico que aumentou acentuadamente com baixas doses de dobutamina. Abaixo das respectivas curvas. Paciente com doença arterial coronariana grave triarterial.

A ecocardiografia de estresse com dipiridamol é uma excelente combinação para avaliar simultaneamente a movimentação da parede e o comportamento do SLG e do SPI, acrescentando informações diagnósticas e prognósticas. GLS e RLS com dobutamina aumentam a precisão para detectar a viabilidade e o *strain rate*, enquanto Doppler (modo M) pode ser de grande ajuda para visualizar o fenômeno da contração pós-sistólica, que também pode ser visualizado nas curvas e no alvo com *strain* 2D, mostrando dissincronismo/isquemia caso apareça durante o teste.

O estabelecimento de valores normais para o *strain* no estresse em indivíduos saudáveis pode ajudar a interpretar melhor os dados de *strain* em pacientes com suspeita de doença cardiovascular.^256^

O SLG no VE não é homogêneo e há um gradiente dos segmentos basais ao ápice. Conhecido como gradiente basal-apical, é observado tanto no *strain* circunferencial quanto no longitudinal, com menores valores nos segmentos basais e maiores valores no ápice. Também foi encontrado gradiente entre o subendocárdio e o epicárdio.

Embora a literatura forneça valores de referência para os segmentos apical, médio-ventricular e basal, a falta de homogeneidade da deformação regional complica as análises posteriores. Contudo, a análise da deformação regional pode ajudar a confirmar a isquemia na ecocardiografia de estresse.

Três cortes de eixo curto nos níveis basal, médio-ventricular e apical devem ser usados para avaliar a deformação circunferencial e o twist e torção. Porém, a aquisição do ápice idêntico à imagem basal em repouso nem sempre é viável durante a ecocardiografia de estresse, tornando-a menos adequada para esse tipo de avaliação.

### 23.4. O *Strain* Tem Memória Isquêmica?

Em um estudo com 101 pacientes com comportamento isquêmico segundo análise visual, o SLG médio em repouso foi de −22,3 ± 4,3%; diminuindo significativamente no pico do exercício para −16 ± 3,2%; 3 minutos após a recuperação, os pacientes apresentaram valores superiores aos obtidos em repouso, −24,3 ± 5,1%. Com isso, as alterações do SLG e do SPI não persistiram após a recuperação dos distúrbios de motilidade segmentar analisados visualmente.^257^

Esse comportamento é consistente com um estudo experimental da Universidade de Osaka que comprovou que a recuperação da deformação e da taxa de deformação foi rápida e completa (em relação ao tempo de oclusão). No entanto, os índices pós-sistólicos foram mais duradouros.

**Figura 23.3:**
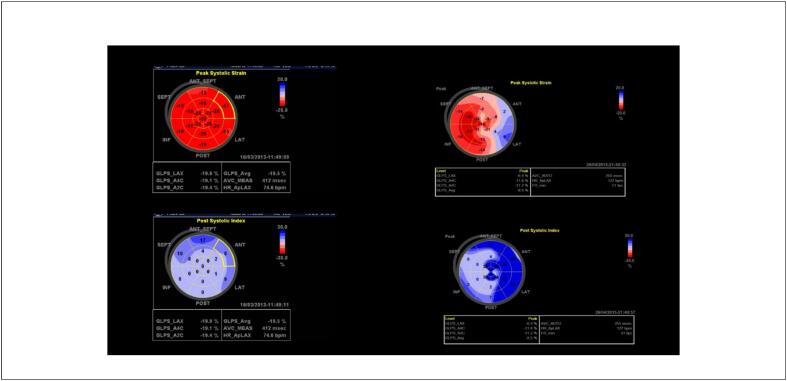
Mulher de 57 anos com angina estável. Strain longitudinal global normal em repouso com dissinergias múltiplas e extensas em baixa carga, deterioração do pico de strain longitudinal e deformação pós-sistólica significativa: Angiografia coronária: Obstrução grave distal do tronco principal esquerdo: Artéria descendente anterior, Artérias coronárias direita e circunflexa com obstruções graves. Strain longitudinal global em repouso: 19,5% Exercício: 9,5%.

A conclusão é que se a análise do SLG e do SPI for realizada vários minutos após o exercício, com frequências inferiores a 100 bpm, quando não há mais distúrbios de contratilidade e o tempo de isquemia foi curto, a distensão se recupera rapidamente, mesmo em valores supernormais (Figuras 23.3 e 23.4).

**Figura 23.4:**
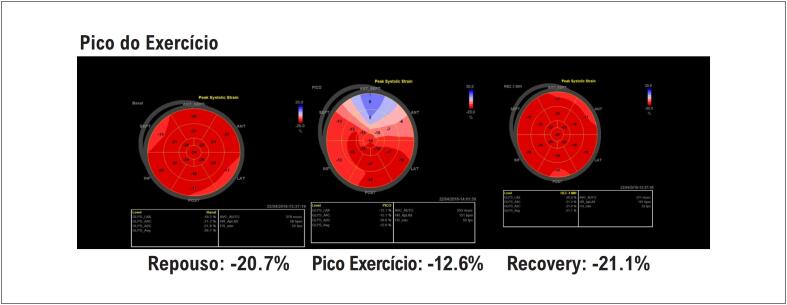
Paciente de 65 anos. Ecocardiografia de estresse positivo com isquemia nos segmentos apical, septal anterior, anterior e inferior com strain longitudinal global no início −20,7%, pico −12,6% e após 3 minutos de recuperação −21,1%. Angiografia coronária: Obstruções graves das artérias coronárias direita e descendente anterior. Exemplo de que o esforço em repouso não prevê o resultado da ecocardiografia de estresse e não possui memória isquêmica.

Esse comportamento transitório explica por que pacientes com angina e sem distúrbios de motilidade apresentam um estiramento 2D em repouso na faixa normal fora do episódio de dor (se de curta duração).

### 23.5. O *Strain* Longitudinal Global Basal Pode Predizer Doença Arterial Coronariana na Ausência de Anormalidade Parietal Visual sob Estresse?

Há relatos de que o SLG em repouso é preditor da presença de doença coronariana do tronco principal esquerdo e de doença triarterial em pacientes sem anormalidades na motilidade regional. De acordo com o estudo de Jin-Oh Choi et al., o *strain* 2D global foi menor em 108 pacientes com doença triarterial ou do tronco principal esquerdo, mesmo sem anormalidades de contratilidade (com ponto de corte de −17,9%) com sensibilidade e especificidade de 79% em relação ao normal.^258^

O estudo de Montgomery relata que a análise do SLG pode detectar DAC moderada e grave comparável ao resultado da análise visual durante a ecocardiografia de estresse. Os autores afirmam que o SLG inferior a −20% tem valor preditivo negativo de 80% para lesões > 50% e de 90% para lesões > 70%.^255^

Por outro lado, em um grupo de 124 pacientes sem distúrbios contráteis em repouso submetidos a cineangiocoronariografia, o SLG não permitiu prever o resultado da ecocardiografia de estresse nem a presença de DAC significativa.^260^

### 23.6. Limitações

Embora a imagem de *strain* 2D durante a ecocardiografia de estresse tenha demonstrado seu benefício na detecção de alterações sutis na função ventricular esquerda, seu uso apresenta diversas limitações. Fatores técnicos como qualidade de imagem, movimento respiratório e posição do paciente podem afetar a precisão e a reprodutibilidade das medidas de *strain*. Além disso, atualmente não existe um método padronizado para análise de *strain* na ecocardiografia de estresse, o que pode limitar a comparabilidade e aumentar a variabilidade dos resultados entre os diferentes estudos.

Embora a experiência tenha mostrado resultados promissores, estudos maiores são necessários para estabelecer a acurácia diagnóstica, a sensibilidade e a especificidade da imagem com *strain* na detecção de isquemia miocárdica durante o exercício.

Ademais, a integração das medidas de *strain* somadas a outros parâmetros obtidos durante a ecocardiografia de estresse, como a análise visual de anormalidades parietais, linhas B, reserva diastólica, reserva contrátil e reserva cronotrópica, que é a verdadeira arte da ecocardiografia de estresse, não é realizada na maioria dos laboratórios do mundo, e sua utilidade está confirmada para diferentes doenças cardíacas, como demonstram os resultados do estudo Stress 2030 liderado pelo Dr. Eugenio Picano et al.^261^

É conveniente ter em conta que o resultado dessas novas técnicas também depende do operador e que são necessárias tecnologia, experiência e formação adequadas para a sua utilização racional. Com experiência, tecnologia adequada e excelente qualidade de imagem, essa limitação não é tão crítica para a ecocardiografia de estresse.

A FC elevada dificulta a análise do estiramento 2D no exercício e com altas doses de dobutamina, pois a resolução temporal tem dificuldade de rastrear os pixels dos segmentos basal e médio do miocárdio.

Embora a imagem de *strain* tenha mostrado benefícios em potencial na ecocardiografia de estresse, incluindo maior precisão na detecção de isquemia miocárdica e identificação de disfunção ventricular esquerda subclínica em pacientes assintomáticos, ainda será necessário realizar um trabalho de pesquisa considerável para definir completamente seu papel na prática clínica de rotina.

## 24. Ecocardiografia de Estresse com *Strain* Miocárdico para Além da Cardiopatia Isquêmica

Uma das aplicações do SLG durante a ecocardiografia de estresse é a determinação da reserva contrátil (RC), que complementa as informações fornecidas pela variação da FE. A resposta normal é o incremento de dois pontos absolutos do SLG do VE durante o estresse em comparação com o repouso.^262^

Na regurgitação mitral primária moderada ou mais grave sem disfunção ventricular, Magne et al. demonstraram que a ausência de RC por SLG à ecocardiografia de estresse de exercício é um preditor importante e independente de eventos cardíacos futuros.^128^

Um estudo interessante de Arbucci et al. demonstrou que a ausência de RC por SLG durante a ecocardiografia de estresse de exercício, em pacientes assintomáticos com estenose aórtica importante, está associada a uma maior taxa de eventos e menor sobrevida. Além disso, a avaliação de RC por SLG foi o melhor preditor de eventos em comparação com a medida tradicional, a avaliação de RC por FE.^263^

Na estenose aórtica de baixo fluxo e baixo gradiente com FE reduzida, a avaliação de SLG de repouso e durante a ecocardiografia de estresse com dobutamina adiciona informações prognósticas importantes aos parâmetros ecocardiográficos tradicionais. Conforme demonstrado por Dahou et al., SLG inferior a 9% em repouso e inferior a 10% ao estresse está associado a maior mortalidade tanto em pacientes tratados conservadoramente quanto àqueles submetidos a intervenções.^264^

A diferenciação entre cardiomiopatia isquêmica e não isquêmica na presença de disfunção ventricular importante é um desafio para a ecocardiografia de estresse. Muitas vezes, a avaliação anatômica é imprescindível. No entanto, em pacientes com disfunção leve ou moderada do VE, o incremento sustentado da contratilidade e do SLG sugere a presença de cardiomiopatia não isquêmica.^99^ Vale ressaltar que essa resposta também é observada em atletas durante a ecocardiografia de estresse, em geral com incremento supranormal da deformação, sendo de grande valor na distinção entre o coração do atleta e cardiomiopatias.^265,266^

Na cardiomiopatia hipertrófica, o uso do SLG durante a ecocardiografia de estresse auxilia na identificação de pacientes sob maior risco cardiovascular. Além da ausência de RC, a presença de dissincronia ao exercício também é um parâmetro relevante a ser analisado nessa população.^267^

A maior dificuldade do uso do SLG durante a ecocardiografia de estresse é a diferenciação entre a redução patológica da deformação e a redução secundária devido às limitações inerentes à técnica. Taquicardia, hiperventilação, imagem inadequada, variabilidade de resultados entre os aplicativos de software e inexperiência com o método são alguns dos desafios para o emprego do *strain* no estresse (Tabela 24.1), como mencionado anteriormente.

**Tabela 24.1:** Lista de limitações do uso do strain longitudinal global durante a ecocardiografia de estresse e as possíveis soluções a serem adotadas

Problemas	Soluções
Tempo adicional à ecocardiografia de estresse/necessidade de aquisição de imagens adicionais	Utilizar de rotina os três cortes apicais no protocolo de estresse
Imagem inadequada	Ajuste adequado da profundidade, largura do setor e taxa de quadros (> 60 qps) Evitar a aquisição de clipes com variabilidade de FC superior a 10% Verificar a qualidade do ECG e evitar a aquisição de clipes extrassistólicos/pós-extrassistólicos
Frequência cardíaca elevada	Utilizar imagens de recuperação imediata, dentro do primeiro minuto pós esforço, na ecocardiografia de estresse físico.

ECG: eletrocardiograma; FC: frequência cardíaca.

Na doença não isquêmica, valoriza-se mais o valor global do que o valor regional da deformação, o que facilita seu emprego na prática. A influência negativa da taquicardia e da hiperventilação na avaliação da deformação durante a EEF pode ser reduzida com a utilização de imagens de recuperação imediata (até o primeiro minuto após o término do exercício). O estresse com dipiridamol, por sua menor influência cronotrópica e alta qualidade de imagem bidimensional, facilita consideravelmente a avaliação do SLG.

O uso do *strain* se tornou frequente na prática clínica e deve ser adotado sempre que possível. O caráter quantitativo, independente e objetivo o transformam em uma ferramenta promissora e que deve ser incorporada à ecocardiografia de estresse.

Atualmente, a utilidade do SLG do VE para avaliação da RC se tornou importante. Independentemente da patologia estudada, a ausência de RC está associada a pior evolução clínica e maior taxa de eventos. A factibilidade da avaliação da RC com SLG durante a ecocardiografia de estresse é maior em estudos com vasodilatador, seguido da dobutamina e, por fim, do exercício.

## 25. Ecocardiografia de Estresse Tridimensional

### 25.1. A Tecnologia da Ecocardiografia Tridimensional e sua Aplicação na Imagem do Ecocardiograma

A ecocardiografia tridimensional permite a visualização em tempo real de cinco dimensões físicas das estruturas cardíacas, considerando-se os três planos ortogonais de definição anatômica, o plano dos fluxos cardíacos e o plano do dimensionamento temporal.^268-271^ Dessa forma, a técnica acrescenta informações morfofuncionais importantes em comparação com as informações derivadas da análise ecocardiográfica bidimensional.^268-271^ As primeiras imagens relacionadas à ultrassonografia tridimensional (3D) datam da década de 50, em que o interesse estava direcionado à observação da órbita humana. Na década de 70, foram realizadas as primeiras imagens das estruturas cardíacas. A partir de então, ocorreram diversos incrementos e avanços na observação ecocardiográfica cardíaca tridimensional^48,272-281^ até chegarmos ao momento atual, em que é possível realizar uma análise matricial multiangular tridimensional do coração em tempo real. Isso foi possível graças à nanotecnologia, à observação digital estrutural e ao avanço indiscutível da velocidade de análise morfofuncional com o desenvolvimento de algoritmos dedicados a identificação matricial e dos elementos volumétricos de imagem (voxels). A análise simultânea em tempo real de múltiplos planos de observação estrutural do coração se encaixa perfeitamente na ecocardiografia de estresse, possibilitando a realização de exames em menor de tempo de aquisição, com grande reprodutibilidade, pequena variação interobservador, proporcionando ainda a identificação estrutural a partir de novas projeções espaciais.^48,272-281^ A aquisição tridimensional também permite a redução do encurtamento espacial das imagens (*foreshortening*) durante a sua aquisição, situação que pode ocorrer durante o emprego da ecocardiografia bidimensional. Atualmente, a ecocardiografia de estresse 3D pode ser realizada a partir de imagem biplanar guiada por observação tridimensional (*full-volume*) ou a partir de imagem matricial (*X-plane*). A análise ecocardiográfica de estresse 3D depende sobretudo da qualidade da imagem inicial, sendo amplificada com a associação de ARUS. Contudo, o emprego de ARUS pode ocasionar uma redução da resolução temporal, que já apresenta limitação ao 3D, apesar da melhora considerável da definição das bordas endocárdicas em estudos de estresse 3D com imagem em harmônica e qualidade subótima.^278^ Por esse motivo, seu uso junto ao estresse 3D ainda não é amplamente recomendado. Observamos que a aquisição em imagem bidimensional permite a análise com maior número de quadros (maior *frame rate*) e a análise da perfusão miocárdica (quando utilizada com ARUS) também utiliza o *frame rate*, porém menor, enquanto a análise 3D propicia a avaliação a partir de maior volume de amostra (*3D Full-volume*), ou de análise biplanar em tempo real (*3D-X-plane*). No entanto, a análise em *3D Full-volume* pode redundar em artefatos de aquisição (*stitch*) por movimento de translação dos voxels e apresenta baixas taxas de repetição dos voxels e baixa resolução temporal e espacial (*3D Full-volume*), o que limita a análise da perfusão miocárdica. Em comparação, análise em 3D matricial (*X-plane*) apresenta melhor taxa de repetição dos quadros.

Novos modelos de equipamentos de ecocardiografia estão sendo disponibilizados para a prática clínica, com a possibilidade de usar transdutores e algoritmos que permitem melhor qualidade da imagem, com *volume rate* tridimensional mais elevado, ampliando a sua realização futura.

Como modelos de realização do estresse 3D, podemos empregar a análise multiplanar 3D com a observação simultânea do eixo longo longitudinal e do eixo curto do VE e de dados volumétricos das projeções apicais. A imagem para aquisição em 3D apical *Full-Volume* deve ser realizada com o transdutor em posição apical (ápex do VE), com o paciente em apneia durante a aquisição (para a minimização do *stitching* eventual das imagens, derivado da translação cardíaca), com o volume de amostragem largo o suficiente para a análise de todo o VE, porém sem que ocorra excesso de abertura do setor (que redunda em menor volume rate), e com o registro do ECG. Caso ocorram arritmias, como a fibrilação atrial, isso pode ocasionar erros na análise 3D. A hiperventilação durante a aquisição da imagem também pode representar dificuldades à obtenção da melhor qualidade de imagem.

Para o futuro, a ecocardiografia de estresse 3D estará relacionada ao emprego de novos algoritmos de interpretação de imagem e de transdutores menores, com a aquisição das imagens a partir de um batimento cardíaco único, com maior número de voxels. Isso levará a melhor qualidade das imagens, sobretudo da qualidade da imagem lateral, o que por sua vez levará à redução do fenômeno de *stitching* e ao menor tempo de realização do exame.

### 25.2. A Aplicação da Ecocardiografia Tridimensional Junto à Ecocardiografia de Estresse

A escolha da aplicação de imagens tridimensionais em tempo real para o exame de ecocardiografia de estresse é evidente quando se analisa as vantagens na captura de imagens e interpretação dos fenômenos isquêmicos e alterações da geometria ventricular.

Peteiro et al. compararam o desempenho da qualidade tridimensional com a da bidimensional para isquemia induzida ao esforço,^282^ bem como Yang et al.,^274^ que usaram o protocolo com dobutamina. Esses estudos iniciais demonstraram sensibilidade e especificidade semelhantes aos protocolos padrões com imagem bidimensional, com as vantagens de facilidade e menor tempo de aquisição da imagem.

Em um estudo com 44 pacientes submetidos a angiografia, Badano et al.^275^ demonstraram sensibilidade e especificidade da técnica tridimensional comparáveis às da bidimensional em aparelhos com taxas de quadros mais elevadas, bem como a vantagem do método tridimensional para a análise de alterações apicais relacionadas ao território da ADA. Os estudiosos apontam a ecocardiografia tridimensional como equivalente à bidimensional, mas com vantagens em alguns aspectos relevantes.

Usamos a facilidade dos aparelhos tridimensionais na ecocardiografia de estresse.^283^ A primeira vantagem está em usar o método de três planos simultâneos, chamado Tri Plano, que oferece os cortes de quatro câmaras apical, duas câmaras apicais e eixo longo apical. Infelizmente, essa modalidade não é oferecida por todos os fabricantes de aparelhos tridimensionais. O ajuste do aparelho permite que, após a obtenção de uma boa janela quatro câmaras, automaticamente se enxergue as outras janelas com boa taxa de quadros, normalmente acima de 30 quadros por segundo, com frequência alcançando 60 quadros por segundo. Obter apenas uma janela para observar o quadro completo pela técnica 3D diminui o tempo de obtenção do clipe para um terço, agilizando a execução da ecocardiografia de esforço.

**Figura 25.1:**
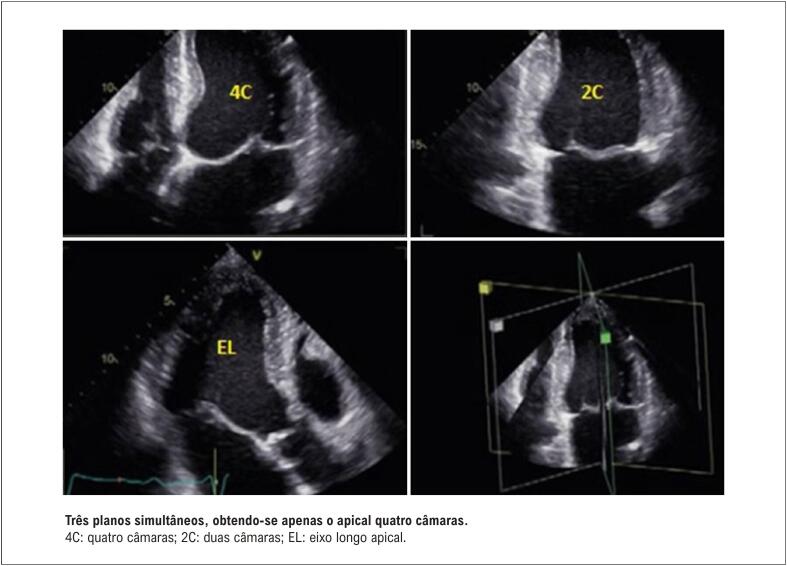
Janela apical única, permitindo a análise de todas as paredes do ventrículo esquerdo, poupando tempo e o esforço do examinador.

Em exames realizados de acordo com o protocolo de Balke (exercício), é possível capturar imagens a qualquer momento do exercício ou após a interrupção, normalmente usando o repouso, baixa carga de esforço (elevação de 20% na frequência de repouso), pico do esforço (FC submáxima ou 85% da FC máxima prevista para a idade) e recuperação. Apenas uma janela apical e focada no corte quatro câmaras perfaz cada estágio da ecocardiografia de esforço e reduz o esforço repetido para o membro superior do examinador, que é poupado quando evita-se a rotação para obter os dois cortes apicais subsequentes padrão (Figura 25.1).

O corte *multislice*, que oferece múltiplos cortes transversais do ventrículo, normalmente não é envolvido, mas pode ser usado para casos duvidosos em imagem adquirida em 3D previamente. Com a técnica *multislice*, é possível "cortar" o VE transversal e milimetricamente e observar a análise segmentar de modo mais detalhado e minucioso, comparando a contração do VE em cada etapa da ecocardiografia de esforço.

Alguns serviços utilizam o protocolo que combina a aquisição bidimensional triplano com imagens tridimensionais em tempo real, usando o protocolo de captura e análise do primeiro e analisando imagens do segundo, em separado.^277^ O método é utilizado também e principalmente no ensino da técnica para iniciantes. Lidando com os dois formatos, o examinador pode escolher a imagem com maior representatividade para as alterações provocadas pelo estresse físico ou farmacológico. Esse processo pode ajudar na adaptação ao modo tridimensional, com sua menor taxa de quadros, mas melhor interpretação de alterações volumétricas.

**Figura 25.2:**
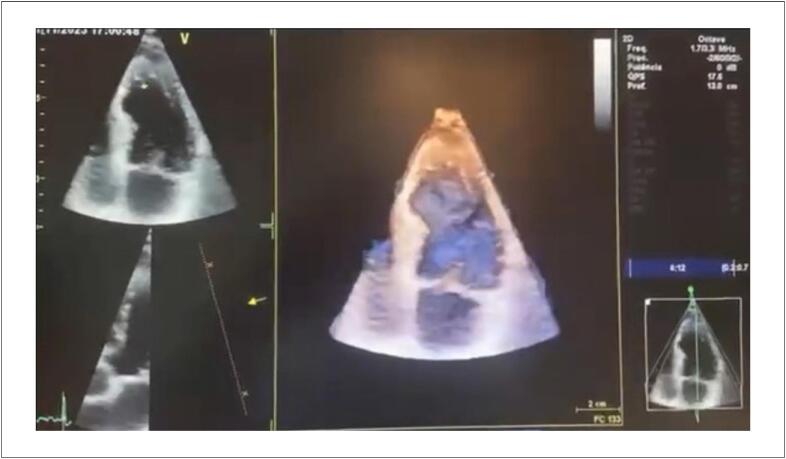
Imagem tridimensional à ecocardiografia de estresse.

A obtenção de imagens tridimensionais precisa de ajuste no aparelho para permitir a obtenção de melhor taxa de volumes por segundo. Trabalhando com a menor profundidade e abertura possíveis, é possível obter taxas acima de 24 volumes por segundo. A possibilidade de rotação dos eixos do VE para análise mais focada no ápice ou na base, bem como o giro para análise da contração nas laterais da imagem, permite uma interpretação da contração superior àquela obtida na aquisição convencional (Figura 25.2).

Dada a capacidade de adquirir todo o volume do VE em um único batimento, a ecocardiografia tridimensional em tempo real oferece uma promessa substancial para se tornar o padrão de referência na avaliação de pacientes com suspeita de DAC.

A maior produtividade com menor tempo para o exame e maior conforto para o executor do exame são vantagens significativas. A execução e a análise do exame tridimensional exigem uma adaptação do serviço.

Em centros com alto volume de ecocardiografia de estresse, a aquisição de aparelhos capazes de realizar o exame de estresse tridimensional pode ser importante para otimizar a dinâmica do serviço.

## 26. A Ecocardiografia de Estresse na Sala de Emergência: Indicações e Aplicações

A dor torácica é uma das principais queixas em salas de emergências hospitalares. Nos EUA, a queixa de dor torácica representa 7 milhões de atendimentos anuais, sendo 30% destes categorizados como SCA. Estima-se que até 4% dos pacientes são liberados da sala de emergência erroneamente em decorrência de apresentação atípica ou ECG não diagnóstico.^284^ Em uma época de custos crescentes na saúde, uma internação hospitalar de múltiplos dias para "descartar" a SCA não é mais sustentável, e a condição requer protocolos e fluxos de avaliação eficazes.

Para os pacientes nos extremos do espectro de risco coronariano, a triagem é fácil, e aqueles com SCA definida são encaminhados diretamente para o cateterismo cardíaco, enquanto aqueles com probabilidade muito baixa de isquemia coronariana são liberados. O maior desafio se concentra nos pacientes com risco cardíaco baixo a intermediário e sem evidências claras de diagnóstico de SCA, seja por ECG não diagnóstico, seja por enzimas cardíacas de fase aguda negativas. Nesse grupo não desprezível de pacientes, o dilema diagnóstico pode ser caro e, muitas vezes, frustrante.

### 26.1. Utilidade da Ecocardiografia de Estresse na Emergência

A ecocardiografia na sala de emergência permite avaliar a função cardíaca em repouso e detectar diagnósticos diferenciais importantes que causam dor torácica aguda, como dissecção de aorta e embolia pulmonar, reduzindo sensivelmente o tempo de avaliação. A utilização da modalidade de estresse, tanto farmacológico quanto físico, dependendo das características do paciente, disponibiliza de forma rápida e eficaz uma ferramenta importante para estratificação de risco não invasiva.

Uma das principais limitações do método é a qualidade das imagens, que pode ser afetada por fatores como obesidade, doença pulmonar subjacente ou dificuldades técnicas. Para superar essa barreira, como nos casos em que a janela é desfavorável e em que mais de dois segmentos não são visualizados adequadamente, é possível utilizar o ARUS para melhorar a acurácia do exame.

Em 2007, foram publicados os resultados do estudo ASSENCE (Avaliação de Custo-Efetividade de Diversas Estratégias de Diagnóstico Precoce em Pacientes com Dor Torácica Aguda e Eletrocardiograma Não Conclusivo). Trata-se de um ensaio clínico randomizado de 10 centros que comparou a relação custo-efetividade da EED e atropina precoce em comparação com avaliação padrão e ergometria. Foram incluídos pacientes com ECG não diagnóstico de SCA, com biomarcadores iniciais negativos para lesão miocárdica e capacidade de realizar teste de esforço (ergometria). O principal objetivo do estudo foi a relação custo-efetividade, levando em consideração o tempo de hospitalização durante a admissão inicial e as hospitalizações repetidas, procedimentos diagnósticos hospitalares e ambulatoriais e tratamentos. Os desfechos secundários incluíram frequência de eventos cardiovasculares adversos maiores (MACE) precoces e tardios, reinternação e revascularização coronariana. Entre os 110 pacientes no braço da ecocardiografia de estresse, 90 (82%) receberam alta direta, em comparação com 78 dos 89 (88%) no braço do ECG de esforço. Para esses pacientes, os custos gerais em 2 meses foram 39% menores no braço da ecocardiografia de estresse em comparação com o braço do teste ergométrico (US$ 1.029 vs. US$ 1.684; p=0,005). Nos pacientes que necessitaram de internação, os custos não diferiram significativamente entre as estratégias diagnósticas. Assim, o estudo ASSENCE confirmou a tendência geral de que a adição de exames de imagem às estratégias de diagnóstico sem imagem dos cuidados padrões é custo-efetiva. A origem dessa economia foi atribuída a menos eventos tardios (nenhum no braço da ecocardiografia vs. 11% no braço da ergometria), incluindo reinternações, SCA e intervenção percutânea tardia após ECG de estresse negativo.^285^

No mesmo ano, Jeetley et al.^286^ apresentaram um ensaio clínico randomizado de eficácia clínica, de centro único, em que 433 pacientes tiveram avaliações iniciais negativas para SCA e foram randomizados para ecocardiografia de estresse ou ergometria. O desfecho primário foi a identificação correta dos pacientes que apresentavam alto risco de um desfecho combinado de morte cardíaca, infarto do miocárdio não fatal ou revascularização coronariana. O desfecho secundário foi o custo para o diagnóstico. O risco pré-teste foi classificado de acordo com o escore de risco TIMI (*Thrombolysis In Myocardial Infarction*). O risco pós-teste foi considerado baixo se os resultados do teste eram negativos e alto se eram positivos, independentemente do risco pré-teste. Um número significativamente maior de pacientes com ecocardiografia de estresse do que pacientes com ergometria foram classificados como de baixo risco pós-teste e, portanto, elegíveis para alta imediata (77% vs. 33%; p < 0,001). Além disso, um ecocardiograma de estresse positivo para alto risco foi mais preciso do que a ergometria na previsão desse desfecho primário (51% vs. 29%; p = 0,01). A taxa de coronariografias normais no grupo de ergometria positiva foi quase duas vezes maior que no grupo de ecocardiografia de estresse (39% vs. 23%). Significativamente mais pacientes com ergometria do que pacientes com ecocardiografia de estresse necessitaram de mais testes diagnósticos durante o período de acompanhamento (47% vs. 20%; p < 0,001). O custo médio para o diagnóstico foi significativamente menor no grupo da ecocardiografia de estresse do que no grupo da ergometria (£ 326 vs. £ 495; p = 0,01), evidenciando os "benefícios clínicos e de custo-efetividade" significativos da ecocardiografia de estresse em relação à ergometria em pacientes com suspeita de SCA.^286^

Em 2021, uma força-tarefa liderada pela AHA, ACC e ASE publicou uma diretriz de avaliação e diagnóstico de dor torácica, na qual se estabeleceu que pacientes com dor torácica aguda e de baixo risco não necessitam de exames adicionais de imagem anatômicos ou funcionais e podem ser liberados para avaliação ambulatorial.^287^ A ecocardiografia transtorácica (ETT), como foi comentado, pode visualizar e auxiliar no diagnóstico diferencial entre as inúmeras causas de dor torácica aguda, como dissecção aórtica aguda, derrame pericárdico, cardiomiopatia de estresse ou catecolinérgica (síndrome de Takotsubo) e CMH, entre outras. A visualização da função ventricular esquerda e direita e as anormalidades da contratilidade segmentar permitem a avaliação do risco de DAC e podem ajudar na decisão clínica. Após a exclusão de SCA, em pacientes de risco intermediário, a ecocardiografia de estresse pode ser utilizada para definir a presença e a gravidade da cardiopatia isquêmica e para fins de estratificação de risco. Também para pacientes de risco intermediário, com angiotomografia inconclusiva, a ecocardiografia de estresse pode ser uma alternativa diagnóstica.^287^

**Figura 26.1:**
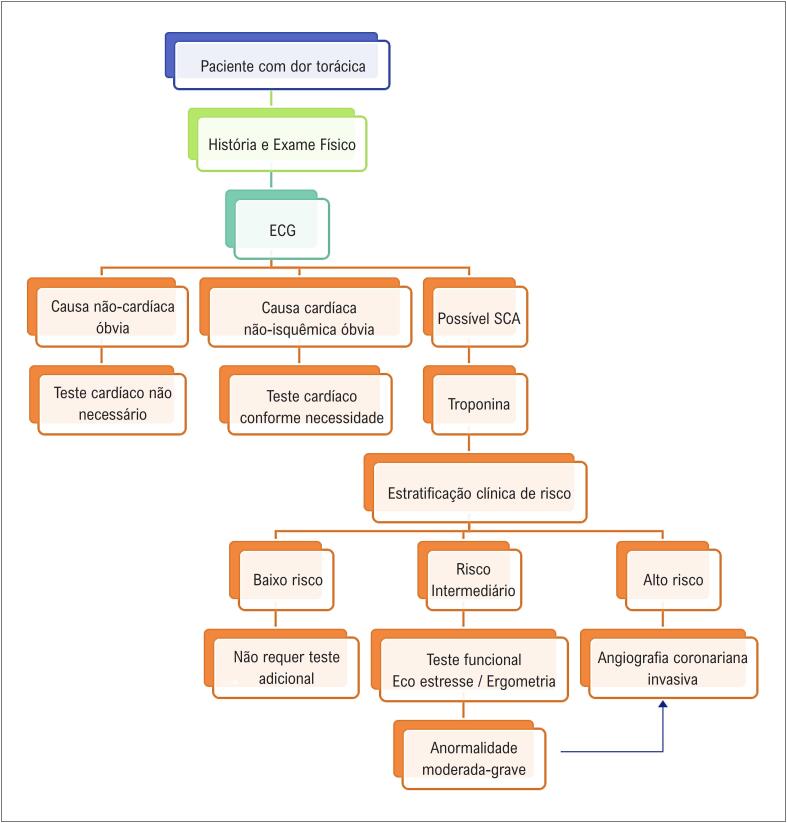
Organograma adaptado da diretriz de manejo de síndrome coronariana aguda da Sociedade Europeia de Cardiologia (2023).^288^

Mais recentemente, em 2023, foram publicadas as diretrizes para manejo da SCA da Sociedade Europeia de Cardiologia. Nelas, a ecocardiografia de estresse também foi colocada como ferramenta importante para a triagem em pacientes sem elevação ou com elevação incerta de biomarcadores e ECG não diagnóstico, com nível de evidência A e indicação classe IIA^288^ (Figura 26.1).

Assim, a ecocardiografia de estresse na sala de emergência é uma ferramenta valiosa no diagnóstico e manejo de pacientes com suspeita de SCA, principalmente naqueles com risco intermediário, sem ECG diagnóstico e sem alterações enzimáticas. No entanto, é importante considerar suas limitações e garantir que os médicos estejam treinados na técnica. Importante também é que a logística do serviço esteja preparada para realizar o exame em curto espaço de tempo, preferencialmente nas primeiras 24 horas, garantido a custo-efetividade do método conforme observado nos diferentes ensaios clínicos.

## 27. Ecocardiografia de Estresse no Pós-Transplante Cardíaco

A ecocardiografia de estresse é uma técnica diagnóstica que revela isquemia miocárdica induzível em pacientes transplantados cardíacos. O desenvolvimento de doença coronariana aterosclerótica, conhecida como vasculopatia do enxerto, é a principal causa de falência do enxerto e morte durante o primeiro ano de transplante cardíaco.

A angiografia coronária por cateterismo cardíaco tem sido, ao longo dos anos, usada rotineiramente para avaliar a DAC do enxerto, mas é um procedimento invasivo e apresenta maior risco de morbidade em pacientes transplantados cardíacos.

A ecocardiografia de estresse é capaz de identificar doença vascular do enxerto e tem valor prognóstico reconhecido na área. Assim, um ecocardiograma de estresse normal, sem alterações na contratilidade segmentar, justifica o adiamento de estudos invasivos.^289^

Durante um estudo, devido à denervação cirúrgica do transplante cardíaco, alguns pacientes atingiram sua FC ideal com baixas doses de dobutamina. Isso ocorre porque os pacientes transplantados cardíacos apresentam resposta cronotrópica aumentada à estimulação beta-adrenérgica com esse medicamento. Em outros casos, é necessária a adição de atropina, apesar da dose máxima de dobutamina, o que em geral acontece em casos de receptores idosos.^290^

A dobutamina é o agente estressor mais utilizado, com sensibilidade entre 70 e 80% para a detecção de vasculopatia do enxerto.^291^ Devido à baixa resposta da FC ao exercício causada pelo estado de desnervação, os protocolos de exercício apresentam uma sensibilidade muito limitada, de 15 a 33%.^292^

Um estudo de Bacal et al.^293^ com ecocardiografia de estresse positivo mostrou que este foi um preditor independente de eventos cardíacos e/ou morte em um seguimento de 4 anos. Por outro lado, um ecocardiograma de estresse negativo com dobutamina indicou baixo risco para doença do enxerto coronariano e baixa taxa de incidência de efeitos cardíacos adversos.^294^

A EED é a modalidade mais recomendada para vigilância rotineira da vasculopatia do enxerto durante os primeiros 5 anos após o transplante cardíaco devido à alta prevalência da doença no pós-transplante cardíaco.^295^

## 28. Ecocardiografia de Estresse em Subgrupos Especiais (Bloqueio de Ramo Esquerdo, Bloqueio do Ramo Direito e Fibrilação Atrial)

### 28.1. Bloqueio de Ramo Esquerdo

O BRE é uma condição frequente, de etiologia variada e desafiadora do ponto de vista diagnóstico. O BRE afeta a contração miocárdica, o enchimento diastólico do VE e a perfusão coronariana em graus variáveis de acordo com gravidade do bloqueio e da patologia cardíaca subadjacente.^296^

Aproximadamente 2% dos pacientes encaminhados para teste ergométrico apresentam BRE fixo ou intermitente.^297^ A prevalência de DAC em pacientes com BRE varia entre 30 e 50%.^298^ Na presença de BRE, o ECG não é interpretável para avaliação de isquemia, sendo necessário a realização de uma técnica de imagem para a investigação não invasiva de DAC.

A ativação elétrica alterada interfere no movimento da parede septal, que pode variar desde um movimento normal até um movimento paradoxal. Na presença de um complexo QRS com duração < 150 ms, frequentemente o espessamento parietal é normal e a capacidade de contração está preservada. Já o padrão de movimentação paradoxal da parede septal é encontrado com mais frequência quando a duração do complexo QRS ultrapassa os 150 ms e/ou na presença de fibrose septal.

Apesar da maior dificuldade na interpretação da motilidade septal devido ao movimento anormal da parede, a ecocardiografia de estresse é a melhor opção para o diagnóstico de DAC nesse cenário.^44,298^ É uma modalidade mais específica do que a cintilografia do miocárdio,^44,299^ com boa sensibilidade nesses casos, embora essa sensibilidade seja menor para avaliação do território da ADA esquerda na presença de discinesia/dissincronia septal vista à ecocardiografia basal.^298^

O valor prognóstico da ecocardiografia de estresse nos pacientes com BRE é excelente, especialmente em pacientes sem infarto do miocárdio prévio. Cortigiani et al. avaliaram o valor preditivo prognóstico da ecocardiografia de estresse farmacológico em pacientes com BRE. Nesse estudo, duas variáveis ecocardiográficas estavam associadas a um risco elevado de morte por causa cardíaca: o WMSI em repouso e a variação do WMSI no pico do estresse. A sobrevida em cinco anos foi de 77% no grupo de pacientes com teste positivo para isquemia miocárdica e de 92% no grupo com teste normal (p = 0,02).^300^

O valor diagnóstico e prognóstico da ecocardiografia de estresse pode ser melhorado pela adição da avaliação simultânea da reserva de velocidade do fluxo coronariano na ADA esquerda^301^ ou da perfusão miocárdica com ARUS.^302^

### 28.2. Bloqueio do Ramo Direito

O bloqueio do ramo direito (BRD) é encontrado com frequência na população geral saudável, mas pode ser causado por condições que afetam o ramo direito do feixe de condução. As causas possíveis incluem trauma, alterações estruturais, doenças infiltrativas e infecciosas, miocardite e DAC.

O BRD está presente em aproximadamente 2% a 3% dos pacientes com DAC.^296,297^ Nesse cenário, sua presença é um preditor independente de mortalidade por qualquer causa.^303^ Em especial, a ocorrência de BRD no contexto da SCA está significativamente associada com o aumento na mortalidade intra-hospitalar, mesmo após intervenção coronariana percutânea primária.^304^ A ecocardiografia de estresse é uma excelente modalidade diagnóstica para a investigação de DAC, uma vez que o BRD normalmente não afeta o movimento regional da parede. Além disso, ela fornece uma estratificação prognóstica eficiente e aditiva aos parâmetros simples da eletrocardiografia em repouso, como o bloqueio fascicular anterior esquerdo. Em pacientes com suspeita de DAC submetidos a ecocardiografia de estresse, o BRD associado ao bloqueio divisional anterossuperior do ramo esquerdo se mostrou um preditor independente de mortalidade.^305^

### 28.3. Fibrilação Atrial

A prevalência de FA é muito baixa entre os jovens (< 1% em pessoas com menos de 40 anos), mas aumenta com a idade, atingindo até 17% dos indivíduos com mais de 80 anos.^306^ A DAC é uma das condições cardiovasculares mais comumente associadas à FA.

O encurtamento da fase diastólica na FA pode gerar resultados falso-positivos durante o teste ergométrico, uma vez que a perfusão subendocárdica está comprometida. Nesse contexto, a ecocardiografia de estresse é uma modalidade eficaz. Em geral, o estresse com vasodilatadores é melhor tolerado do que o estresse com dobutamina. Entretanto, apesar da resposta cronotrópica aumentada à infusão de dobutamina em pacientes com FA comparados aos pacientes em ritmo sinusal, a EED apresenta bom valor diagnóstico nesses pacientes. Hobday et al.^307^ demonstraram que um percentual maior dos pacientes com FA atingiram a FC alvo e precisaram de menor dose de atropina em comparação com os pacientes em ritmo sinusal. Nesse mesmo estudo, resultados positivos para isquemia miocárdica ocorreram de forma semelhante entre os pacientes com FA e ritmo sinusal, e a utilização de betabloqueadores foi similar.^307^ A atropina, por outro lado, deve ser usada com parcimônia em pacientes portadores de FA, pelo risco de elevar demais a FC, ocasionando risco de internação hospitalar.

O aparecimento de FA durante a ecocardiografia de estresse é uma complicação mais comum em pacientes com substrato arritmogênico, quando o agente estressor utilizado é a dobutamina. Em uma revisão publicada com base em 26 estudos sobre as complicações associadas à EED, a incidência de FA foi de 0,9%.^308^ Em um estudo recente que avaliou retrospectivamente 4.917 pacientes consecutivos submetidos a EED, foram identificados como principais fatores relacionados ao desenvolvimento de FA durante o exame, a história prévia de FA paroxística (p = 0,02) e a idade avançada, com incidência de 4% nos pacientes com mais de 80 anos (p < 0,0001).^309^ Na maioria dos pacientes, o retorno para o ritmo sinusal ocorre durante a primeira hora após o início da arritmia,^310^ geralmente sendo necessário a administração de betabloqueador tipo metoprolol.

## 29. Ecocardiografia de Estresse no Pré-Operatório de Cirurgia Vascular, em Mulheres e Idosos

### 29.1. Pré-Operatório de Cirurgia Vascular

Pacientes submetidos a cirurgias vasculares apresentam risco elevado de eventos cardiovasculares durante o perioperatório. Além das características clínicas dos pacientes, isso também pode ser atribuído à coexistência da DAC.^311,312^ Além disso, a natureza e a complexidade das intervenções vasculares desempenham papel relevante nesse cenário.^313^

Assim, a estratificação pré-operatória é de extrema importância e tem como objetivo identificar pacientes mais suscetíveis a eventos cardiovasculares, possibilitando implementação de medidas que reduzirão a morbimortalidade. Nesse contexto, a ecocardiografia de estresse (físico ou farmacológico) se destaca como um método não invasivo, amplamente disponível e de baixo custo.

A EEF oferece benefícios na avaliação da capacidade funcional e na resposta hemodinâmica ao esforço. No entanto, a presença de doença vascular periférica e a baixa capacidade funcional podem impossibilitar a eficácia do teste, sendo, portanto, recomendado o estresse farmacológico com dobutamina ou dipiridamol nesse subgrupo de pacientes. A escolha do agente farmacológico deve ser pautada pela disponibilidade, experiência de cada centro, mecanismo de ação dos medicamentos e doenças vasculares preexistentes.

A EED tem se mostrado útil e segura, podendo ser empregada inclusive em pacientes com aneurisma de aorta abdominal.^314^ O teste com agentes vasodilatadores (adenosina ou dipiridamol) também pode ser considerado uma alternativa viável e com boa acurácia,^312^ cabendo ressaltar que o seu uso é contraindicado na presença de doença carotídea grave bilateral.^313^

Pacientes que apresentam ecocardiograma de estresse positivo têm maior incidência de eventos cardiovasculares. Os exames sob estresse farmacológico demonstram excelente valor preditivo negativo, variando entre 90 e 100%, enquanto o valor preditivo positivo é mais baixo, entre 25 e 45%.^312^ Os fatores preditores mais significativos de eventos adversos no pós-operatório são a presença de isquemia e história de insuficiência cardíaca congestiva. Na EED, a FC na qual a isquemia se desenvolve pode ser utilizada para estratificar os pacientes em risco baixo, intermediário e alto para infarto e óbito no pós-operatório.^315,316^

A ocorrência de novas alterações da motilidade parietal induzidas pelo estresse, extensa (três ou mais segmentos) ou limitada (um ou dois segmentos), associada com história de infarto prévio, tem sido caracterizada como fatores independentes para a ocorrência de eventos cardíacos tardios.^317^

Em uma meta-análise que englobou seis testes não invasivos na avaliação pré-operatória de cirurgias vasculares, os testes de estresse farmacológico apresentaram sensibilidade e especificidade global mais elevadas, sendo que a EED demonstrou tendência para desempenho diagnóstico superior aos outros exames.^318^

Em múltiplos estudos e meta-análises, o valor prognóstico da EED foi, no mínimo, tão eficaz quanto o da cintilografia do miocárdio em prever a ocorrência de eventos adversos no pós-operatório.^3,311,319^

### 29.2. Mulheres

A doença cardiovascular (DCV) é a principal causa de morte em mulheres em todo o mundo. O diagnóstico e o manejo apropriados permanecem um desafio, uma vez que pacientes do sexo feminino podem apresentar sintomas atípicos e, portanto, receber tratamento inadequado.^320^

Inúmeros relatos mostram que as mulheres têm maior probabilidade de desenvolver angina pectoris como primeiro sintoma de DAC (47% vs. 32%) e são menos propensas a apresentar infarto agudo do miocárdio (6% vs. 10%) em comparação com os homens.^321^

A ecocardiografia de estresse é uma ferramenta bem estabelecida e de grande utilidade na investigação diagnóstica de mulheres com suspeita de DAC, bem como de estratificação de risco daquelas com a doença diagnosticada. Ela demonstra sensibilidade, especificidade e valor prognóstico sem diferenças específicas entre os sexos.^322^ Além disso, a acurácia diagnóstica da EED tem se mostrado similar tanto entre mulheres com dor atípica quanto em casos angina.^323,234^

Em estudo realizado por Biagini et al.,^325^ foram incluídos 2.276 homens e 1.105 mulheres com DAC suspeita ou conhecida. Eles foram submetidos a EED e a estratificação de risco foi feita após seguimento de 7 anos, observando-se os desfechos de infarto do miocárdio não fatal e morte cardiovascular. Os resultados mostraram que a EED fornece informações prognósticas independentes tanto em homens quanto em mulheres.

Do ponto de vista técnico, é importante destacar que as mulheres apresentam maior incidência de hipotensão arterial durante a EED. Cavidades ventriculares pequenas com maior grau de hipertrofia relativa podem facilitar o surgimento de obstrução dinâmica na VSVE, levando à hipotensão e à interrupção prematura do teste. Além disso, em corações menores, há maior probabilidade de obliteração da cavidade ventricular esquerda, o que pode mascarar alterações sutis na motilidade parietal.^326^ Também pode ocorrer dor torácica na vigência de obstrução dinâmica da VSVE, gerando dúvidas sobre a presença de isquemia miocárdica. O protocolo é o mesmo, mas nesses casos é importante avaliar não somente a contratilidade parietal, mas também a presença de gradiente intraventricular,^327^ uma vez que fatores anatômicos e hemodinâmicos cardíacos estão mais presentes nesse subgrupo de pacientes.

Em resumo, a ecocardiografia de estresse desempenha um papel de grande importância no diagnóstico e prognóstico da DAC nas mulheres.

### 29.3. Idosos

A população de pacientes com suspeita de DAC com mais de 65 anos é especialmente vulnerável e está em constante crescimento. A busca pela abordagem diagnóstica mais eficaz deve ser constante a fim de garantir o tratamento ideal e os melhores desfechos.

A ecocardiografia de estresse físico ou farmacológico surge como ferramenta diagnóstica fundamental, combinando os benefícios da imagem cardíaca com a análise da função cardiovascular sob estresse.

Em pacientes com mais de 65 anos, as informações provenientes da ecocardiografia de estresse (especialmente a resposta do volume sistólico final do VE e a FE durante o exercício), quando incorporadas aos critérios clínicos, aos dados do eletrocardiograma e ao ecocardiograma em repouso, aprimoram a capacidade de prever eventos cardíacos e a mortalidade por todas as causas.

Em um estudo com 2.632 idosos (56% homens) capazes de se exercitar, a EEF permitiu valor prognóstico adicional em relação às variáveis clínicas (idade, diabetes, infarto prévio e teste ergométrico). No esforço físico, a carga de exercício foi o melhor preditor de eventos cardíacos e morte.^327^

Diante da incapacidade de se exercitar devido às particularidades dessa faixa etária, a EED tem sido apontada como alternativa viável. Biagini et al.^329^ estudaram 1.434 pacientes com idade > 65 anos (média 72 ± 3 anos) submetidos a EED para avaliação de DAC. A presença de anormalidades na motilidade parietal foi considerada preditor independente de todas as causas de mortalidade e de desfechos adversos, enquanto o exame normal estava correlacionado com baixa incidência de eventos cardíacos. Cortigiani et al.^330^ demonstraram que a taxa de eventos nos octogenários foi 4,5 vezes maior do que nos idosos entre 65 e 69 anos.

A segurança e a acurácia conferem à ecocardiografia de estresse um papel crucial na avaliação diagnóstica e prognóstica de pacientes idosos, contribuindo para o manejo adequado desses indivíduos.

## 30. Ecocardiografia de Estresse na Pediatria: Indicações e Protocolos

Enquanto a ecocardiografia de estresse é uma técnica bem estabelecida na cardiologia para pacientes adultos, a baixa prevalência de patologias como DAC, doenças valvares e cardiomiopatia nas crianças causa uma escassez de dados publicados sobre essa modalidade e sua impopularidade de uso em pediatria. Assim, a ecocardiografia de estresse ainda não é tão utilizada em pacientes pediátricos quanto em pacientes adultos devido às dificuldades inerentes à realização do exame. Poucos centros utilizam essa modalidade em pacientes pediátricos e há poucas publicações na literatura.

A utilização do estresse amplia a avaliação ecocardiográfica, fornecendo dados em um ambiente fisiológico que se assemelha mais ao estado tipicamente ativo dessa faixa etária.^331^

Assim como na população adulta, as duas questões fundamentais que se busca responder são: 1) há isquemia miocárdica induzível? 2) há alterações hemodinâmicas significativas?^332^

Como nos adultos, o tipo de estresse pode ser físico, na esteira, na bicicleta e ciclomaca, ou farmacológico, com dobutamina e, eventualmente, dipiridamol.

A EEF é bem tolerada em crianças, pois não requer acesso venoso nem sedação, porém só pode ser realizada em crianças acima de 6-7 anos que são capazes de realizar o exercício e têm estatura suficiente para caminhar na esteira ou pedalar na bicicleta ou ciclomaca.^332-335^ Em geral, utiliza-se o protocolo de Bruce modificado nas duas modalidades.^332-335^ O protocolo de James também pode ser utilizado na bicicleta/ciclomaca.^333^ Já a EED não tem limitação de idade; porém, em crianças menores de 8 anos, é necessário sedação profunda e/ou anestesia geral, e em crianças maiores pode ser necessário sedação consciente.^333^ Os protocolos utilizados são semelhantes aos usados com adultos.^333-336^ Geralmente, a infusão de dobutamina começa com 5 mcg/kg/min e é aumentada em intervalos de 3 minutos para 10, 20, 30, 40, podendo chegar a 50 mcg/kg/min. Se a FC máxima alvo (85% da FC máxima para a idade) não for atingida para a obtenção de um teste conclusivo, uma dose baixa de atropina (0,01 mg/kg) é administrada na dose máxima de dobutamina. Contudo, a FC a ser atingida nessa população é muito alta.

Uma alternativa é usar doses baixas ou moderadas (entre 5 e 20 mcg/kg/min) em infusão contínua de dobutamina para avaliar a reserva contrátil cardíaca.^333,337^ A dose padrão do dipiridamol é de 0,84 mg/kg, conforme o padrão adulto já mencionado.

Os efeitos colaterais são semelhantes aos observados em pacientes adultos.^333-336^ Complicações como arritmias ventriculares sustentadas são raras, mas não há dados suficientes sobre essas complicações em pacientes pediátricos na literatura.^333^

As principais indicações para realização de ecocardiografia de estresse em pediatria são: avaliação de isquemia miocárdica, detecção precoce de disfunção miocárdica, avaliação dinâmica de gradientes na CMH, avaliação da função do ventrículo direito e avaliação para a realização de atividades físicas nos pacientes com cardiopatias congênitas,^332^ além do exame também ter aplicação clínica no pós-transplante cardíaco e pós-quimioterapia.^333,338^

As Tabelas 30.1 e 30.2 apresentam as indicações, com um resumo dos artigos mais recentes.

**Tabela 30.1:** Indicações e tipos de ecocardiografia de estresse em crianças, adolescentes e adultos jovens

Indicação	Nº Pcts	Idade	Tipo de estresse	Achados
**Doença de Kawasaki**				
Pahl et al. (1995)^335^	28	10,7 anos (mediana)	EEF/esteira	EEF detectou alteração da contratilidade segmentar, sendo superior à avaliação da eletrocardiografia isolada
Noto et al. (1996)^336^	50	13,6 anos (média)	EED	EED foi segura e acurada para diagnóstico de estenose de artérias coronárias em crianças
Noto et al. (2014)^340^	58	23,8 anos (média)	EED	EED foi útil na estratificação de risco e na avaliação do prognóstico
**Doença Vascular do Enxerto Pós-TX Cardíaco** Perez et al. (2022)^334^	95	16,3 anos (mediana)	EEF/esteira	EEF alterada foi associada ao ↑de risco para eventos CV e óbito, enquanto EEF normal foi associada à sobrevida livre de eventos CV
**OAAC** Brothers et al. (2007)^341^	24	24 anos (mediana)	EEF/esteira e bicicleta	EEF foi útil para avaliação de isquemia e no cuidado pós-operatório
**PO Cirurgia de Jatene** Chen et al. (2013)^342^	32	16,2 ± 2,1 (média ± DP)	EEF/bicicleta	EEF demonstrou reserva contrátil diminuída em adolescentes e em adultos jovens no PO
**Sobreviventes de Câncer infantil** Ryerson et al. (2015)^343^	80	13 anos (média)	EEF/bicicleta	Pacientes apresentaram melhora da função diastólica e sistólica no exercício sugerindo capacidade de compensar disfunção cardíaca leve
**PO Cirurgia de Ross** Pauliks et al. (2012)^339^	26	14,9 anos (média)	EEF/esteira	EEF foi viável em crianças e a maioria apresentou capacidade ao exercício normal
**PO T4F** Bhatt et al. (2019)^344^	29	18.3 ± 4.83 ↓ performance ao exercício 12.8 ± 3.26 ↑ performance ao exercício (média ± DP)	EEF/esteira	A resposta cronotrópica preservada, mais do que reserva contrátil do VD, foi um fator importante para o desempenho ao exercício
**Estenose de Tubo VD-TP** Hasan et al. (2012)^345^	40	17 anos (mediana)	EEF/esteira	EEF detectou disfunção do VD e do tubo no pico do exercício
**Gradiente na VSVE na CMH** El Assaad et al. (2020)^346^	91	12 anos (mediana)	EEF/esteira e bicicleta	EEF foi segura e demonstrou ser efetiva na identificação de pacientes de baixo risco para eventos CV

CMH: cardiomiopatia hipertrófica; CV: cardiovasculares; DP: desvio padrão; EED: ecocardiografia de estresse com dobutamina; EEF: ecocardiografia de estresse físico; OAAC: origem anômala aórtica das artérias coronárias; PO: pós-operatório; TP: tronco pulmonar; TX: transplante; VE: ventrículo esquerdo; VSVE: via de saída de ventrículo esquerdo; ΔP: gradiente pressórico; VD: ventrículo direito.

**Tabela 30.2:** Indicações simplificadas da ecocardiografia de estresse em crianças, adolescentes e adultos jovens

Indicações de ecocardiografia de estresse em pediatria	Suspeita de doença arterial coronariana: Doença de Kawasaki, vasculopatia do enxerto pós transplante, operações que envolvem translocação coronariana, como o switch arterial na transposição das grandes artérias, origens ou cursos anômalos das artérias coronárias, atresia pulmonar com septo ventricular intacto, hiperlipidemia, diabetes mellitus dependente de insulina e estenose aórtica supravalvar
	Pós quimioterapia: Antraciclinas
	Avaliações de gradientes:Cardiomiopatia hipertrófica ou estenose aórtica e pulmonar
	Avaliações daresposta hemodinâmica e miocárdica em condições específicas: hipertensão pulmonar, cardiomiopatia dilatada, cardiopatias congênitas, regurgitação mitral e aórtica.

Para indicações específicas, os protocolos são ajustados para incluir, por exemplo, imagens Doppler das valvas atrioventriculares para estudar a regurgitação da valva aórtica ou medir gradientes nas vias de saída.

Nos pacientes pediátricos, ao contrário dos adultos, não há dados suficientes sobre o valor prognóstico e impacto clínico da ecocardiografia de estresse na avaliação dinâmica dos gradientes na estenose aórtica e na estenose pulmonar.^333,339^

Por fim, a ecocardiografia de estresse é ainda área em desenvolvimento na cardiologia pediátrica. Sua integração com o Doppler tecidual e tecnologia de *strain* tem permitido uma análise mais quantitativa da função sistólica e diastólica regional e global nesse cenário, mas ainda de modo incipiente. A colaboração entre cardiologistas adultos e pediátricos é necessária para que protocolos visando as particularidades das crianças sejam implementados e essa modalidade de exame seja mais utilizada nesse subgrupo.

## 31. Vantagens e Desvantagens da Ecocardiografia de Estresse em Pediatria

Tanto a EEF quanto a de estresse farmacológico podem ser utilizadas em pediatria.^33,99,347^ Contudo, o médico deve considerar as vantagens e desvantagens de cada abordagem para orientar os testes na população pediátrica (Tabela 31.1).

**Tabela 31.1:** Vantagens e desvantagens de cada abordagem para orientar a testagem na população pediátrica

Modalidade de Imagem	Vantagens	Desvantagens	Usos
Ecocardiograma de estresse físico (EEF)	Não exige punção intravenosa Não exige sedação Possível obter outros dados – ECG e possivelmente VO_2_	Equipamento adicional Paciente deve conseguir realizar exercício em ergômetro	
EEF de esteira	Semelhante a atividades da vida diária VO_2_ máx mais elevado Pode ser mais fácil induzir isquemia	Artefato de movimento Recuperação da frequência cardíaca rápida em crianças Necessita transferência para leito	Avaliação de Isquemia Obstrução do fluxo de VSVE Reserva contrátil
EEF de bicicleta ergométrica	Obtenção das imagens em vários momentos Menos artefatos de movimento Doppler pode ser possível	Cooperação em crianças menores Frequência cardíaca mais baixa VO_2_ mais baixo	Regurgitação valvar Estenose valvar atrioventricular Hipertensão pulmonar Disfunção diastólica
Eco de estresse farmacológico	Menos interferência pulmonar Não necessita transferência para leito Reduz problemas associados à recuperação da frequência cardíaca	Colocação de linha intravenosa	
Eco de estresse com dobutamina	Afeta frequência cardíaca e pressão arterial	Demora cerca de 20 minutos Perfil de efeitos colaterais: arritmias ventriculares, emergência hipertensiva, náusea/vômito	Obstrução coronária dinâmica Baixa dose – estenose aórtica de baixo fluxo e baixo gradiente
Eco de estresse com vasodilatador	Curta duração	Perfil de efeitos colaterais: sibilância, convulsões, hipotensão	Obstrução coronária fixa

A EEF apresenta algumas vantagens sobre o estresse farmacológico na população pediátrica. Primeiro, não há necessidade de obter acesso intravenoso para o estresse físico, minimizando o sofrimento do paciente pediátrico. Segundo, não há necessidade de sedação, que pode ser necessária para períodos de estudo mais longos associados a agentes farmacológicos. Por fim, o ecocardiografista muitas vezes pode escolher entre dois ergômetros para a ecocardiografia de esforço: uma esteira ergométrica ou um cicloergômetro.^3,331,341,346,348-350^

A ecocardiografia de exercício também apresenta várias desvantagens. Primeiro, o equipamento adicional necessário pode ser limitante para alguns laboratórios, dada a necessidade do transdutor pediátrico e do software de estresse. Além disso, a ecocardiografia de exercício requer cooperação significativa do paciente pediátrico, independentemente do ergômetro utilizado. Artefatos pulmonares devido a um aumento fisiológico na frequência respiratória podem limitar as janelas ecocardiográficas junto ao exercício desse subgrupo de pacientes pouco cooperativos. Por fim, a recuperação rápida da FC em crianças pode limitar a capacidade de obter imagens adequadas no pico de estresse.

O tipo de ergômetro selecionado tem vantagens e desvantagens específicas. As esteiras ergométricas se assemelham muito às atividades diárias e de academias. Além disso, geralmente é alcançado um VO_2_ mais elevado, que é muito bom, assim como o maior duplo produto. A principal desvantagem das esteiras ergométricas é que o paciente deve ser capaz de se transferir de forma rápida e segura da esteira para a cama de ecocardiografia devido ao rápido tempo de queda e recuperação da FC na população pediátrica^3^ e o examinador precisa obter essas imagens em curto espaço de tempo. Isso nem sempre é fácil, especialmente em crianças.

Os cicloergômetros supinos permitem imagens em vários períodos de tempo durante o teste de esforço, com monitorização contínua. Os cicloergômetros podem permitir a utilização do Doppler colorido ou espectral de maneira gradual e mais adequada nesse subgrupo, o que pode ser vantajoso ao observar estenose ou regurgitação valvar e hipertensão pulmonar,^3^ obstrução intraventricular na CMH e gradientes na estenose aórtica.^346,350^ Além disso, os cicloergômetros tendem a limitar os artefatos de movimento. Contudo, a cooperação pode ser mais difícil em pacientes mais jovens, pois estes devem continuar a pedalar em um ritmo constante. Normalmente, VO_2_ mais baixo e frequências cardíacas mais baixas são alcançados com cicloergômetros em comparação com esteiras ergométricas.^3^

A ecocardiografia de estresse farmacológico também pode ser realizada em crianças.^3,351,352,340^ Porém, a principal desvantagem é a necessidade de acesso intravenoso para administração do agente farmacológico. As vantagens incluem menor interferência pulmonar, avaliação em vários níveis do teste e não necessidade de transferência para uma cama para obtenção de imagens. Esta técnica limita os problemas associados à recuperação rápida da FC em crianças, uma vez que a monitorização ecocardiográfica é contínua.

Os dois principais agentes farmacológicos utilizados são a dobutamina e os vasodilatadores.^3^ A dobutamina simula o exercício, aumentando a FC e a PA para níveis próximos ao exercício máximo. É utilizado principalmente para provocar isquemia e anormalidade da motilidade parietal em áreas onde possa haver obstrução dinâmica do fluxo arterial coronário em crianças, e também na avaliação de origem anômala das artérias coronárias ou em receptores de transplante.^341,349,351,352^

Os vasodilatadores, como o dipiridamol, a adenosina ou o regadenoson, dilatam as artérias coronárias normais, causando isquemia e anormalidades na motilidade parietal em áreas onde há obstrução coronária fixa.^3^ Podem ser úteis especialmente na avaliação da doença de Kawasaki e de artérias coronárias reimplantadas. Os perfis de efeitos colaterais variam de acordo com o vasodilatador utilizado, como descrito anteriormente.

Apesar de haver muitas opções de ecocardiografia de estresse, ela permanece subutilizada em crianças. Os médicos que compreendem as vantagens e desvantagens de cada modalidade podem selecionar a opção mais apropriada para cada questão clínica.

## 32. Prognóstico da Ecocardiografia de Estresse

A indução de isquemia miocárdica causada por aumento da FC ≥85% de acordo com a idade, durante a ecocardiografia de estresse por esforço físico ou por infusão intravenosa de dobutamina, é um método eficaz para o manejo clínico e prognóstico da cardiopatia isquêmica. A resposta normal é um aumento contrátil de todos os 16-17 segmentos ventriculares.^202^ À resposta de cada segmento, seja normal ou hipercinética, é atribuído um valor de 1 (hipocinesia leve ou moderada) 2 (hipocinesia grave), 3 (acinesia), 4 ou 5 (discinesia). Para simplificar, atualmente utiliza-se mais o seguinte esquema: 1 = normal ou hipercinético, 2 = hipocinético (em qualquer grau), 3 = acinético e 4 = aneurisma. A soma permite calcular o WMSI pela divisão de todos os segmentos exibidos por 16 ou 17 segmentos analisados. O resultado normal é 16/16 = 1 ou 17/17=1 e indica contratilidade global normal. Em um exemplo anormal, a hipocinesia moderada de dois segmentos soma 4, mais acinesia 4 de um segmento, soma 8, divide 16/8 e o WMSI é 2, anormal.

Em 3.121 pacientes^202^ divididos em três grupos de risco de acordo com WMS acompanhados por 3 anos, WMSI de baixo risco de 1 teve eventos de 0,8% ao ano; com WMSI de 1,1-1,7 o risco intermediário, de 2,6% ao ano; e com alto risco de WMS > 1,7, os eventos foram de 5,5% por ano.

Outro estudo, com 1.560 pacientes, relacionou o pico do WMSI com a FEVE.^353^ Os participantes foram divididos em três grupos com seguimento de 3 anos. O estudo observou que o risco baixo teve índice de eventos de 0,7%/ano, o intermediário 2,0%/ano e o alto 4,4%/ano. Os eventos foram infarto do miocárdio, morte ou outras complicações cardiovasculares.

Em 2.575 indivíduos portadores de DAC com ecocardiografia de estresse de pico negativo,^354^ classificados em dois grupos, sem alteração contrátil em repouso ou durante exercício máximo, ou com alteração contrátil prévia (por infarto) não agravada durante a ecocardiografia de estresse, o critério de anormalidade foi o desenvolvimento de nova lesão coronariana ≥50% em um ano. A mortalidade por qualquer causa em indivíduos com resultados negativos em 1 ano foi de 1,1% e naqueles com doença coronariana prévia foi de 3,1%/ano. Os preditores de novas lesões coronarianas em um ano foram sexo masculino, diabetes, doença coronariana prévia e ecocardiografia de estresse não isquêmico porém anormal. Esses pacientes com ecocardiografia de estresse negativa devem ser acompanhados devido ao maior risco de morbidade e mortalidade, mesmo que o resultado do exame atual seja negativo.

Em 609 pacientes com BRE^355^ investigados com ecocardiografia de estresse de exercício, com acompanhamento de quase 5 anos, o WMSI foi obtido em repouso e pós-exercício. A diferença do WMSI (delta) entre os dois momentos do teste foi de 29%. A mortalidade de pacientes com isquemia foi de 25% e não isquemia de 13%.

Os eventos cardíacos maiores foram de 18% vs. 10% e o delta do WMSI é considerado um preditor independente.

A ecocardiografia de estresse tem maior valor preditivo em relação aos demais parâmetros clínicos.

As principais causas da finalização/interrupção do exame são: aparecimento ou agravamento da alteração contrátil, angina pectoris, alterações isquêmicas no ECG, arritmias significativas, hipertensão arterial ou hipotensão significativas, dispneia ou fadiga limitantes e tremor intenso. A finalização do teste por interrupção ou por chegar ao final do protocolo sem atingir os *endpoints* de alteração contrátil ou obtenção da FC alvo deve ser relatada no laudo.

Diretrizes de ecocardiografia de estresse que tratam sobre os riscos e prognóstico futuro do método foram publicadas recentemente.^3,7^

## 33. Novas Aplicações da Ecocardiografia de Estresse no Pós-Radioterapia de Tórax e no Vasoespasmo

A radioterapia (RT) é um dos pilares do tratamento oncológico. Doses elevadas de radiação no tórax são usadas com frequência no tratamento de câncer de pulmão, esôfago, sistema linfático e mama. Devido à proximidade anatômica, o coração invariavelmente recebe doses de radiação que podem induzir cardiotoxicidade. Estudos mostram que a RT acelera o processo de aterosclerose coronariana, calcificação valvar e fibrose pulmonar e do pericárdio.

A toxicidade cardiovascular (CV) relacionada à RT é progressiva, tipicamente se desenvolve 5 a 10 anos após o tratamento inicial e pode causar DAC e insuficiência cardíaca (IC) com incidência até seis vezes maior do que na população geral. A latência entre a RT e o aparecimento da DAC varia de alguns anos a várias décadas, dependendo do risco CV pré-tratamento, idade do paciente, doses acumuladas de RT e tratamentos combinados com quimioterapia.^356^ Uma avaliação minuciosa dos riscos CV com escores de riscos amplamente difundidos, assim como a avaliação de alterações cardíacas preexistentes, se torna fundamental.

Ao considerar que o paciente submetido a RT normalmente possui uma grande carga acumulada de radiação, métodos de imagem que não exigem doses extras se tornam prioritários.^357,358^

A ecocardiografia de estresse e a busca por alteração de contratilidade regional e função ventricular esquerda são o protocolo mais seguido nos últimos 40 anos.

Em artigo recente, um grupo de estudiosos liderado pelo Dr. Eugenio Picano sugere um protocolo ABCDE expandido, que inclui os passos F, G, P e R devido à complexidade multivariada das causas de dano. A avaliação valvar mitral, a da progressão de sua calcificação e a repercussão frente ao estresse formam o passo F. A avaliação da progressão da calcificação valvar aórtica e seu gradiente são o passo G. Testar a resistência vascular pulmonar é o passo P e as alterações da função do VD representam o passo R.^359^

Informações adicionais podem ser relevantes e devem ser estimuladas quando possível. Uma avaliação pré-exame minuciosa e um exame de estresse direcionado com ênfase prioritária na dúvida diagnóstica, baseado no bom senso clínico, parecem ser o mais adequado.

O vasoespasmo coronariano é umas das principais causas de estenose dinâmica das artérias coronárias e pode causar angina, angina instável, infarto agudo do miocárdio, síncope e morte súbita. Seu diagnóstico continua a ser um grande desafio clínico. O teste provocativo invasivo com ergonovina foi descrito em vários estudos, com algumas ressalvas, como mencionado anteriormente. O estudo invasivo expõe o paciente a radiação e a contrastes que podem ser nefrotóxicos, além da ergonovina não estar disponível comercialmente em vários países. Estudos descrevem protocolos não invasivos com ergonovina, adenosina ou dobutamina.^360^ Porém, o protocolo que tem se mostrado mais promissor é o de hiperventilação (5 minutos de hiperventilação com frequência respiratória de 25 ipm) combinada com exercício em bicicleta (incremento de 25 W a cada 2-3 min). Esses dois passos podem esclarecer se a angina é por vasoespasmo, ao alterar a contratilidade regional (passo A), ou por disfunção microvascular coronariana (passo D). Estudos de validação, teste de eficácia e segurança em larga escala desse protocolo ainda são necessários. Dados adicionais devem ser publicados após a conclusão do estudo Stress Echo 2030.^261^

## 34. Ecocardiografia de Estresse com o Protocolo ABCDE

**Figura 34.1:**
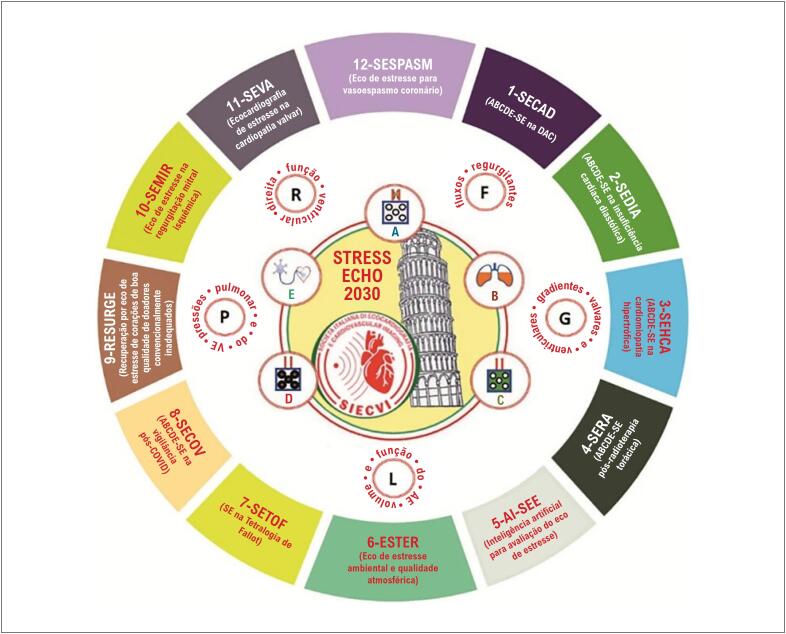
O "coração" do protocolo ABCDE do Stress Echo 2030.^261^

O teste funcional com ecocardiografia de estresse se baseia na detecção de anormalidades da motilidade regional com ecocardiografia bidimensional e está incorporado nas diretrizes clínicas. No entanto, subutiliza a versatilidade única da técnica, ideal para descrever as diferentes anormalidades funcionais subjacentes à mesma resposta ao movimento da parede durante o estresse. O protocolo ABCDE, idealizado pelo Dr Eugenio Picano na Itália, iniciou com o estudo multicêntrico Stress Echo 2020 e posteriormente o Stress Echo 2030, com o objetivo de transcender a utilização da ecocardiografia de estresse para uma gama de aplicações onde ela pode acrescentar informações relevantes^261^ (Figura 34.1).

Cinco parâmetros convergem conceitual e metodologicamente no protocolo ABCDE,^261^ avaliando múltiplas vulnerabilidades do paciente isquêmico.

As cinco etapas do protocolo ABCDE são: 1) A: movimento/espessamento regional da parede (amplamente difundida); 2) B: linhas B por ultrassonografia pulmonar avaliando congestão pulmonar extravascular; 3) C: reserva contrátil do VE por ecocardiografia volumétrica bidimensional; 4) D: reserva de velocidade de fluxo coronariano na coronária descendente anterior médio-distal esquerda com Doppler pulsátil; e 5) E: avaliação da FC por eletrocardiografia com uma derivação.

**Figura 34.2:**
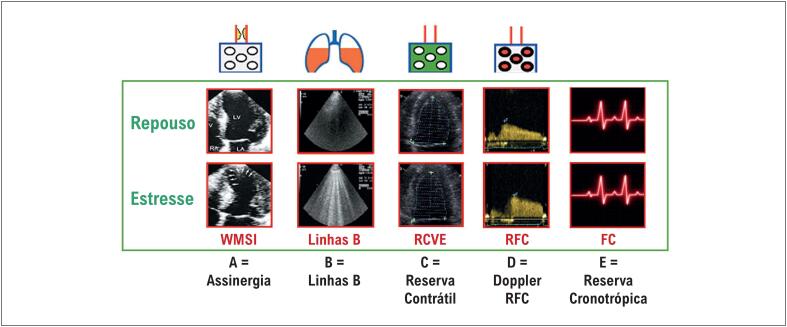
Integração de quatro principais imagens à ecocardiografia de estresse. Os quatro objetivos fisiopatológicos são: avaliação da estenose epicárdica (A = assinergia), congestão pulmonar (B = linhas B), função miocárdica do VE (C = reserva contrátil) e função microvascular coronariana (D = Doppler RFC), E = reserva cronotrópica, que pode ser avaliada adicionalmente.^261^ RFC: reserva de fluxo coronariano; WMSI: Wall Motion Score Index - Em português, Índice de Escore da Motilidade da Parede; RCVE: Reserva Contrátil do Ventrículo Esquerdo; FC: Frequência Cardíaca.

A ecocardiografia de estresse ABCDE oferece informações sobre cinco reservas funcionais: fluxo epicárdico (A); reserva diastólica/congestão (B), reserva contrátil (C), microcirculação coronariana (D) e reserva cronotrópica (E) (Figura 34.2).

O novo formato é mais abrangente e permite melhor caracterização funcional, estratificação de risco e adaptação personalizada da terapia instituída.

O protocolo ABCDE^261^ é um teste funcional adequado para todos os tipos de estresse e todos os pacientes e vai além da DAC. Ele responde à necessidade de sustentabilidade nos cuidados de saúde, uma vez que requer tecnologia universalmente disponível e é de baixo custo, isento de radiação e praticamente inócuo.

### 34.1. Protocolo ABCDE

- **A para Assinergia:**
  É baseado na suposta contratilidade miocárdica, ou seja, alteração induzida por estresse na presença de limitação de fluxo pela estenose coronariana. O desequilíbrio entre oferta e demanda de oxigênio leva à redução do espessamento radial do miocárdio, parâmetro às vezes limitado por sua natureza semiquantitativa e subjetiva. A dependência do operador pode ser minimizada (mas não eliminada) por treinamento especializado e pela adoção de medidas como a acreditação e o controle de qualidade dos laboratórios de ecocardiografia.
  A avaliação da motilidade regional indica que quanto maior o número de segmentos afetados (definidos universalmente), pior o prognóstico. Esse parâmetro é centrado na DAC.
- **B para Linhas-B:**
  O aumento da pressão atrial esquerda constitui a base de diversas condições. Sua consequência fisiopatológica tem relação com a saída de líquido no interstício pulmonar e pericapilar como substrato hidrostático. Na sua forma aguda, esse líquido pode ser detectado por ultrassom na forma de linhas de eco que é capaz de reverberá-lo. Durante a EEF, a indução de processos fisiológicos que podem provocar o limite da homeostase da doença é um parâmetro com valor prognóstico significativo.^361^
- **C para Contratilidade:**
  A contratilidade é a capacidade inerente do miocárdio de se contrair independentemente de alterações na pré ou pós-carga.
  Durante a ecocardiografia de estresse, o volume sistólico final do VE (VSF) é progressivamente menor, resultando em picos de PA mais elevados e aumento da carga. A relação entre a PAS e o VSF prediz a capacidade de esvaziamento do VE.^362^
  A elastância do VE mede a reserva contrátil por meio da comparação do seu desempenho em repouso e condições de estresse. Essa medida pode ser obtida pela divisão da relação PAS/VSF em condições de estresse, pelas condições de repouso.
  O estresse físico e com dobutamina tem valores validados, se estes estão acima de 2,0; e com o uso de vasodilatadores, a proporção que indica contratilidade apropriada está acima de 1,1.
- **D para Doppler da Artéria Coronária:**
  O fluxo coronariano pode ser afetado por condições epicárdicas de estenose e disfunção muscular.
  No primeiro cenário, em um determinado ponto de medida da vazão, pode ocorrer uma limitação pelo fluxo de sangue comprometido a jusante da obstrução em situações de estresse.
  Na segunda, diversas condições podem ocorrer, tais como hipertrofia perivascular e lesão do endotélio, alteração causada por inflamação e aterosclerose, impedindo o fluxo sanguíneo adequado.
  A RFC é medida por Doppler na ecocardiografia, usando a razão de velocidade na região medial ou distal da ADA sob estresse e em condições de repouso. Os valores normais são aqueles acima de 2,0 em qualquer modalidade de ecocardiografia de estresse.
- **E para Eletrocardiograma:**
  A variabilidade da FC em resposta ao estresse pode fornecer informações sobre a função autonômica do sistema de condução cardíaca, constituindo um marcador durante o teste. Esse parâmetro, a reserva de FC, é obtido pela divisão da FC máxima atingida pelo valor da FC em condições de repouso.
  É classificado como critério positivo se a relação for inferior a 1,80, para estresse físico ou dobutamina, e abaixo de 1,22 para vasodilatadores.
  Os betabloqueadores reduzem os valores da FC em condições de repouso e estresse, mas sem impacto nessa relação para fins prognósticos.^361^

Quando adicionado à avaliação tradicional da ecocardiografia de estresse, o protocolo ABCDE resulta em um espectro mais amplo de variáveis avaliado pelo protocolo de estresse. Juntos, eles permitem melhor estratificação de risco e auxiliam na definição de diagnósticos mais precisos.

Pacientes com doenças microvasculares e disfunção diastólica às vezes têm apresentações atípicas e podem se beneficiar desse novo protocolo, pois este amplia o poder diagnóstico da ecocardiografia de estresse tradicional.^363^

O protocolo ABCDE propõe a plena utilização da ecocardiografia de estresse consolidada de forma simplificada para uma gama imensa de informações adicionais, e apesar do maior consumo de tempo, vem ganhando espaço nos laboratórios de ecocardiografia por permitir maior ganho diagnostico.

## 35. Recomendações para o Manejo da Ecocardiografia de Estresse

**Table:** ECO ESTRESSE NA DOENÇA ISQUÊMICA CRÔNICA CONHECIDA OU SUSPEITA

Recomendações	Força de Recomendação	Certeza da Evidência
No diagnóstico de doença isquêmica crônica em pacientes com probabilidade pré-teste intermediária. Na vigência de sintoma de angina em pacientes com stent ou revascularização prévios. Na obstrução coronária moderada pela angiotomografia ou angiografia coronária: significado funcional. Na estratificação de risco pós-infarto do miocárdio. Na investigação de dispneia (equivalente anginoso).	**FORTE**	**ALTA**

**Table:** ECO ESTRESSE NA SALA DE EMERGÊNCIA: DOR TORÁCICA AGUDA

Recomendações	Forca de Recomendação	Certeza da Evidência
Na dor torácica aguda em pacientes de risco intermediário (sem anormalidades no ECG e/ou biomarcadores negativos). Em pacientes com suspeita de dor torácica anginosa e com troponina negativa ou discretamente aumentada, na estratificação do risco do paciente, desde que na ausência de alterações eletrocardiográficas ou recorrência de dor.	**FORTE**	**MODERADA**

**Table:** ECO ESTRESSE NA DOENÇA VALVAR

Recomendações	Forca de Recomendação	Certeza da Evidência
Ecocardiograma de estresse com dobutamina em baixa dose em pacientes portadores de estenose valvar aórtica baixo fluxo baixo gradiente (clássica) com FE<50%, para confirmar a gravidade da estenose. Ecocardiograma de estresse com exercício pode ser utilizado para avaliar a repercussão hemodinâmica da lesão valvar em pacientes que apresentam discrepância entre os sintomas e os dados do ecocardiograma de repouso.	**FORTE**	**ALTA**

**Table:** ECO ESTRESSE DIASTÓLICO

Recomendações	Forca de Recomendação	Certeza da Evidência
Eco de estresse sob exercício preferêncialmente em ciclomaca ou em esteira ergométrica, para pacientes com dispneia a esclarecer, e: Disfunção diastólica grau 1; Escore H_2_FPEF ou HFA-PEFF com pontuação intermediária (2–5 pontos e 2–4 pontos respectivamente).	**FORTE**	**ALTA**

**Table:** ECO ESTRESSE COM AGENTES DE REALCE ULTRASSONOGRÁFICO (ARUS) PARA ANÁLISE DA BORDA ENDOCÁRDICA

Recomendações	Forca de Recomendação	Certeza da Evidência
Eco de estresse com ARU, quando dois ou mais segmentos miocárdicos contíguos não são visualizados (no plano apical), para melhorar a acurácia diagnóstica.	**FORTE**	**ALTA**

**Table:** ECO ESTRESSE COM AGENTES DE REALCE ULTRASSONOGRÁFICO (ARUS) PARA ANÁLISE DA PERFUSÃO MIOCÁRDICA

Recomendações	Forca de Recomendação	Certeza da Evidência
Avaliação de perfusão miocárdica para aumento da acurácia na identificação da DAC obstrutiva e para a análise de viabilidade miocárdica.	**FRACA**	**ALTA**

**Table:** ECO ESTRESSE EM PEDIATRIA

	Forca de Recomendação	Certeza da Evidência
Parece vantajoso ao observar estenose ou regurgitação valvar, obstrução dinâmica da via de saída do ventrículo esquerdo e direito. Hipertensão arterial pulmonar. Avaliação de isquemia miocárdica (OAAC, OACE, doença de Kawasaki, em pacientes submetidos a cirurgia cardíaca com manipulação de coronárias como em pós operatório de Jatene). Pós transplante para avaliar isquemia	**FRACA**	**BAIXA**

OAAC: Origem anômala aórtica das artérias coronárias; OACE: Origem anômala da coronária esquerda do tronco pulmonar.

## Data Availability

os conteúdos subjacentes ao texto do Posicionamento estão contidos no manuscrito.

## Lista de abreviaturas e siglas

ADA: Artéria descendente anterior
ARUS: Agente de realce ultrassonográfico
ATC: Angiotomografia das coronárias
BAV: Bloqueio atrioventricular
BCRE: Bloqueio completo do ramo esquerdo
BRD: Bloqueio do ramo direito
BRE: Bloqueio de ramo esquerdo
CCD: Cateterismo cardíaco direito
CCDC: Cardiopatia crônica da doença de Chagas
CMD: Cardiomiopatia dilatada
CMH: Cardiomiopatia hipertrófica
CV: Cardiovascular
DA: Doença arterial
DAC: Doença arterial coronariana
DC: Débito cardíaco
DCV: Doença cardiovascular
DVM: Doença valvar mitral
EAO: Estenose aórtica
ECG: Eletrocardiograma
EED: Ecocardiografia de estresse farmacológico com dobutamina
EEDiast: Ecocardiografia de estresse diastólico
EEDIP: Ecocardiografia de estresse com dipiridamol
EEE: Ecocardiografia de estresse de esforço
EEF: Ecocardiografia de estresse físico
EM: Estenose mitral
ETT: Ecocardiografia transtorácica
FA: Fibrilação atrial
FC: Frequência cardíaca
FD: Função diastólica
HP: Hipertensão pulmonar
IAO: Insuficiência aórtica
IC: Insuficiência cardíaca
ICFEp: Insuficiência cardíaca com fração de ejeção preservada
IM: Índice mecânico
LFLG: *low-flow, low-gradient*
MACE: eventos cardiovasculares adversos maiores (*major adverse cardiovascular events*)
MCPD: Marca-passo definitivo
PA: Pressão arterial
PAD: Pressão arterial diastólica
PAS: Pressão arterial sistólica
PEG: Polietilenoglicol
PET: Tomografia por emissão de pósitrons (*positron emission tomography*)
PEVE: Pressão de enchimento do ventrículo esquerdo
PM: Perfusão miocárdica
PSAP: Pressão sistólica da artéria pulmonar
RC: Reserva contrátil
RFC: Reserva de fluxo coronariano
RM: Reserva miocárdica
RNM-C: Ressonância magnética do coração
RT: Radioterapia
SCA: Síndrome coronariana aguda
SLG: *Strain* longitudinal global
SPECT: Tomografia computadorizada por emissão de fóton único (*single-photon emission computed tomography*)
SPI: Índice pós sistólico
SR: Strain rate
VD: Ventrículo direito
VE: Ventrículo esquerdo
VSF: Volume sistólico final
VSVE: Via de saída do ventrículo esquerdo
WMSI: Escore de motilidade parietal (*wall motion score index*)
