# Supplementary material for: Diretriz sobre Ecocardiografia de Estresse – 2026
Source: Arq Bras Cardiol. 2026 May 4;123(4):e20260223. [Article in Portuguese] doi: 10.36660/abc.20260223 (PMC13398863; doi:10.36660/abc.20260223)
Supplement: Translation

# Translation: Guideline on Stress Echocardiography – 2026

**DOI:** 10.36660/abc.20260223i

**Authors:** Ana Cristina Camarozano, Salvador Vicente Spina, José Roberto Matos Souza, Marcelo Dantas Tavares de Melo, Daniela do Carmo Rassi Frota, Silvio Henrique Barberato, Fabio Villaça Guimarães, Harry Acquatella, Vera Márcia Lopes Gimenes, Jorge Alfredo Lowenstein, Ricardo H Pignatelli, José Maria Del Castillo, Joselina Luiza Menezes Oliveira, José Sebastião de Abreu, José Luis de Castro e Silva Pretto, Issam Shehadeh, Alex dos Santos Felix, Mercedes Maldonado Andrade, Antônio Carlos Sobral Sousa, Maria Estefânia Bosco Otto, Andrea de Andrade Vilela, Pedro Gutierrez Fajardo, Liz Andréa Villela Baroncini, Cristiano Vieira Machado, Fábio Cañellas Moreira, José María Hernández Hernández, Marco Stephan Lofrano Alves, Samira Saady Morhy, Márcio Silva Miguel Lima, Miguel Osman Dias Aguiar, Wilson Mathias, Letícia dos Santos de Oliveira Rocha, João César Nunes Sbano, Barbara Athayde Linhares Martins Vrandecic, Fabio Roston, Arnaldo Rabischoffsky, Juliana Paixão Etto Rolim, Raphael Aparecido Barreto da Silva, Guilherme de Rossi, Kamila Fernanda Staszko, Jolie Britt, Marcelo Luiz Campos Vieira

**Affiliations:** 1 Curitiba PR Brazil Universidade Federal do Paraná, Curitiba, PR – Brazil; 2 Buenos Aires BA Argentina Hospital Aeronáutico Central, Buenos Aires, BA – Argentina; 3 Ramos Mejía Pcia BA Argentina Centro Médico Don Bosco, Ramos Mejía Pcia, BA – Argentina; 4 Campinas SP Brazil Universidade de Campinas, Campinas, SP – Brazil; 5 João Pessoa PB Brazil Universidade Federal da Paraíba, João Pessoa, PB – Brazil; 6 Goiânia GO Brazil Hospital das Clinicas da Universidade Federal de Goiás, Goiânia, GO – Brazil; 7 Curitiba PR Brazil Pontifícia Universidade Católica do Paraná, Curitiba, PR – Brazil; 8 Marilia SP Brazil Instituto do Coração de Marília, Marilia, SP – Brazil; 9 Caracas CCS Venezuela Centro Medico de Caracas, Caracas, CCS – Venezuela; 10 São Paulo SP Brazil Hospital do Coração, São Paulo, SP – Brazil; 11 Buenos Aires BA Argentina Cardiodiagnóstico, Investigaciones Medicas Buenos Aires, Buenos Aires, BA – Argentina; 12 Houston TX United States of America Texas Children's Hospital- Baylor College of Medicine, Houston, TX – United States of America; 13 Recife PE Brazil Escola de Ecografia de Pernambuco, Recife, PE – Brazil; 14 Recife PE Brazil Universidade Católica de Pernambuco, Recife, PE – Brazil; 15 Aracaju SE Brazil Universidade Federal de Sergipe, Aracaju, SE – Brazil; 16 Fortaleza CE Brazil Clinicardio de Fortaleza e Cardioexata, Fortaleza, CE – Brazil; 17 Passo Fundo RS Brazil Hospital São Vicente de Paulo, Passo Fundo, RS – Brazil; 18 Esteio RS Brazil Clínica Cardiovision, Esteio, RS – Brazil; 19 Rio de Janeiro RJ Brazil Instituto Nacional de Cardiologia, Rio de Janeiro, RJ – Brazil; 20 Rio de Janeiro RJ Brazil Diagnósticos da América S.A. (DASA-RJ), Rio de Janeiro, RJ – Brazil; 21 Rio de Janeiro RJ Brazil Complexo Hospitalar Americas, Rio de Janeiro, RJ – Brazil; 22 São Paulo SP Brazil Instituto Dante Pazzanese de Cardiologia, São Paulo, SP – Brazil; 23 Aracaju SE Brazil Hospital São Lucas, Rede D'Or de Aracaju, Aracaju, SE – Brazil; 24 Brasília DF Brazil Universidade de Brasília, Brasília, DF – Brazil; 25 São Paulo SP Brazil Hospital Israelita Albert Einstein, São Paulo, SP – Brazil; 26 São Paulo SP Brazil Hospital Lefort Morumbi, São Paulo, SP – Brazil; 27 Guadalajara JAL Mexico Hospital de Especialidades San Francisco de Asis, Guadalajara, JAL – Mexico; 28 Guadalajara JAL Mexico Cardiotest, Guadalajara, JAL – Mexico; 29 Guadalajara JAL Mexico Laboratorio de Ecocaerdiografia, Guadalajara, JAL – Mexico; 30 Curitiba PR Brazil Hospital Nossa Senhora Das Gracas, Curitiba, PR – Brazil; 31 São Paulo SP Brazil Fleury Medicina e Saúde, Grupo Fleury, São Paulo, SP – Brazil; 32 Porto Alegre RS Brazil Santa Casa De Misericórdia De Porto Alegre, Porto Alegre, RS – Brazil; 33 Monterrey NL Mexico Imaging Cardiac Department. Doctors Hospital Auna, Monterrey, NL – Mexico; 34 São Paulo SP Brazil Instituto do Coração do Hospital das Clínicas da Faculdade de Medicina da Universidade de São Paulo, São Paulo, SP – Brazil; 35 São Paulo SP Brazil Hospital Beneficência Portuguesa de São Paulo, São Paulo, SP – Brazil; 36 Curitiba PR Brazil Hospital Pequeno Príncipe, Curitiba, PR – Brazil; 37 São Paulo SP Brazil Grupo Fleury Medicina e Saúde, São Paulo, SP – Brazil; 38 Belo Horizonte MG Brazil Hospital Biocor/Rede D'or, Belo Horizonte, MG – Brazil; 39 Londrina PR Brazil Clínica Clinicardio, Londrina, PR – Brazil; 40 Arapongas PR Brazil Hospital Norte Paranaense HONPAR, Arapongas, PR – Brazil; 41 Londrina PR Brazil Grupo Cura (Labimagem e Ultramed), Londrina, PR – Brazil; 42 Rio de Janeiro RJ Brazil Hospital Pró-Cardíaco, Rio de Janeiro, RJ – Brazil; 43 São Paulo SP Brazil Hospital Sírio Libanê, São Paulo, SP – Brazil; 44 São Paulo SP Brazil Alta (Rede Dasa), São Paulo, SP – Brazil; 45 Campinas SP Brazil Centro Especializado em Cardiologia LTDA, Campinas, SP – Brazil; 46 Curitiba PR Brazil Complexo do Hospital de Clínicas da Universidade Federal do Paraná (CHC-UFPR), Curitiba, PR – Brazil; 47 Houston TX United States of America Baylor College of Medicine, Houston, TX – United States of America; 48 Houston TX United States of America Texas Children's Hospital, Houston, TX – United States of America; 49 São Paulo SP Brazil Faculdade de Medicina da Universidade de São Paulo, São Paulo, SP – Brazil

**Table:** 

Guideline on Stress Echocardiography – 2026	
The report below lists declarations of interest as reported to the SBC by the experts during the period of the development of these statement, 2025/2026.	
Expert	Type of relationship with industry
Alex dos Santos Felix	Nothing to be declared
Ana Cristina Camarozano	Nothing to be declared
Andrea de Andrade Vilela	Nothing to be declared
Antônio Carlos Sobral Sousa	Nothing to be declared
Arnaldo Rabischoffsky	Nothing to be declared
Barbara Athayde Linhares Martins Vrandecic	Nothing to be declared
Cristiano Vieira Machado	Nothing to be declared
Daniela do Carmo Rassi Frota	Financial declaration A - Economically relevant payments of any kind made to (i) you, (ii) your spouse/partner or any other person living with you, (iii) any legal person in which any of these is either a direct or indirect controlling owner, business partner, shareholder or participant; any payments received for lectures, lessons, training instruction, compensation, fees paid for participation in advisory boards, investigative boards or other committees, etc. from the brazilian or international pharmaceutical, orthosis, prosthesis, equipment and implants industry: - AstraZeneca Brazil Ltda: Forxiga.
Fábio Cañellas Moreira	Nothing to be declared
Fabio Roston	Financial declaration A - Economically relevant payments of any kind made to (i) you, (ii) your spouse/partner or any other person living with you, (iii) any legal person in which any of these is either a direct or indirect controlling owner, business partner, shareholder or participant; any payments received for lectures, lessons, training instruction, compensation, fees paid for participation in advisory boards, investigative boards or other committees, etc. from the brazilian or international pharmaceutical, orthosis, prosthesis, equipment and implants industry: - Boston Scientific – Proctorship in left atrial appendage occlusion..
Fabio Villaca Guimarães filho	Nothing to be declared
Guilherme De Rossi	Other relationships Any economically relevant equity interest in companies in the healthcare or education industry or in any companies competing with or supplying to SBC: - Health area.
Harry Acquatella	Nothing to be declared
Issam Shehadeh	Financial declaration A - Economically relevant payments of any kind made to (i) you, (ii) your spouse/partner or any other person living with you, (iii) any legal person in which any of these is either a direct or indirect controlling owner, business partner, shareholder or participant; any payments received for lectures, lessons, training instruction, compensation, fees paid for participation in advisory boards, investigative boards or other committees, etc. from the brazilian or international pharmaceutical, orthosis, prosthesis, equipment and implants industry: - GE Healthcare: Hands-on training in stress echocardiography. Other relationships Funding of continuing medical education activities, including travel, accommodation and registration in conferences and courses, from the brazilian or international pharmaceutical, orthosis, prosthesis, equipment and implants industry: -Torrent: Cardiology; Medley Sanofi: Zinpass Eze.
João Cesar Nunes Sbano	Nothing to be declared
Jolie Britt	Nothing to be declared
Jorge Alfredo Lowenstein	Nothing to be declared
José Luis de Castro e Silva Pretto	Nothing to be declared
José Maria Del Castillo	Nothing to be declared
José María Hernández Hernández	Other relationships Funding of continuing medical education activities, including travel, accommodation and registration in conferences and courses, from the brazilian or international pharmaceutical, orthosis, prosthesis, equipment and implants industry: - Vitalmex: LVAD – Vitacor. Employment relationship with the brazilian or international pharmaceutical, orthosis, prosthesis, equipment and implants industry, as well as any employment relationship with health insurance companies or medical audit companies (including part-time jobs) in the year to which your declaration refers: - Vitalmex: LVAD – Vitacor.
José Roberto Matos Souza	Nothing to be declared
José Sebastião de Abreu	Nothing to be declared
Joselina Luzia Menezes Oliveira	Nothing to be declared
Juliana Paixão Etto Rolim	Nothing to be declared
Kamila Fernanda Staszko	Nothing to be declared
Letícia dos Santos de Oliveira Rocha	Nothing to be declared
Liz Andréa Villela Baroncini	Nothing to be declared
Marcelo dantas tavares de melo	Nothing to be declared
Marcelo Luiz Campos Vieira	Nothing to be declared
Márcio Silva Miguel Lima	Nothing to be declared
Marco Stephan Lofrano Alves	Nothing to be declared
Maria Estefânia Bosco Otto	Nothing to be declared
Mercedes Maldonado Andrade	Nothing to be declared
Miguel Osman Dias Aguiar	Other relationships Any economically relevant equity interest in companies in the healthcare or education industry or in any companies competing with or supplying to SBC: - Health area.
Pedro Gutierrez-Fajardo	Nothing to be declared
Raphael Aparecido Barreto da Silva	Financial declaration B - Research funding under your direct/personal responsibility (directed to the department or institution) from the brazilian or international pharmaceutical, orthosis, prosthesis, equipment and implants industry: - Lilly: obesity, lipoprotein(a); Amgen: heart failure, lipoprotein(a), coronary artery disease; Sanofi: atrial fibrillation; Vertex: renal failure; Boehringer: heart failure; AstraZeneca: dyslipidemia.
Ricardo H Pignatelli	Nothing to be declared
Salvador Vicente Spina	Nothing to be declared
Samira Saady Morhy	Nothing to be declared
Silvio Henrique Barberato	Financial declaration A - Economically relevant payments of any kind made to (i) you, (ii) your spouse/partner or any other person living with you, (iii) any legal person in which any of these is either a direct or indirect controlling owner, business partner, shareholder or participant; any payments received for lectures, lessons, training instruction, compensation, fees paid for participation in advisory boards, investigative boards or other committees, etc. from the brazilian or international pharmaceutical, orthosis, prosthesis, equipment and implants industry: - Pfizer: Amyloidosis; AstraZeneca: HFpEF. Other relationships Funding of continuing medical education activities, including travel, accommodation and registration in conferences and courses, from the brazilian or international pharmaceutical, orthosis, prosthesis, equipment and implants industry: - Pfizer: Amyloidosis.
Vera Marcia Lopes Gimenes	Nothing to be declared
Wilson Mathias Junior	Nothing to be declared

## Table of contents

**1. The Role of Resting Echocardiography Performed at the Time of Physical or Pharmacological Stress Echocardiography** 9
**2. The Evolution of Stress Echocardiography: from Inception to the Present Day** 9
**3. Ischemia-Inducing Methods** 9
  **3.1. Exercise Stress Echocardiography (Bicycle, Treadmill, or Recumbent Bicycle/Echo Couch)** 10
  **3.2. Pharmacological Stress Echocardiography** 10
  **3.3. Stress Echocardiography in Coronary Spasm Provocative Testing** 10
  **3.4. Pacing Stress Echocardiography** 11
**4. Exercise Stress Echocardiography (Treadmill, Cycle Ergometer, or Reclining Ergometer/Echo Couch)** 11
**5. Dobutamine Stress Echocardiography in the Assessment of Myocardial Ischemia** 13
  **5.1. Dobutamine Stress Echocardiography Protocol** 13
  **5.2. Type of Myocardial Response** 15
  **5.3. Assessment During DSE and in Recovery** 17
  **5.4. Transient Ischemic Dilatation of the Left Ventricle** 19
  **5.5. Comparison with Other Imaging Modalities** 19
  **5.6. Value of a Negative DSE** 19
  **5.7. Complications of DSE** 19
**6. Dipyridamole Stress Echocardiography** 20
  **6.1. Protocols** 20
  **6.2. Adverse Effects** 20
  **6.3. Contraindications** 20
  **6.4. Coronary Flow Reserve** 21
  **6.5. Accuracy** 22
**7. Adenosine or Regadenoson Stress Echocardiography** 22
  **7.1. Use During Stress Echocardiography** 22
    **7.1.1. Indications** 23
  **7.2. Protocol for Adenosine Stress Echocardiography** 23
  **7.3. Protocol for Regadenoson Stress Echocardiography** 23
  **7.4. Contraindications** 24
  **7.5. Adverse Effects** 24
  **7.6. Diagnostic Accuracy** 24
**8. Other Stress Echocardiography Modalities: Pacing, Ergonovine, Handgrip, and Hyperventilation** 24
  **8.1. Pacing Stress Echocardiography** 24
  **8.2. Addition of Handgrip Exercise to Stress Echocardiography** 24
  **8.3. Ergonovine and Hyperventilation Stress Echocardiography** 24
**9. Diastolic Stress Echocardiography** 25
  **9.1. Normal and Abnormal Response to Exercise of Diastolic Function** 25
  **9.2. Indications for Stress Echocardiography** 25
  **9.3. Protocols Used in Diastolic Stress Echocardiography** 26
  **9.4. Interpretation of Diastolic Stress Echocardiography** 26
**10. Stress Echocardiography in MINOCA (Myocardial Infarction with Nonobstructive Coronary Arteries) and in Microvascular Disease** 27
  **10.1. Pathophysiology** 27
  **10.2. Main Diagnostic Methods in MINOCA** 27
  **10.3. The Stress Echocardiogram in MINOCA** 27
**11. Stress Echocardiography in Mitral Valve Disease** 29
  **11.1. Mitral Stenosis** 29
  **11.2. Mitral Regurgitation** 30
  **11.3. Stress Echocardiography Protocol for Evaluation of Mitral Valve Lesions** 31
**12. Stress Echocardiography in Aortic Valve Disease** 31
  **12.1. Aortic Stenosis** 31
  **12.2. Aortic Insufficiency** 32
**13. Stress Echocardiography in Dilated Cardiomyopathy** 33
**14. Stress Echocardiography in Chagas Cardiomyopathy** 34
**15. Stress Echocardiography in Hypertrophic Cardiomyopathy** 36
**16. Stress Echocardiography in the Evaluation of Right Ventricular Function and Pulmonary Hypertension** 36
**17. Stress Echocardiography in Post-COVID-19 assessment** 37
**18. Stress Echocardiography and Coronary Flow Reserve in Native Vessels** 37
**19. Stress Echocardiography and Coronary Flow Reserve in Grafts** 39
  **19.1. Stress echocardiography after myocardial revascularization** 39
  **19.2. Coronary flow reserve after myocardial revascularization** 39
**20. Stress Echocardiography in the Assessment of Myocardial Viability** 39
  **20.1. Stress Echo Protocols for Assessment of Myocardial Viability** 41
  **20.2. Assessment of Myocardial Viability by Longitudinal Strain** 41
**21. Stress Echocardiography with Ultrasound Enhancing Agents for Delineation of the Endocardial Border** 43
  **21.1. Ultrasound Enhancing Agents** 43
    **21.1.1. Administration** 43
**22. Stress Echocardiography with Ultrasound Enhancing Agents for Myocardial Perfusion Analysis** 44
**23. Stress Echocardiography with Myocardial Strain Imaging in Ischemic Heart Disease** 46
  **23.1. Speckle Tracking-Derived Strain Versus Tissue Doppler-Derived Strain During Stress Echocardiography** 46
  **23.2. Two-Dimensional Strain During Stress Echocardiography: Diagnostic and Prognostic Value in Coronary Artery Disease** 47
  **23.3. Which Strain is Most Useful in Stress Echocardiography? Global or Regional? Longitudinal or Circumferential? And What is the Importance of Post-Systolic Thickening?** 48
  **23.4. Does Strain Have Ischemic Memory?** 49
  **23.5. Can Baseline Global Longitudinal Strain Predict Coronary Artery Disease in the Absence of Visually Apparent Wall Motion Abnormalities During Stress?** 50
  **23.6. Limitations** 51
**24. Stress Echocardiography with Myocardial Strain Imaging Beyond Ischemic Heart Disease** 51
**25. Three-Dimensional Stress Echocardiography** 52
  **25.1. Three-Dimensional Technology and Its Applications in Echocardiography** 52
  **25.2. The Application of Three-Dimensional Echocardiography in Stress Echocardiography** 52
**26. Stress Echocardiography in the Emergency Department: Indications and Applications** 54
  **26.1. Utility of Stress Echocardiography in the Emergency Department** 55
**27. Stress Echocardiography in the Heart Transplant Recipient** 56
**28. Stress Echocardiography in Special Subgroups (Left Bundle Branch Block, Right Bundle Branch Block, and Atrial Fibrillation)** 57
  **28.1. Left Bundle Branch Block** 57
  **28.2. Right Bundle Branch Block** 57
  **28.3. Atrial Fibrillation** 57
**29. Stress Echocardiography for Preoperative Assessment Before Vascular Surgery, in Women, and in Older Adults** 58
  **29.1. Preoperative Assessment Before Vascular Surgery** 58
  **29.2. Women** 58
  **29.3. Older Adults** 59
**30. Stress Echocardiography in Pediatrics: Indications and Protocols** 59
**31. Advantages and Disadvantages of Stress Echocardiography in Pediatrics** 61
**32. Prognostic Value of Stress Echocardiography** 62
**33. Novel Applications of Stress Echocardiography: After Radiation Therapy to the Chest and in Vasospasm** 63
**34. Stress Echocardiography with the ABCDE Protocol** 63
  **34.1. The ABCDE Protocol** 64
**35. Recommendations for the Management of Stress Echocardiography** 66
**References** 67

**Figure:** * May be considered in specific situations.

## 1. The Role of Resting Echocardiography Performed at the Time of Physical or Pharmacological Stress Echocardiography

One of the first descriptions of physical stress echocardiography (SE), published in 1979, compared images obtained at rest to those obtained during bicycle exercise.^1^ Analysis of the images in the article clearly shows the technical limitations of the time, with definition issues making both resting and exercise images difficult to interpret. Even so, regional wall motion abnormalities (RWMAs) were found in 10 patients, all of whom had significant coronary artery stenoses. The method has since evolved, and resting echocardiography now includes a wide range of refined diagnostic tools, such as Doppler (in its various modalities), digital recording of cardiac cycles with a high frame rate, reliable measurements of chamber volumes, analysis of ventricular and atrial deformation, estimation of intracavitary pressures, and analysis of valve areas, as well as a vast number of cardiovascular risk markers that can be analyzed at rest. This broad range of complex analyses, allowing thorough morphological and functional evaluation, has established resting echocardiography as a standalone modality.

Picano et al., in a recent European consensus statement,^2^ set out the limited roles of resting echocardiography, performed in conjunction with stress echocardiography, including assessment of image quality, mapping windows for standard views, ruling out other obvious causes of symptoms (e.g., pericardial effusion, aortic stenosis, hypertrophic cardiomyopathy), and, above all, eliminating contraindications to stress testing, such as acute ischemia, aortic dissection, and pulmonary embolism, among others. As defined in this consensus and other guidelines,^3^ the resting echocardiogram performed immediately before a stress echocardiogram is thus incomplete, as its purpose is to observe parameters that may undergo dynamic changes. It is a "snapshot" focused on the clinical indication for the imaging modality and on the resting abnormalities that should be evaluated under stress, and therefore cannot be regarded or requested as a complete morphological and functional assessment of a standard echocardiogram.

Therefore, the conventional resting echocardiogram is a distinct examination from SE and should preferably be performed at another time. It is also the first-line modality of choice which should precede SE to rule out situations that would limit, contraindicate, or altogether prevent performance of SE, such as those mentioned above, as well as assess individual patient variations which may limit ultrasound image quality and thus compromise interpretation of SE.

## 2. The Evolution of Stress Echocardiography: from Inception to the Present Day

Coronary artery disease remains a major cause of morbidity and mortality. Therefore, diagnostic and therapeutic resources capable of mitigating its effects from both an individual and community perspective remain objects of great medical interest, with a significant potential for socioeconomic impact.

The development of accessible diagnostic methods that are easy to perform, reproducible, low-cost, preferably non-invasive, and have good accuracy has always been a focus of research in cardiology, especially in the field of coronary artery disease.

The use of drugs to induce an imbalance between myocardial oxygen supply and consumption was initially proposed as an adjunct to electrocardiography, first using dipyridamole, and later, dobutamine. The induction of pharmacological stress in conjunction with echocardiography only became established following the initial work of Picano et al., using dipyridamole, and Berthe & Piérard in 1986, using dobutamine.^5^ These have become the most established drugs in daily clinical practice ever since, with well-defined dosage protocols, diagnostic criteria, and safety levels, often further combined with atropine to maximize the heart rate levels achieved and thus optimize diagnostic yield.^6-13^

Physical exercise, whether performed on a treadmill or cycle ergometer, remains the first-choice stress modality for patients who are physically and clinically able to tolerate exertion, as it is a more physiological method. In patients with limitations to physical exertion, such as those with orthopedic or neurological conditions, lung diseases, visual impairments, or poor coordination during physical activity, among others, pharmacological protocols are the preferred alternative.^7^

Both exercise and pharmacological SE have proven, through results obtained in recent decades, to be safe in older adults, women, patients with coronary artery disease, chronic kidney disease, valvular heart disease, and peripheral vascular disease, as well as in the post-transplant setting. Their diagnostic performance indices are quite high, similar to those of nuclear medicine methods, and they exhibit high agreement with anatomical-structural methods, such as computed tomography (CT) angiography and coronary angiography.^2,3,14,16^

It is worth noting that, in addition to its purpose of detecting myocardial ischemia, SE is extremely valuable for assessing myocardial viability in patients who have already experienced a cardiac event – a pathophysiological concept with diagnostic, therapeutic, and prognostic relevance for those with coronary artery disease.^10-12^

Finally, it also bears stressing that, beyond its role in coronary artery disease, SE is widely indicated in the assessment of diastolic dysfunction, especially in patients with heart failure with preserved ejection fraction (HFpEF); non-ischemic (infiltrative or storage) cardiomyopathies; mitral and aortic valve disease; congenital heart disease; pulmonary hypertension; athlete's heart; graft vascular disease; and in the post-transplant setting, among other indications.^2,3,14-20^

## 3. Ischemia-Inducing Methods

The primary objective of SE in coronary artery disease is to demonstrate the development of myocardial ischemia, its extent, and its distribution, and, secondarily, to stratify risk by demonstrating hemodynamic changes – ventricular dilatation, decreased blood pressure, diastolic dysfunction, altered coronary flow reserve (CFR) and/or myocardial reserve (MR), among others.^21^

Myocardial ischemia may manifest as the presence of significant obstructive epicardial lesions (> 50% left main coronary artery [LMCA] or > 70% other epicardial arteries), or as the presence of a compromised microcirculation without significant epicardial lesions. The utility of each test is based on their ability to detect these abnormalities^22^ (Figure 3.1).

**Figure 3.1:** Anatomical and functional objectives of stress echocardiography. LMCA: left main coronary artery; EA: epicardial artery; CFR: coronary flow reserve; MR: myocardial reserve; LBBB: left bundle branch block.

Another interesting role of SE is to objectively demonstrate a decrease in coronary flow through non-significant intermediate coronary lesions, with no evidence of ischemia on conventional tests, through measurement of the CFR.^23^

### 3.1. Exercise Stress Echocardiograph (Bicycle, Treadmill, or Recumbent Bicycle/Echo Couch)

Exercise SE is the most physiological of all provocative tests. It is based on demonstrating ischemia through an increase in the double product (maximum systolic blood pressure × peak heart rate achieved) and myocardial oxygen consumption. It is considered the first-line modality of choice, except when contraindicated or unavailable. Its utility is greatest in patients with significant epicardial lesions, primarily affecting the subendocardial layer, as assessed by wall motion (thickening), extent, and distribution, with significant risk when involvement includes three or more segments. Furthermore, it allows for simultaneous observation of the hemodynamic repercussions of ischemia: diastolic dysfunction (transmitral flow, E/e′), ventricular dilatation, mitral regurgitation, reduced myocardial reserve, arrhythmias, and pulmonary B lines, among others.

### 3.2. Pharmacological Stress Echocardiography

- Dobutamine: based on ability to demonstrate ischemia in the presence of significant epicardial lesions through an increase in double product and myocardial oxygen consumption by acting on adrenergic receptors, increasing cardiac inotropism and chronotropism. Used in patients who are unable to exercise, at centers where exercise SE is unavailable, or to assess myocardial ischemia and viability (the latter under discussion) in patients with baseline regional wall motion abnormalities. As useful as exercise SE;
- Vasodilators (dipyridamole and adenosine): act on the microcirculation, demonstrating ischemia through direct vasodilation of healthy arterioles to the detriment of diseased arterioles, inducing the so-called "coronary steal" phenomenon. This occurs due to a buildup of adenosine in the vascular system (indirectly when dipyridamole is given and directly when exogenous adenosine is given), where it exerts a very potent vasodilating effect. These drugs do not significantly increase the double product, allowing for a more practical assessment of CFR and myocardial strain. They are useful in patients with complete left bundle branch block (LBBB), a permanent pacemaker, contraindications to exercise or dobutamine, suspected microvascular disease, atrial fibrillation, hypertension, and those with symptomatic epicardial lesions.

### 3.3. Stress Echocardiography in Coronary Spasm Provocative Testing

- Ergonovine test: the "gold standard" due to its high sensitivity (93%) and specificity (91%). Stimulates α-adrenergic receptors.^24-26^
- 5-Hydroxytryptamine (serotonin): is high-risk, not indicated for routine use, has not achieved clinical reproducibility. Contractile changes indicative of ischemia occur early, even before pain or electrocardiogram (ECG) changes; may induce Takotsubo syndrome.^27^
- Hyperventilation test: induces coronary spasm by decreasing H+ ions in the plasma with an increase in pH (alkalosis), thus increasing intracellular calcium, inducing contraction and vasospasticity of arterial smooth muscle cells in the epicardial coronary arteries. Highly sensitive (90%).^28,29^
- Cold test: In the presence of endothelial dysfunction and elevated catecholamines, coronary vasoconstriction occurs.^28,29^
- The combination of exercise or dobutamine SE with the cold test and/or hyperventilation test can increase sensitivity and diagnostic power to identify coronary vasospasm.

### 3.4. Pacing Stress Echocardiography

Performed in patients with an implanted permanent pacemaker (PPM) with right atrial and/or right ventricular stimulation. The mechanism involves using the pacemaker to increase the heart rate, which increases the double product and induces ischemia. Regional wall thickening is assessed by taking into account the septal movements caused by the pacemaker catheter implantation site (Figure 3.2).

**Figure 3.2:** Pacemaker stress echocardiography according to the site of stimulation and septal movement.

This modality is highly sensitive (98%) and safe (96%), as the heart rate can be reduced instantaneously.^30,31^

It is a good alternative for diagnosis of ischemia in patients who cannot exercise, have contraindications to pharmacological testing, and have had a PPM implanted (Figure 3.2).

## 4. Exercise Stress Echocardiography (Treadmill, Cycle Ergometer or Bicycle)

Exercise stress echocardiography (ESE) is a testing modality that investigates myocardial function in an effective, physiological, and safe manner, seeking to replicate the daily routine of patients with preserved physical capacity. This practical, non-invasive examination provides valuable information regarding the diagnosis of myocardial ischemia, the behavior of blood pressure, and the etiology of cardiac arrhythmias, while also allowing evaluation of the behavior of pressure gradients in the left ventricular outflow tract, pulmonary artery systolic pressure, and valvular lesions. It is worth noting that analysis of these parameters must be correlated with the onset of symptoms (if any) before, during, and after physical exertion.^3^ Over the last two decades, the utility of this methodology for managing patients with various conditions, such as coronary artery disease, cardiomyopathies, valvular heart disease, and cardiac arrhythmias, has been demonstrated and recognized, including its utility in preoperative risk stratification for noncardiac surgery.^3^

Considering the development of new cardiac imaging modalities, such as CT coronary angiography and cardiac magnetic resonance (CMR) imaging, ESE has made surprising progress. Although CMR has demonstrated indisputable ability to diagnose various cardiac conditions in detail, its accuracy for diagnosing myocardial ischemia is not superior to that of ESE or myocardial perfusion scanning. Furthermore, because ESE is an easily accessible, low-cost, highly reproducible methodology, it is very useful for monitoring patients with these heart conditions over time.^33^ It is important that new generations of echocardiographers also take an interest in the method as a useful modality for both clinical diagnostics and scientific research, always seeking to improve their ability to obtain images of satisfactory quality, so as not to jeopardize interpretation.^7^

It is the echocardiographer's duty to conduct initial assessment of the clinical condition of the patient to be studied, as well as ascertain their preference of treadmill, cycle ergometer, or reclining ergometer ("echo couch") when the echocardiography laboratory offers more than one choice. It is also important to discuss the limitations of the various stress-inducing methods, so that the choice of method is based on the physical capacity of the individual as well as on the local conditions of each laboratory. The patient should be made aware that ESE integrates four components: ECG, the exercise stress test per se, a targeted transthoracic echocardiogram, and the stress echocardiogram, each of which must also take into account the patient's symptoms and physical capacity. The echocardiographer, in turn, must supervise every step of the examination: the resting phase, the pre-test phase, the stress phase, and finally, the recovery phase. The presence of paramedics throughout the examination is also necessary to ensure, above all, that the recording of ECG tracings and echocardiographic images is not compromised.

In most facilities, as is the case in our echo lab, the treadmill is the cornerstone of ESE, primarily because it is the preferred exercise modality for the majority of patients. Using this machine, an experienced echocardiographer can, in most cases, get the patient to attain the recommended maximum heart rate (220 – age), especially if the patient is aware of the need to reach this target. However, the quality of echocardiographic images is often compromised when maximum exertion is reached, due to lung hyperventilation. Therefore, it is recommended that the patient be made to reach an intermediate heart rate between the submaximal rate (85% of the maximum heart rate) and the maximum heart rate for optimal image interpretation, as well as for images to ideally be obtained no later than 90 seconds after maximum exertion (when using a treadmill). This procedure facilitates the acquisition and recording of echocardiographic images at peak physical exertion, with the patient lying on the examination table in the left lateral decubitus position, which should be obtained rapidly after achieving the target heart rate in order not to reduce the sensitivity of the test.. When using a cycle ergometer or stationary bicycle, the endpoint of exertion is almost always determined by the patient (provided they reach the target heart rate to ensure that the test is conclusive), and obtaining images during exertion while the patient is seated is more laborious for the echocardiographer. The reclining ergometer or "echo couch" was developed to address this issue. It makes capturing images somewhat easier, since the individual remains in a semi-supine position throughout the examination.^32^ The most elevated setting on the reclining ergometer allows for the best test quality, by allowing the patient to exert greater thrust with the legs.

Currently, in addition to the classic indications for ESE – patients who are capable of physical exertion and present with chest pain, suspected or known coronary artery disease, fatigue, or suspected HFpEF – the 2021 American Heart Association Guidelines for Hypertrophic Cardiomyopathy (HCM)^33^ recommend initial assessment with left ventricular outflow tract (LVOT) gradient measurement in all HCM patients at rest, during Valsalva strain, and in semi-supine, sitting, or standing positions. For asymptomatic patients with a resting LVOT gradient < 50 mm Hg, ESE is indicated. HCM is considered obstructive when the gradient is > 50 mm Hg. A labile or obstructive LVOT gradient is defined as one not observed during resting echocardiography, but provoked by stress testing. It is also worth noting that a resting LVOT gradient ≥ 50 mm Hg is a contraindication to exercise testing. The presence of systolic anterior motion (SAM) of the mitral valve, mitral regurgitation, ventricular dyssynergy, and left ventricular diastolic dysfunction grade 3, should also be assessed^34^ (Figure 4.1).

**Figure 4.1:** Proposed flow chart for the use of exercise stress echocardiography in patients with HCM without a gradient (LVOTG = 0 mm Hg) or with a gradient (LVOTG < 50 mm Hg). HCM: hypertrophic cardiomyopathy; LVOTG: left ventricular outflow tract gradient.

ESE protocols for the stationary bicycle/cycle ergometer, treadmill, and reclining ergometer/echo couch modalities are given in Figure 4.2.

**Figure 4.2:** Protocols for exercise stress echocardiography using a stationary bicycle/cycle ergometer, treadmill, or reclining ergometer/echo couch (in the supine position). Image capture on cycle and reclining ergometers is continuous, while on a treadmill images are only captured before and immediately after exertion. The Bruce protocol remains widely used for treadmill tests to this day, whereas on cycle or reclining ergometers, the Balke protocol is preferred, with load increased gradually (in 25W increments), as shown in the figure every 2 minutes,^2^ and may go beyond 125 W.

## 5. Dobutamine Stress Echocardiography in the Assessment of Myocardial Ischemia

In myocardial ischemia, the basis of pharmacological stress echocardiography with dobutamine (DSE) is the evaluation of the physiological response of the myocardium to different degrees of coronary artery obstruction, especially in terms of subendocardial perfusion, while the subepicardial layer is only affected at a later stage if ischemia persists.^35,36^

Indication. DSE is an alternative method for evaluating myocardial ischemia when a patient is unable to perform physical exercise. DSE was initially developed for assessment of patients with known or suspected coronary artery disease.^37^ Its most common indication is to assess the presence, location, or extent of myocardial ischemia. Its high specificity (88%) yields a high negative predictive value for myocardial infarction and cardiac death.^38^ Indications for DSE as per the latest guidelines are listed in Table 5.1.^39^

**Table 5.1:** Indications for dobutamine stress echocardiography in myocardial ischemia^3,12,35-43^

1) Diagnosis of CAD with intermediate pretest probability.
2) Acute chest pain in intermediate-risk patients (no abnormalities on ECG and negative biomarkers).
3) Symptomatic angina in patients with a history of stent or CABG.
4) Moderate coronary obstruction detected by CT angiography or coronary angiography: functional significance.
5) Risk stratification after myocardial infarction.
6) Etiologic diagnosis of dyspnea.

DSE may not detect ischemia of small distal vessels, and patients with obesity or emphysema may have poor acoustic windows, resulting in suboptimal images even with the use of ultrasound enhancement or ultrasound contrast agents.

The selection of the most appropriate examination is based on the patient's clinical picture, including the nature of the patient's symptoms, their risk profile, and the strengths and limitations of the chosen test modality.^40^

Equipment. Some equipment and drugs must be present in the echo lab for DSE (Table 5.2).^39^

**Table 5.2:** Equipment and medications required for performing pharmacological stress echocardiography with dobutamine ^3,12,35-43^

*1) Digital echocardiography system with stress protocol and ability to display images simultaneously in quad-screen format.*
2) Device for electrocardiographic and blood pressure monitoring (preferably continuous).
3) Crash cart (including drugs and supplies for cardiopulmonary resuscitation and a defibrillator/cardioverter).
4) Oxygen supply (if necessary).
5) Equipment for intubation.
6) Dobutamine infusion pump.
7) Drugs for the management of allergic reactions, in addition to dobutamine, atropine, and metoprolol (and other drugs and their antidotes, if a different pharmacological protocol is used).

### 5.1. Dobutamine Stress Echocardiography Protocol

Defined in 2007 by the American Society of Echocardiography guidelines.^12^

Patient preparation: Patients should fast for 4 to 6 hours before the test and may continue to take their usual medications. However, the following should be discontinued at least 24 hours before the test: amiodarone, calcium channel blockers, nitrates, substances containing caffeine and nicotine (for dipyridamole stress echocardiography in particular), and, most importantly, beta blockers (to avoid a false-negative result).^37^ The latter should be discontinued ideally 3 to 4 days before the examination, if the goal is to diagnose myocardial ischemia.

Stages of the procedure: 1) Placement of IV access; 2) Placement of 12-lead electrodes; 3) Placement of sphygmomanometer; 4) Focused resting echocardiogram, for later comparison with images obtained under stress; 5) Image acquisition at rest, low-dose, intermediate-dose (optional), peak of dobutamine effect and recovery phase, with cine loops to be archived of the main views: long axis, short axis (at the level of the papillary muscles), apical 4-chamber, apical 2-chamber and, optionally, apical 3-chamber (adds value for evaluation of the LV apex or as a replacement for the parasternal long-axis view). Some authors choose to acquire all images through the apical window (apical 4-, 3-, and 2-chamber views). The key aspect is to observe all walls of the left ventricle irrigated by the main coronary vessels; 6) Continuous ECG, heart rate (HR), and blood pressure (BP) monitoring; 7) If necessary, isometric exercise (handgrip) will be performed in conjunction; atropine infusion if necessary, to enhance the examination; metoprolol infusion if necessary, to antagonize the effects of dobutamine (when necessary); 8) Report of examination.

Dobutamine Action and Pharmacokinetics: Dobutamine exhibits a strong adrenergic agonist effect, acting on beta1 receptors to increase heart rate and contractility and on beta2 receptors to cause peripheral vasodilation. Its action begins 1 to 2 minutes after infusion, and it has a half-life of 2 minutes. Dobutamine is the most commonly used agent for pharmacological SE due to its high sensitivity and controllable side effect profile.^37^ It is metabolized in the liver and peripheral tissues, but there is no need for dose reduction in patients with renal or hepatic impairment. It is usually administered in escalating doses, starting at 5 mcg/kg/min and increasing at 3-minute intervals up to 10, 20, 30, and 40 mcg/kg/min. Dobutamine may be started at a low dose of 2.5 mcg/kg/min and titrated to a dose of 7.5 mcg/kg/min to facilitate the recognition of myocardial viability in segments that are already abnormal (akinetic) at rest.

A conclusive test requires reaching the target heart rate (85% of the maximum heart rate predicted for age). However, the development of new contractility abnormalities or worsening of preexisting abnormalities should prompt termination of the test due to ischemia. Additionally, significant arrhythmias, severe arterial hypertension, and intolerable symptoms may also lead to test interruption. When the target heart rate cannot be achieved with dobutamine alone, maximal isometric exercise and/or atropine may be added to increase the sensitivity of the test, particularly in patients who are on beta blockers, calcium channel blockers, or nitrates, as well as in those with single-vessel disease.^3,41,42^ Care should be taken when classifying a test as "submaximal" when the patient reaches 85% of the predicted maximum heart rate for age. This level corresponds to the target heart rate (i.e., the submaximal heart rate, 85% of maximum) and is therefore sufficient to characterize the test as conclusive, rather than submaximal or inconclusive.

Atropine: administered in doses of 0.25 to 0.5 mg at intervals of 30 seconds to 1 minute as needed to achieve the target heart rate, up to a total dose of 2 mg. The 0.25 mg dose is suggested for older adults, patients with a small body surface area, patients with neurological disorders or those who are already near the target HR, to avoid untoward effects. Administering atropine at the lowest dose of dobutamine (20 or 30 mcg/kg/min) facilitates earlier achievement, with fewer side effects, a lower cumulative dose of dobutamine, and a shorter test duration. Discontinuation of beta-blockers facilitates reaching the target HR, and inadequate discontinuation may lead to episodes of hypertension or arrhythmia ^3,41^ at peak dobutamine effect, due to sudden loss of beta-adrenoceptor blockade.

Beta blockers: can be administered to reverse the effects of dobutamine (their main purpose in this setting), but can also be used to increase the sensitivity of the test for patients with single-vessel disease when administered at peak dobutamine stress.^42^

Patients may develop minor or more significant arrhythmias, such as atrial fibrillation and/or non-sustained ventricular tachycardia, which usually resolve after discontinuation of the dobutamine infusion; in such cases, beta blockers should be administered. If sustained ventricular tachycardia occurs, one should first consider myocardial ischemia.^3^

Drugs used in DSE, their doses and side effects are listed in Table 5.3.

**Table 5.3:** Intravenous drugs used during stress echocardiography, their adverse effects, and contraindications^3,12,35-43^

Dobutamine:	Dosage: 5 to 40 mcg/kg/min, increased gradually every 3 minutes. Adverse effects: nausea, tremor, palpitations, headache, hypotension, hypertension, chest pain, arrhythmia. Contraindications are listed in Table 5.4.
Atropine:	Dose: up to 2 mg, starting at a dose of 20 or 30 mcg of dobutamine (early protocol), unless test cessation is indicated. Adverse effects: tachycardia, palpitations, dry mouth, mydriasis, nausea, agitation, urinary retention, increased intraocular pressure. Contraindications: closed-angle glaucoma, prostate hypertrophy, urinary retention.
Metoprolol	Dose: 1 to 10 mg. Adverse effects: bradycardia, hypotension, dizziness, headache. Contraindications: bronchial asthma, severe lung disease, tachy-brady syndrome, AV block.

Absolute and relative contraindications: the contraindications to all modalities of stress testing are summarized in Table 5.4.^3^

**Table 5.4:** Contraindications to dobutamine stress echocardiography in myocardial ischemia^38,39,43^

**Absolute contraindications**
1) Acute MI in last 3-5 days
2) Unstable angina
3) Uncontrolled, symptomatic arrhythmia with hemodynamic instability
4) Severe, symptomatic aortic stenosis
5) Acute myocarditis or pericarditis
6) Severe hypertension (BP > 200/110 mm Hg at rest)
7) Active endocarditis
8) Pulmonary embolism or pulmonary infarction
9) Obstructive hypertrophic cardiomyopathy
10) Recent or mobile ventricular thrombus
**Relative contraindications**
1) Hyperthyroidism, severe anemia, recent stroke
2) Intracranial or thoracic aortic aneurysm (>40 mm).
3) Moderate aortic stenosis

Informed consent: Must be signed by all patients before the test.

Image acquisition: The echocardiographer should ensure that all images are obtained on the same plane and at the same depth throughout the examination, for more reliable comparison in quad-screen format.^38^ Adjustments should be made while obtaining the baseline resting images and maintained throughout the examination.

### 5.2. Type of Myocardial Response

A normal DSE is defined by the presence of normal regional and global wall motion at rest, regional and global hyperkinesis at stress, and a significant reduction in LV chamber size due to reduced preload and afterload (physiological left ventricular cavity obliteration) (Figure 5.1).

**Figure 5.1:** Dobutamine stress echocardiography, 2D echocardiography – normal left ventricular response: reduction of the left ventricular cavity and hyperkinesis of all walls.

An ischemic response is defined by the development of a new or worsening wall motion abnormality during stress, affecting at least one myocardial segment (Figure 5.2).

**Figure 5.2:** Dobutamine stress echocardiography, 2D echocardiography – Lateral ischemia and septum with dilatation of the left ventricle (LV).

Myocardial ischemia can be caused by epicardial vessel disease or by other causes, such as microvascular disease, a hypertensive response to exercise, or underlying cardiomyopathy.

A severe resting wall motion abnormality, such as akinesis, which remains unchanged with stress is considered a "fixed response" and represents a transmurally infarcted region or one with a limited epicardial rim of viability. It should be noted that akinesis becoming dyskinesis is considered to represent a mechanical response of an infarcted segment or increased intraventricular pressure, not ischemia.

The presence of any aneurysm should be reported, due to its prognostic and therapeutic implications.^3^

The differences in regional and global wall motion responses to DSE stress are listed in Table 5.5.

**Table 5.5:** Types of response to dobutamine stress echocardiography as compared to rest and low-dose states

**Normal regional response:** Hyperkinesis and increased contractile force.
**Ischemic regional response:** Hypokinesis and reduced contractile force.
**Normal global response:** decrease in end-diastolic and end-systolic volume and a marked increase in ejection fraction.
**Ischemic global response:** decrease in ejection fraction and ventricular cavity dilatation.

Cessation of DSE: DSE should be ceased: 1- when the target heart rate is reached; 2- if regional wall motion abnormalities are detected in at least 2 segments; 3- upon onset of test-limiting symptoms or any complex arrhythmia; 4- upon onset of any hypotension or significant hypertension (SBP ≥ 220-240 mm Hg and/or DBP ≥ 120 mm Hg); 5- at the end of the protocol. The use of ultrasound enhancing agents or contrast should be considered to improve endocardial definition when two or more contiguous segments are poorly visualized at rest on apical view.

The adverse effects most commonly observed during DSE are a feeling of warmth, nausea, chills, urinary urgency, palpitations (secondary to tachycardia) and, rarely, mild headache.^3^ Severe headache in response to dobutamine should prompt suspicion of hypertension.

Test interpretations and conclusions in myocardial ischemia are listed in Table 5.6.^3^

**Table 5.6:** Interpretation of segmental myocardial thickening in the results of pharmacological stress echocardiography with dobutamine for the detection of myocardial ischemia

1. Rest = normal Stress = hyperkinesis Conclusion = normal
2. Rest = normal or hypokinesis Stress = hypokinesis or akinesis Conclusion = ischemia
3. Rest = akinesis Stress = improves at low dose and worsens at high dose Conclusion = viability and ischemia
4. Rest = akinesis Stress = akinesis or dyskinesis Conclusion = scarring/fibrosis

Test endpoints: Absolute and relative endpoints for DSE are listed in Table 5.7.

**Table 5.7:** Absolute and relative endpoints for stress echocardiography

1- Reached 85% of the maximum heart rate (HR) predicted for age (220-age). 2- Maximum drug dose administered (end of protocol) or limiting symptoms. 3- Echocardiographic evidence of myocardial ischemia (echo positivity), regardless of any other factor. 4- Sustained ventricular tachycardia/complex arrhythmias. 5- Symptomatic decline in blood pressure (SBP > 20 mm Hg) or heart rate.
Relative endpoints for stress echocardiography: 1- New or worsening regional wall motion abnormality in at least one previously unaffected segment or in any segments already abnormal at rest. 2- Left ventricular dilatation. 3- Onset of arrhythmias (atrial fibrillation, supraventricular tachycardia, complex ventricular arrhythmia).

Accuracy of the method – Several studies have demonstrated excellent accuracy of DSE, using coronary arteriography as the gold standard for comparison. For detection of coronary artery disease (CAD), DSE has sensitivity similar to that of tomographic nuclear perfusion imaging, with higher specificity. DSE has greater sensitivity for the detection of lesions in the left anterior descending artery and in LMCA or multivessel disease.^3^

If ischemia is not detected and stress is inadequate, either due to limited acoustic window or failure to achieve the target heart rate, the test result may be false negative.

Patients with false-positive stress echocardiogram results have similar outcomes to those with true-positive results. They should receive intensive-risk factor management and careful follow-up.^3^

Overall interpretation – The report should include one of the following as a conclusion:

Normal, ischemia, absence of wall thickening (fibrosis), or a combination of these findings (provided the appropriate endpoint is reached). If the end of the protocol was reached uneventfully but the target heart rate was not achieved, the test can be described as inconclusive (or negative up to the heart rate actually reached). In the latter case, it is important to stress that the sensitivity of the test for diagnosing myocardial ischemia is reduced in such cases because the target heart rate was not reached.

For image interpretation, the size, shape, and function of the LV are compared at each stage in 4 simultaneous views and in each view in simultaneous stages. These images must be obtained from the same position at each stage of the test. In the normal response to stress, the LV is smaller compared to its resting dimension, its shape is maintained, and endocardial excursion increases, with a consequent increase in myocardial thickness.^43^

Left ventricular dilatation and reduced LV ejection fraction during DSE generally indicate extensive ischemia.

Each segment is scored as follows:

1. Normal wall motion (myocardial thickening > 50%);
2. Hypokinesis (myocardial thickening 10%–50%);
3. Severe hypokinesis or akinesis (myocardial thickening < 10%);
4. Dyskinesis (paradoxical septal motion and systolic bulging).

The wall motion score index (WMSI) is calculated by dividing the sum of all segment scores by the total number of myocardial segments (16 or 17, if including the LV apex). In normal, non-ischemic myocardium, the WMSI is 1.^39^ The echocardiographic views to be obtained and the corresponding coronary perfusion are shown in Table 5.8.

**Table 5.8:** Echocardiographic assessment of the segments of the heart and coronary irrigation territories

VIEW	CORONARY SUPPLY
Parasternal long-axis or apical 3C	LAD, RCA and CX
Apical 4C	LAD, RCA and CX
Apical 2C	LAD, RCA
Parasternal short-axis view	LAD, RCA and CX

LAD: left anterior descending artery; RCA: right coronary artery; CX: circumflex artery.

Difficulty in interpreting test results: patients with pacemakers may have an inadequate rate response and may also exhibit regional wall motion abnormalities due to the stimulation effect; therefore, dobutamine often fails to elicit adequate responses in these patients.

Risk stratification and prognosis: DSE is generally used for the diagnosis of CAD with ischemia, assessment of the risk of cardiac events, and prognosis. Risk stratification with normal DSE is associated with prognosis and compares favorably with that after normal myocardial SPECT imaging (thallium-201, technetium-99, or sestamibi).

Compared to patients undergoing ESE, those undergoing DSE are generally older and have more comorbidities; wall motion and ejection fraction were identified on multivariate analysis as the best predictors of cardiac events.^3^

Reporting DSE results: the report should contain the data listed in Table 5.9.^38^ Particular attention should be paid to the basal inferior and apical segments, which must be assessed on multiple images to prevent a false-positive finding of ischemia.

**Table 5.9:** Recommendations for reporting results of dobutamine stress echocardiography^38,39,43^

Clinical information and indication for examination.
**Rest:** Regional wall motion assessment.
Wall motion: Number, location, and severity of regional (or global) abnormalities.
2- Estimation of the ejection fraction.
**Stress:** Regional wall motion assessment.
1- Wall motion: Number, location, and severity of regional (or global) abnormalities. 2- Estimation of the ejection fraction.
3- Assessment of regional wall motion should be repeated as many times as necessary.
4- If an ultrasound enhancement agent (contrast agent) is used, report the agent and dose administered.
Data on the stress testing protocol:
1- Doses of agent(s) for pharmacological stress.
2- Whether the target HR was achieved. HR and BP at each stage of the protocol.
3- Clinical and ECG findings during the examination, at rest and during stress.
4- Post-test echocardiographic findings.
5- Reason for test cessation (endpoint of protocol, ischemia, symptoms, or complications).

### 5.3. Assessment During DSE and in Recovery

The development of regional wall motion abnormalities during the early stages of stress is indicative of severe coronary artery obstruction, with little or no myocardial perfusion reserve. At low doses, in the presence of moderate coronary obstruction with some myocardial reserve still preserved, increased blood flow leads to increased myocardial thickening and wall motion. At high doses, the combination of tachycardia and coronary obstruction results in a marked decline in subendocardial flow, with a corresponding decrease in regional wall motion.

The persistence of wall motion abnormalities during the recovery period may be due to myocardial stunning, and is indicative of more severe ischemia. Late ischemic recovery imaging should be considered in patients with severe and extensive stress-induced. Most patients who present with abnormal DSE findings and subsequently undergo coronary angiography are found to have obstructive coronary artery disease.^3^

Extent and severity of regional wall motion abnormalities:

Stress-induced hypoperfusion reflects the area of myocardium at risk, and its severity reflects the magnitude of wall motion abnormalities, which are exponentially correlated with increased adverse cardiovascular events.^3^

### 5.4. Transient Ischemic Dilatation of the Left Ventricle

Patients with abnormal stress echocardiograms and transient ischemic dilatation of the LV have higher rates of multivessel disease and adverse cardiovascular events.

Left bundle branch block (LBBB) - DSE can be used for detection of ischemia and risk stratification in patients with LBBB^3^ (Figure 5.3).

**Figure 5.3:** Dobutamine stress echocardiography, 2D echocardiography – left bundle branch block – Long axis: at rest – normal septum. Peak stress – slightly anomalous septum; immediate post-stress – highly anomalous septum.

In a study of patients with LBBB who underwent both DSE and cine coronary angiography, Yanik et al.^44^ observed good sensitivity, specificity, and accuracy of DSE for identifying ischemia: 82%, 95%, and 90% respectively in the left anterior descending (LAD) artery territory and 88%, 96%, and 93% in the circumflex and right coronary artery territories. Mairesse et al.,^45^ in a similar study using DSE, myocardial perfusion imaging, and coronary angiography, found that DSE performed well in detecting ischemia in the LAD territory, with a sensitivity of 83%, specificity of 92%, and accuracy of 87%.

Older adults – DSE is the first-line SE modality of choice for older adults, as well as for patients with musculoskeletal or neurological conditions that preclude exercise stress testing.^46^ If the desired HR is not reached, isometric exercise to half-maximal strength for 3 to 4 minutes is recommended,^47^ the patient should keep their mouth open and abdomen relaxed, to prevent an inadvertent vagal maneuver.

### 5.5. Comparison with Other Imaging Modalities

Large studies and meta-analyses have compared the predictive value of DSE and SPECT in different patient populations. The PROMISE study compared anatomical imaging using CT angiography versus functional imaging in 10,003 stable patients with symptoms suggestive of CAD; however, stress SPECT or DSE was performed in only 1,083 patients. None of these studies comparing the value of DSE versus other modalities considered the incremental value of ancillary findings detected by echocardiographic imaging performed at the time of stress echocardiography, including detection of non-ischemic causes of cardiac symptoms.^3^

DSE has diagnostic and prognostic accuracy similar to that of radionuclide stress perfusion imaging or cardiac magnetic resonance imaging.

### 5.6. Value of a Negative DSE

Samiei et al.^48^ studied 705 patients with no prior history of CAD who had a negative ESE or DSE. They found that those who experienced events were older, diabetic, or hypertensive, and that results should be interpreted with greater caution due to the lower negative predictive value of DSE in these patients. The accuracy of diagnosis can also be significantly affected by the use of beta blockers, resulting in a false negative test.^43,49^

### 5.7. Complications of DSE

According to a meta-analysis of 26 studies, complications occur in < 0.1% of DSEs. The most common is arrhythmia, associated with significant ischemia and EF < 35%. Atrial fibrillation and nonsustained ventricular arrhythmias occur in approximately 3% of patients. Frequent premature atrial or ventricular contractions occur in about 10% of cases. Sustained ventricular tachycardia is uncommon.^43^ Angina and/or hypotension can occur due to midventricular obstruction or left ventricular outflow tract obstruction (the latter may lead to chest pain and arterial hypotension). Despite its safety, approximately 50% of patients undergoing DSE report some adverse reaction, with nausea, flushing, headache, neck or chest tightness, paresthesia, urinary urgency, dyspnea, and dry mouth being most common.^38^

Figure 5.4 shows a dobutamine stress echocardiogram, incorporating three-dimensional imaging, positive for inducible myocardial ischemia in the septal region.

**Figure 5.4:** 3D echocardiography – Short axis: at rest – normal septum. 3D echocardiography – Short axis: peak stress – akinetic septum.

## 6. Dipyridamole Stress Echocardiography

Dipyridamole is a pharmacological myocardial stressor that causes a buildup of endogenous adenosine, which then stimulates A2A receptors in the coronary arterioles.^50^ It causes a 4- to 5-fold increase in coronary and myocardial blood flow from baseline without significantly increasing the heart rate or systolic volume, while simultaneously reducing systolic blood pressure.^3^ Thus, in the presence of critical epicardial stenosis or microcirculatory dysfunction, administration of dipyridamole leads to heterogeneity in blood flow between stenotic segments and normal coronary arteries.^3^ The resulting mismatch in supply and demand leads to a reduction in subendocardial blood flow in regions supplied by stenosed arteries, a phenomenon known as coronary steal.^51^

The indications, types of equipment required, patient preparation, stages of the procedure, image acquisition, informed consent, and types of myocardial response are similar to those described above for dobutamine.

### 6.1. Protocols

There are 2 standard protocols for administering dipyridamole:

1. **Two-infusion protocol:**
  Intravenous infusion at 0.84 mg/kg over 10 min, in two separate infusions: 0.56 mg/kg over 4 min, followed by 4 min of observation with no medication; if the test is still negative, an additional 0.28 mg/kg of dipyridamole is administered over 2 minutes. If no endpoint (in this case, ischemia) is reached, 0.25 mg of atropine is administered every minute to a maximum total dose of 1 mg (Figure 6.1).
2. **Accelerated protocol:**
  Intravenous infusion at 0.84 mg/kg over 6 minutes, followed by a 4-minute observation period with no medication; administration of atropine at the end of the test is not necessary (Figure 6.2).
  Aminophylline - With both protocols, aminophylline should be available for immediate administration in case of any dipyridamole-related adverse event and should be routinely infused at the end of the test, regardless of outcome,^5,50^ due to the prolonged half-life of dipyridamole.
  Mechanism of Action and Pharmacokinetics: Dipyridamole works by inhibiting reuptake of endogenous adenosine. The alpha half-life of dipyridamole (i.e., the initial decline after peak concentration) is approximately 30 to 45 minutes. The beta half-life (the terminal decline in plasma concentration) is approximately 10 hours. Dipyridamole is metabolized in the liver by glucuronidation and excreted in bile.^52,53^ Due to its vasodilating effect, it may cause a precipitous decline in blood pressure and a slight increase in heart rate.

**Figure 6.1:** Standard (stepwise) high-dose dipyridamole + atropine protocol.

**Figure 6.2:** Accelerated high-dose dipyridamole protocol.

### 6.2. Adverse Effects

The major adverse effects of dipyridamole include onset of atrioventricular (AV) block, potentially progressing to complete heart block, although to a lesser degree than that observed with adenosine (2%); chest pain (in about 20% of patients), which is nonspecific and not necessarily indicative of the presence of CAD; and minor side effects including headache, dizziness, nausea, hypotension, and flushing.^52^

### 6.3. Contraindications

The main contraindications to dipyridamole stress echocardiography (Table 6.1) include: bronchospastic lung disease with continuous wheezing or a history of significant reactive airway disease, uncontrolled hypertension (SBP > 200 mm Hg or DBP > 110 mm Hg), hypotension (SBP < 90 mm Hg), known hypersensitivity to dipyridamole, caffeine intake in the last 24 hours, and second- or third-degree AV block without a functioning pacemaker.^52^ As with other SE protocols, the presence of unstable angina or complicated acute coronary syndrome, complex cardiac arrhythmias, symptomatic severe aortic stenosis, and hypertrophic cardiomyopathy with LVOTO are also absolute contraindications.^3^

**Table 6.1:** Contraindications to dipyridamole stress echocardiography

Contraindications applicable to all protocols	Contraindications specific to dipyridamole stress echo
Unstable angina	Bronchospastic lung disease with continuous wheezing
Acute or complicated coronary syndrome	History of significant reactive airway disease
Complex cardiac arrhythmia Decompensated heart failure	Second- or third-degree AV block without a functioning pacemaker
Severe, symptomatic aortic stenosis	SBP < 90 mm Hg
Hypertrophic cardiomyopathy and left ventricular outflow tract obstruction	Known hypersensitivity to dipyridamole
	Caffeine intake in the last 24 hours

### 6.4. Coronary Flow Reserve

Pharmacological stress with dipyridamole is the safest, simplest, and most suitable method for combined assessment of coronary flow reserve and regional wall motion/myocardial thickening.^50^ However, individual patient characteristics, the local cost of the medication, and examiner preference should be taken into consideration when choosing one modality over another. For example, in patients with severe hypertension and/or a history of significant atrial or ventricular arrhythmias, dipyridamole may be the more appropriate choice.

### 6.5. Accuracy

Pharmacological stress testing with dipyridamole (accelerated protocol or standard protocol with atropine), when compared to dobutamine/atropine, has high diagnostic accuracy, similar to that of maximal treadmill exercise stress echocardiography, with lower sensitivity and higher specificity. Furthermore, dipyridamole/atropine stress echocardiography can overcome the effects of concomitant beta blocker therapy, achieving high heart rates and comparable diagnostic value.^54^ Nevertheless, due to their cardioprotective effect, when beta-blockers are not discontinued before the examination, they may also lead to a false-negative result.

Several meta-analyses have compared the accuracy of perfusion imaging to that of stress echocardiography.^3^ The two have been found similarly sensitive for detecting CAD, but stress echocardiography has greater specificity. For the detection of LMCA disease or multivessel CAD, stress echocardiography showed greater sensitivity compared to nuclear myocardial perfusion imaging, which compares relative differences in perfusion and may thus miss balanced or global ischemia.^3,55-57^

## 7. Adenosine or Regadenoson Stress Echocardiography

Adenosine is a purine present in all body tissues,^58,59^ derived from adenine nucleotides and degradation of ATP. It is metabolized just as easily as it is generated, making its levels highly variable.^58,60,61^ Its action is mediated through four transmembrane receptors: the A1, A2A, A2B, and A3 adenosine receptors.^58,59,62^ All are G protein-coupled receptors (GPCRs),^59^ with A1 and A3 receptors binding to inhibitory G proteins and A2 receptors binding to stimulatory G proteins.^59-61^ This means they recruit distinct pathways through adenosine binding.^58-73^ The adenosine receptors can thus be divided into two main subtypes: A1 and A3 receptors, which inhibit the enzyme adenylate cyclase, and A2 receptors, which stimulate adenylate cyclase. A1 receptors predominate in the myocardium, while A2 receptors are found in the coronary arteries (endothelial and smooth muscle cells). Therefore, activation of A2A receptors dilates the coronary arteries, causing hyperemia and increasing coronary blood load. Activation of A2A receptors in the presence of critical coronary stenosis plays a key role in inappropriate vasodilation and subendocardial ischemia, leading to vertical and horizontal steal phenomena and regional wall motion abnormalities. These effects are also present in a dipyridamole stress test.

Intravenous infusion of adenosine or an intracoronary bolus of adenosine induces maximum flow (hyperemia). Under physiological conditions, the epicardial coronary artery exhibits laminar flow and little or no pressure gradient from proximal to distal. Coronary stenosis induces flow acceleration at the site of obstruction and can decrease pressure distal to lesions at rest, limiting hyperemia flow acceleration and causing an additional decrease in post-stenotic pressure during stress.^22^

Under normal physiological conditions, myocardial perfusion is autoregulated by pre-arterioles in the epicardium and arterioles within the myocardium, such that microvascular tone contributes to the majority of coronary resistance. Pathological microvascular conditions, including perivascular fibrosis (hypertension,^64^ coronary artery disease,^65^ myocardial hypertrophy, diabetes mellitus, status post heart transplantation, etc.), can cause microvascular angina by increasing microvascular tone at rest and/or limiting vasodilation during stress.

Therefore, the use of vasodilators to induce stress seems justified in non-invasive functional tests. To optimize the ischemic effect and assess changes in regional wall motion, higher doses are required than those needed for perfusion imaging. Therefore, the protocols used in the SE laboratory must adopt higher and faster doses of vasodilator compared to perfusion imaging, resulting in distinct protocols.

The cardiovascular effects of exogenous adenosine administered intravenously in humans are vagal inhibition and increased heart rate at low doses, while at high doses it can cause inhibition of the sinoatrial node and atrioventricular conduction, bradycardia, atrioventricular block, sympathetic stimulation, vasodilation in all arteriolar beds (except the renal pre-glomerular arterioles, where it promotes vasoconstriction), and hyperventilation, which is explained by interaction with carotid chemoreceptors.

### 7.1. Use During Stress Echocardiography

Intravenous infusion of adenosine causes a slight increase in heart rate and cardiac output and a slight decrease in systemic pressure (similar to dipyridamole). Mild tachycardia occurs despite the direct negative chronotropic and dromotropic effects of adenosine due to stimulation of myocardial A1 receptors, either by direct stimulation of sympathetic excitatory arterial chemoreceptors^67^ or, indirectly, through systemic vasodilation (Figure 7.1).

**Figure 7.1:** The use of adenosine in stress echocardiography demonstrates the major contribution of the vasodilatory effect in increasing coronary flow reserve (CFR), with a progressive, significant increase during the examination seen after 1 minute 23 seconds of adenosine infusion. The CFR increased from 16 cm/s at rest to 42 cm/s.

In normal individuals, coronary blood flow increases four to five times from baseline after administration of adenosine, an increase comparable to that caused by high-dose of dipyridamole and substantially greater than that induced by exercise or dobutamine, during which coronary blood flow increases about three times the baseline value.^67^ The maximum coronary dilating effect is achieved within 2 minutes following adenosine administration and wears off rapidly, within 2–5 minutes after the infusion is stopped.^68^

Regadenoson theoretically has attractive features which make it close to the "ideal" agent: rapid onset of action, adequate duration of effect that allows image acquisition, and fewer side effects. The reason for this is its selective stimulation of the A2A receptors specifically responsible for coronary vasodilation, avoiding the undesirable effects of stimulation of A1, A2B, and A3 receptors (such as dyspnea, headache, and flushing). The incidence of these side effects is not substantially reduced when compared to that seen with adenosine, although their severity is decreased.^69^

#### 7.1.1. Indications

The primary indication for adenosine/regadenoson stress testing is the detection of both epicardial and microvascular coronary artery disease causing myocardial ischemia. Like dipyridamole, it can also be used to assess myocardial viability, but with lower reproducibility compared to dobutamine.

The longstanding safety record and short half-life of adenosine make it especially suitable for patients with severe aortic stenosis,^70,71^ in the postoperative period of asymptomatic children who have undergone arterial switch surgery for transposition of the great arteries,^72^ or older adults,^73^ who may be especially vulnerable to complications during dipyridamole or dobutamine stress.

One possibly emerging application of regadenoson is in patients with moderate-to-severe chronic obstructive pulmonary disease who are candidates for stress imaging and wish to avoid adenosine-induced bronchoconstriction and resulting respiratory compromise,^3,74,75^ although in these patients the use of dobutamine may be more reasonable.

### 7.2. Protocol for Adenosine Stress Echocardiography

A dose of 140 μg/kg/minute infused over 6 minutes is routinely recommended for myocardial ischemia. Increasing the dose to 160 and 210 μg/kg/minute may be advised if the test is negative at the 140 μg/kg/minute step; this is only necessary in 16–18% of cases.^68,76,77^ When side effects are intolerable, the dose may also be titrated down. Atropine or handgrip exercise can be used to potentiate the test. As is routine in any stress test, continuous ECG, blood pressure, and, in this case, pulse oximetry monitoring is mandatory.

Standardized images targeting all coronary territories (apical 4-chamber, 2-chamber, and 3-chamber views or parasternal long-axis, transverse LV, apical 4-chamber and 2-chamber views) should be acquired at rest, in an intermediate stage at 2 minutes of infusion, before the end of the infusion, and during the recovery phase.

To assess coronary flow reserve in the LAD, the maximum diastolic velocity of mid-distal flow is recorded at rest, during maximal hyperemia (which typically occurs within 90 to 120 seconds of infusion), and before the end of the infusion.

A dose of 100 μg/kg/minute infused over 2 minutes can be used for the assessment of myocardial viability.

### 7.3. Protocol for Regadenoson Stress Echocardiography

Currently, regadenoson is administered as a 0.4 mg bolus in 5 mL of solution (without weight-based dose adjustment) injected over 10 seconds, followed by a 5 mL saline flush to ensure proper drug delivery. The optimal time for acquiring perfusion images is 2 to 10 minutes after drug infusion. Regadenoson allows combined assessment of myocardial perfusion imaging and regional wall motion analysis, and is most commonly used in the United States and the United Kingdom. Atropine can be used as an adjunct to potentiate stress.^78^

### 7.4. Contraindications

Absolute contraindications include hypotension and severe airway obstruction, active bronchospasm, and second- or third-degree AV block (unless protected by a pacemaker). Use of these agents should be avoided in patients with signs or symptoms of unstable angina or cardiovascular instability.^75^

Patient preparation before stress echocardiography: Antianginal drugs and xanthine-containing products – including beverages such as coffee, teas, yerba mate, chocolate, soft drinks, and even foods such as bananas – decrease the sensitivity of adenosine SE, and should therefore be avoided starting 24 hours before the stresstest.

### 7.5. Adverse Effects

Adverse effects are not infrequent and can be limiting in a significant number of patients – up to 20%.^79^ The most frequent limiting adverse effects of adenosine-mediated vasodilator stressors include shortness of breath, flushing, headache, hypotension, intolerable chest pain (sometimes unrelated to underlying ischemia, possibly induced by direct stimulation of myocardial A1 adenosine receptors), and high-degree AV block. First- or second-degree AV block occurs in 2% of tests, hypotension below 90 mm Hg in 3%, and severe bronchospasm in 0.1%.^80,81^ Minor side effects, such as a feeling of warmth, flushing, headache, and nausea, occur in 80–84% of tests.^81^ Hemodynamically significant decrease in systolic blood pressure (> 20 mm Hg) is more likely in patients with severe aortic stenosis.^82^ Exogenous adenosine has an even more pronounced negative chronotropic and dromotropic effect than endogenous adenosine,^83^ making the onset of advanced AV blocks more frequent with adenosine than with dipyridamole or adenosine triphosphate at equivalent doses. Most reactions to adenosine resolve within 1 to 5 minutes. On very rare occasions, aminophylline infusion is required.

### 7.6. Diagnostic Accuracy

Based on a published meta-analysis of 11 studies, adenosine stress echocardiography had the same sensitivity (79%), specificity (91.5%), and accuracy for detection of regional wall motion abnormalities as exercise stress echocardiography, dipyridamole echocardiography, and dobutamine echocardiography, with superior specificity compared to SPECT stress imaging.^84^

## 8. Other Stress Echocardiography Modalities: Pacing, Ergonovine, Handgrip, and Hyperventilation

### 8.1. Pacing Stress Echocardiography

In patients with contraindications to physical or pharmacological stress, pacing stress echocardiography (PASE) by transesophageal atrial pacing may be an alternative. Stimulation is performed by an external pacemaker connected to a bipolar esophageal electrode and a recording catheter protected by a 10F sheath, introduced transnasally or transorally under topical anesthesia. The lowest current capable of providing stable capture is used, starting at 10 bpm above the resting heart rate and increasing by 20 bpm every 2 minutes up to 85% of the predicted maximum heart rate for age.^85^ he criteria for test cessation are intolerable symptoms, significant arrhythmia, ECG with ST segment depression ≥ 2 mm at 80 ms after the J point, SBP > 220 mm Hg, DBP > 110 mm Hg, or SBP < 90 mm Hg. Serious adverse events are rare. Second-degree Wenckebach-type AV block may occur. Compared to dobutamine stress, PASE was associated with less hypotension or hypertension, arrhythmias, or limiting symptoms; shorter duration and recovery times; and more frequent achievement of the target heart rate. There was excellent agreement between the two methods for assessment of regional wall motion.^85^ In patients with a permanent pacemaker in place, stress can be achieved through noninvasive programming.^86^ The target is 85% of the maximum heart rate or until signs of ischemia develop. PASE demonstrated a sensitivity of 70%, specificity of 90%, and accuracy of 78% for diagnosis of obstructive CAD, and was an independent predictor of all-cause mortality.

### 8.2. Addition of Handgrip Exercise to Stress Echocardiography

Isometric handgrip exercise produces a slight increase in HR and oxygen consumption compared to isotonic exercise. However, it produces a sudden and more significant increase in blood pressure. Using a hand dynamometer, a submaximal protocol (25–50% of maximum grip strength) can be maintained for 3-4 minutes.^87^ Isometric exercise has low sensitivity on its own for investigation of obstructive CAD, but is useful when combined with pharmacological stress.^88^ When combined with longitudinal strain assessment, it showed a sensitivity of 80%, specificity of 66%, and AUC of 0.77 for diagnosis of myocardial ischemia.^89^ It has also been used as an alternative to the cycle ergometer for diastolic stress testing for diagnosis of HFpEF. It also has an advantage over the cycle ergometer regarding motion artifacts. A submaximal test with 40% of maximum handgrip for 3–5 minutes produces O_2_ consumption similar to low-load (20 W) cycle ergometer exercise, with a comparable increase in E/e’ ratio.^90^

### 8.3. Ergonovine and Hyperventilation Stress Echocardiography

Ergonovine has been used for vasoconstrictive stress testing. The protocol consists of administration of an intravenous bolus (0.025 to 0.05 mg) every 5 minutes until evidence of ischemia is present or a total dose of 0.35 mg is reached. Its main indication is the diagnosis of vasospastic angina, with a sensitivity of around 91% and a specificity of around 88%. In a study of 3,094 patients, ergonovine stress was positive in 8.6%. There were no documented cases of myocardial infarction or death. However, 0.6% of patients had transient symptoms, and one patient experienced sudden death but was successfully resuscitated. This modality demonstrated prognostic value for major adverse cardiac events.^91^ Another form of stress used to diagnose vasospastic angina is hyperventilation, with or without cold water immersion. The test consists of a period of hyperventilation with deep breathing at 30 breaths per minute for 6 minutes, followed by immersion of the patient's hand in ice water for 2 minutes. ECG monitoring and echocardiography are performed during the stress period and 15 minutes after its completion. The criteria for cessation are the presence of chest pain and ST elevation >1 mm in at least two leads. Onset of a new regional wall motion abnormality during stress is diagnostic of vasospasm. When compared to intracoronary acetylcholine injection during angiography, this method showed a sensitivity of around 91%, specificity of 90%, and accuracy of 91%.^92,93^

## 9. Diastolic Stress Echocardiography

Heart failure with preserved ejection fraction (HFpEF) is a major public health problem worldwide due to population aging with an increased prevalence of comorbidities. The most common clinical manifestation of HFpEF is dyspnea on exertion.^94,95^ Diagnosis is challenging in patients who only present symptoms during exercise, as natriuretic peptides, cardiac output (CO), and left ventricular filling pressure (LVFP) may all be normal or only slightly abnormal compared to individuals with normal diastolic function (DF). In these cases, the increase in LVFP becomes evident only during exercise, highlighting the importance of stress testing.^94,95^ Diastolic SE is a modality capable of providing information in these questionable cases, using parameters obtained from assessment of left ventricular dysfunction at rest and during exercise.^96-98^

### 9.1. Normal and Abnormal Response to Exercise of Diastolic Function

Normal DF allows for adequate LV filling at rest and during exercise, with increased CO and no elevation of LVFP. The most commonly studied parameters are the ratio of the peak early mitral filling velocity (E) to the early diastolic mitral annular velocity (e’) obtained by tissue Doppler imaging at rest and during exertion. In normal relaxation, during exercise, there is a proportional increase in these velocities, with the E/e’ ratio remaining unchanged.^96,97^ In patients with myocardial disease and abnormal relaxation, there is no increase in e’ as there is in E, and an increase in the E/e’ ratio is expected. E/e’ values < 10 during exercise suggest normal LVFP, whereas values > 14 (average e’) or > 15 (using septal e’ alone) suggest increased LVFP. Estimating pulmonary artery systolic pressure (PASP) using peak tricuspid regurgitation velocity (TRV) at rest and during exercise is also useful, as PASP increases with increasing LVFP.^94,97^

### 9.2. Indications for Stress Echocardiography

For patients with HFpEF and dyspnea on exertion, assessment at rest may be insufficient, thus justifying stress testing to induce an increase in LVFP.^94,97^ Guidelines from different societies suggest diastolic SE in suspected clinical signs of HFpEF.^3,94,98,99^ Figure 9.1 illustrates the recommendations.

**Figure 9.1:** Indications for diastolic stress echocardiography according to current guidelines.^94,95,98,100^

HFA-PEFF score: suggested by the European Society of Cardiology for the diagnosis of HFpEF. H_2_FPEF score: cited in the U.S. guideline for diagnosis of HFpEF.

### 9.3. Protocols Used in Diastolic Stress Echocardiography

Diastolic SE can be performed using a stationary bicycle or treadmill, or on a reclining ergometer/echo couch. The reclining ergometer (echo couch) is the most suggested by evaluations of regional wall motion and DF during each stage and at peak stress.^3,94,99^ When a treadmill is used, regional wall motion can be assessed after exercise ceases (60-90 seconds later), followed by assessment of DF. In both modalities, patients are encouraged to reach the maximum HR predicted for age or continue until the onset of limiting symptoms. Tables 9.1 and 9.2 show the advantages and limitations of the protocols and the variables which can be assessed^3,98^ and the parameters acquired during diastolic stress echocardiography.

**Table 9.1:** Protocols, advantages, and limitations

Modality	Protocols	Advantages	Limitations
Bicycle	Standard: initial load 25 W, 60 rpm, 25-W increments every 2 or 3 minutes.	- Physiological. - More time for acquisition. - Validated by more studies.	- Limited availability. - Difficulty in obtaining images. - Lower workload achieved compared to treadmill.
Treadmill	Bruce: initial incline 10%, followed by 2% increments at each 3-minute stage. Modified Bruce: initial incline 0%, duration 3 minutes per stage, with gradually increasing incline.	- Physiological. - Availability. - High workloads reached. - Higher double product at the expense of a higher HR. - More specific for detecting ischemia than bicycle.	- Difficulty in obtaining images. - Possibility of normalization of post-exercise changes. - Wait until HR is < 100-110/min in recovery (to avoid merging of Doppler flow waves). - No hemodynamic validation.

W: Watts; rpm: revolutions per minute, HR: heart rate

**Table 9.2:** Parameters acquired during diastolic stress echocardiography at rest and during (bicycle) or immediately after (treadmill) exercise

Parameter	Acquisition	Advantages	Limitations
E	- Apical 4-chamber. - Measure at peak early diastolic velocity.	Feasible and reproducible.	- The E and A velocity waves merge at high HR. - Decreases with age. - Acquire when HR is between 100 and 110 bpm.
e' (lateral, medial)	- Apical 4-chamber. - Tissue Doppler imaging of mitral annulus in the septal and lateral region.	Feasible and reproducible.	- The e' and a' velocity waves merge at high HR. - Decreases with age. - Limited accuracy in patients with mitral annulus calcification, prosthetic ring annuloplasty, pericardial disease.
E/e' ratio	- Ratio between the E and e' velocities.	Hemodynamically validated.	- Decreased accuracy in normal individuals, mitral annulus calcification, significant mitral and pericardial disease, regional wall motion abnormalities.
Peak TRV	- Apical 4-chamber or parasternal RV inflow view. - Continuous Doppler.	Indirect adjunct to estimation of LV filling pressure.	- Increased velocity can occur due to illness or as a normal response to increased flow during exercise. - A full signal is not obtained in 1/3 of patients. - Less reliable if there is marked tricuspid regurgitation.

LV: left ventricle; RV: right ventricle; E: peak early mitral filling velocity; A: late left ventricular filling velocity by atrial systole; e’: early diastolic mitral annular velocity obtained by tissue Doppler imaging; HR: heart rate; bpm: beats per minute.

### 9.4. Interpretation of Diastolic Stress Echocardiography

Functional capacity should be compared to that expected for age and sex.^3,94,99^ Table 9.3 illustrates normal and abnormal responses to diastolic SE.

**Table 9.3:** Possible echocardiographic findings at diastolic stress echocardiography

	Diastolic SE in normal diastolic function		Diastolic SE in diastolic dysfunction	
	Pre-exercise	During or immediately after exercise	Pre-exercise**	During or immediately after exercise
Septal E/e' (average)	< 10	< 10	< 14	> 15 (>14)
Peak TRV* (m/s)	< 2.8	< 2.8	< 2.8	> 2.8 / >3,4

* Peak TRV values vary across guidelines. According to ASE/EACVI, it is considered abnormal if > 2,8 m/s (higher sensitivity), whereas according to HFA/ESC, it is abnormal if > 3.4 m/s (higher specificity).^94,99^

** In abnormal diastolic SE, the septal e’ velocities at rest should be < 7 cm/s, or, if only the lateral e’ is evaluated, it should be < 10 cm/s.

Diastolic SE is important for detecting changes that are not present at rest and only appear during physical exertion, and current guidelines recommend its use to identify patients with HFpEF.

## 10. Stress Echocardiography in MINOCA (Myocardial Infarction with Nonobstructive Coronary Arteries) and in Microvascular Disease

MINOCA (myocardial infarction with nonobstructive coronary arteries) encompasses those cases of acute coronary syndrome (ACS) with troponin levels elevated above the 99th percentile, symptoms, or abnormal ECG findings in the absence of epicardial coronary artery obstructions > 50%.^101^ MINOCA accounts for approximately 5-10% of ACS cases.^101,102^ The prognosis for patients with MINOCA is variable and depends on the underlying mechanism.^101^ This condition is more prevalent in younger individuals, particularly females, and conventional coronary heart disease risk factors such as dyslipidemia, smoking, and family history have a reduced prevalence.^103,104^ Identifying MINOCA and ruling out potential differential diagnoses is extremely important for appropriate and early clinical management.^104^

### 10.1. Pathophysiology

Currently, MINOCA is classified into 2 types:^103,104^

- Type I: Plaque-related events: rupture, erosion, and dissection of the coronary artery wall;
- Type II: Imbalance between oxygen supply and consumption, which can occur in coronary spasm, coronary embolism, endothelial dysfunction, anemia, hypertension, hypotension, extremes of heart rate, hypoxemia, etc.

It is important to stress that conditions such as Takotsubo syndrome are not considered MINOCA, as they are not ischemic in nature. However, 10–29% of patients with Takotsubo syndrome have comorbid coronary artery disease.^104^ Other differential diagnoses which must be considered and extensively investigated include myocarditis, sepsis-related changes, pulmonary embolism, and cardiac contusion. When ischemic causes are suspected, invasive hemodynamic assessment is necessary to detect the presence of significant coronary obstruction or occlusion of small secondary branches.^101-104^

### 10.2. Main Diagnostic Methods in MINOCA

The diagnostic workup begins with the typical presentation of chest pain and elevated troponin (> 99^th^ percentile, with a typical curve-like pattern), with or without abnormal ECG findings.^104^ With the diagnosis of ACS established, the patient then undergoes coronary angiography, and the possibility of obstruction ≥ 50% is ruled out after careful review of the images. Anatomical-functional diagnosis can be supplemented by the use of intracoronary ultrasound and optical coherence tomography, methods that allow analysis of plaque characteristics and detection of plaque rupture. Fractional flow reserve measurement can identify plaques that visually appear to cause < 50% obstruction, but have significant repercussions nonetheless.^103-105^

Currently, CMR imaging is the modality of choice for evaluating patients with suspected MINOCA. This method provides an accurate differential diagnosis of clinical mimics of MINOCA. Liang et al.,^102^ in a retrospective study in which CMR was used to reclassify over 800 patients originally diagnosed with MINOCA, found that only 27% of cases were actually MINOCA and 73% had other conditions, such as myocarditis.

Figure 10.1 illustrates the diagnostic algorithm that can be followed when MINOCA is suspected.

**Figure 10.1:** Algorithm for MINOCA diagnosis and/or stratification. ACS: acute coronary syndrome; TT Echo: transthoracic echocardiography; PE: pulmonary embolism; S.: syndrome; CMR: cardiac magnetic resonance imaging; OCT: optical coherence tomography; TE Echo: transesophageal echocardiography. Adapted from Del Buono et al.^107^

Pharmacological stress echocardiography plays a role in MINOCA by evaluating the absence of regional wall motion abnormalities or, more accurately, in cases of vasospasm, which can be diagnosed during the recovery phase of pharmacological stress tests with rapid infusion of beta blockers or aminophylline.^2,3,106^

### 10.3. The Stress Echocardiogram in MINOCA

The latest Brazilian Society of Cardiology Guidelines on Unstable Angina and Myocardial Infarction^103^ contain a Class IIa recommendation for stress echocardiography in MINOCA of microvascular etiology for risk stratification purposes, as the presence of regional wall motion abnormalities carries a worse prognosis.

The ischemic cascade in MINOCA exhibits a different behavior from that observed in epicardial disease, referred to as the alternative and classic ischemic cascade, respectively (Figure 10.2). In the alternative cascade, the primary change is a reduction in coronary flow reserve. In this setting, the standard stress echocardiography protocol which only assesses regional wall motion has low sensitivity. Thus, the ABCDE protocol^106^ is indicated to increase the accuracy of examination and allow risk stratification. This protocol comprises: assessment of ventricular volumes and regional wall motion abnormalities (A), lung ultrasound for B-lines (B) to investigate congestion in the four anterior thoracic segments, assessment of left ventricular contractile (C) reserve by calculating the ejection fraction using volumetric 2D echo, Doppler (D)-based assessment of coronary flow reserve in the left anterior descending artery, and ECG (E)-based assessment of chronotropic reserve.^2^ We strongly encourage use of the ABCDE protocol, as previously described in the aforementioned guideline.^106^ Many of these patients report chest pain on examination, which does not occur at the end of the classic ischemic cascade, but rather at the start.

**Figure 10.2:** Comparison of the ischemic cascade in epicardial disease (classic) and in MINOCA (alternative). Adapted from Picano et al.^2^

Ultrasound enhancement agents can be used to evaluate left ventricular opacification and analyze the endocardial borders, as in epicardial coronary artery disease.^2,3^ Use of these agents for analysis of coronary reserve in the ABCDE protocol and for assessment of myocardial perfusion is considered off-label, but can be useful at centers with appropriate expertise.^2,3^

It is worth noting that MINOCA is a diagnosis of exclusion in ACS, where stress echocardiography plays a diagnostic role primarily to assess microvascular disease and coronary spasm, combined with the ABCDE protocol to increase the sensitivity of the examination.

## 11. Stress Echocardiography in Mitral Valve Disease

SE – preferably using exercise stress – plays an important role in assisting with the refinement of severity diagnosis, prognostic stratification, and definition of treatment options in patients with mitral valve disease (MVD).^108,109^ Physiological conditions and comorbid diseases can modify the clinical presentation and interfere with the accuracy of conventional diagnostic methods in assessing the severity of MVD, which is influenced by hemodynamic conditions such as preload, afterload, and heart rate. In some cases, there may be discordant severity criteria on echocardiography and symptoms may be difficult to assess (due to sociocultural aspects, cognitive changes, sedentary lifestyle, etc.), leading to what is known as "clinical-echocardiographic dissociation." In these patients, it is important to consider supplementing conventional echocardiography with other imaging methods, as well as with SE for functional assessment, to better define treatment goals.

There are three core indications for SE in the evaluation of native mitral valve disease: 1) Severe asymptomatic MVD; 2) Non-severe symptomatic MVD; and 3) MVD with low transvalvular flow. Physical exercise (ESE) is the stress method most indicated for assessment of MVD, as it allows evaluation of exercise tolerance and emergent symptoms and the acquisition of hemodynamic data in a physiological setting (reproducible of activities of daily living), although dobutamine stress can also be used in cases of mitral stenosis (MS) and patients with limited exercise ability.^110^ For ESE, either a treadmill, stationary bicycle/cycle ergometer, or reclining ergometer/echo couch can be used, with the latter having the advantage of allowing continuous echocardiographic imaging. This allows the echocardiographer to detect abnormalities at low workload (before peak exertion) and obtain data and images at peak exertion, which may be underestimated with the use of a treadmill because image capture is done after peak exertion.

### 11.1. Mitral Stenosis

Rheumatic MS remains a significant public health issue in Brazil and other developing countries, with a high incidence in Africa, some regions of Asia, and across Latin America.^111,112^ It is still the most prevalent cause of MS worldwide, followed by degenerative diseases. The incidence of degenerative MS associated with mitral annular calcification increases with age, and is the predominant cause of MS in industrialized countries.^113^

SE has a well-validated indication for evaluating the severity and hemodynamic repercussions of MS, especially in patients who present with a discrepancy between the degree of stenosis determined by imaging methods and their clinical findings (clinical-echocardiographic dissociation).^108,114,115^ Patients with moderate MS as quantified on resting echocardiography are often quite symptomatic on exertion, and SE may be able to demonstrate hemodynamic changes that justify these symptoms, such as a significant increase in transvalvular gradients and PASP (Table 11.1). Poor prognostic criteria include an absolute increase in mean gradient above 15 mm Hg with exertion^108,110^ or an increase in PASP above 60 mm Hg.^116^ Other situations in which SE may be useful in MS are in patients scheduled to undergo major surgery or patients who intend to become pregnant, to predict how the disease will behave in hemodynamically adverse situations.^117^ In patients with exercise limitations, dobutamine stress echocardiography can be used, as it has been validated for this specific population, and, in this case, an absolute mean gradient value of 18 mmHg indicates a worse prognosis.^118^ The risk of acute pulmonary edema and hemodynamic decompensation when these patients undergo SE must be taken into account.^118^

**Table 11.1:** Use of stress echocardiography in assessment of mitral valve disease^116,108,110,117^

Mitral valve condition	Type of stress	Guideline-established criteria	Clinical trial-established criteria	Parameters of interest
Primary mitral regurgitation	Physical activity	-	Exercise-induced increase in PASP > 60 mm Hg Contractile reserve Exercise-induced increase in EROA > 10 mm	Symptoms Pressure response Exercise-induced increase in PASP > 60 mm Hg New regional wall motion abnormalities Exercise-induced increase in LVEF < or > 5% Exercise-induced increase in GLS < or > 2%
Mitral stenosis	Physical activity (preferable)	Mean transmitral gradient increase > 15 mm Hg at peak exertion (or > 18 mm Hg with dobutamine)	PASP increase > 60 mm Hg	Symptoms Pressure response Exercise-induced increase in PASP > 60 mm Hg New regional wall motion abnormalities Exercise-induced decrease in LVEF Mean transmitral gradient increase > 15 mm Hg at peak exertion

Adapted from Lancellotti et al.^116^

### 11.2. Mitral Regurgitation

The main cause of mitral regurgitation (MR) in developed countries is degenerative mitral valve disease,^119^ whereas in developing countries, rheumatic fever remains an important etiological factor and driver of morbidity and mortality.^120^ Recent guidelines have established severity criteria for MR.^133^ Echocardiography plays an important role in determining the optimal timing of intervention, especially in symptomatic patients, as well as in asymptomatic ones with significant ventricular remodeling, left ventricular dysfunction (LVEF ≤ 60%), atrial fibrillation (AF), and/or pulmonary hypertension.^114,122^

Echocardiography plays a fundamental role in the evaluation of patients with MR, especially in those with discrepancies between clinical and echocardiographic findings, such as symptomatic patients with non-severe MR.^123^ Stress testing has the added benefit of assessing the patient's functional capacity, not only clarifying the correlation between symptoms and hemodynamics but also stratifying risk in patients who are reportedly asymptomatic despite severe MR. Symptomatic patients with apparently non-severe MR on baseline echocardiography may have dynamic mechanisms in play that become clearer during SE.^124^ Therefore, exercise testing may be indicated in patients with mitral valve disease who have no other apparent cause of dyspnea on exertion, to rule out the presence of dynamic MR.

SE provides important objective data and can be performed in conjunction with cardiopulmonary exercise testing by adding other functional assessment variables, such as oxygen consumption.

In patients with primary MR, the main parameters that can be analyzed during SE are worsening of MR on exertion (increase in ERO > 10 mm), right ventricular dysfunction, and increase in PASP > 60 mm Hg^125,126^ (Table 11.1), which are predictors of worse prognosis. The onset of symptoms or a drop in systolic blood pressure are also indicative of worse prognosis.

In a study by Kusunose et al., the presence of tricuspid annular plane systolic excursion (TAPSE) < 1.9 cm or PASP > 57 mm Hg during exertion were predictors of progression to mitral valve surgery in asymptomatic patients with severe MR.^127^ The absence of myocardial reserve, as indicated by an increase in ejection fraction ≤ 5% or an absolute increase in LV global longitudinal strain ≤ 2%, has also been shown to be associated with a worse prognosis in MR patients undergoing SE.^128,129^

SE can be used to evaluate patients with secondary MR, especially when an ischemic component is suspected as the underlying functional mechanism. In patients with nonischemic dilated cardiomyopathy (NIDCM), improvement in MR severity resulting from mobilization of contractile reserve is common and portends a good prognosis, while in patients with ischemic cardiomyopathy, there may be worsening of MR severity, indicating a worse prognosis^130,131^ and suggesting that the mitral valve must be repaired during coronary artery bypass graft surgery.

The suggested examination protocol for stress echocardiography in MVD is shown below.

### 11.3. Stress Echocardiography Protocol for Evaluation of Mitral Valve Lesions

Exercise stress echocardiography is preferred and can be performed on a reclining ergometer (echo couch), stationary bicycle, or treadmill. Exercise is symptom-limited and test cessation should occur upon exceeding the target heart rate or if fatigue or complications arise (arrhythmias, typical chest pain, dizziness, precipitous decline in blood pressure). ECG and noninvasive blood pressure monitoring throughout the procedure are mandatory.

When using a cycle ergometer, the protocol may start with a 25W load, increasing by 25W every 2–3 minutes, with the advantage of being able to continuously monitor the echocardiogram and evaluate right ventricular function, parameters for assessing valvular lesions (mean gradients, severity of mitral valve regurgitation), left ventricular function, and PASP. The maximum workload achieved is lower than that obtained on a treadmill, although it is sufficient for the evaluation of valvular heart disease during exercise.

In the case of treadmill testing, the Bruce or modified Bruce protocols, or ramp protocol, are commonly used under the supervision of a qualified exercise testing provider. Fatigue or any complications should prompt test cessation. Echocardiographic images and data should be acquired at rest and immediately after exertion, taking care not to prolong the post-exercise examination excessively (image capture ideally within 90 seconds of cessation of exertion).

## 12. Stress Echocardiography in Aortic Valve Disease

### 12.1. Aortic Stenosis

Aortic valve stenosis is an increasingly prevalent condition in our setting, particularly due to senile valvular degeneration associated with population aging.

Significant aortic stenosis (AS) is defined by an aortic valve area (AVA) of ≤ 1.0 cm^2^. Normally, the pressure gradient across the valve correlates with the transvalvular flow and the valve orifice.^132^

A complete assessment of the aortic valve requires evaluation of the transaortic gradient and flow, as well as of the AVA at rest and under stress, especially when transvalvular flow is reduced. If the AVA is < 1 cm^2^ with a high transvalvular gradient at rest (mean gradient > 40 mm Hg) and the peak aortic valve velocity is > 4.0 m/s, a diagnosis of significant aortic stenosis can already be established.^114^

Among the four categories of AS – high-gradient, classic low flow-low gradient (LFLG), paradoxical LFLG, and normal flow-low gradient – stress echocardiography is most important in the classic LFLG form, i.e., with a reduced ejection fraction. A low resting gradient in the presence of a reduced AVA does not rule out severe AS, which can occur due to a reduced ejection fraction, but low-flow AS with preserved ejection fraction (paradoxical) can also occur.

Indication: SE is definitively indicated in AS when the ejection fraction is reduced (i.e., AVA ≤ 1.0 cm^2^, LVEF < 50%, and mean transaortic gradient < 40 mm Hg). In these cases, it becomes important to differentiate truly severe fixed aortic valve stenosis from pseudo-severe aortic stenosis, in which the primary problem is cardiomyopathy, leading to overestimation of the severity of the aortic valve lesion due to inadequate valve opening secondary to myocardial impairment.^133-135^ The dobutamine stress protocol used in these cases is demonstrated in Figure 12.1.

**Figure 12.1:** Low-dose dobutamine protocol used in classic low-flow, low-gradient aortic stenosis.

In classic LFLG AS, low-dose DSE is indicated to assess for the presence of contractile/flow reserve, which, if present, allows assessment of the severity of AS. Three response patterns may be seen^133^ (Figure 12.2):

1. Increased flow reserve ≥ 20% with a clear increase in AVA to > 1.0 cm^2^ and a slight increase in the transvalvular gradient, indicating pseudo-severe AS; optimized medical management should be recommended.
2. Increased flow reserve ≥ 20% with no change in AVA (remains ≤ 1.0 cm^2^) and a increase in the transvalvular gradient (generally to a mean gradient of >40 mm Hg), indicating true-severe, fixed aortic stenosis; aortic valve replacement is recommended.
3. When there is no adequate increase in flow reserve (stroke volume increases by less than 20%), and the other parameters therefore remain essentially unchanged, the test is compromised and the severity of AS is considered indeterminate. In these cases, a computed tomography scan with calculation of the aortic valve calcium score helps estimate AS severity. This scenario occurs in one-third of patients and is associated with worse prognosis. Nevertheless, this response pattern should not contraindicate surgery, since many such patients experience improved cardiac performance after aortic valve replacement.

**Figure 12.2:** Indications for dobutamine stress echocardiography in classic low-flow, low-gradient aortic stenosis and possible responses to the test according to the presence or absence of flow reserve. AS: aortic stenosis; Dob: dobutamine; LVEF: left ventricular ejection fraction; AVA: aortic valve area; SVI: stroke volume index; MG: mean gradient.

Clavel et al.^136^ suggest that, when the stroke volume is <20% during DSE, the projected AVA should be calculated considering an estimated normal transvalvular flow (i.e., flow = stroke volume/ejection fraction), and the projected valve area at a normal flow rate, which is standardized as 250 mL/s, can be calculated using the following formula: projected AVA = resting AVA + (change in AVA/change in flow) × (250 – flow rate), where a projected AVA ≤1.0 cm^2^ is indicative of severe AS.^136-138^ A > 20-bpm increase in HR during DSE or the development of arrhythmias or hypotension should prompt test cessation.

For patients with paradoxical LFLG AS, with EF ≥ 50%, AVA ≤ 1.0 cm^2^, mean gradient < 40 mm Hg, and indexed stroke volume ≤ 35 mL/m^2^, DSE is not officially recommended due to limited scientific evidence and the possibility of significant hypotension due to the risk of a dynamic gradient in the left ventricular outflow tract. A Computed tomography calcium scoring showing values ≥ 2000 Agatston units in men and ≥ 1200 in women suggests the possibility of significant AS. In addition, values ≥ 3000 in men and ≥ 1600 in women confirm significant AS.^137^

For asymptomatic patients with severe high-gradient AS, exercise stress echocardiography, preferably done on a reclining ergometer or echo couch, is considered to add prognostic value beyond that of exercise stress test alone.^99^ The finding of an increase in mean transvalvular gradient > 20 mm Hg during exercise reflects lower valvular compliance and greater lesion severity.^99,139^ These patients have a higher risk of cardiovascular events. Other findings that SE may demonstrate include an increase in PASP > 60 mm Hg. However, there is still no robust data to support a routine indication for ESE in these patients^110^ (Figure 12.3).

**Figure 12.3:** Different stress echocardiography modalities and parameters of interest according to the type of aortic valve lesion. DSE: dobutamine stress echo; AR: aortic regurgitation; AS: aortic stenosis; AVA: aortic valve area; Ex: exercise; Ex-MG: mean gradient during exercise; LVEDD: left ventricular end-diastolic diameter; LVEF: left ventricular ejection fraction; LVESD=left ventricular end-systolic diameter, LVESI=left ventricular end-systolic index, PAP=pulmonary artery systolic pressure.

### 12.2. Aortic Insufficiency

In severe, chronic aortic insufficiency (or aortic regurgitation), patients may be asymptomatic and still progress to latent or overt ventricular dysfunction, which may be irreversible.

In these cases, ESE or DSE is able to identify the presence or absence of a contractile reserve (increase in ejection fraction by at least 5% or absolute longitudinal strain ≥ 2%) or flow reserve (increase in stroke volume ≥ 20%) before surgical intervention, factors which predict functional recovery after aortic valve replacement.^110,140^

Validation of SE to predict outcomes in this subgroup of asymptomatic patients with severe aortic insufficiency is limited, mainly due to the small number of available studies.^114,141^

## 13. Stress Echocardiography in Dilated Cardiomyopathy

Dilated cardiomyopathy (DCM) is a primary heart disease characterized by myocardial enlargement in the absence of other previously known etiologies, as well as systolic dysfunction. As the number of diagnosed cases grows, assessing patients’ current status and prognosis is becoming increasingly important, given that advances in treatment have enabled increased survival rates.

SE has proven to be of great importance in this population for etiological diagnosis and prognostic stratification through analysis of contractile reserve; assessment of functional status under induced stress conditions; determination of who will benefit from beta blocker therapy; and identification of patients with a worse prognosis in terms of cardiac death and need for transplantation.

Although there is no established consensus on the optimal protocol, high-dose dobutamine (up to 40 mcg/kg/min) without addition of atropine is most common, mainly because it elicits a more complete contractile response and allows for the assessment of inotropic reserve in these patients who are often on beta blockers, reducing the risk of false negative results without adding to the risk of major complications.^99^ It has been suggested that an increase in contractility, expressed by the change in the WMSI at peak dobutamine stress and baseline values, and an increase ≥ 5% in LVEF during dobutamine infusion are associated with better survival rates, lower rates of hospitalization for heart failure, and an increase in LVEF during follow-up. Patients with a contractile reserve respond better to beta blocker therapy, are less likely to require transplantation, and show an inverse relationship to the extent of interstitial fibrosis.^99,142-146^ Conversely, the absence of contractile reserve in patients with preserved or reduced LVEF is frequently associated with limited coronary flow reserve, and is therefore a marker of latent left ventricular systolic dysfunction and subclinical cardiomyopathy.^99^

The most common adverse event associated with high-dose dobutamine in this population was complex ventricular arrhythmia (multifocal premature ventricular contractions in 6.4% and nonsustained ventricular tachycardia in 1.6%), generally occurring in patients with hypokalemia.^142,143^

The vasodilator-based protocol (dipyridamole 0.84 mg/kg over 10 min, with the addition of handgrip exercise at the peak of infusion to increase test sensitivity) is rarely used to assess contractile reserve; however, it has been proposed as an alternative to dobutamine, as it is less arrhythmogenic, better tolerated, and provides prognostic information similar to that in patients with ischemic heart disease, in addition to not being influenced by beta blockade;^147^ however, it has not gained much traction in clinical practice for this indication when compared to dobutamine.^49,148-150^

The following tables provide recommendations for stress echocardiography in DCM (Tables 13.1, 13.2, 13.3, 13.4 and 13.5).

**Table 13.1:** Parameters to be assessed in stress echocardiography for dilated cardiomyopathy

**1. What should be assessed during stress echocardiography of the patient with dilated cardiomyopathy?**	
- Contractile reserv	- Diastolic reserve
- Stress-induced ischemia	- Dynamic mitral regurgitation
- Change in PASP	- Pulmonary congestion (B-lines)

**Table 13.2:** Parameters to be measured in stress echocardiography for dilated cardiomyopathy

**2. What should be measured?**
- E and A waves
- e' (PW Doppler)
- Mitral and tricuspid regurgitation (CW Doppler)

**Table 13.3:** Time points for measurement of parameters in stress echocardiography for dilated cardiomyopathy

**3. When to measure (dobutamine protocol)?**
- Baseline - Low dose - Peak infusion

**Table 13.4:** Expected responses to stress echocardiography in dilated cardiomyopathy

**4. Expected responses:**
- Increase or no increase in left ventricular contractility
- Reduction or increase in mitral regurgitation
- Changes in PASP and E/e' ratio
- Presence or absence of B-lines in the lung fields

**Table 13.5:** Protocols used for stress echocardiography in dilated cardiomyopathy (A: dobutamine; B: dipyridamole)

**5. Protocols** (Figure 12.4).

**Figure 12.4:** Protocols used for stress echocardiography in dilated cardiomyopathy (A: dobutamine; B: dipyridamole).

## 14. Stress Echocardiography in Chagas Cardiomyopathy

Patients with chronic Chagas disease cardiomyopathy (CCDC) frequently present with atypical chest pain and ECG abnormalities, such as ST segment changes and pathological Q waves, along with regional wall motion abnormalities and perfusion disorders that mimic the presentation of atherosclerotic coronary artery disease.^151^

As recently as a few decades ago, patients with Chagas disease were predominantly rural workers with a low risk profile for obstructive coronary artery disease. With the growing urbanization of this population from 1980 onwards, they became exposed to the same risk factors for atherosclerotic disease as individuals not infected with *Trypanosoma cruzi*.^152^ However, in most cases, examination of the epicardial coronary arteries reveals absence of significant subepicardial atherosclerotic obstruction, with any changes being attributed to coronary microvascular dysfunction.^153^

Some studies have evaluated the applicability of stress echocardiography in Chagas disease. Microvascular ischemia is considered an early disorder in the natural history of the disease, preceding regional ventricular dysfunction and possibly related to induction of myocardial hibernation or stunning. Results obtained from a stress echocardiography study using dipyridamole demonstrated a reduction in coronary vasodilator reserve, an index of microvascular dysfunction, in patients with the indeterminate form of the disease when compared to normal controls.^154^

Regarding chronotropic response, dobutamine echocardiography demonstrated that patients with Chagas disease exhibited blunted inotropic and chronotropic responses when compared to a control group. Furthermore, pharmacological stress echocardiography can demonstrate the presence of a biphasic contractile response in these patients, despite the absence of epicardial coronary artery disease.^155^

Regarding predictive value for progression of heart disease, the presence of complex ventricular arrhythmias on stress echocardiography was an independent predictor of disease progression in a group of patients in the early stages of Chagas cardiomyopathy.^156^

Finally, regarding the use of dobutamine in arrhythmogenic heart disease, pharmacological stress echocardiography proved safe in a cohort of patients with Chagas cardiomyopathy under investigation for coronary artery disease. The presence of an abnormal regional wall motion index at rest was an independent predictor for the development of complex arrhythmias during the examination.^157^

## 15. Stress Echocardiography in Hypertrophic Cardiomyopathy

HCM is a common heart disease of genetic origin, with an autosomal dominant inheritance pattern and equal distribution by sex. Prevalence varies depending on age, subclinical or clinical status, and ethnicity.^158^ Clinical suspicion may be aroused by manifestation of symptoms, detection of a heart murmur, an abnormal ECG pattern, or a cardiac event. In addition, it may be an incidental finding during echocardiography performed for other indications.^159^

Echocardiography plays a very important role in confirming the diagnosis, determining the pattern of hypertrophy, evaluating LVOTO, systolic anterior motion (SAM) of the mitral valve, and mitral regurgitation (MR), as well as assessing left ventricular systolic and diastolic function, left atrial volume, and pulmonary pressure.^160^

SE has been widely used in this setting, primarily to measure dynamic and inducible LVOTO in symptomatic patients with a resting peak LVOT gradient < 50 mm Hg, to detect MR, and to monitor treatment response. The importance of a gradient > 50 mm Hg, whether at rest or post-exercise, lies in the fact that it is the conventional limit for invasive intervention when symptoms are not controlled with pharmacotherapy.^158^ ESE is the most recommended modality in these cases.

Exercise can be performed on a reclining ergometer/echo couch, stationary bicycle, or treadmill. The treadmill has been suggested to induce a higher LVOT gradient. The choice of protocol should take into account the patient's overall condition, the physician's experience, and the resources at the institution's disposal.

Before the test, it is important to be aware of the patient's condition (functional class, hydration status, timing of last meal, intake of alcoholic beverages, etc.) and pharmacological factors (vasodilators, diuretics), as these can influence the outcome of the examination. Unlike in other stress test modalities, beta blockers should not be withdrawn before exercise in this patient population.

Ultrasound enhancement agents are useful in confirming septal and apical hypertrophy, detecting apical thrombi, and excluding apical aneurysms.^161^

It is essential that the patient be informed of the purpose of the study and its possible outcomes. As one of the goals of SE is to quantify the LVOT gradient in symptomatic patients with no gradient or a low resting gradient, it is recommended that the gradient be measured as frequently as possible throughout the examination. SAM, MR, left ventricular wall motion abnormalities, and diastolic dysfunction should also be assessed.

The ECG should be monitored continuously for any change. Clinical and echocardiographic parameters are evaluated during exercise and in the early and late recovery phases, while preload decreases gradually. If exercise stress does not cause a significant increase in the LVOT gradient, assessment of the post-exercise gradient in the orthostatic position should be considered. A hypertensive response, any reduction in BP > 10 mm Hg compared to the resting BP before the examination, dyspnea, chest discomfort, and sustained tachyarrhythmia are indications for test cessation.^162^ LVOTO at a low exercise load correlates with low functional capacity. LVOTO is a risk factor for sudden death and major cardiovascular events.^163^

Since stress echocardiography is an operator-dependent technique, a supervised learning curve is essential for the echocardiographer to gain expertise in patients with HCM, in order to obtain reliable and reproducible results that allow for informed decisions to support patient management and follow-up. Notably, the clinical course of HCM is heterogeneous and unpredictable. SE has the potential to differentiate non-obstructive HCM from HCM with a dynamic gradient and, taken together with exercise capacity, blood pressure, and heart rate response, predict the future development of heart failure, thus informing the choice of management strategies available to each patient in the context of this complex genetic disease. Procedures such as surgical septal myectomy and percutaneous alcohol septal ablation have Class I indications for symptomatic patients with a resting LVOT gradient and those without an acceptable response to pharmacological therapy.^164^

## 16. Stress Echocardiography in the Evaluation of Right Ventricular Function and Pulmonary Hypertension

Pulmonary hypertension (PAH) is a pathophysiological derangement that can involve a wide range of clinical conditions and be associated with countless respiratory and/or cardiovascular diseases. Regardless of the underlying disease process and the site of functional changes, PAH is defined by a mean resting pulmonary artery pressure (mPAP) of >20 mm Hg as measured by right heart catheterization (RHC).^165^

Recently, there has been increasing interest in the application of SE for the detection and assessment of the severity of PAH and right heart dysfunction.^166,167^

RHC is the gold-standard method for evaluating pulmonary hemodynamics. It allows diagnosis and classification of PAH. According to current consensus guidelines, exercise RHC is the recommended method for assessment of cardiopulmonary hemodynamics. However, this method entails some technical challenges, requiring experienced personnel, special equipment, and additional procedure time.^165^

SE of the pulmonary circulation and right ventricle reveals relevant prognostic differences between healthy individuals, athletes, high-altitude residents, and patients with various cardiorespiratory conditions. It can assess pulmonary circulation and right ventricular behavior noninvasively in these individuals, and it also aids in the diagnosis of latent or overt PAH, with or without RV dysfunction, adapting to increased workload.^168^

Similar to the concept of exercise RHC, supplementing standard echocardiography with a stress test can improve diagnostic accuracy in detecting postcapillary pulmonary hypertension. Furthermore, exercise stress has added value in the management of patients with PAH, as it also allows assessment of complex RV mechanics (e.g., contractile reserve) and the ability of the pulmonary vasculature to accommodate increased flow.^168^

The echocardiographic measures of RV function that are independent predictors of mortality in PAH include a TAPSE < 18 mm,^169-173^ RV fractional area change (FAC) < 35%, a peak tricuspid annular systolic velocity (S’) < 9.7 cm/s,^174^ and a Tei index > 0.40 by pulsed Doppler or > 0.55 by tissue Doppler.^175^

All the metrics described above can be evaluated at rest and after peak stress (exercise or dobutamine stress echocardiography). To date, most data have been derived from healthy volunteers, but several small and medium-sized studies have evaluated the prognostic role of right ventricular contractile reserve in patients with PAH, valvular heart disease, and heart failure.^176^

Whether non-invasive echocardiographic assessment is equally reliable and generally feasible for defining disease conditions with or at risk of PAH is still unknown. Similar limitations apply to the echocardiographic assessment of RV function during exercise.^167,176^

One potential noninvasive measure of RV contractile reserve using exercise stress was proposed by Grünig et al., who demonstrated that an exercise-induced increase in pulmonary artery pressure (PAP) was a measure of RV contractile reserve and an independent prognostic factor in patients with precapillary pulmonary hypertension.^177^

## 17. Stress Echocardiography in Post-COVID-19 Assessment

Most publications regarding the cardiovascular manifestations of COVID-19 have evaluated the acute phase of infection, demonstrating that the presence of cardiac dysfunction detected by resting echocardiography was associated with a worse prognosis in patients with severe COVID-19.^178^ Due to the high risk of cross-contamination via droplet transmission, exercise stress echocardiography was largely postponed or canceled during the pandemic.^179,180^ In low-risk COVID-19 patients where the indication was appropriate and postponement was not possible, pharmacological stress echocardiography was the preferred option.^179,180^

After the end of the pandemic state of emergency, several studies attempted to describe the long-term consequences of COVID-19 on the cardiovascular system, but most still only used resting echocardiography. Subsequently, very few publications explored the use of stress echocardiography as a tool in the assessment of patients after recovery from SARS-CoV-2 infection. A prospective study of 174 young athletes with normal ventricular function at rest after COVID-19 aimed to identify myocardial involvement through a comprehensive functional protocol that included physical stress echocardiography.^181^ Signs of myocardial involvement were found in five athletes (2.9%), three of whom showed clinically significant changes on exertion, including ventricular arrhythmia in two and regional wall motion abnormalities in one subject.^181^ Although rare, myocardial involvement was only detected during exertion, suggesting that screening with exercise stress echocardiography may be useful in selected cases for guidance on returning to competitive athletic activity after SARS-CoV-2 infection. Another study evaluated 71 patients recovering from COVID-19, most of whom were symptomatic, using a protocol that combined physical stress echocardiography and cardiopulmonary exercise testing.^182^ Peak oxygen consumption (VO_2_) was lower in post-COVID-19 patients compared to the control group, due to a combination of chronotropic incompetence and blunted left ventricular stroke volume reserve (insufficient increase in stroke volume during exertion).^182^ These studies highlight the importance of assessing cardiac function not only at rest but also under stress, such as exercise, to fully understand the impact of COVID-19 infection on the heart. Ongoing investigations may, through the use of stress echocardiography, provide a better understanding of the relationship between a history of SARS-CoV-2 infection, its cardiovascular implications, and physical activity.^183^

The use of ultrasound enhancing agents (UEAs) warrants special mention, as these agents are indispensable tools for modern stress echocardiography practice. After December 2020, coinciding with the start of COVID-19 vaccination in the U.S. population, an increase in serious adverse reactions to UEAs containing polyethylene glycol (PEG), such as Sonovue or Lumason (Bracco Diagnostics) and Definity (Lantheus Medical Imaging), was observed. The possibility of cross-reactivity between these UEAs and mRNA-based COVID-19 vaccines (Pfizer/BioNTech and Moderna) was considered as a possible cause, since both contain PEG.^184^ A retrospective case-control study suggested that individuals with severe adverse reactions to Lumason occurring since March 2021 in the U.S. were more likely to have been vaccinated against COVID-19 than those who did not experience such adverse reactions.^185^

## 18. Stress Echocardiography and Coronary Flow Reserve in Native Vessels

Coronary flow reserve (CFR) can be assessed through direct analysis of arterial flow during SE. Specific presets allow visualization, without the use of microbubbles, of the left anterior descending (LAD) coronary artery during pharmacological stress with adenosine, dipyridamole, or even dobutamine in at least 90% of cases; the success rate is lower in the posterior descending (PDA) and circumflex (Cx) arteries, particularly in ESE.^23,186-189^

The CFR is calculated by dividing the diastolic velocity obtained at hyperemia by the diastolic velocity recorded at rest (Figure 18.1). Testing for correlation between the CFR measured during SE and the percentage of coronary stenosis found that a CFR ≥ 2 indicates normality and is consistent with a good prognosis. The CFR may be reduced (<2) by coronary stenosis and/or a compromised microcirculation, and is an independent predictor of adverse outcomes. Lower CFR values are more indicative of significant coronary stenosis (Table 18.1).^187,190-194^

**Figure 18.1:** Normal baseline CFR in the left anterior descending artery, increasing significantly (CFR = 3.3) with 30 mcg/kg/min dobutamine. LAD: left anterior descending artery; DSE: dobutamine stress echocardiography; HR: heart rate; DPV: diastolic peak velocity; CFR (coronary flow reserve) = DPV during stress / DPV at baseline. Normal CFR (≥ 2) and early CFR (before reaching the target heart rate of the test), recorded during intermediate stages of DSE.

In intermediate LAD stenosis (50–70%), percutaneous coronary intervention can be indicated when the CFR is reduced versus medical management when the CFR is normal, as there was no difference in the rate of death, myocardial infarction, unstable angina, or myocardial revascularization between the two groups.^195^

An abnormal CFR has high sensitivity and specificity for diagnosing restenosis in the three main coronary arteries (Table 18.1). One study showed high feasibility of recording in the ADA (95%), PDA (85%), and Cx (81%), with a UEA or microbubble contrast used in 22% of cases.^194^

**Table 18.1:** Correlation between coronary flow reserve and percent coronary stenosis

Authors	No. of cases	CFR (cutoff)	Stressor	% Stenosis	Sensitivity	Specificity	Accuracy
Nohotomi et al.^187^	110 LAD	< 2	Dipyridamole	> 50%	94%	65%	81%
Voci et al.^190^	44 (LAD+PDA)	< 2	Adenosine	≥ 70%	89%	96%	93%
	18 PDA	1.4 ± 0.54		≥ 70%	89%	91%	93%
	19 LAD	1.12 ± 0.49		≥ 70%	89%	100%	95%
Lowenstein et al.^191^	132 LAD	< 2	Dipyridamole	> 70%	87%	73%	80%
Matsumara et al.^192^	138 LAD	-	Adenosine				
	30 LAD	< 2		≥ 70%	90%	93%	
Soylu et al.^193^	44 LAD	< 1.6	Dipyridamole	≥ 55%	100%	94%	
Hyodo et al.^194^	175 vessels	-	Adenosine	> 50%			
	166 LAD	< 2		> 50%	86%	91%	
	149 PDA	< 2		> 50%	92%	92%	
	142 CX	< 2		> 50%	91%	92%	

CFR: Coronary flow reserve; LAD: Left anterior descending artery; PDA: Posterior descending artery; CX: Circumflex artery.

Dobutamine is the most commonly used stressor for pharmacological SE and also has a vasodilatory effect.^196^ The first study to assess CFR in the LAD under dobutamine stress showed 90% viability. Cases with ischemia (as determined by regional wall motion abnormalities) had a mean CFR of 1.51 ± 0.51, while those without ischemia had a mean CFR of 2.76 ± 0.65.^186^

During dobutamine stress echocardiography, a normal CFR can be attained early, i.e., before the target heart rate for the test is reached.^197^ Cases reaching a normal CFR before the heart rate endpoint of the test showed a better composite outcome than cases in which normal CFR was recorded only after the submaximal heart rate had been exceeded and cases in which the CFR was abnormal. Once an early normal CFR has been reached, CFR determination can be stopped and the SE continued as normal.^179^

Among diabetic patients undergoing dipyridamole and dobutamine SE, in cases without ischemia, an abnormal CFR in the LAD was independently predictive of mortality, and this finding was similar for both techniques.^199^ This was further corroborated in a study evaluating CFR in the LAD and PDA during dipyridamole stress, which found that an abnormal CFR was predictive of worse prognosis (death and myocardial infarction) in patients without ischemia.^189^

## 19. Stress Echocardiography and Coronary Flow Reserve in Grafts

Anatomical analysis of epicardial vessel lesions by coronary angiography may not be sufficient to assess the impact of coronary lesions, especially in intermediate obstructions (50–70%) and in patients with multivessel disease,^200^ partly due to the changes that stenosis causes in the regulation of circulation. Stenosis > 50% of the vessel lumen increases coronary resistance and decreases pressure distal to the obstruction, with a decrease in CFR.^201^ For this reason, physiological assessment of the coronary circulation, as a determinant of myocardial revascularization, has improved the outcomes of both surgical and percutaneous procedures, as well as the post-procedure prognosis. For this purpose, several diagnostic methods stand out beyond coronary angiography, including stress echocardiography and CFR analysis by transthoracic Doppler imaging.

### 19.1. Stress Echocardiography after Myocardial Revascularization

Just as a regional wall motion score > 1.7 on stress echocardiography performed before revascularization is predictive of increased postoperative complications,^202^ an abnormal study after revascularization more than doubles the risk of adverse cardiovascular events,^203^ even in asymptomatic patients. Although vasodilator stress echocardiography is the best option for these cases, low-dose dobutamine stress, while influenced by several factors, has shown good sensitivity (86.4%) in detecting recovery of contractile reserve after revascularization, especially in previously hypokinetic segments.^204^

### 19.2. Coronary Flow Reserve after Myocardial Revascularization

Fractional flow reserve, assessed during interventional cardiology studies using either a pressure gradient or flow velocity through the obstruction, is an important tool for the functional analysis of obstructive coronary artery disease (Class IA recommendation).^205^ Following revascularization, transthoracic Doppler coronary flow measurement and CFR analysis with vasodilator stress can be used as a substitute for invasive methods.^206^ In internal thoracic (mammary) artery grafts, the finding of biphasic flow with diastolic predominance is indicative of graft patency (Figure 19.1). Increased coronary flow velocity distal to the graft site after vasodilator infusion (dipyridamole 0.84 mg/kg over 6 minutes, although the CFR obtained at 4 minutes is already considered satisfactory, or adenosine 0.14 mg/kg/min over 2 minutes) allows determination of CFR. A ratio between flow at peak hyperemia and baseline flow >2 is considered a good response^207^ (Figure 19.2).

**Figure 19.1:** A: Detection of internal thoracic artery flow with pulsed Doppler sample volume placement. B: Recording of flow in the internal mammary-to-coronary artery graft, showing biphasic flow with diastolic predominance, indicating that the graft is patent.

**Figure 19.2:** Coronary flow in an internal thoracic artery graft. A: Baseline coronary flow obtained in the anterior descending artery after anastomosis. B: Flow obtained 6 minutes after dipyridamole infusion, showing a coronary flow reserve (CFR) of 2.35. The slope of the diastolic deceleration time waveform correlates with the resistance offered by the vascular bed to the passage of flow during diastole. Vasodilation reduces vascular resistance, resulting in a faster deceleration of blood flow.

In venous grafts, flow within the graft is predominantly systolic, emptying into the coronary artery during diastole (Figure 19.3). The analysis and interpretation of CFR are similar to those in arterial grafts.

**Figure 19.3:** Coronary flow in a venous graft to the anterior descending artery. A: Flow in the venous graft, with systolic predominance. B: Flow in the artery distal to the graft, biphasic and with diastolic predominance.

Some conditions can decrease the CFR through arterial grafts without necessarily causing stenosis, such as: atheromatosis of the left subclavian artery or of the arterial graft, which can decrease flow to the grafted artery; diffuse atheromatosis of the distal bed of the irrigated artery; increased microvascular resistance secondary to previous myocardial infarction; abnormalities in the coronary microcirculation; and the timing of the examination, considering the gradual remodeling of arterial grafts, with a progressive increase in CFR. For venous grafts, causes of decreased CFR include graft spasm, a rare but serious condition; graft atherosclerosis; and fibrointimal hypoplasia with fibrosis.

## 20. Stress Echocardiography in the Assessment of Myocardial Viability

The clinical approach to akinetic myocardial areas demonstrably viable on imaging (hibernating myocardium) is controversial, mainly after two large trials, STICH (Surgical Treatment for Ischaemic Heart Failure) and HEART (Heart Failure Revascularization), showed that despite improvement in left ventricular ejection fraction there was no positive impact on the long-term prognosis of these patients.^208,209^ Hibernating myocardium refers to dysfunctional areas due to chronic hypoperfusion caused by obstructive coronary artery disease, which is possibly reversible after revascularization.^210^ This has long been an intriguing phenomenon in cardiology, and remains a controversial topic. Among imaging methods, positron emission tomography (PET) has the highest sensitivity and negative predictive value, while dobutamine stress echocardiography is most specific and has the highest positive predictive value per analyzed segment.^211^ Currently, there is ongoing conceptual reconsideration regarding how revascularization should be guided by prior analysis of myocardial viability and what would the benefit of doing so. The classic concept ascribed greater value to the presence of viability, regardless of coronary anatomy for revascularization. This strategy has been shown to improve ejection fraction, quality of life, symptoms, and ischemic heart failure; however, improvement in the ejection fraction per se did not necessarily translate into an improvement in survival. The current approach aims for a more myocardial-specific functional assessment, improving therapeutic planning towards a more rational and physiological revascularization. Therefore, there is a greater concern in preserving viable ischemic segments where the anatomy is favorable for surgical revascularization, thereby reducing the rate of fatal heart attacks and arrhythmias.^210^ Interestingly, percutaneous revascularization guided by viability studies has not demonstrated any benefit.^212,213^

### 20.1. Stress Echo Protocols for Assessment of Myocardial Viability

Prior to myocardial viability studies, a routine targeted transthoracic echocardiogram is performed. This will allow the examiner to understand the baseline parameters to be analyzed under dynamic conditions, as well as the quality of the echocardiographic window and the need to use ultrasound enhancing agents. Assessment of myocardial viability is performed using the low-dose dobutamine pharmacological stress echocardiogram protocol (starting at 2.5 or 5 mcg/kg/min, with increments to 7.5 and 10 mcg/kg/min every 3 to 5 minutes, up to 20 mcg/kg/min). The protocol can also be performed in patients who are on beta blockers. Although viability is assessed with low-dose of dobutamine, it is worth continuing the examination with higher doses (up to 40 mcg/kg/min, with or without atropine) to evaluate not only viability but also the presence of stress-induced myocardial ischemia, which greatly contributes to optimizing management. This of course applies only to those patients whose condition allows the protocol to progress further, preferably those whose ejection fraction is not greatly reduced due to marked hypokinesis of multiple segments in addition to akinetic areas; in these cases, the risk of the high-dose protocol is greater and the sensitivity of the method for diagnosing ischemia is reduced due to the marked, "balanced" abnormality of all walls of the left ventricle.

Segments akinetic at rest are deemed viable when they exhibit increased thickness (≥ 0.5 cm) and contractility (contractile reserve) during dobutamine stress. A biphasic response as the protocol progresses (improvement at low doses and worsening at higher doses of dobutamine, i.e., 20 mcg/kg/min or more) suggests a greater number of so-called ischemic viable myocardium and is the best possible response, suggesting there will be functional recovery after revascularization. This is especially true when viability, referred to as a biphasic response, is observed in four or more segments that are akinetic at rest in patients with LV systolic dysfunction. Generally, this group of patients shows better responses to myocardial revascularization, including improvement in ejection fraction, compared to those who only show improvement in contractility at both low and high doses of dobutamine (sustained improvement) or those who show worsening of contractility in hypokinetic regions at low-dose pharmacological stress^214,215^ (Figure 20.1).

**Figure 20.1:** Summary algorithm for management of hibernating myocardium. CABG: coronary artery bypass graft; LVEF: left ventricular ejection fraction; OMT: optimal medical therapy.

### 20.2. Assessment of Myocardial Viability by Longitudinal Strain

Assessing myocardial viability through analysis of global longitudinal strain (GLS) of the left ventricle on two-dimensional (2D) echocardiography is an active, relevant area of research. Measuring strain and strain rate (SR) by echocardiography is a challenging but promising technique for the assessment of resting ventricular function, myocardial viability, and ischemia during stress testing. At low doses of dobutamine, there is an increase in strain and strain rate in viable segments and a reduction in post-systolic shortening. Conversely, in segments with transmural involvement after infarction, there is only a slight, transient increase in SR, with no increase in GLS, and post-systolic shortening is increased rather than reduced. Figure 20.2 summarizes the main changes seen in echocardiographic myocardial deformation analysis.^215-217^ Despite interesting findings which add information to the method, studies are still needed to confirm the clinical benefit of guiding therapy on the basis of incremental myocardial deformation data; therefore, GLS still cannot replace existing techniques.

**Figure 20.2:** Main changes in myocardial deformation analysis by global longitudinal strain for assessment of myocardial viability.

Regardless of the clinical decision for surgical revascularization or medical treatment, the presence and extent of viable segments demonstrate a basis for therapeutic response such as cardiac resynchronization therapy or pharmacotherapy, not only myocardial revascularization.^218^

Therefore, patients with viable myocardial segments will have a better therapeutic response and a better prognosis when undergoing clinical or surgical interventions.

## 21. Stress Echocardiography with Ultrasound Enhancing Agents for Delineation of the Endocardial Border

Ultrasound enhancing agents (UEAs) – the more recent nomenclature for microbubble or ultrasound contrast – can and should be used in all stress modalities.

In treadmill stress tests, images are obtained at rest and immediately after exertion (ideally up to 90 seconds after the cessation of exercise, at which time the patient lies down and the images are acquired). When using a reclining or cycle ergometer, images are obtained continuously throughout the various stages of exertion, just as in pharmacological stress echocardiography. Therefore, the administration of UEAs differs according to the type of stressor.

It is well known that some patients lack a satisfactory echocardiographic window, which can compromise the quality and interpretation of the examination. In these cases, the use of UEAs, which allow perfect visualization of the borders of the walls of the heart, is essential. Detailed visualization of all segments is essential for the detection of CAD. Visualization of all segments at rest and during stress is also important to determine the extent of induced ischemia or the presence of previous infarction.^219,220^

Currently, there is a trend to avoid referring to these agents as "contrast agents" so as not to associate them with those used in conventional radiology or magnetic resonance imaging, which have infinitely greater toxic and allergenic potential than the agents used for ultrasound.^221^ For this reason, we will use the acronym UEA instead, as described above.

Stress echocardiography with UEA exhibits greater sensitivity, specificity, and diagnostic accuracy.^222,223^

Furthermore, UEAs can be cost-effective when used with appropriate indications, especially when two or more segments are poorly visualized on apical views,^223^ thus avoiding the use of other imaging modalities with higher cost and potential for renal toxicity or radiation exposure.

### 21.1. Ultrasound Enhancing Agents

As of the time of writing, only one UEA is registered and therefore available for use in Brazil: Sonovue (Bracco), which is composed of microbubbles generated from sulfur gas. These bubbles mostly have a diameter of < 5 micrometers and thus pass through the pulmonary capillaries, rapidly (in about 2 seconds) reaching the left side of the heart and delineating the borders of the left ventricular walls. These agents improve image quality and confidence in the interpretation of examination findings.^219-222^

There is a wealth of literature corroborating the safety of UEAs. Nevertheless, the potential for anaphylactoid reactions – although exceedingly rare – mandates the immediate availability of medications and equipment for cardiopulmonary resuscitation.^223^

#### 21.1.1. Administration

Most modern echocardiography equipment features specific presets for UEA delineation of the endocardial border and ventricular cavity opacification (rather than for perfusion assessment). Although UEAs can be administered as a bolus or continuous infusion, we believe the bolus method is more practical and yields excellent results. Enough UEA should be administered to opacify the heart homogeneously, without causing swirling at the apex or attenuation at the base. The echocardiographer should always use the device's factory-set UEA protocols, which should feature a low mechanical index (MI) (< 0.3), harmonics, or a nonlinear pulse sequence.^224,225^ In more modern systems, a very low MI (< 0.2) avoids destruction of the microbubbles, enhances the microbubble signal, and reduces artifacts, allowing for excellent delineation of the endocardial borders.

Some echo labs use UEAs in all SE examinations to improve accuracy; however, cost-effectiveness is questionable in this setting, since in many centers only around 20% of cases appear to actually require such enhancement.

During pharmacological stress associated with UEA, contrast-specific stress presets are used for endocardial border delineation. When we wish to evaluate perfusion (which depends on specific, more sophisticated software, not the same used for endocardial border analysis), we switch from the endocardial border delineation preset to the appropriate preset for myocardial perfusion (pulse inversion, power modulation, or cadence pulse sequencing). It is worth noting that myocardial perfusion analysis requires specialist expertise and a greater learning curve than endocardial border analysis.

For UEA use, a Jelco-type peripheral IV catheter with a 2-way infusion extension set or, preferably, a 2-way stopcock, leaving one of the lines in a straight line for the administration of the UEA. The UEA is administered before the start of the test (for baseline image acquisition) and before/during each step of the stress protocol, to ensure comparable images are obtained. For the first injection, we administer a bolus of approximately 0.5 mL, which may or may not be followed by a slow saline flush (rapid administration of the UEA bolus and/or saline flush will cause hyperechoic signal, with shadowing in the mid-distal regions of the heart). During the various stages of dobutamine infusion, we no longer perform a saline flush after each bolus.

During treadmill SE, we prefer to place the IV line in the patient's left upper extremity (preferably the antecubital fossa). As per our institutional protocol, the UEA is administered approximately 15-20 seconds before peak exertion and before the patient is moved to the examination table for acquisition of immediate post-exercise images. Here, again, there is no need to flush the line after administering the UEA. Baseline images are obtained as soon as the patient lies on the examination table.

Quad-screen mode than places the images side by side to allow perfect interpretation. Ideally, the UEA should therefore be administered at every step of the stress protocol (Figure 21.1).

**Figure 21.1:** Side-by-side echo images in quad-screen format, allowing for perfect interpretation of the different stages of the examination.

It is worth noting that, when using UEAs in stress echocardiography, the ideal approach is to begin acquiring images from the apical view. This window may even be the only one used to obtain images of all left ventricular walls, because the left ventricular apex should be well opacified and is the area most affected by the destruction of bubbles caused by insonation; therefore, it should be examined first.

## 22. Stress Echocardiography with Ultrasound Enhancing Agents for Myocardial Perfusion Analysis

The volume of blood in the coronary circulation is mostly (about 90%) found within the capillaries. Just as UEAs opacify the left ventricular cavity upon insonation, microbubbles within the capillaries cause a speckled intramyocardial appearance. The intensity of this brightness thus correlates with the volume of blood within the microvascular system.

Following UEA administration, myocardial perfusion (MP) is assessed in all three apical views of the left ventricle. This can be done either during continuous infusion of the agent or, more routinely, after bolus injections.

When performing concomitant analysis of myocardial contraction and perfusion, the microbubble destruction-replenishment method is used, employing a very low MI, especially when using specific software for real-time perfusion analysis (MI < 0.2), as is currently considered optimal (rather than standard endocardial border delineation software). The replenishment technique is important for determining the rate of myocardial wall refilling and the presence or absence of ischemia.

After administration of the UEA, as soon as adequate LV opacification is achieved, a high-energy pulse (flash; high MI: 0.8 – 1.2)^226^ is delivered, which will destroy the microbubbles in the intramyocardial capillary blood volume while relatively sparing any intracavitary microbubbles. If there is limiting obstruction of coronary flow in the epicardial arteries and/or microcirculation, the end result is attenuation or even absence of myocardial brightness in the affected segment. (Figure 22.1).

**Figure 22.1:** A 60-year-old woman undergoing dobutamine stress echocardiography with ultrasound-enhancing agent. She presented with chest discomfort and ST segment depression in precordial leads on the ECG tracing. Note the area of impaired myocardial perfusion at the apex of the left ventricle, with local hypokinesis (arrows). Stress echocardiography positive for apical myocardial ischemia.

This analysis is routinely performed during the examination; however, offline analysis has proven superior because it allows objective assessment of the two components that make up MP: capillary blood volume (component α) and capillary blood velocity (component β). MP is determined by the product of these 2 components (α × β), as demonstrated in Figure 22.2. A reduction in the α component may be related to infarcted areas or significant restriction of microvascular flow, while a decrease in the β component correlates with reduced capillary flow, associated with obstructive CAD or MINOCA. In MINOCA, there is a delay and MP deficit, but with preserved myocardial thickening, since there is no subendocardial involvement.^227^ The diagnosis is made by perfusion-contraction analysis, where MP and regional wall motion are evaluated together, as well as by demonstrating a microvascular flow reserve <2, preferably determined on vasodilator stress echocardiography.^228,229^ The β component has greater sensitivity than the α component for detecting CAD.^230^ As a general rule, normal MP is based on myocardial refill (full restoration of the signal measured in decibels) in under 5 seconds at rest (for a heart rate of 60 bpm) or in up to 2 seconds during stress (for a heart rate of 120 bpm). This analysis should be performed at end-systole.^230^

**Figure 22.2:** Example of offline myocardial perfusion analysis. Apical 2-chamber view of the LV. Note extensive perfusion deficit on the anterior wall (red). A, capillary blood volume analysis (α component). B, capillary blood velocity analysis (β component). C, microvascular blood flow analysis (α × β).

MP assessment significantly enhances the conventional subjective assessment of myocardial regional wall motion/thickening (the cornerstone of stress echocardiography), thus increasing the sensitivity of the method for detecting CAD, as demonstrated by several studies.^231-233^ It also compares favorably to SPECT scintigraphy in terms of sensitivity for detecting obstructive CAD.^234^ One of the reasons is precisely the fact that nuclear perfusion imaging assesses capillary blood volume alone,^235^ while echocardiographic analysis of MP also allows evaluation of capillary blood flow velocity, which is known to be a more sensitive marker for detecting CAD.^230^

Accuracy studies for CAD detection have shown that stress echocardiography combined with MP analysis has a sensitivity of 83% and specificity of 79% with vasodilator protocols^236,237^ and a sensitivity and specificity of 88% and 77%, respectively, when using dobutamine or physical stress.^237^

In addition to enhancing detection of CAD, SE analysis of myocardial perfusion is a good prognostic indicator. A properly performed stress test negative for myocardial ischemia, coupled with MP analysis demonstrating normal perfusion, carries an excellent prognosis, with a very low incidence of major adverse cardiovascular events or death in the short to medium term.^238^ Myocardial perfusion imaging is also superior to clinical data and LVEF for predicting ventricular remodeling over time.^239^

MP analysis can also assist in assessing myocardial viability. Histologically, the basis for the viability of myocardial tissue (and, thus, the possibility of functional recovery) lies in the relationship between the density of microvascularization and the quantity and extent of tissue collagen (fibrosis). Therefore, extensive areas of marked perfusion deficit denote transmural involvement and reduced myocardial viability, a finding comparable to those of CMR using gadolinium contrast.^240,241^

Recommendation:

- In echo labs having staff trained to perform it, the incorporation of contrast echocardiography with myocardial perfusion analysis to stress echocardiography adds power to identify and stratify obstructive CAD;
- Myocardial perfusion analysis can aid in analyzing the myocardial viability of segments that clearly do not show improved contractility with administration of dobutamine.

## 23. Stress Echocardiography with Myocardial Strain Imaging in Ischemic Heart Disease

The introduction of speckle-tracking echocardiography (STE) has enabled the quantitative assessment of myocardial deformation (or strain) during stress echocardiography. Strain imaging provides a novel approach to assessment of myocardial function that is both objective and quantitative, and has several advantages over conventional echocardiography. The aim of this chapter is to provide an overview of the current state of knowledge regarding the utility of GLS (global longitudinal strain) and RLS (regional longitudinal strain) analysis in stress echocardiography, with particular focus on ischemic heart disease.

### 23.1. Speckle Tracking-Derived Strain Versus Tissue Doppler-Derived Strain During Stress Echocardiography

As previously mentioned, SE is used to assess CAD by inducing physiological stress on the heart through exercise or pharmacological stimulation with dobutamine or dipyridamole. Comprehensive imaging, which includes assessment of myocardial strain, has been shown to improve the accuracy of SE for CAD diagnosis and prognosis.

Tissue Doppler imaging and its derived parameters, such as myocardial strain and strain rate, have provided alternative approaches to quantify regional wall motion at rest or during stress with greater accuracy.^49,242^ Tissue Doppler is feasible during stress testing, but requires a high frame rate of at least 140 frames per second. Its main limitation is that the peak velocity and strain variables depend on the angle between the incident ultrasound beam and the myocardial wall; therefore, the apical region is not evaluable^243^ through Doppler imaging.

Non-Doppler 2D strain imaging is a novel echocardiographic technique used to measure strain and strain rate. It employs a two-dimensional analysis of motion by plotting points on the ultrasound image.^244^

Currently available software allows for the spatial and temporal processing of images, as well as the recognition and selection of these points (unique fingerprints of the heart, known as "speckles") on the echo image. The displacement of each cluster of speckles (pixels) represents local tissue motion. Tracking these pixels allows calculation of tissue velocity, strain, and strain rate using 2D ultrasound. Non-Doppler 2D strain imaging is a simple procedure that requires acquisition of only one cardiac cycle and can be easily processed and interpreted after data acquisition.^245^

Ischemia is defined as a regional reduction in myocardial thickening, which can also result in a delay in the onset and termination of systolic contraction.

It bears stressing that ischemia is not only a spatial phenomenon, but also a temporal one; experimentally, with a coronary occlusion time of 40 seconds, myocardial thickening not only decreases but is delayed and continues into the post-ejection or diastolic period (a phenomenon known as tardokinesis, post-systolic contraction, or post-systolic strain).

Unfortunately, the human eye does lack sufficient temporal resolution to detect subtle contractile changes in real time. Conversely, strain can be automatically tracked during the cardiac cycle by measuring the distance between two pixels on a myocardial segment. Numerous small, clustered regions of myocardial speckles (kernels) are tracked, and the software averages their movements before extracting regional curves and the global average. The quality of tracking can be checked for each segment, with manual adjustment of the region if necessary. This novel method allows for the simultaneous evaluation of the three components of myocardial strain: radial, longitudinal, and circumferential. In practice, apical views are used to obtain the GLS and strain rate, while parasternal short-axis views are used to evaluate radial and circumferential strain.^246^

Hanekom et al. compared the use of 2D strain versus tissue Doppler imaging for detecting ischemia during dobutamine stress echocardiography in 150 patients. They found that speckle tracking was a feasible means of measuring strain during DSE and had similar accuracy to tissue Doppler in detecting ischemia in the LAD territory. However, 2D deformation was not as accurate as tissue Doppler in detecting ischemia in the territories supplied by the circumflex and right coronary arteries.^247^

In practice, there is no competition between Doppler-based strain and speckle tracking-based 2D strain imaging; rather, they supplement one another, each having its own advantages and limitations. Doppler-based strain imaging should be used with higher heart rates, lower-quality windows, and to assess basal and medial velocities, and 2D strain imaging when wishing to prioritize accuracy and apical analysis of the left ventricle (LV). Two-dimensional strain is recommended for heart rates between 40 and 100 bpm, high-quality acoustic windows, and for longitudinal and circumferential assessment of the LV, as well as rotation and torsion, prioritizing the automatic nature of analysis and shorter study time.

### 23.2. Two-Dimensional Strain During Stress Echocardiography: Diagnostic and Prognostic Value in Coronary Artery Disease

Reant et al. investigated the applicability of 2D strain for detecting ischemia during dobutamine stress echocardiography in swine. The main results of the study demonstrated that 2D strain is as reliable as sonomicrometry for assessing regional LV function. The authors demonstrated that longitudinal and circumferential abnormalities precede a decrease in radial strain in the presence of ischemia. This finding can be explained by the fact that subendocardial myocardial fibers are primarily oriented longitudinally, which makes longitudinal function more sensitive to ischemia and, therefore, is affected earlier than radial function.^248^

Elamragyy et al. conducted a prospective study of 101 patients with an intermediate probability of CAD risk. The patients were evaluated by DSE and speckle-tracking imaging. All underwent coronary angiography within 1 month. The sensitivity and specificity of DSE for detecting coronary artery disease were 79.6% and 92.3%, respectively. By adding a GLS cutoff of ≤ −20.5, the sensitivity and specificity for diagnosis of CAD increased to 95.9% and 84.6%, respectively.^249^

Other authors have also demonstrated that GLS measured during DSE had greater sensitivity but lower specificity compared to analysis of regional wall motion abnormalities.

Of particular interest is a study by Uusitalo et al., which evaluated strain and post-systolic index in 50 patients with intermediate risk for CAD after dobutamine infusion during the initial recovery phase. The results showed an increase in the diagnostic accuracy of the examination. Among patients with CAD, strain was −16.4 ± 4.8 compared to −19.3 ± 3.9 in healthy individuals (p < 0.001), with a significant increase in post-systolic index.^250^

The results of the Uusitalo study suggest that incorporating strain and post-systolic index measurements during early recovery after infusion of 20 mcg/kg/min of dobutamine may improve the diagnostic accuracy of DSE in patients at intermediate risk for CAD.

Although viability assessment remains controversial, there is a large body of literature that supports the idea that the behavior of GLS and SPI can be used to recognize viable myocardium based on its response to low-dose dobutamine.

Strain imaging during DSE is easier to perform compared to exercise echocardiography because it is less affected by respiratory motion. DSE also allows for more consistent image acquisition due to the stable increase in heart rate and contractility, which can improve the accuracy and reliability of myocardial strain measurements.

Strain imaging during DSE has been extensively studied, but results regarding viability and applicability of imaging obtained during exercise echocardiography are still lacking. This may be due to technical difficulties associated with image acquisition during exercise, as well as to increased patient movement and respiratory artifacts, which can affect the accuracy of strain measurements.

Currently, there is little data – and only from small series – comparing conventional image acquisition versus GLS and RLS measurement during stress echocardiography on a treadmill or reclining ergometer.

The feasibility of speckle-tracking during exercise stress echocardiography was evaluated in 67 healthy adult patients, with satisfactory results. The study stratified patients by age group and sex, with groups divided into 23-to-35, 35-to-55, and 55-to-80 years old. The results showed that GLS increased significantly during exercise across all age groups, regardless of sex. The mean absolute increase in GLS was 5.3%.^251^ This suggests that strain echocardiography may be a useful tool for assessment of cardiac function during stress testing.

Currently, it is possible to demonstrate, in patients with a good acoustic window, high feasibility of GLS assessment at rest (99%) and in the immediate (<30 s) post-exercise period (97.5%) on a reclining ergometer; the absence of increase or decrease in longitudinal strain of the apical segments was consistent with the presence of visually detected ischemia in 78.6% of examinations, while apical longitudinal strain increased in 96.2% of cases negative for ischemia according to standard subjective assessment of regional wall motion abnormalities.

However, strain analysis was only reliable for segments irrigated by the LAD. Therefore, 2D strain analysis was feasible in the immediate post-exercise period, but high heart rates led to nonspecific findings in the mid- and basal inferolateral segments.^252^

Key reasons why only the apical segments could be analyzed reliably may include: 1) 2D axial resolution is generally very good; 2) lateral resolution decreases as depth of field increases. If the lateral resolution is low, the consequence is a "blurred" image that does not allow speckles to be tracked as easily in distant fields; 3) translational movement of the heart during exercise increase the difficulty of tracking the basal and mid-cavity segments; and 4) at elevated heart rates, frame-to-frame changes are abrupt and angular independence begins to be lost with a lower line (and information) density, which remains sufficient for tracking in the near field, but is insufficient for distant fields.^252^

Dipyridamole stress echocardiography is one of the several protocols recommended for diagnostic and prognostic elucidation of CAD. However, its use in the echo lab is not widespread, not for truly scientific reasons but rather due to a lack of knowledge or for personal, geographical, or economic reasons, as well as limited availability. Given at high doses over rapid infusion times with the addition of atropine and/or isometric handgrip exercise, dipyridamole provides a level of diagnostic accuracy comparable to that of dobutamine, with a three times lower rate of serious complications.^57,253^

In a retrospective study by Lowenstein-Haber D et al. involving 150 patients referred for dipyridamole stress echocardiography, apical and LAD coronary reserve were assessed and visual analysis of regional wall motion was performed.^254^ Abnormal regional apical longitudinal strain and low coronary reserve during dipyridamole stress were the only predictors of worse outcomes, regardless of visual analysis of regional wall motion abnormalities.

A previous report from the same group also demonstrated that 2D strain analysis significantly improved the sensitivity of dipyridamole stress echocardiography for detecting coronary artery disease compared to standard analysis of myocardial contractility (83.3% vs. 50%; p = 0.001), without compromising specificity (100%).^255^

Identical conclusions were drawn in investigations by other groups, which have also proven the added value of measuring two-dimensional strain alongside conventional visual analysis of regional wall motion.

### 23.3. Which Strain is Most Useful in stress echocardiography? Global or regional? Longitudinal or Circumferential? And What is the Importance of Post-Systolic Thickening?

Most studies that evaluated the feasibility and accuracy of GLS assessment during exercise stress echocardiography were performed on small groups of healthy individuals.

Although RLS analysis can provide more detailed information about specific areas of the heart, it can also be technically more difficult and complex to perform and interpret. On the other hand, GLS provides a more complete assessment of overall myocardial function and has been shown to be a powerful predictor of adverse cardiovascular outcomes.

However, it is also important to note that simply observing the magnitude of strain may not reflect the entirety of the situation. The timing of peak strain, both global and regional (percent post-systolic contraction and deformation), and measures of mechanical dispersion are of paramount importance and can provide information about abnormalities in myocardial mechanics, as can be seen in the following example (Figures 23.1 and 23.2).

**Figure 23.1:** A 50-year-old man with positive risk factors reported interscapular pain at the onset of exercise. Note decrease in strain and post-systolic deformation at peak exertion (white arrows). Coronary angiography: 2 significant blockages along the left anterior descending artery.

**Figure 23.2:** Little increase in global strain with dobutamine stress, but marked dispersion of the curves and post-systolic contraction are indicative of ischemia. Upper left corner: polar map showing a baseline strain of 21.6%. Middle: Strain during dobutamine stress is 21.4%. Right: Strain during recovery is 20.8%. Midline: In blue, the percentage of post-systolic strain that increased sharply with low-dose dobutamine. Below the respective curves. Patient with severe triple-vessel coronary artery disease.

Dipyridamole stress echocardiography is an excellent modality for simultaneously assessing regional wall motion and the behavior of GLS and SPI, adding diagnostic and prognostic information. GLS and RLS measurement added to dobutamine stress increases its accuracy for detecting myocardial viability, and strain rate assessment by M-mode Doppler can be very helpful in visualizing the post-systolic shortening phenomenon, which can also be visualized in the curves and target with 2D strain showing dyssynchrony/ischemia if it occurs during the test.

Establishing normal values for myocardial strain during stress echocardiography in healthy individuals can help better interpret strain data in patients with suspected cardiovascular disease.^256^

LV GLS is not homogeneous; there is a gradient from the basal segments to the apex, known as the basal-to-apical gradient. This gradient is observed in both circumferential and longitudinal strain, with lower values in the basal segments and higher values at the apex. A gradient between the subendocardium and the epicardium has also been found.

Although the literature provides reference values for apical, mid-cavity, and basal segments, the lack of homogeneity in regional strain significantly complicates subsequent analyses. Nevertheless, regional strain analysis can help confirm ischemia in stress echocardiography.

Three short-axis views at the basal, mid-cavity, and apical levels should be used to assess circumferential strain and twist and torsion. However, acquiring an apex view identical to the baseline (resting) image is not always feasible during stress echocardiography, making it less suitable for this type of assessment.

### 23.4. Does Strain Have Ischemic Memory?

In a study of 101 segments exhibiting behavior deemed ischemic on visual analysis, the mean GLS at rest was −22.3 ± 4.3%; at peak exercise, it decreased significantly to −16 ± 3.2%; 3 minutes after recovery, values were higher than those obtained at rest (-24.3 ± 5.1%). Thus, changes in GLS and SPI did not persist after recovery of the visually detected regional wall motion abnormalities.^257^

This behavior is consistent with the findings of an experimental study from the University of Osaka, which demonstrated that the recovery of strain and strain rate was rapid and complete relative to occlusion time. However, post-systolic indices were slower to recover.

The authors conclude that if analysis of GLS and SPI is performed several minutes after exercise at heart rates below 100 bpm, when there are no more derangements of contractility, and if the ischemia time was short, strain recovers rapidly, even at supranormal values (Figures 23.3 and 23.4).

**Figure 23.3:** A 57-year-old woman with stable angina. Normal global longitudinal strain at rest with multiple, extensive dyssynergies at low workload, deterioration of peak longitudinal strain, and significant post-systolic strain. Coronary angiography: Severe distal obstruction of the left main; severe obstructions of the left anterior descending artery, right coronary artery, and circumflex artery. Rest global longitudinal strain: 19.5% Stress: 9.5%.

**Figure 23.4:** A 65-year-old patient. Stress echocardiography positive for ischemia in the apical, anterior septal, anterior, and inferior segments, with a global longitudinal strain of −20.7% at baseline, −12.6% at peak stress, and −21.1% in the recovery period. Coronary angiography: Severe obstruction of the right coronary and left anterior descending arteries. This case exemplifies that resting strain does not predict the outcome of stress echocardiography and has no ischemic memory.

This transient behavior explains why patients with angina and no regional wall motion abnormalities exhibit resting 2D strain within the normal range outside of an anginal episode (if it is brief).

### 23.5. Can Baseline Global Longitudinal Strain Predict Coronary Artery Disease in the Absence of Visually Apparent Wall Motion Abnormalities During Stress?

There are reports in the literature that resting GLS can predict the presence of LMCA involvement and triple-vessel disease in patients without regional wall motion abnormalities. According to Jin-Oh Choi et al., global 2D strain was lower in 108 patients with triple-vessel or LMCA disease, even in the absence of regional wall motion abnormalities (with a cutoff point of −17.9%), with a sensitivity and specificity of 79% compared to normal.^258^

Montgomery reports that GLS analysis can detect moderate to severe CAD with comparable accuracy to the results of visual analysis during stress echocardiography, with a GLS < −20% having an 80% negative predictive value for lesions > 50% and 90% for lesions > 70%.^255^

On the other hand, in a group of 124 patients without regional wall motion abnormalities at rest undergoing coronary angiography, GLS did not allow prediction of the outcome of stress echocardiography nor of the presence of significant CAD.^260^

### 23.6. Limitations

Although 2D strain imaging during stress echocardiography has demonstrated its benefit in detecting subtle changes in left ventricular function, it has several limitations. Technical factors such as image quality, respiratory motion artifacts, and patient position can affect the accuracy and reproducibility of strain measurements. Furthermore, there is currently no standardized method for strain analysis in stress echocardiography, which may limit comparability and increase the heterogeneity of results across different studies.

Although experiments have shown promising results, larger studies are needed to establish the diagnostic accuracy, sensitivity, and specificity of strain imaging in detecting myocardial ischemia during exercise.

Furthermore, the integration of strain measurements with other parameters obtained during stress echocardiography, such as visual analysis of wall motion abnormalities, B-lines, diastolic reserve, contractile reserve, and chronotropic reserve – the true art of stress echocardiography – is not performed in most laboratories worldwide, despite its proven utility for various heart diseases, as demonstrated by the results of the Stress 2030 study led by Dr. Eugenio Picano et al.^261^

It is also important to keep in mind that the outcome of these new techniques is operator-dependent, and that proper technology, experience, and training are necessary for their rational use. In trained hands, using the right technology, with very good image quality, this limitation is not so critical for stress echocardiography.

High heart rate makes 2D strain analysis difficult during exercise, while with high-dose dobutamine, the temporal resolution is insufficient for speckle tracking in the basal and mid-cavity segments of the myocardium.

Although strain imaging has shown potential benefits in stress echocardiography, including greater accuracy in detecting myocardial ischemia and identifying subclinical left ventricular dysfunction in asymptomatic patients, much research remains to be done to fully define its role in routine clinical practice.

## 24. Stress Echocardiography with Myocardial Strain Imaging Beyond Ischemic Heart Disease

One of the applications of GLS during SE is the determination of contractile reserve (CR), supplementing information provided by the change in ejection fraction. The normal response is an absolute two-point increase in left ventricular GLS during stress compared to rest.^262^

In primary mitral regurgitation of moderate or greater severity without ventricular dysfunction, Magne et al. demonstrated that the absence of CR as measured by GLS during stress testing is an important, independent predictor of future cardiac events.^128^

An interesting study by Arbucci et al. demonstrated that the absence of CR as measured by GLS during exercise testing in asymptomatic patients with significant aortic stenosis was associated with a higher event rate and lower survival. Furthermore, assessment of CR by GLS was the best predictor of events compared to the traditional assessment of CR by the ejection fraction.^263^

In LFLG aortic stenosis with reduced ejection fraction, the assessment of GLS at rest and during dobutamine stress added important prognostic information to traditional echocardiographic parameters. As demonstrated by Dahou et al., a GLS of less than 9% at rest and less than 10% during stress was associated with higher mortality in both conservatively and interventionally treated patients.^264^

Distinguishing ischemic from nonischemic cardiomyopathy in the presence of significant ventricular dysfunction is challenging on SE. Anatomical assessment is often essential. However, in patients with mild or moderate LV dysfunction, sustained increases in contractility and GLS suggest the presence of nonischemic cardiomyopathy.^99^ It is worth noting that this response is also observed in athletes during SE, generally with a supranormal increase in strain, and is of great value in distinguishing between athlete's heart and cardiomyopathy.^265,266^

In HCM, the measurement of GLS during SE helps identify patients at higher cardiovascular risk. In addition to the absence of CR, the presence of dyssynchrony during exercise is another relevant parameter to be analyzed in this population.^267^

The greatest difficulty in assessing GLS during SE is differentiating between pathological strain reduction and reduction secondary due to limitations inherent to the technique. Tachycardia, hyperventilation, inadequate image quality, variability in results between software programs, and operator inexperience are some of the challenges for measuring strain during SE (Table 24.1), as previously mentioned.

**Table 24.1:** Limitations to global longitudinal strain measurement during stress echocardiography and potential solutions

Problems	Solutions
Additional time required for SE / need to acquire additional images	Routine use of all three apical views in the stress protocol
Inappropriate image quality	Proper adjustment of depth, sector width, and frame rate (>60 fps) Avoid acquiring clips with heart rate variability greater than 10% Check ECG quality and avoid acquiring images during/after premature ventricular contractions
High heart rate	When performing ESE, use images obtained during immediate recovery (within the first minute after exercise cessation).

ECG: electrocardiogram; ESE: exercise stress echocardiography.

In non-ischemic disease, global strain is more valuable than regional strain, which facilitates its application in practice. The negative influence of tachycardia and hyperventilation on assessment of strain during physical exercise can be reduced by acquiring images at immediate recovery (up to the first minute after cessation of exercise). Dipyridamole stress, due to its lower chronotropic influence and high quality two-dimensional images, allows for much easier evaluation of GLS.

Strain measurement during SE is a reality, and should be done whenever possible. Its quantitative, independent, objective nature makes this method a promising tool to be incorporated into SE.

The utility of left ventricular GLS for evaluating CR has made this an important parameter, and regardless of the condition studied, absence of CR is associated with worse clinical outcomes and a higher rate of events. It is worth noting that the feasibility of CR assessment by GLS during SE has been greatest in studies using vasodilator stress, followed by dobutamine and, lastly, exercise.

## 25. Three-Dimensional Stress Echocardiography

### 25.1. Three-Dimensional Technology and its Applications in Echocardiography

Three-dimensional echocardiography allows real-time visualization of 5 physical dimensions of cardiac structures, considering the 3 orthogonal planes of anatomical definition, cardiac flow images, and the temporal dimension.^268-271^ It thus adds important morphofunctional information when compared to two-dimensional echocardiographic analysis.^268-271^ The first experiments with 3D ultrasonography imaging date back to the 1950s, when interest was focused on observing the human orbit. The first images of cardiac structures were obtained in the 1970s. Since then, several incremental advances to 3D echocardiography have been made,^48,272-281^ up to the current state of the art, in which real-time three-dimensional multi-angle matrix-array imaging of the heart is possible. This has become reality through the use of nanotechnology, digital structural observation, and undeniable advancements in the speed of morphofunctional analysis since the development of algorithms for matrix and voxel identification. Simultaneous real-time analysis of multiple planes of the heart is a perfect fit for stress echocardiography, enabling examinations to be performed with shorter acquisition times, high reproducibility, and less interobserver variability, while also providing structural information from new spatial projections.^48,272-281^ Three-dimensional acquisition also reduces the issue of image foreshortening during acquisition, which can be problematic in two-dimensional echocardiography. Currently, 3D stress echocardiography can be performed using biplanar imaging guided by three-dimensional observation (full-volume) or matrix-array imaging (X-plane). Three-dimensional stress echocardiography is primarily dependent on the quality of the initial image, and is greatly enhanced by the use of UEAs. However, UEA administration can sometimes lead to a reduction in temporal resolution, which is already a limitation of 3D imaging, despite significant improvement in the delineation of endocardial borders in 3D stress studies with harmonic imaging and suboptimal quality.^278^ For this reason, their use in conjunction with 3D stress echo is not yet widely recommended. Two-dimensional image acquisition allows analysis of a higher number of frames (higher frame rate) and assessment of myocardial perfusion (when used with ARUs); it also uses the frame rate, although lower, whereas 3D analysis allows evaluation from a larger sample volume (3D Full-volume) or real-time biplanar analysis (3D X-plane). However, 3D full-volume analysis can result in acquisition artifacts ("stitch") due to voxel translational movement, has low voxel rates and low temporal and spatial resolution, which limits analysis of myocardial perfusion. In comparison, the 3D Xplane technique has a better frame rate.

New models of echocardiography systems are becoming available for clinical use, with the possibility of employing novel transducers and algorithms that allow for better image quality, with a higher three-dimensional volume rate, expanding the future applications of 3D imaging.

Models for 3D stress echocardiography include 3D multiplanar analysis with simultaneous observation of the long and short axes of the left ventricle, plus volumetric data from apical views. Acquisition of 3D apical full-volume images should be performed with the transducer at the LV apex, with the patient holding their breath during acquisition to minimize potential image stitching resulting from cardiac translation, with a sampling volume large enough to analyze the entire LV but without excessive sector aperture, which results in a lower volume rate, during ECG recording. Occasionally, arrhythmias such as atrial fibrillation can cause errors in 3D analysis. Hyperventilation during image acquisition can also hinder obtaining the best image quality.

In future, 3D stress echocardiography will involve the use of new image interpretation algorithms and smaller transducers, with single-beat image acquisition and a higher voxel count, leading to improved image quality (particularly of lateral images), reduce stitching, and shorten examination time.

### 25.2. The Application of Three-Dimensional Echocardiography in Stress Echocardiography

The possibility of using real-time three-dimensional imaging for stress echocardiography arises naturally when considering its advantages in capturing images and interpreting ischemic phenomena and changes in ventricular geometry.

Peteiro was one of the first echocardiographers to compare the performance of 3D versus 2D echocardiography for exercise-induced ischemia,^282^ as did Yang et al.,^274^ using the dobutamine protocol. These initial studies demonstrated sensitivity and specificity similar to standard 2D imaging protocols, with the advantage of ease of image acquisition and shorter acquisition time with 3D echo.

Badano et al.,^275^ in a cohort of 44 patients who underwent angiography, demonstrated that the sensitivity and specificity of 3D echo were comparable to those of the 2D technique when using equipment with higher frame rates, and that 3D imaging compared favorably when analyzing apical changes in the territory of the left anterior descending artery. The authors suggest 3D dimensional echocardiography is broadly equivalent to conventional 2D echocardiography, but with advantages in some relevant aspects.

We used the ease of three-dimensional imaging in stress echocardiography.^283^ The first advantage lies in using the three-plane simultaneous method (Tri Plane), which provides apical 4-chamber, apical 2-chamber, and long-axis views. Unfortunately, this feature is not offered by all manufacturers of 3D devices. Proper device setup allows us to automatically view the other windows – with a satisfactory frame rate, typically above 30 frames per second and commonly reaching 60 frames per second – when we obtain a good quad-camera window. Obtaining only one window to view the complete frame using the 3D technique reduces the clip acquisition time to one-third, thus reducing examination time.

When performing examination during the Balke exercise protocol, images can be captured at any point during exertion or after termination of exertion. Typically, images are obtained at rest, low load (20% increase in resting heart rate), peak exertion (submaximal heart rate or 85% of age-predicted maximum heart rate), and recovery. Each stage of the SE comprises only one apical window, focused on the 4-chamber view. This reduces repetitive strain on the examiner's upper limb, which is spared by avoiding rotation to acquire the two subsequent standard apical views (Figure 25.1).

**Figure 25.1:** Single apical window allowing analysis of all walls of the left ventricle, saving examiner time and effort.

We do not routinely use multislice imaging, which provides multiple cross-sectional views of the ventricle, but it can be used for cases deemed questionable based on previously acquired 3D images. The multislice technique allows millimeter-by-millimeter "cross-sections" of the LV to be obtained, providing a more detailed and thorough assessment of regional wall motion by comparing the LV contraction at each stage of the SE.

Some echocardiography laboratories use a protocol that combined 2D Tri Plane acquisition with real-time 3D images, using the capture and analysis protocol of the former and analyzing images of the latter separately.^277^ We also do this at our service, primarily when teaching the technique to novices. By handling both formats, the examiner can choose the image that best represents the changes elicited by physical or pharmacological stress. This process can aid the examiner in adapting to 3D mode, with its lower frame rate but better interpretation of volumetric changes.

Obtaining 3D images requires adjustments to the echocardiography equipment to achieve the best volume rate per second. By working with the smallest possible depth of field and aperture, rates above 24 volumes per second can be achieved. The possibility of rotating the axes of the LV for more focused analysis at the apex or base, as well as rotation for analysis of regional wall motion in lateral views, allows a superior interpretation of contractility compared to conventionally acquired images (Figure 25.2).

**Figure 25.2:** Three-dimensional imaging in stress echocardiogram.

With its ability to acquire the entire LV volume in a single beat, real-time 3D echocardiography offers substantial promise to become the gold standard for assessment of patients with suspected CAD.

Increased productivity with a shorter examination time and greater comfort for the examiner are significant advantages. The implementation and analysis of 3D imaging requires some adaptation.

We believe that, for high-volume centers performing a large number of SEs, acquiring equipment capable of 3D stress imaging can be a very interesting resource to further optimizethe service dynamics.

## 26. Stress Echo Cardiography in the Emergency Department: Indications and Applications

Chest pain is one of the main reasons for seeking care at hospital emergency departments (EDs). In the United States alone, 7 million yearly ED visits have a presenting complaint of chest pain, with 30% of these categorized as acute coronary syndrome (ACS). It is estimated that up to 4% of patients are wrongly discharged from the ED due to atypical presentation or a nondiagnostic ECG.^284^ In an era of rising healthcare costs, hospital stays of several days to "rule out" ACS are no longer sustainable, requiring effective assessment protocols and workflows.

For patients at the extremes of the coronary risk spectrum, screening is straightforward; those with well-defined ACS are referred directly for cardiac catheterization, while those with a very low probability of coronary ischemia are discharged outright. The greatest challenge lies in those patients who have low to intermediate cardiac risk and lack clear evidence to support an ACS diagnosis, whether due to a nondiagnostic ECG or negative acute-phase cardiac biomarkers. In this non-negligible patient population, the diagnostic dilemma can be costly and often frustrating.

### 26.1. Utility of Stress Echocardiography in the Emergency Department

ED echocardiography allows assessment of cardiac function at rest, as well as detection of important differential causes of acute chest pain (such as aortic dissection and pulmonary embolism), significantly reducing evaluation time. The use of stress modalities, which may be pharmacological or physical depending on the patient's characteristics, provides a quick, effective, and important tool for noninvasive risk stratification.

Among the main limitations of the method is image quality, which can be affected by factors such as obesity, underlying lung disease, or technical difficulties. To overcome this barrier, such as in cases with an unfavorable window and more than two segments not adequately visualized, an ultrasound enhancement agent (UEA) can be used to improve the accuracy of the examination.

In 2007, the results of the ASSENCE (Assessment of cost-effectiveness of Several Strategies of Early diagnosis in patients with acute chest pain and Non-Conclusive Electrocardiogram) trial were published. This 10-center randomized clinical trial compared the cost-effectiveness of early dobutamine + atropine stress echocardiography versus standard assessment and exercise stress testing. Patients with ECGs that were not diagnostic of ACS, negative myocardial injury markers at baseline, and who were able to undergo stress testing were included. The primary objective was to assess cost-effectiveness, taking into account the length of hospital stay during initial admission and subsequent hospitalizations, inpatient and outpatient diagnostic procedures, and treatments. Secondary outcomes included the frequency of early and late major adverse cardiovascular events (MACE), readmission, and coronary revascularization. Of 110 patients in the stress echo arm, 90 (82%) were discharged outright, versus 78 of 89 (88%) in the exercise stress test arm. For these patients, overall costs over 2 months were 39% lower in the stress echo arm compared with the exercise stress test arm (US$1,029 vs. US$1,684; p = 0.005). In patients who required hospitalization, costs did not differ significantly between diagnostic strategies. Thus, the ASSENCE trial confirmed the general trend that adding imaging to non-image diagnostic strategies as standard of care is cost-effective. This cost-effectiveness has been attributed to fewer late events (none in the echo arm vs. 11% in the exercise stress arm), including readmissions, ACS, and late percutaneous intervention, after negative stress ECG.^285^

In the same year, Jeetley et al.^286^ reported the results of a single-center, randomized clinical efficacy trial of 433 patients who had initial negative workups for ACS and were randomized to stress echocardiography or exercise stress testing. The primary outcome was the correct identification of patients at high risk for a composite outcome of cardiac death, nonfatal myocardial infarction, and revascularization. The secondary outcome was the cost of diagnosis. Pretest risk was classified using the TIMI (Thrombolysis In Myocardial Infarction) risk score. Post-test risk was considered low if test results were negative and high if they were positive, regardless of pretest risk. A significantly greater number of patients who underwent stress echocardiography were classified as low post-test risk and therefore eligible for immediate discharge compared to those undergoing exercise stress testing (77% vs. 33%; p < 0.001). Additionally, a stress echocardiogram positive for high risk was more accurate than a positive exercise stress test in predicting this primary outcome (51% vs. 29%; p = 0.01). The rate of normal coronary angiographies in the positive exercise stress group was almost twice as high as in the stress echo group (39% vs. 23%). Significantly more patients undergoing exercise stress testing than patients undergoing stress echocardiography required additional diagnostic tests during the follow-up period (47% vs. 20%; p < 0.001). The mean cost to diagnosis was significantly lower in the stress echo group than in the ergometry group (£326 vs. £495; p = 0.01), highlighting the significant "clinical and cost-effectiveness benefits" of stress echocardiography compared to exercise stress testing in patients with suspected ACS.^286^

In 2021, a task force led by the AHA, ACC, and ASE published a guideline for the assessment and diagnosis of chest pain, establishing that patients with acute, low-risk chest pain do not require additional anatomical or functional imaging studies and can be discharged to outpatient evaluation.^287^ Transthoracic echocardiography (TTE), as mentioned above, can be used to visualize and assist in the differential diagnosis between myriad causes of acute chest pain, including acute aortic dissection, pericardial effusion, stress or catecholamine-induced (Takotsubo) cardiomyopathy, and hypertrophic cardiomyopathy, among others. Visualization of left and right ventricular function and RWMAs allows assessment of CAD risk and can aid in clinical decision-making. In intermediate-risk patients in whom ACS has been ruled out, stress echocardiography can be used to ascertain the presence and severity of ischemic heart disease, as well as for risk stratification purposes. For intermediate-risk patients with inconclusive CT angiography, stress echocardiography may also be a diagnostic alternative.^287^

More recently, in 2023, the European Society of Cardiology published guidelines for the management of ACS, in which stress echocardiography was highlighted as an important screening tool in patients with absent or uncertain elevation of biomarkers and a nondiagnostic ECG (Class of recommendation IIA, Level of evidence A)^288^ (Figure 26.1).

**Figure 26.1:** Flow chart adapted from the 2023 European Society of Cardiology Acute Coronary Syndrome Management Guidelines.^288^

Therefore, stress echocardiography in the ED is a valuable tool for the diagnosis and management of patients with suspected ACS, especially those at intermediate risk, with a nondiagnostic ECG, and no cardiac enzyme elevation. However, it is important to consider the limitations of the technique and ensure that physicians are well-trained in its performance. It is also important that the laboratory logistics be set up to perform the test quickly, preferably within the first 24 hours, ensuring the cost-effectiveness of the method as observed in different clinical trials.

## 27. Stress Echocardiography in the Heart Transplant Recipient

Stress echocardiography is a diagnostic technique that can be used to reveal inducible myocardial ischemia in heart transplant recipients. Development of the atherosclerotic coronary artery disease known as cardiac allograft vasculopathy (CAV) is the leading cause of graft failure and death during the first year after heart transplantation.

Coronary angiography via cardiac catheterization has been routinely used for many years to assess CAD in the cardiac allograft, but it is an invasive procedure and carries a higher risk of morbidity in heart transplant recipients.

SE is capable of identifying CAV and has recognized prognostic value for this purpose. A normal stress echocardiogram with no RWMAs is sufficient to postpone invasive investigations.^289^

It has been reported in the literature that, due to surgical denervation of the cardiac allograft, some patients attain their optimal heart rate at low doses of dobutamine during SE. This occurs because heart transplant recipients exhibit an increased chronotropic response to beta-adrenergic stimulation. In other cases, usually in elderly recipients, add-on atropine will be required despite the maximum dose of dobutamine.^290^

Dobutamine has been the most widely used stressor agent in the post-transplant population, with a sensitivity of 70%–80% for detecting CAV.^291^ Due to the blunted HR response to exercise secondary to denervation in heart transplant recipients, exercise stress protocols have very low sensitivity in these patients (15%–33%).^292^

A study by Bacal et al.^293^ found that positive stress echocardiography was an independent predictor of cardiac events and/or death at 4-year follow-up. On the other hand, a negative dobutamine stress echocardiogram was indicative of low risk for CAV and a low incidence of adverse cardiac events.^294^

Dobutamine stress echocardiography is the most recommended method for routine surveillance of CAV during the first 5 years after heart transplantation, considering the high prevalence of this condition in cardiac graft recipients.^295^

## 28. Stress Echocardiography in Special Subgroups (Left Bundle Branch Block, Right Bundle Branch Block, and Atrial Fibrillation)

### 28.1. Left Bundle Branch Block

Left bundle branch block (LBBB) is a common condition with various etiologies, and is challenging from a diagnostic standpoint. LBBB affects myocardial contraction, LV diastolic filling, and coronary perfusion to varying degrees depending on the severity of the block and the underlying cardiac pathology.^296^

Approximately 2% of patients referred for exercise testing present with fixed or intermittent LBBB.^297^ The prevalence of CAD in patients with LBBB ranges from 30% to 50%.^298^ In the presence of LBBB, the ECG is not interpretable for evaluation of ischemia; in these cases, imaging is required for noninvasive investigation of CAD.

Altered electrical activation interferes with septal wall movement, which can range from normal to paradoxical. In the presence of a QRS complex with a duration < 150 ms, wall thickening is often normal and contractility is preserved. The paradoxical septal wall motion pattern is more commonly found when the QRS complex duration exceeds 150 ms and/or when septal fibrosis is present.

Despite the greater difficulty in interpreting septal motility due to abnormal wall movement, SE is the best option for diagnosing CAD in this scenario.^44,298^ It is a more specific modality than myocardial perfusion scanning,^44,299^ with good sensitivity, although this sensitivity is lower for evaluating the LAD territory if septal dyskinesia/dyssynchrony is seen on baseline (resting) echocardiography.^298^

The prognostic value of SE in patients with LBBB is excellent, especially in those without prior myocardial infarction. Cortigiani et al. evaluated the predictive and prognostic value of pharmacological SE in patients with LBBB. In this study, two echocardiographic variables were associated with an increased risk of death from cardiac causes: the resting wall motion score and the change in wall motion score at peak stress. Five-year survival was 77% in the group of patients who tested positive for myocardial ischemia and 92% in the group with a normal test (p = 0.02).^300^

The diagnostic and prognostic value of SE can be improved by adding simultaneous assessment of coronary flow velocity reserve in the LAD^301^ or assessment of myocardial perfusion with ultrasound enhancing agents.^302^

### 28.2. Right Bundle Branch Block

Right bundle branch block (RBBB) is a common finding in the healthy general population; however, it can also be pathological, caused by conditions affecting the right branch of the bundle of His. Possible causes include trauma, structural changes, infiltrative diseases, infections, myocarditis, and CAD.

RBBB is present in approximately 2% to 3% of patients with CAD.^296,297^ In this scenario, its presence is an independent predictor of all-cause mortality.^303^ The onset of RBBB in the context of ACS in particular has been significantly associated with increased in-hospital mortality, even after primary percutaneous coronary intervention.^304^ SE is an excellent diagnostic modality for investigating CAD in this setting, as RBBB typically does not affect regional wall motion. Furthermore, it provides an efficient prognostic stratification to add-on simple resting ECG parameters, such as left anterior fascicular block. In patients with suspected CAD undergoing SE, co-occurrence of RBBB and left anterior fascicular block has been shown to be an independent predictor of mortality.^305^

### 28.3. Atrial Fibrillation

The prevalence of atrial fibrillation (AF) is very low among young people (<1% in the population under 40 years of age) but increases with age, affecting up to 17% of individuals over 80 years old.^306^ CAD is one of the cardiovascular conditions most commonly associated with AF.

Shortening of the diastolic phase in the setting of AF can cause false-positive results during exercise testing, since subendocardial perfusion is compromised. In this context, SE is an effective approach. Generally, vasodilator stress is better tolerated by these patients than dobutamine stress. Nevertheless, despite the increased chronotropic response to dobutamine infusion in patients with AF compared to patients in sinus rhythm, DSE has good diagnostic value in these patients. Hobday et al.^307^ demonstrated that a higher percentage of patients with AF attained their target heart rate and required lower doses of atropine compared to patients in sinus rhythm. In the same study, positive results for myocardial ischemia occurred at similar rates among patients with AF and sinus rhythm, and the use of beta blockers was also similar.^307^ Atropine, on the other hand, should be used sparingly in patients with AF due to the risk of significant increases in heart rate, potentially requiring hospitalization.

New onset of AF during SE is a more common complication in patients with an arrhythmogenic substrate when dobutamine is used as the stressor agent. In a review of 26 studies on the complications of DSE, the incidence of AF was 0.9%.^308^ In a recent retrospective assessment of 4,917 consecutive patients who underwent DSE, the main factors associated with onset of AF during the test were a history of paroxysmal AF (p = 0.02) and advanced age, with an incidence of 4% in patients aged over 80 years (p < 0.0001).^309^ Most patients revert to sinus rhythm within the first hour after onset of AF,^310^ but administration of a beta blocker such as metoprolol is usually required.

## 29. Stress Echocardiography for Preoperative Assessment Before Vascular Surgery, in Women, and in Older Adults

### 29.1. Preoperative Assessment Before Vascular Surgery

Patients undergoing vascular surgery have a high risk of cardiovascular events during the perioperative period. This can be attributed not only to the clinical characteristics of these patients, but also to the presence of comorbid CAD.^311,312^ Furthermore, the nature and complexity of vascular interventions play a relevant role in this scenario.^313^

Therefore, preoperative stratification is paramount in these patients. The goal is to identify those who are more susceptible to cardiovascular events, enabling implementation of measures to reduce morbidity and mortality. In this context, stress echocardiography (physical or pharmacological) stands out as a noninvasive, widely available, low-cost method.

SE can be used to assess functional capacity and the hemodynamic response to exertion. However, the presence of peripheral vascular disease and low functional capacity may compromise the effectiveness of exercise testing; therefore, pharmacological stress with dobutamine or dipyridamole is recommended in this subgroup of patients. The choice of pharmacological agent should be guided by availability, local experience, mechanism of action, and pre-existing vascular diseases.

DSE has proven useful and safe, and can even be used safely in patients with abdominal aortic aneurysm.^314^ Vasodilators (adenosine or dipyridamole) can also be considered a viable alternative which provides good accuracy.^312^ However, their use is contraindicated in patients with severe bilateral carotid artery involvement.^313^

Patients with a positive stress echocardiogram have a higher incidence of cardiovascular events. Pharmacological stress echocardiography has excellent negative predictive value, in the 90–100% range, while its positive predictive value is lower, ranging from 25–45%.^312^ The most significant predictors of adverse postoperative events in this population are presence of ischemia and a history of congestive heart failure. The heart rate at which ischemia develops during DSE can be used to stratify patients into low, intermediate, and high risk for postoperative myocardial infarction and death.^315,316^

The onset of new stress-induced RWMAs, whether extensive (3 or more segments) or limited (1 or 2 segments), in patients with a history of previous myocardial infarction has been characterized as an independent predictor of late cardiac events.^317^

In a meta-analysis of six noninvasive diagnostic modalities for preoperative assessment of patients undergoing vascular surgery, pharmacological stress tests showed higher overall sensitivity and specificity, with DSE demonstrating a trend towards superior diagnostic performance compared to all other modalities.^318^

In multiple studies and meta-analyses, the prognostic value of DSE was at least equal to that of myocardial perfusion scanning in predicting the occurrence of postoperative adverse events.^3,311,319^

### 29.2. Women

Cardiovascular disease (CVD) is the leading cause of death in women worldwide. Appropriate diagnosis and management remain challenging, as female patients may present with atypical symptoms and, therefore, receive inappropriate treatment.^320^

Studies have shown that women are more likely than men to develop angina as the first manifestation of CAD (47% vs. 32%) and are less likely than men to present with an acute myocardial infarction in this setting (6% vs. 10%).^321^

Stress echocardiography is a well-established, highly useful tool for the diagnostic investigation of women suspected of having CAD, as well as for risk stratification of those already diagnosed with coronary heart disease. It has sensitivity, specificity, and prognostic value, with no specific differences between the sexes.^322^ Additionally, the diagnostic accuracy of DSE has been shown to be similar both in women with atypical pain and in cases of angina.^323,234^

A study by Biagini et al.^325^ included 2,276 men and 1,105 women with suspected or known coronary artery disease. All underwent DSE and risk stratification; the outcomes of nonfatal myocardial infarction and cardiovascular death were observed over a 7-year follow-up. The results showed that DSE provides independent prognostic information in men and women alike.

From a technical standpoint, it is important to note that women have a higher incidence of hypotension during DSE. Smaller ventricular chambers with a higher degree of relative hypertrophy can facilitate the development of dynamic LVOTO, leading to hypotension and premature test cessation. Furthermore, in smaller hearts, there is a higher likelihood of left ventricular cavity obliteration, which can mask subtle changes in wall motion.^326^ In addition, chest pain can occur during dynamic LVOTO, raising doubts regarding the presence of myocardial ischemia. Although the protocol is the same, it is essential that not only regional wall motion but also the presence of an intraventricular gradient be assessed in these cases,^327^ since anatomical and hemodynamic factors which may contribute to such an event are more prevalent in this subgroup of patients.

In summary, SE plays a key role in the diagnosis and prognosis of CAD in women.

### 29.3. Older Adults

The population of patients over 65 years of age with suspected CAD is especially vulnerable and is constantly growing. A constant search for the most effective diagnostic approach is needed to ensure optimal treatment and the best possible outcomes.

Physical or pharmacological stress echocardiography emerges as a key diagnostic tool for this population, combining the benefits of cardiac imaging with analysis of cardiovascular function under stress.

In patients over 65 years of age, integration of information from SE (especially the left ventricular end-systolic volume and ejection fraction responses to exercise) with clinical, ECG, and resting echocardiography findings improves the ability to predict cardiac events and all-cause mortality.

A study of 2,632 older adults (56% men) capable of exercise showed that SE provided additional prognostic value over clinical variables alone (age, diabetes, previous myocardial infarction, exercise stress test findings). In exercise stress echocardiography, exercise load was the best predictor of cardiac events and death.^327^

Given that inability to exercise is common in older adults due to the particularities of this age group, pharmacological stress echocardiography, particularly DSE, has been identified as a viable alternative. Biagini et al.^329^ studied 1,434 patients aged > 65 years (mean age 72 ± 3 years) who underwent DSE for evaluation of CAD. Presence of RWMAs was considered an independent predictor of all-cause mortality and adverse outcomes, while a normal examination correlated with a low incidence of cardiac events. Cortigiani et al.^330^ showed that the rate of events in octogenarians was 4.5 times higher than in the younger elderly (between 65 and 69 years of age).

The safety and accuracy of SE mean it has a crucial role to play in the diagnostic and prognostic evaluation of older adults, contributing significantly to the appropriate management of this patient population.

## 30. Stress Echocardiography in Pediatrics: Indications and Protocols

While stress echocardiography is a well-established technique in cardiology for adult patients, the low prevalence of conditions such as CAD, valvular heart disease, and cardiomyopathy in children has led to a scarcity of published data on this modality and to its unpopularity in pediatric practice. SE is not yet as widely used in pediatric patients as it is in adult patients, due to the inherent difficulties in performing the examination. Few centers offer this modality for pediatric patients, and there is little literature to support it.

The use of stress greatly enhances echocardiographic assessment, providing data in a physiological environment that more closely resembles the typically active state of this age group.^331^

As in the adult population, the two key questions that SE seeks to answer are: 1) is there inducible myocardial ischemia? and 2) are there hemodynamically significant changes?^332^

As in adults, the stressor can be physical (such as treadmill, bicycle, or reclining ergometer exercise) or pharmacological (with dobutamine and, occasionally, dipyridamole).

Exercise stress echocardiography (ESE) is well tolerated in the pediatric population, as it does not require IV access or sedation; however, it can only be performed in children over 6–7 years of age who are able to perform the exercise and are tall enough to walk on a treadmill or pedal a bicycle or reclining ergometer.^332-335^ In both modalities, the modified Bruce protocol is generally used.^332-335^ The James protocol can also be used on a bicycle or reclining ergometer.^333^ There is no age limit for DSE; however, in children under 8 years old, deep sedation and/or general anesthesia is essential, and even in older children, conscious sedation may be required.^333^ The protocols used are similar to those for adults.^333-336^ Generally, dobutamine infusion starts at 5 mcg/kg/min and is increased in 3-minute intervals to 10, 20, 30, 40 and, eventually, up to 50 mcg/kg/min. If the target maximum HR (85% of the maximum HR for age) is not attained and the examination is thus inconclusive, a low dose of atropine (0.01 mg/kg) is administered at peak dobutamine stress. Even so, the target HR for this population is very high.

Alternatively, continuous dobutamine infusion at low to moderate doses (5 to 20 mcg/kg/min) may be used to assess cardiac contractile reserve.^333,337^ The standard dose of dipyridamole is 0.84 mg/kg of body weight, as in the adult protocols already described in previous sections.

Adverse effects are similar to those observed in adult patients.^333-336^ Complications such as sustained ventricular arrhythmias are rare; however, there is insufficient data in the literature regarding these complications in pediatric patients.^333^

The main indications for SE in pediatrics are: assessment of myocardial ischemia, early detection of myocardial dysfunction, dynamic assessment of gradients in hypertrophic cardiomyopathy, assessment of right ventricular function, pre-exercise assessment in patients with congenital heart disease,^332^ and after heart transplantation and chemotherapy.^333,338^

Tables 30.1 and 30.2 describe these indications, with a summary of recently published articles.

**Table 30.1:** Indications for stress echocardiography in children, adolescents, and young adults

Indication	No. pts	Age	Stressor	Findings
**Kawasaki disease**				
Pahl et al. (1995)^335^	28	10.7 years (median)	ESE (treadmill)	ESE detected RWMAs and was superior to ECG alone for this purpose.
Noto et al. (1996)^336^	50	13.6 years (mean)	DSE	DSE was safe and accurate for diagnosis of coronary artery stenosis in children.
Noto et al. (2014)^340^	58	23.8 years (mean)	DSE	DSE was useful for risk stratification and prognostic assessment.
Cardiac allograft vasculopathy Perez et al. (2022)^334^	95	16.3 years (median)	ESE (treadmill)	Abnormal ESE was associated with ↑ risk of CV events and death, while a normal ESE was associated with CV event-free survival.
**AAOCA** Brothers et al. (2007)^341^	24	24 years (median)	ESE (treadmill and bicycle)	ESE was useful for assessing ischemia and guiding postoperative care.
**Status post arterial switch (Jatene) procedure** Chen et al. (2013)^342^	32	16.2 ±2.1 (mean ± SD)	ESE (bicycle)	ESE demonstrated decreased contractile reserve in adolescents and young adults in the postoperative period.
**Childhood cancer survivors** Ryerson et al. (2015)^343^	80	13 years (mean)	ESE (bicycle)	Patients showed improvement in diastolic and systolic function during exercise, suggesting an ability to compensate for mild cardiac dysfunction.
**Status post Ross procedure** Pauliks et al. (2012)^339^	26	14.9 years (mean)	ESE (treadmill)	ESE was feasible in children, most of whom showed capacity for normal exercise.
**Status post ToF repair** Bhatt et al. (2019)^344^	29	18.3±4.83 ↓performance during exercise 12.8 ± 3.26 ↑performance during exercise (mean ± SD)	ESE (treadmill)	Preserved chronotropic response, more than right ventricular contractile reserve, was an important factor for exercise performance.
**Obstructed right ventricular-to-pulmonary artery conduit** Hasan et al. (2012)^345^	40	17 years (median)	ESE (treadmill)	ESE detected right ventricular and conduit dysfunction at peak exercise.
**LVOT gradient in HCM** El Assaad et al. (2020)^346^	91	12 years (median)	ESE (treadmill and bicycle)	ESE proved safe and effective in identifying patients at low risk for CV events.

HCM: hypertrophic cardiomyopathy; CV: cardiovascular; SD: standard deviation; DSE: dobutamine stress echocardiography; ESE: exercise stress echocardiography; AAOCA: anomalous aortic origin of the coronary arteries; LV: left ventricle; LVOT: left ventricular outflow tract; ΔP: pressure gradient.

**Table 30.2:** Indications for stress echocardiography in children, adolescents, and young adults (summarized)

Indications for stress echocardiography in pediatrics	Suspicion of coronary artery disease: Kawasaki disease, cardiac allograft vasculopathy, surgical procedures involving coronary translocation (such as arterial switch procedure for transposition of the great arteries), anomalous origin or course of the coronary arteries, pulmonary atresia with intact ventricular septum, hyperlipidemia, insulin-dependent diabetes mellitus, supravalvular aortic stenosis
	Status post chemotherapy: Anthracyclines
	Assessment of gradients: in hypertrophic cardiomyopathy or aortic and pulmonary stenosis
	Assessment of hemodynamic and myocardial response in specific conditions: pulmonary hypertension, dilated cardiomyopathy, congenital heart disease, mitral and aortic regurgitation

For specific indications, protocols are adjusted to include, e.g., Doppler imaging of the atrioventricular valves to study aortic valve regurgitation or measure gradients in outflow tracts.

In pediatric patients, unlike in adults, there is insufficient data on the prognostic value and clinical impact of SE in the dynamic assessment of gradients in aortic and pulmonary stenosis.^333,339^

Finally, SE is still a developing field in pediatric cardiology. Its integration with tissue Doppler and strain imaging has allowed for a more quantitative analysis of regional and global systolic and diastolic function in this setting, but it is still in its early stages. Cooperation between adult and pediatric cardiologists is essential for the development and implementation of protocols tailored to the specific needs of children and for this type of examination to become more widespread in pediatric practice.

## 31. Advantages and Disadvantages of Stress Echocardiography in Pediatrics

Both exercise stress echocardiography and pharmacological stress echocardiography can be used in pediatric patients.^33,99,347^ However, the physician should consider the advantages and disadvantages of each approach in this population (Table 31.1).

**Table 31.1:** Advantages and disadvantages of each stress echocardiography modality in the pediatric population

Imaging Modality	Advantages	Disadvantages	Uses
Exercise Stress Echocardiogram (ESE)	No intravenous puncture required No sedation required Able to obtain other data – ECG testing and potentially VO_2_	Extra equipment Patient must be able to perform exercise on ergometer	
Treadmill ESE	Resembles daily activities Higher Max VO_2_ May be easier to elicit ischemia	Motion artifact Rapid heart rate recovery in children Need to transfer to bed	Ischemia Assessment Outflow obstruction Contractile reserve
Cycle ESE	Multiple time points for imaging Less motion artifact Doppler imaging may be possible	Cooperation in younger children Lower heart rate Lower VO_2_	Valvar regurgitation Atrioventricular valve stenosis Pulmonary hypertension Diastolic dysfunction
Pharmacologic Stress Echo	Less lung interference No need for transfer to bed Limits heart rate recovery issues	Intravenous line placement	
Dobutamine stress echo	Affects both heart rate and blood pressure	Takes around 20 minutes Side effect profile: ventricular arrhythmias, hypertensive emergency, nausea/vomiting	Dynamic coronary obstruction Low dose – low-flow, low-gradient aortic stenosis
Vasodilator stress echo	Short acting	Side effect profile: wheezing, seizure like activity, hypotension	Fixed coronary obstruction

Exercise SE offers some advantages over pharmacological SE in the pediatric population. First, there is no need to obtain IV access, which minimizes patient distress. Second, there is no need for sedation, which may be necessary for the longer study periods associated with pharmacological stress. Finally, the echocardiographer can often choose between two ergometers for the stress echocardiogram: a treadmill or a cycle ergometer.^3,331,341,346,348-350^

Exercise SE also has several disadvantages, however. First, the additional equipment required can be limiting for some echo labs, especially since there is a need for a pediatric transducer and appropriate stress testing software. Furthermore, exercise echocardiography requires significant cooperation from the pediatric patient, regardless of the ergometer used. Pulmonary artifacts due to a physiological increase in respiratory rate may restrict echocardiographic windows during exercise in this subgroup of relatively uncooperative patients. Finally, the rapid recovery of heart rate in children may limit the ability to obtain adequate images at peak stress.

There are specific advantages and disadvantages to each type of ergometer as well. Treadmill stress very closely resembles both activities of daily living and physical education workouts. It also usually allows attainment of a higher VO_2_, which is excellent for the purposes of the examination, as is the higher double product. The main disadvantage of treadmills is that the patient must be able to transfer quickly and safely from the treadmill to the echocardiography bed or couch, due to the rapid drop in heart rate (post-exercise recovery) in the pediatric population^3^ and the examiner thus needs to obtain these images within a very brief window of time, which is not always easy, especially in children.

Reclining ergometers allow for imaging at various time points during the stress test with continuous monitoring. Cycle ergometers may allow for a gradual and more appropriate use of color or spectral Doppler in this subpopulation, which can be advantageous when assessing valve stenosis or regurgitation and pulmonary hypertension,^3^ intraventricular obstruction in hypertrophic cardiomyopathy, and gradients in aortic stenosis.^346,350^ Furthermore, cycle ergometers tend to limit movement artifacts. However, cooperation can be more difficult in younger patients because they must pedal continuously and at a constant pace. Typically, lower VO_2_max and lower heart rates are achieved with cycle ergometers compared to treadmills.^3^

Pharmacological stress echocardiography can be performed in children.^3,351,352,340^ However, the main disadvantage is the need for IV access to administer the stressor agent. Advantages include less pulmonary interference, assessment at various stages of the test, and no need for transfer to a bed for imaging, thus mitigating the issues associated with rapid heart rate recovery in children, since echocardiographic monitoring is continuous during pharmacological stress.

Two main pharmacological stressors are used in this population: dobutamine and vasodilators.^3^ Dobutamine mimics exercise by increasing heart rate and blood pressure to levels close to those attained during maximal exercise. In children, it is mainly used to induce ischemia and RWMAs in areas where there may be dynamic obstruction of coronary arterial flow, in the assessment of anomalous aortic origin of the coronary arteries, and in heart transplant recipients.^341,349,351,352^

Vasodilators, such as dipyridamole, adenosine, or regadenoson, dilate normal coronary arteries, causing ischemia and regional wall motion abnormalities in areas with fixed coronary obstruction.^3^ In the pediatric population, they can be particularly useful in the assessment of Kawasaki disease and reimplanted coronary arteries. Adverse effect profiles vary depending on the vasodilator used, as described in previous sections of this guideline.

Although many stress echocardiography options are available, it remains underutilized in children. Physicians who understand the advantages and disadvantages of each modality can select the most appropriate option for each clinical scenario.

## 32. Prognostic Value of Stress Echocardiography

Eliciting myocardial ischemia by increasing heart rate to ≥ 85% of predicted for age during SE, whether through physical exercise or intravenous dobutamine infusion, is an effective method to guide clinical management and prognostication in ischemic heart disease, and this has been widely discussed in the literature. The normal response is an increase in contractility of all 16 (or 17) ventricular segments.^202^ The response of each segment is assigned a score of 1 if normal or hyperkinetic, 2 if mildly or moderately hypokinetic, 3 if severely hypokinetic, 4 if akinetic, or 5 if dyskinetic. To streamline assessment, the following scoring system is now more commonly used: 1 = normal or hyperkinetic, 2 = hypokinetic (regardless of severity); 3 = akinetic; and 4 = aneurysm. The sum of the individual segment scores allows calculation of the WMSI, dividing all visualized segments by the 16 or 17 analyzed segments. The only possible normal result is 16/16 = 1 or 17/17 = 1, which denotes normal global contractility. An example abnormal score: moderate hypokinesis in 2 segments (score = 2 each) adds up to 4, plus akinesis in one segment (score = 4) adds up to 8. The 16-segment model was used, so divide 16 by 8; the WMSI index is 2, i.e., abnormal.

In a study of 3,121 patients^202^ divided into three risk groups according to WMSI and followed for 3 years, a WMSI of 1 (low risk) was associated with an event rate of 0.8% per year, a WMSI of 1.1-1.7 (intermediate risk) with an event rate of 2.6% per year, and a WMSI > 1.7 (high risk) with an event rate of 5.5% per year.

Another study with 1,560 patients sought to correlate peak WMSI with LV ejection fraction.^353^ Patients were divided into three groups with a 3-year follow-up period. Event rates were 0.7% per year in the low-risk group, 2.0% per year in the intermediate-risk group, and 4.4% per year in the high-risk group. The events of interest were myocardial infarction, death, or other cardiovascular complications.

In another study, 2,575 individuals with a negative SE at peak stress^354^ were classified into two groups: those without RWMAs at rest or during maximal exercise and those with pre-existing RWMAs (due to infarction) which did not worsen during exercise. The latter all had CAD; the criterion for abnormality was the development of a new ≥ 50% coronary lesion within 1 year of examination. All-cause mortality in those with negative results at 1 year was 1.1%, whereas in those with preexisting CAD it was 3.1%. Predictors of new coronary lesions within 1 year were male sex, diabetes, previous CAD, and nonischemic but abnormal stress echocardiography. These patients should be monitored due to their increased risk of morbidity and mortality, even if the latest SE is negative.

In a study of 609 patients with LBBB^355^ who underwent ESE with a follow-up period of almost 5 years, the WMSI was obtained at rest and after exercise. The difference (Δ) in WMSI between the two time points was 29%. The mortality rate was 25% for patients with ischemia and 13% for those without ischemia.

Major adverse cardiac events occurred in 18% vs. 10%, with ΔWMSI considered an independent predictor.

SE has greater value than other clinical parameters.

The main causes of test cessation are onset or worsening of RWMAs, angina, ischemic ECG changes, significant arrhythmias, significant hypertension or hypotension, limiting dyspnea or fatigue, and severe tremor. Test cessation for any of these reasons or because the end of the protocol was achieved without attaining the target HR or any RWMA endpoints must be noted in the report.

Echo stress guidelines discussing the risks and future prognosis of this method were recently published elsewhere.^3,7^

## 33. Novel Applications of Stress Echocardiography: After Radiation Therapy to the Chest and in Vasospasm

Radiation therapy (RT) is one of the cornerstones of cancer treatment. High-dose irradiation of the chest is commonly used in the treatment of lung, esophageal, and breast cancers and lymphomas. Due to its anatomical proximity to these structures, the heart invariably receives doses of radiation high enough to induce cardiotoxicity. Studies show that RT accelerates the progression of coronary atherosclerosis, valvular calcification, and pulmonary and pericardial fibrosis.

RT-related cardiovascular toxicity is progressive, typically developing 5 to 10 years after initial treatment, and can cause CAD and HF at an incidence up to six times higher than in the general population. The latency period between RT and onset of CAD ranges from a few years to several decades, depending on pre-treatment CV risk, patient age, cumulative radiation dose, and additional treatments, such as chemotherapy.^356^ A thorough assessment of CV risk, including widely available risk scores and evaluation for preexisting cardiac abnormalities, is imperative in these patients.

Considering that patients undergoing RT commonly receive a large cumulative radiation load, the use of imaging methods that do not expose them to extra doses is a priority.^357,358^

Stress echocardiography to search for changes in regional wall motion and left ventricular function has been the most widely used post-thoracic RT protocol in the last 40 years.

In a recently published article, a study group led by Dr. Eugenio Picano suggested an expanded ABCDE protocol, including steps F, G, P, and R, to face the complexity of the multifaceted damage caused by radiation exposure. This expanded protocol included assessment of the mitral valve, its progressive calcification, and the repercussions of this damage under stress (step F); assessment of the progression of aortic valve calcification and transaortic gradients (step G); testing for pulmonary vascular resistance (step P); and alterations in right ventricular function (step R).^359^

Additional information may be relevant and its collection should be encouraged whenever possible. A thorough pre-examination assessment and a targeted stress test, with priority given to addressing the diagnostic hypothesis and based on sound clinical judgment, appears to be the most appropriate approach.

Coronary vasospasm is one of the leading causes of dynamic stenosis of the coronary arteries, with the potential to trigger angina, unstable angina, acute myocardial infarction, syncope, and sudden death. Its diagnosis remains a major clinical challenge. Invasive provocative testing with ergonovine has been reported in several studies, but with some reservations, as mentioned in previous sections. Invasive testing exposes the patient to radiation and potentially nephrotoxic doses of contrast agents; furthermore, ergonovine is not commercially available in several countries. Noninvasive protocols using ergonovine, adenosine, or dobutamine have been described.^360^ However, the most promising protocol has been a combination of hyperventilation (5 minutes at a respiratory rate of 25 breaths per minute) and bicycle exercise (workload increasing in 25W increments every 2-3 minutes). These two steps can clarify whether angina is due to vasospasm leading to RWMA (step A) or to coronary microvascular dysfunction (step D). Large-scale validation studies and efficacy and safety testing of this protocol are still needed. Additional data should be published after completion of the Stress Echo 2030 trial.^261^

## 34. Stress Echocardiography with the ABCDE Protocol

Cardiac functional stress imaging is based on the detection of RWMA using two-dimensional echocardiography and is incorporated into clinical guidelines. However, it underutilizes the unique versatility of the technique, which is ideal for describing different functional abnormalities that may underlie the same regional wall movement response during stress. The ABCDE protocol, devised by Dr. Eugenio Picano in Italy for the Stress Echo 2020 multicenter study and, subsequently, the Stress Echo 2030 study, aims to expand the use of stress echocardiography to a range of applications in which it can add relevant information^261^ (Figure 34.1).

**Figure 34.1:** The "heart" of the Stress Echo 2030 ABCDE protocol.^261^

Five parameters converge conceptually and methodologically in the ABCDE protocol to assess multiple vulnerabilities of the ischemic patient.^261^

The five steps of the ABCDE protocol are: 1) Step A: regional wall motion/thickening (already widely used); 2) Step B: pulmonary ultrasound for B-lines to assess extravascular pulmonary congestion; 3) Step C: left ventricular contractile reserve by two-dimensional volumetric echocardiography; 4) Step D: coronary flow velocity reserve in the mid-distal left anterior descending coronary artery with pulsed Doppler; and 5) Step E: assessment of heart rate by single-lead ECG.

The ABCDE stress echocardiogram provides information on the so-called five functional reserves: epicardial flow (A); diastolic reserve/congestion (B); contractile reserve (C); coronary microcirculation (D); and chronotropic reserve (E) (Figure 34.2).

**Figure 34.2:** Integration of four main stress echo images. The four pathophysiological objectives are: assessment of epicardial stenosis (A = asynergy), pulmonary congestion (B = B lines), left ventricular myocardial function (C = contractile reserve), and coronary microvascular function (D = Doppler RVFC), E = chronotropic reserve, which can be additionally assessed.^261^ WMSI: Wall Motion Score Index; LVCR: Left Ventricular Contractile Reserve; CFVR: Coronary Flow Velocity Reserve; HR: Heart Rate.

The new format is more comprehensive and allows for better functional characterization, risk stratification, and personalized adaptation of prescribed therapies.

The ABCDE protocol^261^ It is a functional test suitable for all types of stress and all patients, and extends beyond CAD. It addresses the need for sustainability in healthcare, as it requires only universally available technology and is low-cost, radiation-free, and virtually harmless.

### 34.1. The ABCDE Protocol

- **A for Asynergy:**
  This is based on the presumptive stress-induced myocardial contractility alteration in the presence of flow-limiting coronary stenosis. The oxygen supply-demand imbalance leads to a reduction in myocardial radial thickening, a parameter sometimes limited by its semi-quantitative and subjective nature. Operator dependence can be minimized (but not eliminated) by specialized training and the adoption of measures such as accreditation and quality control of echocardiography laboratories.
  Assessment of regional wall motion indicates that the greater the number of affected segments (universally defined), the worse the prognosis. This parameter is centered on coronary artery disease.
- **B for B-Lines:**
  Increased left atrial pressure forms the basis for a myriad of conditions. Its pathophysiological consequence concerns fluid outflow into the pulmonary pericapillary interstitium as a hydrostatic substrate. In its acute form, this fluid can be detected by ultrasound in the form of echo lines that reverberate it. During physical SE, the induction of physiological processes that potentially cause the homeostasis threshold imposed by the disease to be exceeded is a parameter that has significant prognostic value.^361^
- **C for Contractility:**
  Contractility is the inherent ability of the myocardium to contract regardless of changes in preload or afterload.
  During SE, the left ventricular end-systolic volume (ESV) progressively decreases, resulting in higher arterial pressure peaks and increased load. The ratio between systolic blood pressure (SBP) and ESV predicts the left ventricular emptying capacity.^362^
  Left ventricular elastance measures the contractile reserve by comparing LV performance at rest and under stress conditions. This metric can be obtained by dividing the SBP/ESV ratio during stress by the ratio at rest.
  Validated values are available for physical and dobutamine stress (both are > 2.0). When using vasodilators, a ratio > 1.1 indicates appropriate contractility.
- **D for Coronary Artery Doppler:**
  Coronary flow can be affected by epicardial conditions, such as stenosis, and myocardial dysfunction.
  In the first scenario, at a given flow measurement point, a limitation may occur due to compromised blood flow downstream of the obstruction under stress situations.
  In the second, several conditions may act to prevent adequate blood flow, such as perivascular hypertrophy, endothelial injury, dysfunction caused by inflammation, and atherosclerosis.
  Coronary flow reserve on echocardiography is measured by Doppler analysis of the velocity ratio in the medial or distal portion of the left anterior descending artery under stress and resting conditions. Normal values in any SE modality are > 2.0.
- **E for Electrocardiogram:**
  Heart rate variability in response to stress can provide information about the autonomic function of the cardiac conduction system, serving as a marker of such function during the examination. This parameter, heart rate reserve, is obtained by dividing the maximum heart rate achieved by the resting heart rate.
  It is classified as positive if the ratio is < 1.80 for physical or dobutamine stress and < 1.22 when vasodilators are used.
  Beta blockers reduce heart rate under both rest and stress conditions, but have no impact on this ratio for prognostic purposes.^361^

When added to conventional SE, the ABCDE protocol results in a broader spectrum of variables being evaluated. Together, they allow for better risk stratification and help define more accurate diagnoses.

Patients with microvascular disease and diastolic dysfunction sometimes have atypical presentations and may benefit from this new protocol, as it expands the diagnostic power of traditional SE.^363^

The ABCDE protocol proposes that the well-established tool of stress echocardiography be used to its fullest extent, in a simplified manner, to provide a vast range of information.

## 35. Recommendations for the Management of Stress Echocardiography

**Table:** STRESS ECHOCARDIOGRAPHY IN KNOWN OR SUSPECTED CHRONIC ISCHEMIC HEART DISEASE

Recommendations	Strength of Recommendation	Certainty of Evidence
For the diagnosis of chronic ischemic heart disease in patients with an intermediate pre-test probability. In the presence of angina symptoms in patients with prior stent implantation or coronary revascularization. In cases of moderate coronary obstruction on coronary CT angiography or invasive coronary angiography: assessment of functional significance. For post–myocardial infarction risk stratification. For the investigation of dyspnea (angina equivalent).	**STRONG**	**HIGH**

**Table:** STRESS ECHOCARDIOGRAPHY IN THE EMERGENCY SETTING: ACUTE CHEST PAIN

Recomendações	Strength of Recommendation	Certainty of Evidence
In acute chest pain in patients at intermediate risk (without ECG abnormalities and/or with negative biomarkers). In patients with suspected anginal chest pain and negative or mildly elevated troponin, for patient risk stratification, provided there are no electrocardiographic changes or recurrence of pain.	**STRONG**	**MODERATE**

**Table:** STRESS ECHOCARDIOGRAPHY IN VALVULAR HEART DISEASE

Recomendações	Strength of Recommendation	Certainty of Evidence
Low-dose dobutamine stress echocardiography in patients with low-flow, low-gradient (classical) aortic valve stenosis with LVEF < 50%, to confirm stenosis severity. Exercise stress echocardiography may be used to assess the hemodynamic impact of the valvular lesion in patients with discrepancy between symptoms and resting echocardiographic findings.	**STRONG**	**HIGH**

**Table:** DIASTOLIC STRESS ECHOCARDIOGRAPHY

Recommendations	Strength of Recommendation	Certainty of Evidence
Exercise stress echocardiography, preferably using a supine cycle ergometer or treadmill, in patients with unexplained dyspnea and: – Grade 1 diastolic dysfunction; – Intermediate H_2_FPEF or HFA-PEFF scores (2–5 points and 2–4 points, respectively).	**STRONG**	**HIGH**

**Table:** STRESS ECHOCARDIOGRAPHY WITH ULTRASOUND ENHANCING AGENTS (UEAs) FOR ENDOCARDIAL BORDER DEFINITION

Recommendations	Strength of Recommendation	Certainty of Evidence
Stress echocardiography with UEAs when two or more contiguous myocardial segments are not visualized (in the apical view), to improve diagnostic accuracy.	**STRONG**	**HIGH**

**Table:** STRESS ECHOCARDIOGRAPHY WITH ULTRASOUND ENHANCING AGENTS (UEAs) FOR MYOCARDIAL PERFUSION ANALYSIS

Recomendações	Strength of Recommendation	Certainty of Evidence
Myocardial perfusion assessment to increase accuracy in identifying obstructive coronary artery disease and for the evaluation of myocardial viability.	**WEAK**	**HIGH**

**Table:** ECO ESTRESSE EM PEDIATRIA

	Strength of Recommendation	Certainty of Evidence
Appears to be advantageous for the assessment of valvular stenosis or regurgitation and dynamic obstruction of the left or right ventricular outflow tracts. Pulmonary arterial hypertension. Assessment of myocardial ischemia (AAOCA, ALCAPA, Kawasaki disease, and in patients undergoing cardiac surgery with coronary manipulation, such as after the Jatene procedure). Post-heart transplantation for ischemia assessment	**WEAK**	**LOW**

AAOCA: Anomalous aortic origin of the coronary arteries; ALCAPA: Anomalous origin of the left coronary artery from the pulmonary artery.

## Data Availability

The data underlying the text of the Position Statement are included in the manuscript.

## List of abbreviations and acronyms

ACS: Acute coronary syndrome
AF: Atrial fibrillation
AS: Aortic stenosis
AV: Atrioventricular
AVA: Aortic valve area
BP: Blood pressure
CAD: Coronary artery disease
CAV: Cardiac allograft vasculopathy
CCDC: Chronic Chagas disease cardiomyopathy
CFR: Coronary flow reserve
CMR: Cardiac magnetic resonance
CO: Cardiac output
CR: Contractile reserve
CT: Computed tomography
CVD: Cardiovascular disease
DBP: Diastolic blood pressure
DCM: Dilated cardiomyopathy
DF: Diastolic function
DSE: Dobutamine stress echocardiography
ECG: Electrocardiogram
ED: Emergency department
ESE: Exercise stress echocardiography
FAC: Fractional area change
GLS: Global longitudinal strain
GPCR: G protein-coupled receptor
HCM: Hypertrophic cardiomyopathy
HFpEF: Heart failure with preserved ejection fraction
HR: Heart rate
LAD: Left anterior descending artery
LBBB: Left bundle branch block
LFLG: Low flow-low gradient
LMCA: Left main coronary artery
LV: Left ventricle
LVEF: Left ventricular ejection fraction
LVFP: Left ventricular filling pressure
LVOT: Left ventricular outflow tract
LVOTO: Left ventricular outflow tract obstruction
MI: Mechanical index
MINOCA: Myocardial infarction with nonobstructive coronary arteries
MP: Myocardial perfusion
MR: Myocardial reserve
MS: Mitral stenosis
MVD: Mitral valve disease
NIDCM: Nonischemic dilated cardiomyopathy
PAH: Pulmonary hypertension
PAP: Pulmonary artery pressure
PASE: Pacing stress echocardiography
PASP: Pulmonary artery systolic pressure
PDA: Posterior descending artery
PEG: Polyethylene glycol
PET: Positron emission tomography
PPM: Permanent pacemaker
RBBB: Right bundle branch block
RHC: Right heart catheterization
RLS: Regional longitudinal strain
RT: Radiation therapy
RV: Right ventricle
RWMA: Regional wall motion abnormality
SAM: Systolic anterior motion
SBP: Systolic blood pressure
SE: Stress echocardiography
SPECT: Single-photon emission computed tomography
SR: Strain rate
TAPSE: Tricuspid annular plane systolic excursion
TRV: Tricuspid regurgitation velocity
TTE: Transthoracic echocardiography
UEA: Ultrasound enhancing agent
WMSI: Wall motion score index
